# Dissecting glioblastoma risk signatures in the tumor immune microenvironment based on multi-dimensional transcriptomics

**DOI:** 10.1093/gigascience/giag035

**Published:** 2026-03-25

**Authors:** Tengyue Li, Wanqi Mi, Huarui Yan, Yining Ma, Han Jiang, Xiaoxu Yang, Yunpeng Zhang, Congxue Hu

**Affiliations:** College of Bioinformatics Science and Technology, Harbin Medical University, 157 Xuefu Road, Nangang District, Harbin 150081, China; College of Bioinformatics Science and Technology, Harbin Medical University, 157 Xuefu Road, Nangang District, Harbin 150081, China; College of Bioinformatics Science and Technology, Harbin Medical University, 157 Xuefu Road, Nangang District, Harbin 150081, China; College of Bioinformatics Science and Technology, Harbin Medical University, 157 Xuefu Road, Nangang District, Harbin 150081, China; College of Bioinformatics Science and Technology, Harbin Medical University, 157 Xuefu Road, Nangang District, Harbin 150081, China; College of Bioinformatics Science and Technology, Harbin Medical University, 157 Xuefu Road, Nangang District, Harbin 150081, China; College of Bioinformatics Science and Technology, Harbin Medical University, 157 Xuefu Road, Nangang District, Harbin 150081, China; College of Bioinformatics Science and Technology, Harbin Medical University, 157 Xuefu Road, Nangang District, Harbin 150081, China

**Keywords:** glioblastoma, single-cell transcriptomics, spatial transcriptomics, tumor microenvironment, machine learning, prognostic signature, imune escape

## Abstract

Glioblastoma (GBM) is characterized by pronounced tumor heterogeneity and a complex immune microenvironment, contributing to poor patient survival outcomes. In this study, we comprehensively dissected the tumor microenvironment (TME) and uncovered potential molecular mechanisms by integrating single-cell, bulk, and spatial transcriptomic data. Hallmarks of malignancy and cell cycle regulatory pathways were consistently enriched across these modalities, promoting tumor cell proliferation and progression. Using a machine learning algorithm, we identified seven hallmark-related prognostic signatures (HMsig), namely AEBP1, ASF1A, PRPS1, DCC, OPHN1, IL13RA2, and HDAC5—whose predictive importance was validated through SHAP analysis. Ligand–receptor (LR) interaction analysis further revealed that interactions involving OPHN1 were associated with poorer prognosis. Along the pseudotime trajectory of T cell differentiation, immune checkpoint genes (ICGs) LAG3, PDCD1, and HAVCR2 were substantially upregulated. Notably, synergistic transcriptional regulation between tumor-related HMsig genes and ICGs in T cells was identified as a key factor influencing patient survival. Spatial transcriptomic analysis demonstrated the existence of synergistic gene interactions, deciphering the immunomodulatory functions of GBM biomarkers within the TME.

## Introduction

Being a high-grade glioma assigned a World Health Organization (WHO) Grade IV, glioblastoma (GBM) stands as one of the tumors that resist cure most stubbornly. Among primary brain cancers, GBM is the most aggressive and occurs most frequently, with a median survival time of only 14–18 months [1]. Currently, the recurrence rate of GBM patients after treatment exceeds 90%. Only a negligible number of patients are likely to approach a state of cure. It’s important to stress that the outlook for GBM patients is extremely poor. As a result, gaining a deeper understanding of the diversity of the pathological biology of GBM and exploring its potential tumor microenvironment (TME) characteristics is paramount.

Unsatisfactory therapeutic outcomes for GBM may be multifactorial, including the decline of T cell killing function, defects in tumor antigen presentation, and characteristics of the tumor’s physical microenvironment. In brain tumors, there are more resident microglia and macrophages compared to infiltrating T cells [2]. Various studies support the hypothesis that GBM is an immunologically “cold” tumor [3]. Unlike immune hot tumors, which show extensive immune infiltration and active anti-tumor immunity, cold tumors are defined by an absence of these responses and weakened immune defenses. Additionally, the efficacy of most small-molecule chemotherapy drugs is limited by the unique structure of brain blood vessels—the blood–brain barrier (BBB)—which strictly regulates the homeostasis of the central nervous system. The inability of drugs to cross the walls of brain blood vessels is likely a major reason for the poor results in many clinical trials of blood–borne drugs [4]. Moreover, the scarcity of T cells in the TME starkly contrasts with findings in melanoma, lung cancer, and other tumor types [5]. Whether GBM is inherently non-immunogenic or the regulatory mechanisms between tumor cells and immune cells in the TME require further investigation.

The significant heterogeneity observed in GBM is largely driven by its regulation of core hallmarks. More precisely, the control of GBM cell proliferation, self-renewal, and the inactivation of differentiation is mediated by the Wnt, Notch, and TGF-β signaling pathways [6]. The WNT/β-catenin cascade is closely associated with various malignant tumors. In normal cells, the Wnt signaling pathway is typically in an inactive state and is highly conserved [7]. Abnormal activation of the Wnt signaling promotes cancer cells to escape immune surveillance, inhibits T cell infiltration, and mediates the anti-tumor immune response [8]. This pathway has recently become an important determinant in the occurrence and development of GBM. GBM is also involved in alterations of multiple metabolic hallmarks, such as glycolysis, oxidative phosphorylation (OXPHOS), and hypoxia [2]. GBM utilizes various unconventional molecules to sustain its growth as well. Hypoxia stimulates adenosine monophosphate-activated protein kinase (AMPK), which in turn regulates the energy acquisition of tumor cells [9]. Additionally, hallmarks related to transcriptional regulation are also involved in the development of tumor tissues. E2F7, a member of the E2F family transcription factors (E2Fs), not only participates in cell cycle regulation as a transcriptional repressor [10, 11], but also contributes to tumor cell proliferation and metastasis. Besides, studies have found that E2F7 may be involved in mediating immune cell infiltration [12, 13]. Thus, transcriptional regulation affects not only tumor cells themselves but also tumor progression by modulating the cellular and molecular components in the TME [14]. In published GBM-related studies, the synergistic action of Transcript factors (TFs) is often observed within the same cell type [11, 15, 16]. However, the transcriptional regulatory mechanisms between TFs across different cell clusters remain unclear.

GBM diffusely infiltrates the brain, intermingling with non-neoplastic brain cells. This intricate TME forms the biological basis for treatment response and tumor recurrence. It is of utmost importance to delve deeply into the interactions between GBM cells and their immune microenvironment. However, the knowledge in this regard is currently insufficient. The enhanced resolution of single-cell sequencing can be utilized to uncover the key characteristics of highly heterogeneous tumors. Accordingly, we integrated transcriptome data from both bulk and single-cell levels. We screen for biomarkers and identify risk genes in GBM by employing machine learning techniques like stepwise Cox regression (StepCox) and random forests (RSF). Meanwhile, we utilize multi-omics analysis to identify TF–gene activity regulation networks and explore the transcriptional regulatory mechanisms between tumor cells and T cells.

## Methods

### Data collection

The multi-dimensional transcriptomic datasets used in this study, including single-cell, bulk, and spatial data, are summarized in Supplementary Table S1 (detailed accession numbers and repository identifiers are provided in the “Data availability” section). For the single-cell RNA-seq analysis, we utilized 15 primary samples derived from 9 patients with wild-type GBM to characterize the TME. For the bulk transcriptome analysis, a total of 769 primary GBM samples with survival information were collected and divided into training and validation cohorts. The training cohort consisted of 428 primary GBM samples obtained from The Cancer Genome Atlas (TCGA) and the Chinese Glioma Genome Atlas (CGGA) databases. For independent validation of prognostic markers, an additional 341 microarray-based disease samples were acquired from four independent Gene Expression Omnibus (GEO) cohorts. Additionally, 105 normal brain samples from the genotype-tissue expression (GTEx) database were utilized as non-tumor references for subsequent analysis. Finally, for the ST profiling, 12 primary wild-type GBM samples were used to analyze the spatial cellular architecture.

### Data preprocessing

For single-cell level data, cells expressing <200 genes or >2,500 genes were filtered out, and cells with mitochondrial gene content exceeding 20% were removed to ensure the quality of cells used in downstream analysis. After quality control filtering, 71,836 cells remained. A gene was retained if it was expressed in at least three cells. Following the removal of unqualified genes, 29,622 genes were retained for analysis. For bulk level data, data from the TCGA and CGGA platforms were converted into transcripts per million (TPM). The Combat algorithm (sva R package, version 3.35.2) was used to remove batch effects from the bulk RNA-seq expression profiles of the TCGA and CGGA platforms. For cases where multiple ENSEMBL IDs map to the same gene, we calculate the average expression level of each gene using “rowMeans” and select the gene with the highest expression level. The log_2_(*x* + 1) transformation was performed to generate clean microarray data. For spatial transcriptomics (ST) data, we loaded raw 10x Visium data, including gene expression matrices, spatial coordinates, and tissue images, using the “Load10X_Spatial” function, followed by data normalization and variance stabilization with “SCTransform” while retaining all genes for downstream analysis.

### Dimensionality reduction and clustering of cells

The preprocessed gene expression matrix and cell annotation information were processed using the Seurat R package (version 5.1.0). The top 2,000 highly variable genes, identified using the standard deviation (SD) algorithm, were used for principal component analysis (PCA). Expression profiles were normalized using the LogNormalize method (feature counts per cell divided by the total counts for that cell, multiplied by a scale factor). To maximize the explanation of data variability with the fewest principal components, the top 16 principal components were manually selected for cell clustering analysis using the UMAP algorithm. The Harmony algorithm [17] (version 1.2.1) was implemented to mitigate batch effects among tumor samples during data integration. To ensure the stability of the clustering results, we evaluated various resolution parameters using the clustree package (version 0.5.1). A resolution of 0.2 was applied to identify major cell lineages. For the subclustering of specific populations, we utilized resolutions of 0.9 for T cells and 0.8 for myeloid cells to resolve fine-grained cell subtypes. Cell clusters were defined using marker genes specific to cell types in GBM tissues, collected from published literature and the CellMarker2.0 database [18]. The complete set of markers used for cell type annotation, along with their corresponding clustering resolutions, is systematically documented (Supplementary Table S2).

### Identification of malignant tumor cells

The inferCNV algorithm [19] (version 1.10.1) was used to infer copy number states by calculating the ratio of gene expression levels in tumor cells to the average expression levels in reference normal cell populations. This methodology is particularly robust for GBM as it was fundamentally established and validated through foundational single-cell research in brain tumors [20–22]. In the single-cell transcriptome data, we used T cells and B cells as the reference set to calculate the CNV score for all cells. Oligodendrocytes and glia/neuronal cells were used as sources of malignant cells in the observation set to identify highly malignant cells. We defined the two cell types with CNV scores exceeding the mean as highly malignant cells. All cell clusters except myeloid cells were used to assess chromosomal copy number variations, including amplifications and deletions. The minimum average read count threshold for each gene in the reference cells of 10X Genomics data is set to 0.1.

### Immune phenotype of tumors based on transcriptomic profiling

We selected the top 50 DEGs from immune cell subsets in single-cell transcriptome data to construct an immune cell gene set. The single-sample gene set enrichment analysis (ssGSEA) algorithm was used to calculate the immune gene set enrichment score (GSES) for each cell to estimate the infiltration degree of different immune cell types in tumor samples. Tumor samples were clustered using ConsensusClusterPlus (version 1.68.0) based on GSES. The tumor samples were divided with *K* = 2, and the tumor samples with significantly enriched immune cells were defined as immune “hot” tumors. The GSES was normalized by the “scale” function, and half-violin plots and heatmaps were generated to visualize the enrichment results before and after scaling. Immune scores were calculated using “Estimate” (version 1.0.13) to validate the ssGSEA results.

### Gene set enrichment analysis

We used the fgsea (version 1.13.0) R package to test the enrichment of hallmark gene sets downloaded from MsigDB (msigdbr R package version 7.5.1). For the single-cell level, the Wilcoxon rank sum test (presto R package version 1.0.0) was used to calculate the DEGs, and the area under the curve (AUC) value was used as the ranking index to generate the pre-sorted list of genes. For bulk-level input, a pre-ranked gene list generated from differential expression analysis using the limma R package (version 3.60.2) was applied. Genes with adjusted *P*-value < 0.05 and |log2FC| > 1 were set as significant DEGs. The GSEA was used for 1,000 permutation tests, and NES > 0 and adjusted *P* value < 0.05 were defined as significant up-regulation of hallmark.

### Machine learning model construction and validation

To construct a model for predicting the prognosis of GBM patients, we first divided all bulk tumor samples into training and test sets at a ratio of 7:3. Then, we performed univariate Cox regression and Kaplan–Meier (KM) survival analysis on the 354 GBM progression-related feature genes identified across multiple dimensions. Only genes that showed statistically significant prognostic relevance (*P* < 0.05) in both tests were retained, resulting in 42 high-value candidate genes. These pre-screened genes were input into an integrated machine learning framework built using the R package mime1 [23].

We constructed and evaluated the model on both the training and test sets using 10-fold cross-validation, selecting the combined model with the highest C-index as the optimal model. Based on this optimal model, we further performed feature screening, ultimately identifying seven prognostic hallmark characteristic genes (HMsig). To comprehensively evaluate the model, we performed a meta-analysis integrating the training and test sets, and conducted ROC analyses on 1-year, 3-year, and 5-year survival predictions for GBM patients. Model performance was compared with existing methods using C-index and ROC values. Furthermore, we collected three external validation sets from the GEO database to validate the model’s performance in predicting patient outcomes (Supplementary Table S1).

### HMsig importance assessment

We employed the SHAP algorithm (fastshap R package version 0.1.1) to quantitatively assess the prognostic influence of HMsig genes. A survival analysis model was constructed through a combined machine learning algorithm model of forward StepCox and RSF. Then, the “explain” function was used to calculate the SHAP value for HMsig genes of each sample to measure their impact on the survival prediction results of patients. Visualize the importance of features through the “sv_importance” function.

### Cell communication analysis

Cell communication analysis was performed by CellChat [24] (version 1.6.1). We employed the established CellChat method to investigate interactions between tumor cells and immune cells. CellChatDB integrates signaling interaction information from the KEGG pathway database and literature from experimental studies. The human ligand–receptor (LR) database from CellChatDB was used as a reference to evaluate cell communication networks between two cell types. DEGs across all cell clusters were identified using the Wilcoxon rank-sum test (*P* < 0.05). To account for noise effects, the triMean quartile method was used to calculate the average expression of LR pairs in cell clusters.

### Construction of HMsig–LR interaction network

The Search Tool for the Retrieval of Interacting Genes/Proteins (STRING) database [25] enables the exploration of potential interactions between genes, constructing interaction networks that illustrate relationships such as physical contacts or regulatory targeting among HMsig and LR pairs. The genes derived from HMsig and LR pairs identified using CellChat were submitted to the STRING database to identify potential gene interaction networks. Subsequently, the gene interaction network was constructed and visualized using Cytoscape [26] (version 3.9.1).

### T cell developmental trajectory

Based on previous studies defining various T cell characteristics, we analyzed T cells and identified four T cell subtypes, which are: naive T cells, Tregs (regulatory T cells), CD8_T_EM (CD8+ T effector memory) cells, and CD8_T_EX (CD8+ T exhausted) cells. Pseudotime trajectories for these T cell types were constructed using Monocle3 [27] (version 1.3.3), a semi-supervised pseudotime analysis algorithm approximating PAGA (partition-based graph abstraction). Dimensionality reduction was performed using the UMAP algorithm via the “reduceDimension” function. We used “get_earliest_principal_node” function to identify the earliest principal node from naive T cells, which is a helper function provided by monocle3. This node was then designated as the root node using the “order_cells” function to calculate pseudotime values for all cells and infer the differentiation trajectory. Temporal distribution density curves for the T cell subtypes were visualized using the ggridges R package (version 0.5.6).

### Gene ontology enrichment analysis

Gene ontology (GO) enrichment analysis was performed using clusterProfiler [28] (version 4.12.0) and org.Hs.eg.db (version 3.19.1). For the pseudotime developmental modules of T cells, GO enrichment analysis was performed based on significantly DEGs within each module. Regarding the transcriptional regulatory subnetworks of tumor cells and T cells, GO enrichment analysis was conducted on genes driven by cell type-specific TFs.

### Construction of transcriptional regulatory networks

We used SCENIC [29] package (version 1.3.1) to identify regulons in single-cell transcriptomes. Single-cell datasets from two tumor cell types, CD8_T_EM, and CD8_T_EX cells were used as input. The GENIE3 algorithm was employed to construct regulatory networks based on motifs and ranked binding sites from RcisTarget. Regulon activity was calculated using AUCell. The regulon specificity score (RSS) for TFs in each cell type was computed using the “calcRSS” function (zThreshold = 0.1, thr = 0.1), to identify cell type-specific TFs. A binary matrix heatmap was generated, and TF–gene regulatory networks were constructed using Cytoscape (version 3.9.1). TF pairs with *r* > 0.2 and *P* < 0.05 were considered significantly positively correlated, indicating synergistic interactions. The “viewMotifs” function was used to examine motifs corresponding to TF–HMsig/immune checkpoint gene (ICG) pairs, exploring the mechanisms of TF synergy.

### Survival analysis

Survival analysis was conducted using the Survival (version 3.6.4) and survminer (version 0.4.9) R packages. For survival analysis of HMsig validated in GEO data, the StepCox(forward)+RSF model was used to calculate risk scores for each patient. Patients were stratified into high- and low-risk groups based on the median risk score, and differences in clinical outcomes were assessed using the log-rank test. KM curves were used to visualize survival characteristics.

To investigate the impact of HMsig LR gene interactions on GBM patient survival, we specifically evaluated the prognostic value of OPHN1–EFNB1 interaction patterns. Accounting for gene–gene interaction effects, we calculated the product of both genes’ expression values, and the optimal cutoff threshold for stratifying high versus low expression groups was determined using the “surv_cutpoint” function. Differences in clinical outcomes were assessed using the log-rank test, and KM curves were generated to visualize survival characteristics.

For survival analysis of synergistic TF pairs in TCGA and CGGA, the mean expression sum of each TF pair was used as the threshold to stratify patients into high and low-expression groups. Differences in clinical outcomes were assessed using the log-rank test, and KM curves were generated to visualize survival characteristics.

### Cell type deconvolution

The cell-type compositions for each spot were determined using the conditional autoregressive deconvolution (CARD) algorithm [30] (version 2.2.0). The CARD object was constructed from the ST data along with the scRNA annotated data described above to reference single-cell RNA sequencing (scRNA-seq) profiles encompassing seven cell types. We applied “CARD.imputation” function to improve CARD-based expression, enabling high-resolution mapping of both cell-type localization and imputed gene activity. Optimized ST data were chosen to reveal cellular distributions and gene expression patterns.

### Statistical analysis

The results of the statistical analysis are presented using box plots and violin plots. The statistical significance of differences between groups was determined using the unpaired, two-tailed student *t*-test, with *P* < 0.05 considered statistically significant. The correlation analysis of TFs was performed using Spearman’s correlation coefficient. These statistical analyses were performed using R software 4.4.1.

## Result

### Result 1 identifying molecular hallmarks for malignant cells

**Figure 1:**
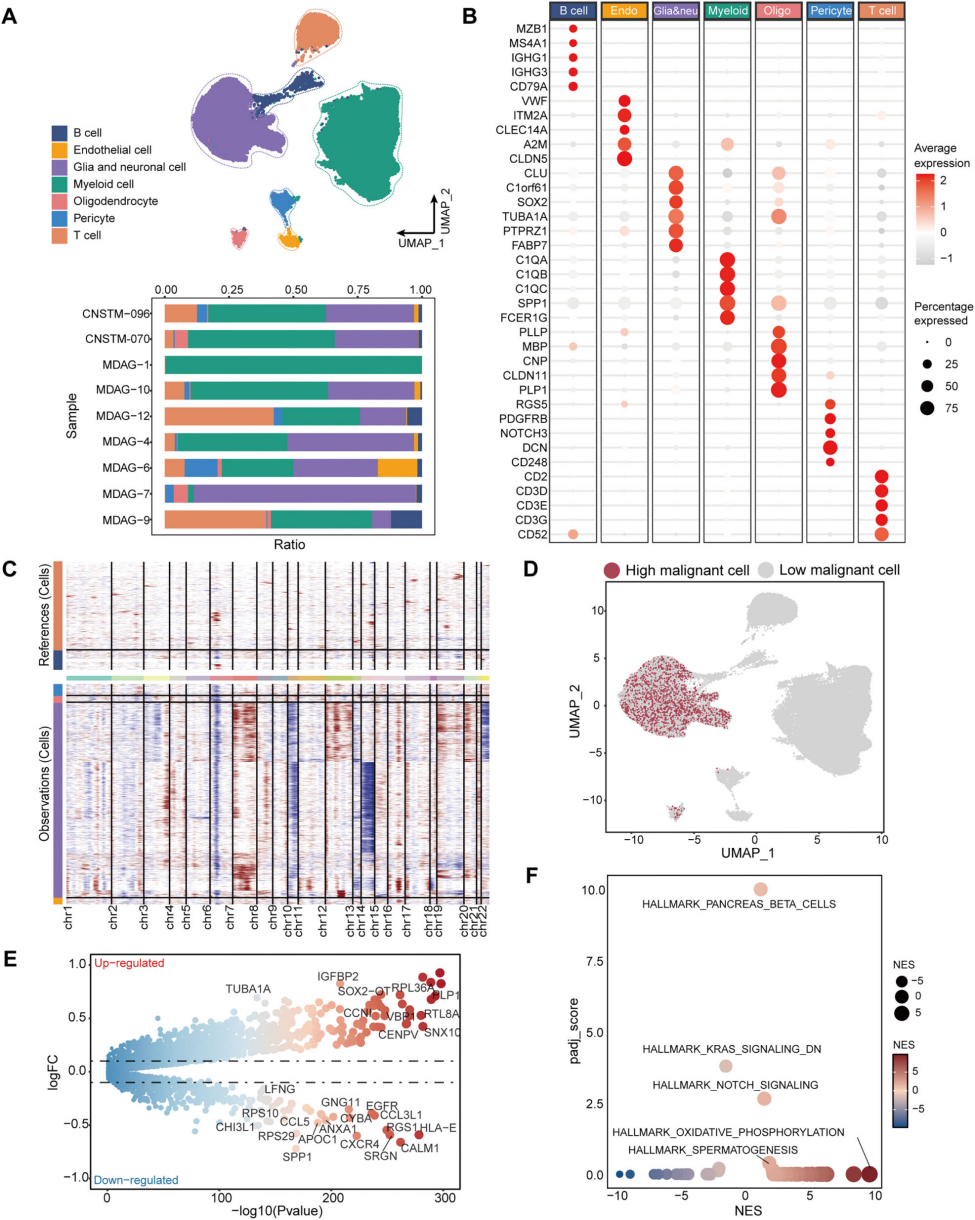
Single-cell transcriptome analysis of human glioma malignant cells. (A) UMAP projections of 71,836 aggregate single cells from 9 patients showing the composition of different cell types in human gliomas (upper panel). The stacked bar plot illustrates the proportion of each cell type across the nine patients (lower panel). The colors of the dots and bars represent different cell types. (B) Dotplot shows the expression of marker genes in different cell types in GBM. Dot size indicates the proportion of expressing cells, colored by average expression levels. (C) Heatmap shows the normal/malignant cell status determined by inferCNV analysis. It displays the relative expression intensity of each cell across various chromosomal regions, with colors representing CNV levels. The upper heatmap represents the results for the reference cells, while the lower heatmap represents the results for the observation cells. (D) UMAP projections are shown by malignant status. (E) Differential expression analysis of tumor malignant cells. The size and color gamut of the dots are determined by the −log10(padj) values. (F) GSEA analysis of tumor malignant cells. The size and color of the dots are determined by the normalized enrichment score (NES) values. The padj_score is derived by −log10(padj).

After rigorous quality control and normalization, the gene expression profiles of 71,836 cells from 9 GBM patients were used for in-depth analysis. We employed the Uniform Manifold Approximation and Projection (UMAP) clustering algorithm, with which all cells were annotated into seven distinct clusters (Fig. 1A; Supplementary Fig. S1A). These clusters included B cells, endothelial cells, glial and neuronal cells, myeloid cells, oligodendrocytes, pericytes, and T cells. Notably, the clusters of glial and neuronal cells and myeloid cells accounted for a much larger proportion of the total cell population than B cells and T cells in GBM patients. Moreover, in certain patients, such as MDAG-1 and MDAG-7, a relatively low level of immune cell infiltration was observed, which prominently emphasizes the momentous individual heterogeneity within GBM tissues. Canonical marker genes exhibited high expression specificity within their corresponding cell lineages, confirming the accuracy of cell type annotation (Fig. 1B; Supplementary Table S2).

To accurately distinguish the malignancy level of tumor cells, the inferCNV algorithm was utilized to compute the copy number variation based on scRNA-seq data (Fig. 1C). Since glial and neuronal cells are recognized as the main origin of malignant GBM cells, those two types of nerve cells with a CNV score exceeding the mean were defined as high malignant. Conversely, 4,829 cells were designated as low malignant according to their CNV scores. CNVs were also detected in pericytes and endothelial cells (Supplementary Fig. S1D). Subsequently, all high malignant and low malignant tumor cells were extracted and subjected to UMAP algorithm once again to visualize the distribution of tumor cells (Fig. 1D).

In order to delve deeper into the unique molecular hallmarks of malignant cells, a differential expression analysis was carried out between the high malignant and low malignant tumor cells. This analysis disclosed that several genes, such as IGFBP2 and VBP1, were significantly upregulated (Fig. 1E). IGFBP2 promotes glioma progression by activating the PI3K/AKT pathway and MMP2 [31], while VBP1 regulates hypoxia responses by degrading HIF-1α, a key transcriptional regulator [32]. GSEA results revealed that the markedly enriched hallmark pathways encompassed the KRAS signaling pathway, the NOTCH signaling pathway, OXPHOS, and spermatogenesis along with an additional 24 markedly up-regulated hallmarks (Fig. 1F). In GBM, normal cells mainly rely on aerobic oxidation of glucose to generate energy. However, tumor cells, in addition to enhanced glycolysis, also utilize the OXPHOS to produce more adenosine triphosphate (ATP), providing ample energy for their abnormal biological behaviors. Beyond the tumor-related metabolic pathways, numerous studies have demonstrated that the Notch signaling pathway, which is associated with cell proliferation, is overactive in GBM [33]. The diversity of these hallmarks implies the complex TME of GBM. Interestingly, the enrichment of the spermatogenesis pathway in GBM suggests that some genes involved in spermatogenesis may play a role beyond their traditional functions in reproductive tissues. Previous studies have indicated that abnormal expression of spermatogenesis-related genes is associated with glioma progression and malignancy [34]. In addition, research has shown that GBM may exploit immune privilege mechanisms by expressing reproductive antigens, which could recruit Tregs activated in the reproductive system [35]. Notably, the spermatogenesis gene Sohlh1 has been shown to inhibit GBM cell proliferation and migration by modulating the Wnt/β-catenin signaling pathway [36], further supporting the essential roles of spermatogenesis-related genes in GBM progression.

### Result 2 dissecting immune hot and cold tumors in GBM through single-cell and bulk transcriptomics

**Figure 2:**
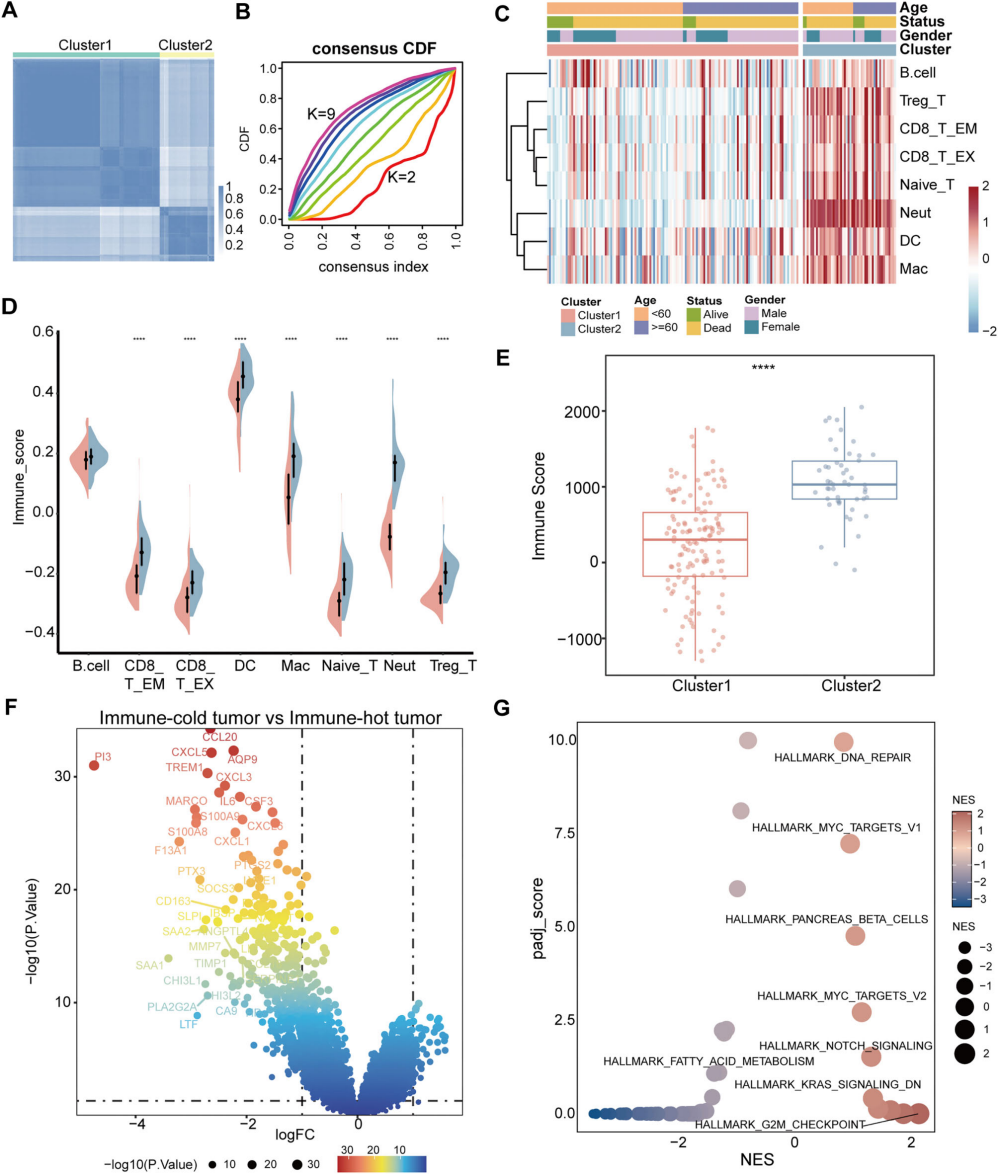
Depicting the immune “cold” and “hot” status of GBM. (A) The consensus score matrix of GBM samples when *k* = 2. (B) The CDF curves of the consensus matrix for each *k* (shown by colors). (C) Heatmap shows the infiltration abundance of eight immune cell types evaluated by ssGSEA for two clusters. The distribution of patient categories, gender, age, and survival status can be seen at the top of the heatmap. (D) The violin plot shows the distribution of eight immune cell types infiltrations between two clusters. The red color represents the cluster of immune “cold” tumor and blue represents the cluster of immune “hot” tumor. The significance of differences between the two clusters is indicated by “***”. (E) The boxplot shows the distribution of immune score inferred by ESTIMATE algorithm between two clusters. (F) Differential expression analysis of immune “cold” tumors. The size and color gamut of the dots are determined by the −log10(*P* value) values. We set |log_2_FC| ≥ 0.5 and *P* value ≤ 0.05 as the thresholds for screening DEGs. (G) GSEA analysis of immune “cold” tumors. The size and color of the dots are determined by the NES (normalized enrichment score) values. The padj_score is derived by −log10(padj).

In GBM, the microenvironment is typically characterized as “cold” due to limited immune infiltration [37]. The study found that the immune microenvironment significantly influences patient prognosis [38]. To systematically dissect these features and their clinical implications, we classified tumors into immune “cold” and “hot” states at the bulk level, integrating single-cell data to resolve limitations of single-modality analysis. We initially conducted subtype identification on T cells and myeloid cells (Supplementary Fig. S1B and C). T cells were categorized into naive T cells, regulatory T cells (Treg), CD8 T EM, and CD8 T EX, whereas myeloid cells were identified as macrophages, dendritic cells, and neutrophils (Supplementary Fig. S1E–H). Based on the results of ssGSEA of the top 50 differentially expressed genes in 8 immune cell clusters at the single-cell level, we conducted consensus clustering analysis. In that way, all GBM samples were divided into *k* (*k* = 2–9) clusters. The cumulative distribution function (CDF) curve of the consensus score matrix and the delta area plot indicated that the optimal number was obtained when *k* = 2 (Fig. 2A and B; Supplementary Fig. S1I). The two clusters (Clusters 1 and 2) exhibited clear differences in immune infiltration, in which B cells, Treg cells, CD8_T_EM cells, and other immune cells exhibit pronounced enrichment in the tumor samples of Cluster 2. Undoubtedly, the immune infiltration abundance in Cluster 2 was remarkably higher compared to Cluster 1 (Fig. 2C and D). Therefore, we defined Cluster 1 as an immune “cold” tumor and Cluster 2 as an immune “hot” tumor. We noticed neutrophils are significantly enriched in immune-hot tumors. Neutrophils exert anti-tumor effects by directly killing tumor cells through ROS, NO, and granular protein release [39–43]. This implies neutrophil enrichment helps differentiate immune “cold” from immune “hot” states.

We also explored the distribution of patient gender, age, and survival status within immune “cold” and “hot” tumor samples. It is undeniable that the number of immune “hot” tumor samples is markedly less than that of immune “cold” tumors. Among GBM patients, the proportion of patients in the survival state was significantly lower than that of deceased patients. Considering the overall GBM patient population, the prognosis of male patients is generally worse than that of female patients [44]. Nevertheless, upon closer examination of the immune “hot” tumor cluster, an intriguing phenomenon emerged. Among the living patients under 60 years old, all were male. This finding implies that within a specific GBM tumor immune subtype and age range, males may possess some undiscovered survival advantages, which deserve further research (Fig. 2C). When additionally observing the relationship between immune cell infiltration and the TME, we noticed the special behavior of B cells in immune “cold” tumors. Regarding the enrichment phenomenon of B cells in immune “cold” tumors, it may be associated with an immunosuppressive function [45, 46]. This observation suggests that B cell activity in GBM is context-dependent, potentially contributing to a pro-tumorigenic environment rather than an active anti-tumor response in specific TME. We employed the ESTIMATE algorithm to verify the analysis results, which corroborated the outcomes of our previous ssGSEA (Fig. 2E).

We performed differential expression analysis on immune “cold” tumors and immune “hot” tumors in order to deepen our understanding of the molecular characteristics of GBM hallmarks (Fig. 2F). CCL20 and CXCL5, as chemokines, could attract immune cells (such as T cells and monocytes) into the TME. Their downregulation implies a weakened ability to recruit immune cells, leading to insufficient infiltration of immune cells in tumor tissues and thus giving rise to the “cold” tumor phenotype. Simultaneously, the GSEA result revealed six significantly upregulated pathways, including DNA repair, Myc-targets-v1, KRAS signaling pathway, etc. (Fig. 2G). The IL2_STAT5, IL6_JAK_STAT3, and inflammatory response hallmarks are remarkably downregulated in immune “cold” tumors (Fig. 2G). In the TME, the JAK-STAT signaling pathway is essential for maintaining the homeostasis of immune cells. STAT5 and STAT3, as dominant TFs in this pathway, can influence genes related to cell proliferation, apoptosis, and immune responses. STAT5 acts as a catalyst for promoting the proliferation of T cells; STAT3 is indispensable for maintaining the cell identity of macrophages [47]. The downregulation of their related pathways leads to the decline of immune killing functions and spurs the generation of an immunosuppressive microenvironment. There were five common hallmarks across both data modalities, namely the E2F transcription factor family, G2/M checkpoint, spindle mitosis, Wnt signaling pathway, and spermatogenesis. A recent pan-cancer study revealed that the androgen receptor (AR) is overexpressed in GBM [48]. *In vitro* experiments using anti-androgen drugs resulted in the inhibition of GBM cell proliferation [49, 50]. Spermatogenesis, a process regulated by androgens and highly active in the male reproductive system, showed significant enrichment in both datasets, suggesting that male patients may have unique susceptibilities or therapeutic responses in certain molecular mechanisms. Research has demonstrated that in high-grade gliomas, there is an enrichment of MYC_TARGETS_V1, G2M checkpoint, and E2F target hallmarks [51]. It is worth mentioning that these five pathways are significant in transcriptional regulation and are also involved in cell cycle regulation. Cancer cells utilize cell cycle checkpoints to delay mitosis and repair DNA damage. In particular, the G2/M phase of the cell cycle carried great weight in DNA repair [52]. The E2F family directly regulates the transcription of genes involved in DNA replication and cell cycle progression [53], further highlighting its transcriptional regulatory functions.

### Result 3 establishment and validation of hallmark-related prognostic signatures in GBM

**Figure 3:**
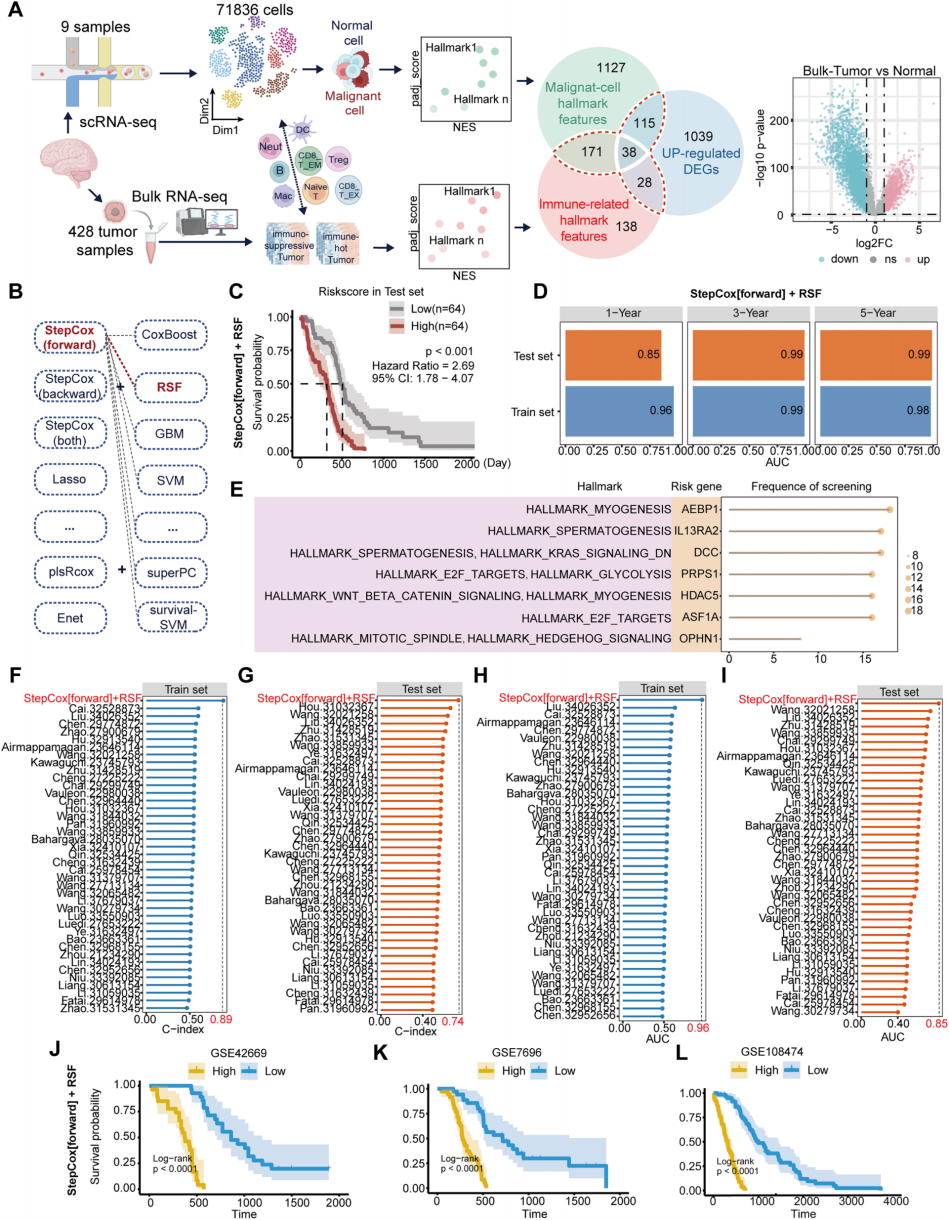
Identifcation of potential GBM features in GBM. (A) An overview of features selection workflow for patient tumors. The upper part shows the feature selection strategy at the single-cell level; the lower part shows the feature selection strategy at the bulk level; the volcano plot on the far right represents the differential analysis between tumor and normal samples at the bulk level. (B) Combined machine learning model framework of Mime algorithm. The combination highlighted in red represents our optimal model. (C) Kaplan–Meier curves of OS according to the StepCox(forward) + RSF in test set. (D) Time-dependent AUC performance of the optimal model for predicting OS at 1, 3, and 5 years. The specific AUC values are labeled in the figure. (E) Selection frequency of HMsig in various machine learning algorithms and their enriched hallmarks. The size of the dots corresponds to the frequency at which the features are selected. (F–I) C-index and ROC analysis comparing our optimized model with published GBM signatures in the TCGA–CGGA cohort. The dashed line corresponding to the red scale represents the C-index/AUC value of our optimized model. (J–L) Kaplan–Meier curves of OS based on the StepCox(forward) + RSF model in an additional GEO validation set.

To establish a GBM risk prediction model, we used a random combination of 11 machine learning algorithms [23] and integrated the previously analyzed hallmarks based on bulk-level GBM transcriptional data with survival information. We used a multi-dimensional intersection strategy to identify hallmark-related feature set for prognostic modeling. This approach integrates three key aspects: the intrinsic malignant characteristics of GBM tumor cells captured by single-cell RNA-seq hallmark genes, the immune cold environment features reflected by bulk tumor hallmark genes, and the overall transcriptomic expression universality across patient populations represented by bulk DEGs. By focusing on the overlap of these gene sets, we prioritized genes that are critical not only for the malignant progression of GBM cells but also for shaping the immunosuppressive environment (Fig. 3A). In total, 352 immune-related tumor hallmark genes at the combined single-cell and bulk levels were obtained. These features were first filtered using univariate Cox regression and KM survival analysis, which identified 42 high-value prognostic candidate genes. Subsequently, these 42 genes were input into our integrated machine learning framework to identify the most essential predictors. The combination of StepCox (forward) and RSF was identified as the optimal model, as it achieved the highest C-index in the validation datasets (Fig. 3B; Supplementary Fig. S2A). Among the evaluated algorithms, the StepCox (forward) model was utilized to achieve an optimal balance between predictive power and model parsimony by effectively eliminating redundant variables, ultimately pinpointing seven core hallmark-related prognostic signatures (HMsig): AEBP1, ASF1A, PRPS1, DCC, OPHN1, IL13RA2, and HDAC5. Survival analysis indicated that the HMsig could effectively distinguish patients with different risk levels, with AUC values for 1-, 3-, and 5-year survival exceeding 0.85 in both sets (Fig. 3C and D; Supplementary Fig. S2B).

Meta-analysis further suggested that the risk score calculated by the StepCox (forward) and RSF model was a risk factor for GBM (Supplementary Fig. S2E and F). We then tallied the frequency with which these seven genes were selected as core features across different algorithms (Fig. 3E, Supplementary Fig. S2C). By comparing our obtained optimal model with existing GBM risk models constructed from different literature and calculating the C-index and AUC values, our optimized model ranked first (Fig. 3F–I), demonstrating superior predictive stability.

Concerning the biological basis of HMsig, recent studies have found that ASF1A was identified as a biomarker for malignant diseases such as lung adenocarcinoma and hepatocellular carcinoma [54–56]. However, no studies have yet reported that ASF1A and OPHN1 can be a risk gene for GBM. ASF1A regulates the expression of cell cycle-related genes and influences glial cell differentiation [57]. OPHN1 not only participates in cell cycle regulation [58, 59] but has also been found to promote tumor progression when overexpressed [60]. To further elucidate the impact of these seven genes on patient survival prediction, we employed the SHAP algorithm to calculate SHAP values based on risk scores derived from the StepCox (forward) and RSF combined model (Supplementary Fig. S2D). SHAP analysis revealed that the AEBP1, ASF1A, and PRPS1 genes had the highest SHAP values, indicating their significant impact on survival prediction. These SHAP values suggest that these three genes are major predictors in HMsig, and their direction is consistently positively correlated with patient risk. This confirms that high expression of these HMsig genes is a key factor leading to elevated risk scores and indicates their role as high-risk determinants in the GBM microenvironment. Additionally, several independent validation datasets from GEO datasets confirmed that HMsig effectively distinguished high-risk from low-risk patients, demonstrating robust validation and strong generalizability (Fig. 3J–L).

### Result 4 revealing immune regulatory mechanisms in TME through cell communication analysis

**Figure 4:**
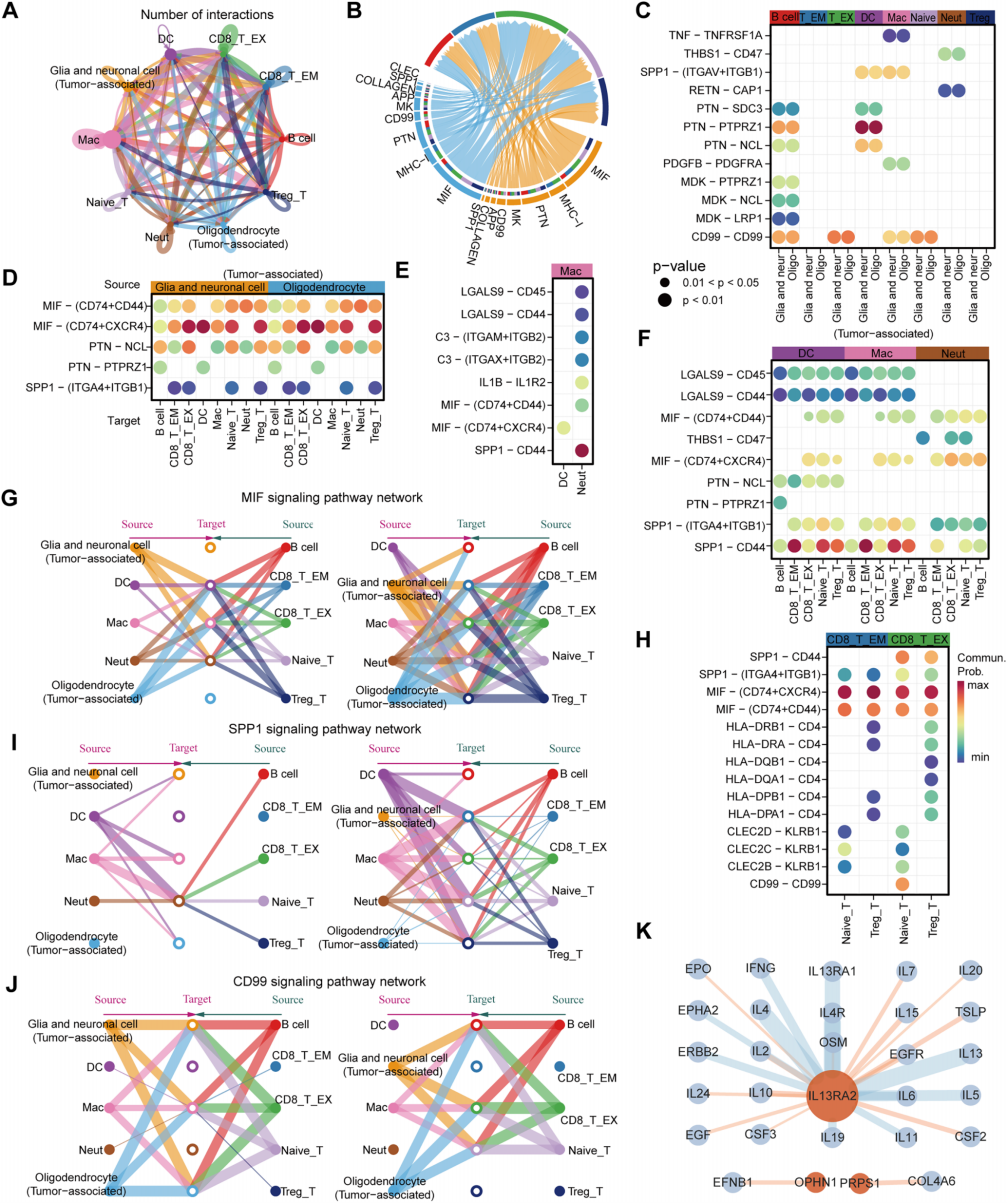
CellChat analysis of the communications between tumor cells and immune cells. Global communications are presented by circle plots showing the number of significant LR pairs in 10 cell clusters. (B) Chord diagram shows the communication strength at the gene signaling pathway level. (C–F, and H) Significant LR pairs between tumor and immune cells, within myeloid cells and T cells, and between myeloid and other immune cells. Dot color reflects communication probabilities, and dot size represents computed *P*-values (one-sided permutation test). Empty space indicates zero communication probability. (G, I–J) Three representative signaling pathways, MIF, SPP1, and CD99 pathways, were further analyzed. The inferred networks of communication between all cell types are displayed using hierarchical plots. (K) PPI networks between HMsig genes and LR pairs. Orange dots represent HMsig genes, blue dots represent LR pairs, and the color and thickness of the lines are determined by the combined score. The combined score is the standardized confidence metric from the STRING database.

The TME is not merely an aggregation of cancer cells but rather a complex ecosystem composed of multiple cell types. These cells interact with one another via the secretion of factors, cytokines, and cell-to-cell contacts, jointly affecting the growth, invasion, and metastasis of tumors. For the purpose of deeply profiling the TME of GBM, the CellChat algorithm was applied to examine the communication between tumor cells and T cells, B cells, and myeloid cells. CD8_T_EM and CD8_T_EX cells engaged in intense interactions with tumor cells, myeloid cells, and other T cells (Fig. 4A). Apparently, the two types of tumor cells have the strongest interaction with immune cells in macrophage migration inhibitory factor (MIF) signaling pathway, followed by pleiotrophin (PTN) signaling pathway. CD8_T_EX cells account for a relatively large proportion in the communication intensity with the two types of tumor cells in the MIF, major histocompatibility complex class I (MHC-I), and PTN signaling pathways (Fig. 4B).

Thereafter, we conducted a more detailed examination of the expression of LR pairs. MIF−(CD74+CXCR4)-mediated firm interactions between tumor cells and T cells (Fig. 4C and D). In GBM, this interaction may lead to the suppression of immune cell activity. Tumor cells can inhibit macrophage activity through the MIF−(CD74−CXCR4) [61]. The interaction of CD99–CD99 was also robust between immune cells and tumor cells (Fig. 4C). The engagement of CD99 and its ligand can upregulate the expression of IL-6 and TNF-α, which in turn promotes tumor cell proliferation and survival, ultimately correlating with poor clinical outcomes [62, 63]. The interaction mediated by SPP1–CD44 between myeloid cells and other immune cells was remarkably strong (Fig. 4E and F). SPP1–CD44 inhibits T cell infiltration, reducing the number of T cells in tumor tissues and exacerbating tumor immune evasion [64]. Other LR pairs, such as KLRB1–CLEC2D, although expressed at relatively low levels in certain cell types, are also essential to the TME. The interaction between the immune checkpoint molecule KLRB1 and CLEC2D is of great significance for tumor progression and immune evasion [65]. Previous studies have demonstrated that the interaction of KLRB1–CLEC2D can mediate immunosuppression and potentiate the development of GBM [66].

These findings implied that MIF, SPP1, and CD99 are indispensable for the TME. Therefore, we constructed hierarchical clustering maps for these three genes to illustrate the communication intensity between cell clusters and identify the key cell clusters involved in cellular communication. In line with our previous LR analysis, tumor cells and immune cells exhibited strong communication via the MIF signaling pathway, which was distinct from the SPP1 and CD99 signaling pathways (Fig. 4G and J). This may be attributed to the expression of MIF receptors on various immune cells, such as CD74 on the surface of macrophages and dendritic cells. Additionally, the binding of MIF to its receptors activates multiple downstream signaling pathways, including ERK1/2, AMPK, and AKT, following MIF-receptor binding, thereby strengthening their interactions [67]. Within the SPP1 signaling pathway, the communication between macrophages, dendritic cells, and T cells was particularly intense (Fig. 4I). SPP1 might enhance the interaction between myeloid cells and T cells, thereby suppressing immune surveillance. Substantial intercellular signaling communication occurred between tumor cells and immune cells in the MIF, SPP1, and CD99 signaling pathways. Among tumor cells and T cells, the direct or indirect interaction became the main factor contributing to the immunosuppressive microenvironment in GBM [61, 68].

Since we did not clearly discern the interaction between the HMsig and LR pairs, we then constructed a protein–protein interaction (PPI) network to explore the associations between them (Fig. 4K). The network analysis identified a direct interaction between EFNB1 and OPHN1. In GBM, the high expression of EFNB1 was related to poor prognosis for patients and could potentially serve as a prognostic marker and therapeutic target [69]. Moreover, elevated expression of both EFNB1 and OPHN1, which interact within the PPI network, was associated with worse patient outcomes (Supplementary Fig. S3A).

### Result 5 elevated expression of ICGs during T cell differentiation

**Figure 5:**
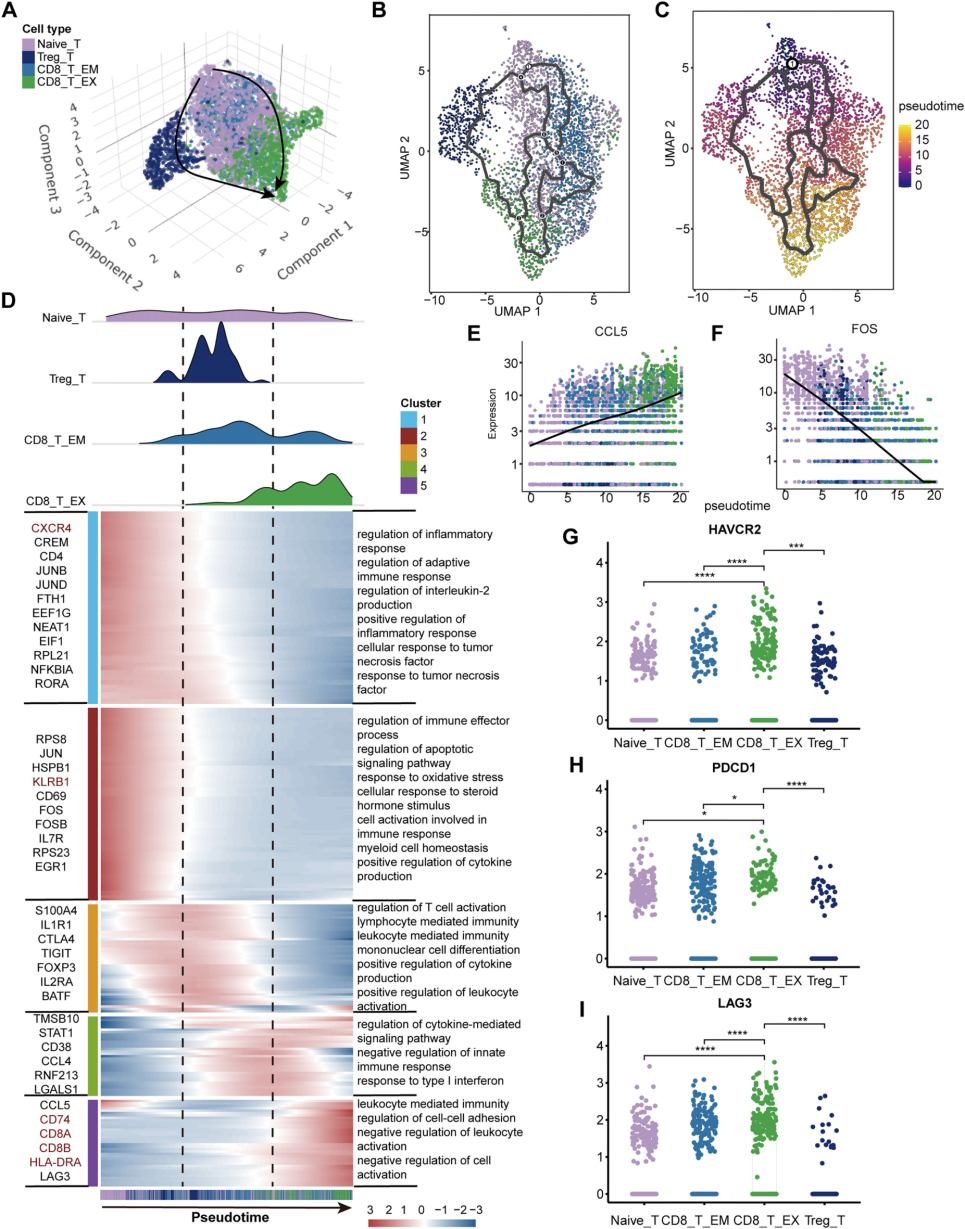
scRNA-seq profiles of dynamic changes in T cells. (A) Three-dimensional pseudotime analysis exploring the cellular trajectory of T cells based on highly variable genes. The black lines with arrows correspond to the principal graph learned by Monocle3, depicting the global developmental flow in 3D space. Individual dots represent single cells color-coded by their respective T cell subtypes. (B) Two-dimensional planar projection of the T cell developmental trajectory. (C) Pseudotime ordering of T cells initialized at the Naive_T cell cluster. The color gradient, ranging from purple (root) to yellow (terminal), represents the continuous progression of cells along the inferred developmental timeline. (D) The cell distribution of each T cell cluster along with the pseudotime (upper panel), color-coded by T cell clusters. Heatmap showing dynamic expression changes of genes in T cells (lower panel). The GO enrichment terms for each cluster are displayed on the right of the heatmap. (E and F) Gene expression dynamics along the trajectory, including CCL5 (E) and FOS (F). The color of dots coded according to their cell types, and the line represents the gene expression at each time point. (G–I) Boxplot showed the expression of immune checkpoint molecules (HAVCR2, PDCD1, LAG3) in T cells. The color of dots coded according to their cell types. The significance of differences between each pair of cell clusters is marked by “*,” with “ns” indicating no significant difference.

T cells show diverse differentiation trajectories as a result of the marked impact of the TME [70]. We established a 3D developmental trajectory for T cells. From this analysis, two common trajectories emerged. One trajectory commenced with naive T cells, passed through Treg cells, and ended at CD8_T_EX cells; the other started from naive T cells, proceeded through CD8_T_EM cells, and terminated at CD8_T_EX cells (Fig. 5A; Supplementary Fig. S3B). This bifurcation suggests that the exhaustion of T cells in GBM is a convergent process that can be achieved either through an immunosuppressive pathway or through the gradual decline of effector memory cell function. Meanwhile, we mapped the 2D trajectory for a more detailed and lucid depiction (Fig. 5B and C). We used the R package clusterProfiler to conduct GO functional enrichment analysis on genes related to T-lineage differentiation. This allowed us to explore the impact of these genes on cellular physiological functions (Fig. 5D; Supplementary Table S3). Genes associated with the initial T cell differentiation stage, including CD4, CXCR4, CD69, and KLRB1, display high expression levels in Clusters 1 and 2. The up-regulation of CXCR4 may be related to the enhanced migration ability of T cells and contribute to the aggregation of T cells [71]. Serving as T cell activation markers, CD69 and KLRB1 are markedly upregulated in activated CD4+ T cells [72, 73]. GO enrichment analysis reveals that Clusters 1 and 2 are enriched in functional pathways like positive regulation of the inflammatory response and cytokine production. Notably, genes expressed by Tregs cells, such as TIGIT, FOXP3, and IL2RA, show high expression in Cluster 3. Cells clustered into Cluster 3 possess functions such as regulation of T cell activation and positive regulation of leukocyte activation. These cells are crucial in immune response and cytotoxic killing. At the terminal stage of T cell differentiation, Cluster 5, characterized by high expression of CCL5 and LAG3, exhibited enrichment of functional pathways related to T cell exhaustion, including intercellular adhesion regulation and inhibiting immune cell activation.

At the same time, we monitored the expression changes of key genes that drive cellular development or state transitions during the pseudotime. CCL5 is a pivotal chemokine determining whether tumors will be infiltrated by T cells. As the T cell pseudotime trajectory progresses, the expression of CCL5 gradually increases (Fig. 5E). While this is beneficial for recruiting T cells to infiltrate the tumor, it also serves as a marker of the malignant transformation process in GBM [74]. FOS, functioning as a TF, is involved in regulating various pathophysiological processes of cells. During T cell activation, the product of the FOS gene participated in modulating the expression of cytokine genes [75]. With the development of the T cell, the expression of FOS gradually decreased, which can affect the recruitment and activation state of T cells (Fig. 5F). Besides, the analysis showed that the genes in Cluster 3 were highly expressed in the early stage of the trajectory and then gradually declined. This suggests that these genes might play an important role in the early stage but were rapidly suppressed or shut down, like FOXP3 and IL2RA expressed by Treg cells (Supplementary Fig. S3C). However, Cluster 5 presented an opposite pattern (Fig. 5D). For instance, the immune checkpoint molecules LAG3 was highly expressed at the terminal stage, corresponding to the temporal distribution of CD8_T_EX. This displays that the high expression of immune checkpoint molecules would lead to the exhaustion state of T cells. Unexpectedly, we found that the LR pairs on the T cell surface were also key genes driving T-lineage evolution. Take HLA-DRA-CD4, for example, it promotes T cell activation and differentiation [76].

The co-expression of multiple co-inhibitory receptors is a crucial marker of T cell dysfunction. Building upon the previous analysis, the results demonstrated that LAG3 was highly expressed at the end stage of T cell differentiation. Thus, we investigated the gene expression of all immunosuppressive checkpoint molecules. We found that PD1 (PDCD1), LAG3 (LAG3), TIM3 (HAVCR2), and two other genes were highly expressed during the T-progression. The high expression of these three ICGs in CD8_T_EX cells is associated with the functional decline of CD8_T_EM cells, potentially facilitating their conversion into CD8_T_EX cells (Fig. 5G–I; Supplementary Fig. S3D and E).

### Result 6 disclosing synergistic TFs regulation in tumor cells and T cells for prognosis

**Figure 6:**
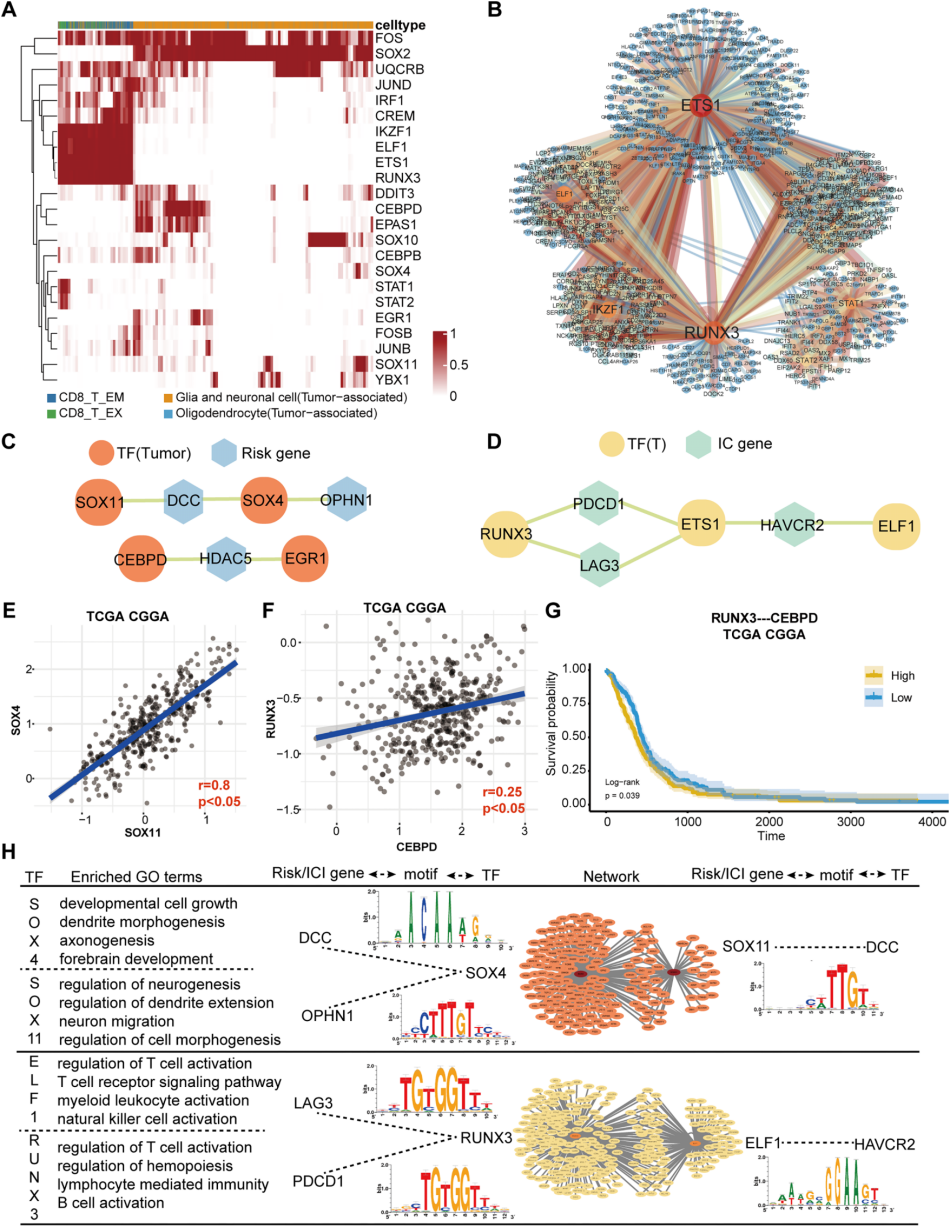
Regulatory roles of TFs between tumor cells and T cells. (A) Heatmap shows the binary activity of cell-type-specific TFs identified via regulon specificity scores in tumor and CD8+ T cells. (B) Transcription regulatory network constructed by CD8_T_EX-specific TFs and its target genes. Warm-toned dots represent TFs, and cold-toned dots represent target genes. The color of the lines represents the number of motifs shared between the TFs and their target genes. (C and D) Schematic diagram of TF–HMsig/ICG regulatory mechanism. Orange circles represent TFs driving HMsig in tumor cells, yellow circles represent TFs driving immune checkpoint molecules in T cells, and blue and green hexagons represent the identified HMsig and ICGs. (E and F) Correlation analysis of TFs in TCGA–CGGA cohort, including SOX4–SOX11 (E) and RUNX3–CEBPD (F). The correlation coefficient and significance markers are indicated in the lower right corner of the figure. (G) Survival analysis of RUNX3–CEBPD in TCGA–CGGA cohort. The yellow and blue line, respectively, indicate the high and low gene expression of RUNX3–CEBPD in TCGA–CGGA cohort. (H) TF co-regulation mechanisms in two cell types. On the left are the GO terms enriched by the TF regulatory subnetworks, while on the right are the TF regulatory subnetworks with synergistic interactions, along with the motifs corresponding to the HMsig/ICG regulated by these TFs. The synergistic mechanisms in tumor cells are shown in the upper section, with the regulatory network highlighted in orange. The synergistic mechanisms in T cells are also displayed in the upper section, with the regulatory network highlighted in yellow.

TFs, as upstream regulatory elements, can directly influence the behaviors of tumor cells and the activities of immune cells. To identify transcription factors that drive the expression of HMsig and immune checkpoint molecules, we performed SCENIC analysis on two tumor cell populations and two CD8 T cell subsets exhibiting strong cellular interactions. In addition, we focused specifically on CD8_T_EM and CD8_T_EX cells because they represent a key transition point in the T cell trajectory, where effector memory T cells with tumor-killing functions shift into exhausted T cells. This transition is highly relevant to the immune escape mechanisms in GBM. The cell-specific TFs, ETS1 and RUNX3, were in an active state in CD8_T_EM and CD8_T_EX cells; SOX2 and EPAS1 were in an active state in tumor cells (Fig. 6A; Supplementary Fig. S4A). Among them, ETS1 was involved in angiogenesis, which enhances the invasiveness and metastatic potential of tumor cells [77]. SOX2, on the other hand, promotes tumor cell proliferation by regulating genes associated with cell cycle progression [78]. All these suggest that TFs also participated in the regulation of the TME. Afterward, we constructed gene regulatory networks (GRNs) for CD8_T_EX, CD8_T_EM, and tumor cells to map specific TFs–gene interactions (Fig. 6B; Supplementary Fig. S4L and M). The regulatory network centered around TFs such as ETS1 and RUNX3 is essential for the occurrence, development, and functional activation of multiple immune cells [79–82]. In light of the HMsig we uncovered, we probed for their corresponding driving TFs and obtained four of them, namely SOX11, SOX4, CEBPD, and EGR1. We then created a schematic diagram of the TF–HMsig/ICG regulatory mechanism (Fig. 6C and D).

Understanding the cooperative interaction among TFs and its impact on GBM is crucial as it significantly affects GBM prognosis. Hence, we scrutinized the correlations among TFs at the bulk transcriptome level and found that the TFs were concordant in expression and significantly associated (Fig. 6E; Supplementary Fig. S4B). Additionally, the expressions of the TFs in two cell types at the single-cell level were consistent as well (Supplementary Fig. S4C–K). We discovered that RUNX3 and CEBPD showed similar expression patterns. These two TFs also significantly differentiate patients in the high-risk and low-risk groups within the TCGA–CGGA datasets. This shows that the synergistic action of TFs with different regulatory modes can influence patient prognosis (Fig. 6F and G).

When cells are confronted with various environmental stresses, such as oxidative stress and nutrient deficiency, the cooperative action of TFs can regulate the gene expression within the cells, assisting the cells in adapting to environmental changes and maintaining cell survival and function [83]. During the process of cell differentiation, the cooperative action of TFs is also a key determinant of cell fate [84]. With the aim to explore the regulatory mechanisms of TFs with cooperative effects among cells, we performed functional enrichment analysis on the GRNs and searched for the motifs of TFs. In tumor cells, the subnetworks regulated by SOX4 and SOX11 were enriched in pathways related to developmental cell growth, neuron migration, and dendrite morphogenesis, among others. Meanwhile, in T cells, the subnetworks regulated by ELF1 and RUNX3 were enriched in functional pathways associated with regulation of T cell activation, lymphocyte-mediated immunity, and lymphocyte proliferation. Although the specific regulatory mechanisms of TFs diverge, TFs in tumor cells and T cells play vital roles in different cellular functions. TFs in tumor cells impact cell development, and those in T cells coordinate the immune response. These findings show a functional similarity between the two clusters of TFs. They are both involved in key cellular processes essential for the functions and survival of their respective cell types (Fig. 6H).

Subsequently, we identified the motifs of TFs associated with HMsig and ICGs, as their expression levels hinge predominantly on specific motifs in their regulatory regions and the binding of corresponding TFs. We found that RUNX3 might simultaneously regulate the expressions of two immune checkpoint molecules, LAG3 and PDCD1. Hence, RUNX3 may coordinate the expression of two immune checkpoint molecules by binding to the same motif, thereby establishing a “dual-brake” mechanism that more efficiently suppresses T cell activity. In the TME, this mechanism could be utilized by tumor cells to promote immune escape through upregulation of RUNX3 and simultaneous activation of PDCD1 and LAG3. Moreover, we discovered that the RUNX3 binding motif is highly conserved between humans and mice through extended motif analysis. Such conservation allows TFs to maintain stable regulation of gene expression and ensure proper biological function. RUNX3 has been shown to play a critical role in T cell differentiation and function, and its dysregulation is associated with impaired immune responses and cancer progression [85]. Additionally, the conservation of TF binding motifs, such as those of RUNX3, is essential for maintaining immune homeostasis and preventing pathological conditions like autoimmune diseases and cancer [86]. The concordance in the preferred binding motif sequences of these two genes is clearly of vital importance for precisely and thoroughly elucidating the immune escape mechanisms.

### Result7 ST profiling of molecular heterogeneity and immunosuppressive microenvironment in GBM

**Figure 7:**
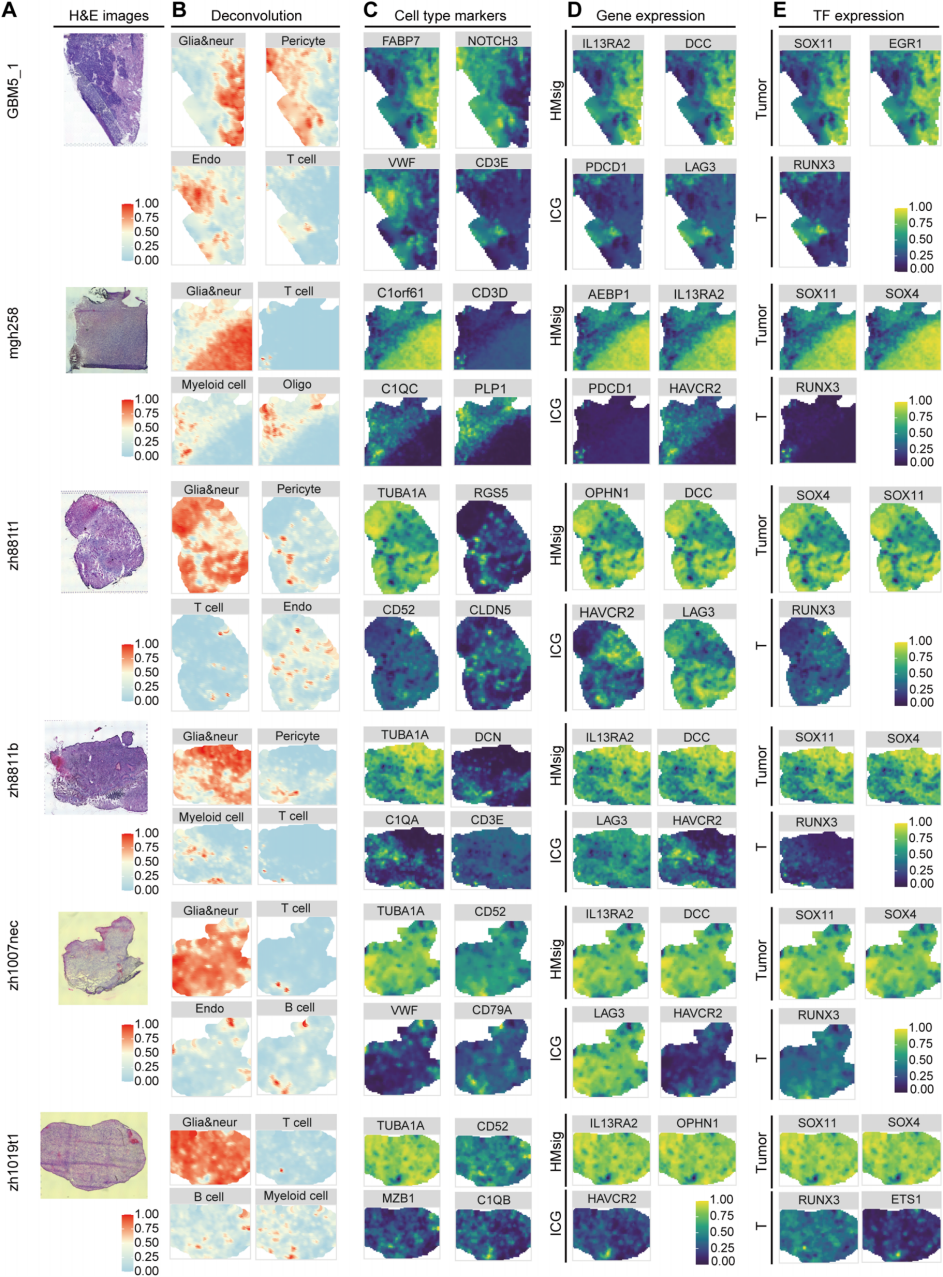
The spatial expression patterns of HMsig, ICG, and TFs. (A) H&E histological images of patients GBM5_1, mgh258, zh881t1, zh8811b, zh1007nec, and zh1019t1 reveal the histological architecture of the tumor slices. (B) Description of ST data using cell type deconvolution. The color of each spot represents the proportion of each cell type in all spots, with the color intensity indicating the relative abundance. (C–E) The expression levels of cell marker, HMsig, ICG, and TFs in representative samples. Dot colored by gene expression levels.

ST techniques allow high-resolution *in situ* measurement of gene expression, revealing expression gradients and identifying spatially organized cellular domains. To delineate the molecular and cellular architecture of GBM across anatomical regions, we collected 12 IDH-wt GBM spatial transcriptome samples (Fig. 7A), including tumors from the right temporal lobe, left frontal lobe, and other regions (Supplementary Table S1). To enable higher-resolution analysis at the cellular level, the CARD deconvolution algorithm was applied to map scRNA-seq-defined cell types onto ST data, covering glia/neuronal cells, oligodendrocytes, endothelial cells, pericytes, myeloid cells, T cells, and B cells (Fig. 7B). The tumor region’s delineation aligns with prior studies [87, 88], with regions predominantly mapped to glia/neuronal cells and oligodendrocytes, validated by the expression of marker gene (Fig. 7C).

The spatial distribution map of cells revealed interpatient heterogeneity. While GBM5_1 and zh881t1 both originated from the right temporal lobe, the tumor in zh881t1 nearly occupied the entire section, highlighting divergent tumor expansion patterns. Similarly, extensive tumor cell distribution was observed in left temporal lobe sections (Supplementary Fig. S5). Notably, 12 samples showed low abundance of T cells, indicating GBM’s inherent tendency to present as an immunologically “cold” tumor type. GBM5_1 exhibited extensive pericyte infiltration, consistent with the brain’s high vascularity and GBM’s angiotropic behavior. Pericytes, as key components of the neurovascular unit, may thus foster a microenvironment for tumor progression [89].

We further examined spatial expression patterns of HMsig, ICGs and TFs mediating regulatory interactions. HMsig such as IL13RA2, DCC, AEBP1 and OPHN1 showed high expression in tumor cells (Fig. 7D). In patient GBM5_1, the delineated tumor region was identified in study [87] as a hypoxic and invasive zone, and the remaining areas were divided into vascular enrichment areas. Additionally, the tumor tissue of patient GBM2 is located in the corpus callosum, and analysis indicates that the tumor area is primarily situated at the tissue margin, which may suggest an invasive tendency of the tumor along the corpus callosum-cortical junction area or perivascular spaces [90] (Supplementary Fig. S5). The high expression of HMsig in this area suggests that it may directly reflect hypoxia stress intensity, spatially colocalizing with tumor invasion or vascular-sparse regions. T cells exhibited enrichment of immune checkpoint molecules, suggesting that there is a state of T cell exhaustion in the TME.

Furthermore, the high expression of cell type-specific TFs suggests their spatially coordinated co-expression patterns (Fig. 7D and E). The tumor-associated TFs SOX4 and SOX11 demonstrate spatially co-regulated expression in diverse samples, underscoring their critical role in modulating HMsig. Meanwhile, T cell-related TFs are also markedly expressed in localized tumor areas, indicating a synergistic interplay between tumor cells and T cells in specific spatial regions. This interaction may contribute to the formation of an immunosuppressive TME. These findings are consistent with cellular distribution patterns and supported by bulk and single-cell transcriptome analyses, reinforcing the immunosuppressive nature of the TME.

## Discussion

This study characterized the TME of GBM by clustering cells from tumor tissues, revealing the existence of individual differences within the microenvironment. Our research demonstrated that hallmarks enriched at both the single-cell and bulk levels are pivotal to cell cycle regulation and profoundly influence the transcriptional governance of GBM. This suggests that investigating the regulation of cell cycle-related genes and TFs is essential for understanding the uncontrolled proliferation of GBM cells. Although numerous studies have explored the GBM microenvironment across both data modalities, no study has yet considered the hallmark features of malignant cells and the immune characteristics of immune “cold” and “hot” tumor samples. Due to the heterogeneity of GBM tissue and the existence of the BBB, there are significant differences in GBM patients’ TME in terms of the degree of immune cell infiltration, immune activity, and responsiveness to immunotherapy. This is also a challenge we need to face currently. We can more vividly reproduce the true state of the patient’s disease by identifying the characteristics of tumor malignant cells. Dividing GBM samples into immune “cold” and immune “hot” states according to immune characteristics can more accurately capture the immune-related features of GBM, and further deeply analyze and clearly present its immunosuppressive microenvironment. We obtained features from the hallmarks of single-cell tumor cells, the hallmarks of bulk immune “cold” samples, and the upregulated differentially expressed genes in bulk tumor cells, with the aim of achieving a more nuanced molecular profile of GBM across multiple scales. The five common hallmarks between single-cell and bulk dimensions confirm the transcriptional robustness of core malignant programs in GBM. These pathways, primarily associated with cell cycle regulation and proliferation, constitute a cross-modal conserved regulatory pattern, avoiding the influence of technical noise inherent in single-modal data. To ensure the clinical relevance of our model, we intersected these hallmark genes with bulk-level immune cold hallmarks and upregulated DEGs from bulk tumor samples. Leveraging this curated gene set, we implemented a combined machine learning framework to construct an optimal prognostic model, leading to the identification of HMsig. After multilayer verification, our optimized model has good predictive and validation efficiency.

In an effort to gain a more in-depth characterization and regulatory mechanisms of TME, we undertook cell communication and cell trajectory analysis. Cell communication analysis revealed that direct or indirect interactions between tumor cells and T cells are the primary cause of the immunosuppressive microenvironment in GBM. These interactions impair immune function, disrupting the TME balance. Notably, the OPHN1–EFNB1 interaction emerged as a critical determinant of poor prognosis. As EFNB1 drives glioma invasion and immune remodeling [91, 92], its interplay with OPHN1—a Rho-GAP modulating RhoA/Rac1 activity—likely governs cytoskeletal plasticity [93]. This synergy facilitates GBM cells to invade and spread more aggressively into the brain tissue. Cell trajectory analysis indicated that the upregulation of immune checkpoint molecules leads to a progressive decline in T cell function. This functional impairment enables tumor cells to escape immune surveillance, a key factor contributing to tumor growth and metastasis.

Through SCENIC analysis, RUNX3 was found to simultaneously regulate the expression of LAG3 and PDCD1. This dual regulation inhibits the immune response of T cells within the TME, further highlighting the complex role of TFs in modulating the immune landscape. Our analysis also showed that TFs exhibit synergistic effects not only within cell clusters but also between different cell clusters. This regulatory interplay significantly impacts patient prognosis. Consistent with the studies by Timothy F. et al. [94, 95], we found that PDCD1 and LAG3 were significantly upregulated in GBM multiforme. However, our study further revealed the mechanism by which RUNX3 simultaneously regulates these two genes through a shared motif, and this finding has not been reported previously. In the future, research on primary GBM could focus on using CRISPR–Cas9 genome editing technologies to verify the synergistic effect of RUNX3 and CEBPD in the process of immune escape, so as to better understand the dynamic immune evasion strategies in GBM. ST analysis further confirmed the co-localization of the HMsig genes with ICGs and their transcription factors (especially SOX4, SOX11, and RUNX3) in specific tissue regions, forming an immunosuppressive microenvironment. The co-expression of SOX4 and SOX11 suggests a spatial regulatory mechanism that enhances HMsig genes expression and drives tumor progression. RUNX3 upregulates the expression of immune checkpoint molecules LAG3 and PDCD1, further promoting the co-regulation represented by the “dual-brake” mechanism, thereby facilitating immune escape by tumor cells. This spatial organization driven by a coordinated transcriptional program provides a structural basis for the persistent treatment resistance in GBM.

In summary, this study revealed diverse immune escape mechanisms in GBM, including the interaction of key LR pairs between cell clusters, the upregulation of immune checkpoint molecules, and the synergistic regulation of RUNX3–CEBPD. These findings displayed the complexity of the TME and the refractory nature of GBM. Although this study identified only seven risk genes, five immune checkpoint molecules and a pair of synergistic TFs across cell clusters that affect patient prognosis, this may be due to the high heterogeneity and the limited representativeness of the tumor samples. Nonetheless, these results still offer fresh viewpoints and possible biomarkers that can enhance our comprehension of the proliferation and prognosis of GBM. Collectively, these results not only enhance our understanding of the complexity of the TME but also provide important theoretical foundations and new research directions for the diagnosis and treatment of GBM.

## Availability of source code and requirements

Project name: GBM-TIME

Project homepage: https://github.com/travilucas/GBM

Operating system(s): Platform independent

Programming language: R

Other requirements: R 4.2.0 or higher

License: MIT license

RRID:SCR_027948

## Additional files

**Supplementary Fig. S1**. Detailed characterization of the GBM microenvironment. (A–C) Clustree plots visualizing the clustering stability of major cell lineages (A), T cell subtypes (B), and myeloid cell subtypes (C) across varying clustering resolutions (0.1 to 1.0). Dot size represents cell count, and colors indicate clustering stability. (D) Box plot illustrating TotalCNV scores across different cell types to distinguish non-malignant clusters. The red dashed line represents the mean of the overall scores. (E, F) UMAP projections showing identified subclusters within T cells (E) and myeloid cells (F). (G, H) Dot plots showing the expression of canonical marker genes in myeloid subtypes (G) and T cell subtypes (H). Dot size indicates the proportion of expressing cells, and color intensity represents average expression levels. (I) Delta area plot showing the relative change in the area under the CDF curve for each *k* (*k* = 2–9).

**Supplementary Fig. S2**. Identification and assessment of the optimal model and HMsig. (A) Heatmap showing the C-index of 101 machine learning-based prediction models across the train and test sets, with the StepCox(forward) + RSF model identified as the optimal combination. (B) Kaplan–Meier curves of OS for high- and low-risk groups in the train set determined by the StepCox(forward) + RSF model. (C) Venn diagram illustrating the overlap of candidate genes between malignant-cell hallmark features, immune-related hallmark features, and up-regulated DEGs. (D) Horizontal bar plot displaying the genetic importance scores of the seven core HMsig genes. (E) Forest plot summarizing the meta-analysis of univariate Cox regression for the optimized model in both train and test cohorts. (F) Heatmap comparing the HR values of our optimized model with other previously published GBM signatures in both train and test sets. *P < 0.05, **P < 0.01.

**Supplementary Fig. S3**. The role of intercellular interactions and T-cell pseudotime dynamics in GBM. (A) Kaplan-Meier survival analysis of overall survival (OS) based on the co-expression of OPHN1 and EFNB1 LR pairs in the GSE7696 cohort. (B) Three-dimensional UMAP visualization of the T-cell developmental trajectory inferred by Monocle3, with cells color-coded by pseudotime beginning from the Naive_T cell cluster. (C) Gene expression dynamics of IL2RA along the T-cell pseudotime trajectory (D, E) Box plots showing the expression levels of immune checkpoint molecules KLRB1 (D) and CLEC2D (E) across T-cell subtypes. The color of dots is coded according to cell types. *P < 0.05, ^****^P < 0.0001; ns: no significant difference.

**Supplementary Fig. S4**. TFs exhibit synergistic effects between tumor cells and T cells. (A) Dot plot illustrating the regulon specificity score used to identify representative regulons for each cell type. The size and color gradient of the dots represent the magnitude and significance of the specificity scores. (B) Heatmap showing the correlation analysis of TFs in the TCGA–CGGA cohort, with colors indicating correlation coefficients and asterisks denoting significance (*P* < 0.05). (C–K) Scatter plots demonstrating the correlation between specific TF pairs at the single-cell level, with correlation coefficients (*r*) and *P*-values indicated in each panel. (L, M) Gene regulatory networks of TFs and their target genes in tumor cells (L) and CD8_T_EM cells (M). Warm-toned dots represent TFs, cold-toned dots represent target genes, and line colors indicate shared motifs.

**Supplementary Fig. S5**. The spatial expression patterns of HMsig, ICGs and TFs in GBM. (A) H&E histological images of patients GBM2, zh916bulk, zh881inf, zh8811a, zh1007inf, and zh1019inf, revealing the structural architecture of the tumor slices. (B) Spatial transcriptomics data visualized through cell type deconvolution across various samples, where color represents cell type and intensity indicates relative abundance. (C) Spatial expression maps of representative cell type markers used to validate deconvolution results. (D, E) Localized expression patterns of risk-related HMsig genes, ICGs and TFs within specific histological regions.

**Supplementary Data S1**. Comprehensive inventory of multidimensional transcriptomic datasets utilized in this study. This file provides a detailed overview of all data cohorts, including single-cell RNA-seq, bulk RNA-seq, and spatial transcriptomics datasets.

**Supplementary Data S2**. Optimized clustering resolution parameters and marker genes for cell type annotation. This table details the specific resolution settings used for clustering and catalogs the marker genes employed to identify major cell lineages and sub-clusters within T-cell and myeloid populations.

**Supplementary Data S3**. GO enrichment results of functional modules along T-cell trajectories. This dataset catalogs the biological processes and molecular pathways associated with the dynamic gene expression changes identified in the T-cell pseudotime analysis.

## Competing interests

The authors declare no competing interests.

## Supplementary Material

giag035_Supplemental_Files

giag035_Authors_Response_To_Reviewer_Comments_original_submission

giag035_GIGA-D-26-00037_original_submission

giag035_GIGA-D-26-00037_Revision_1

giag035_Reviewer_1_Report_original_submission Reviewer 1 -- 2/15/2026

giag035_Reviewer_2_Report_original_submission Reviewer 2 -- 2/17/2026

giag035_Reviewer_2_Report_revision_1 Reviewer 2 -- 3/20/2026

giag035_Reviewer_3_Report_original_submission Reviewer 3 -- 2/28/2026

## Data Availability

All datasets analyzed in this study are publicly available from established repositories. Single-cell RNA-seq data for GBM were obtained from the GEO under accession GSE182109 [96]. Bulk transcriptomic training cohort (*n* = 428) was assembled from TCGA (TCGA-GBM [97], TCGA-GBMLGG [98]) and CGGA (CGGA.mRNAseq_325.RSEM-genes [99], CGGA.mRNAseq_693.RSEM-genes [100]). Normal brain transcriptomic profiles (*n* = 105) were retrieved from GTEx project [101]. Independent validation microarray datasets were obtained from GEO under accessions GSE7696 [102], GSE42669 [103], GSE16011 [104], and GSE108474 [105]. Spatial transcriptomics data for primary IDH-wildtype GBM were obtained from GEO under accessions GSE194329 [87] and GSE237183 [88].

## Notes

Lead contact: Congxue Hu: hucx1996@hrbmu.edu.cn
