# Supplementary figures and images for: Dissecting glioblastoma risk signatures in the tumor immune microenvironment based on multi-dimensional transcriptomics
Source: Gigascience. 2026 Mar 25;15:giag035. doi: 10.1093/gigascience/giag035 (PMC13154832; doi:10.1093/gigascience/giag035)

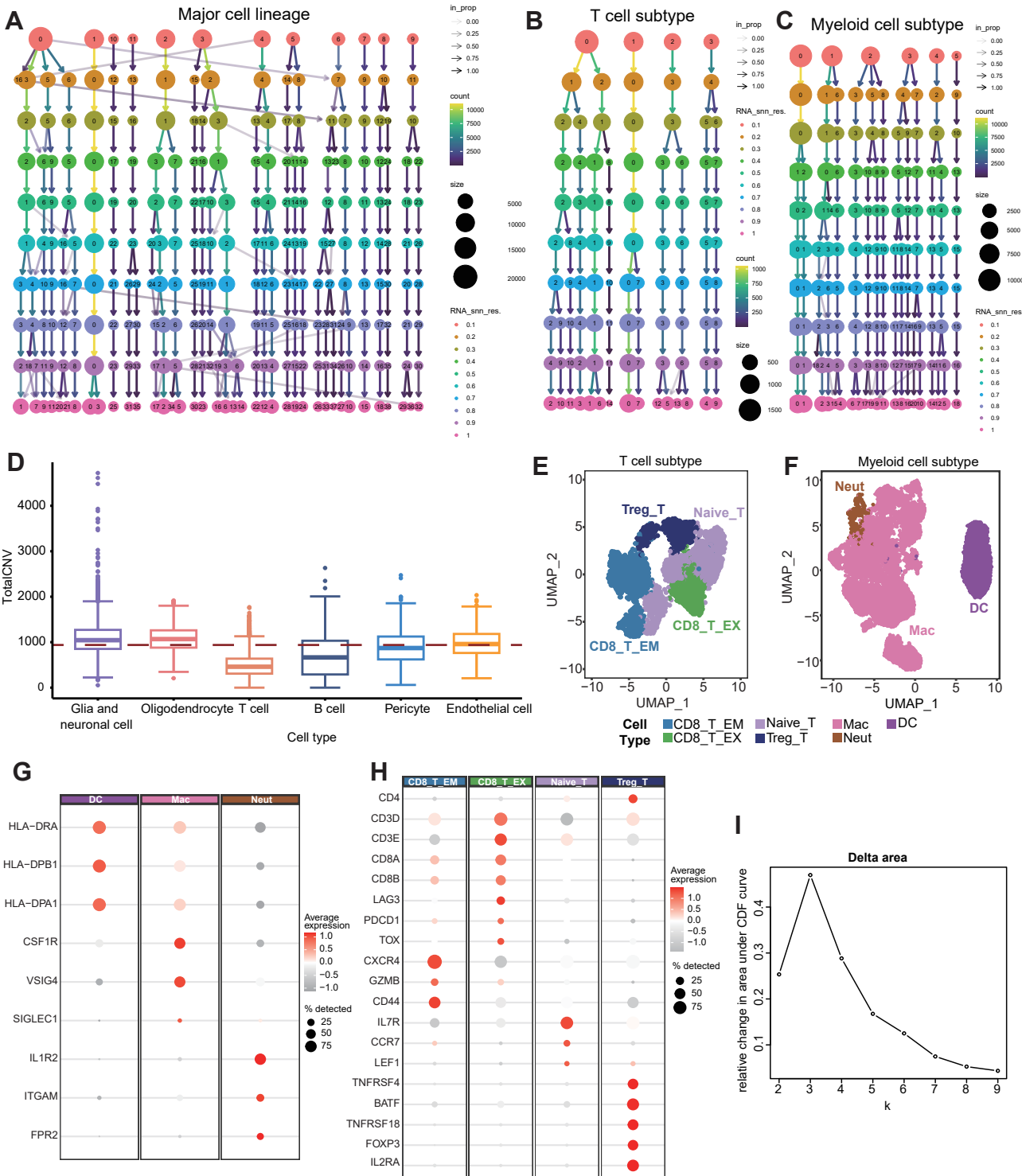

Supplement: giag035_Supplemental_Files [file giag035_supplemental_files.zip › Supplementary Figure1.pdf]

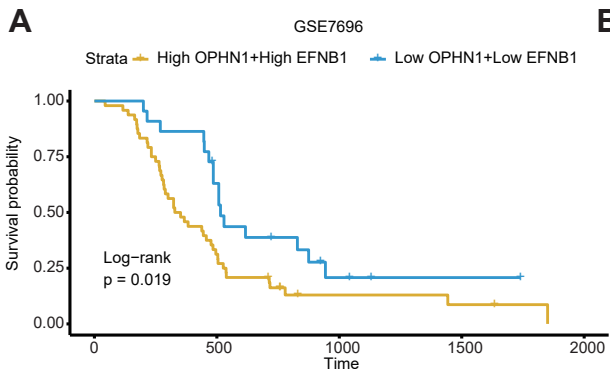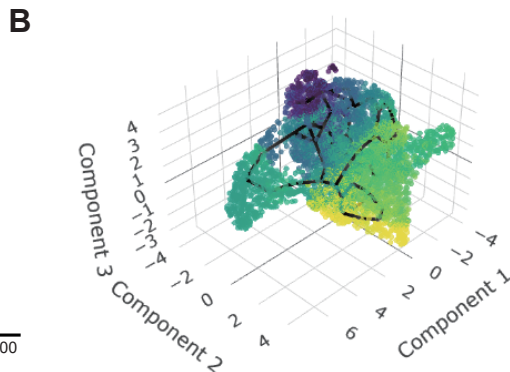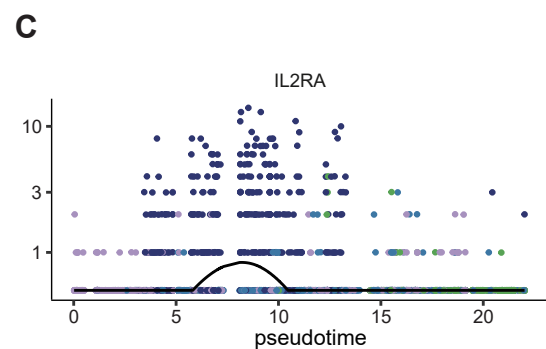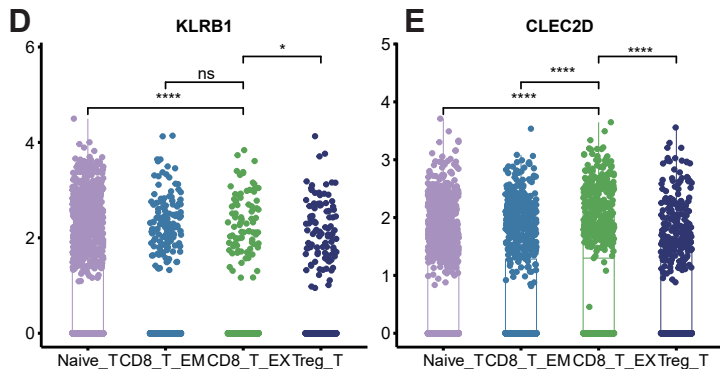

Supplement: giag035_Supplemental_Files [file giag035_supplemental_files.zip › Supplementary Figure3.pdf]

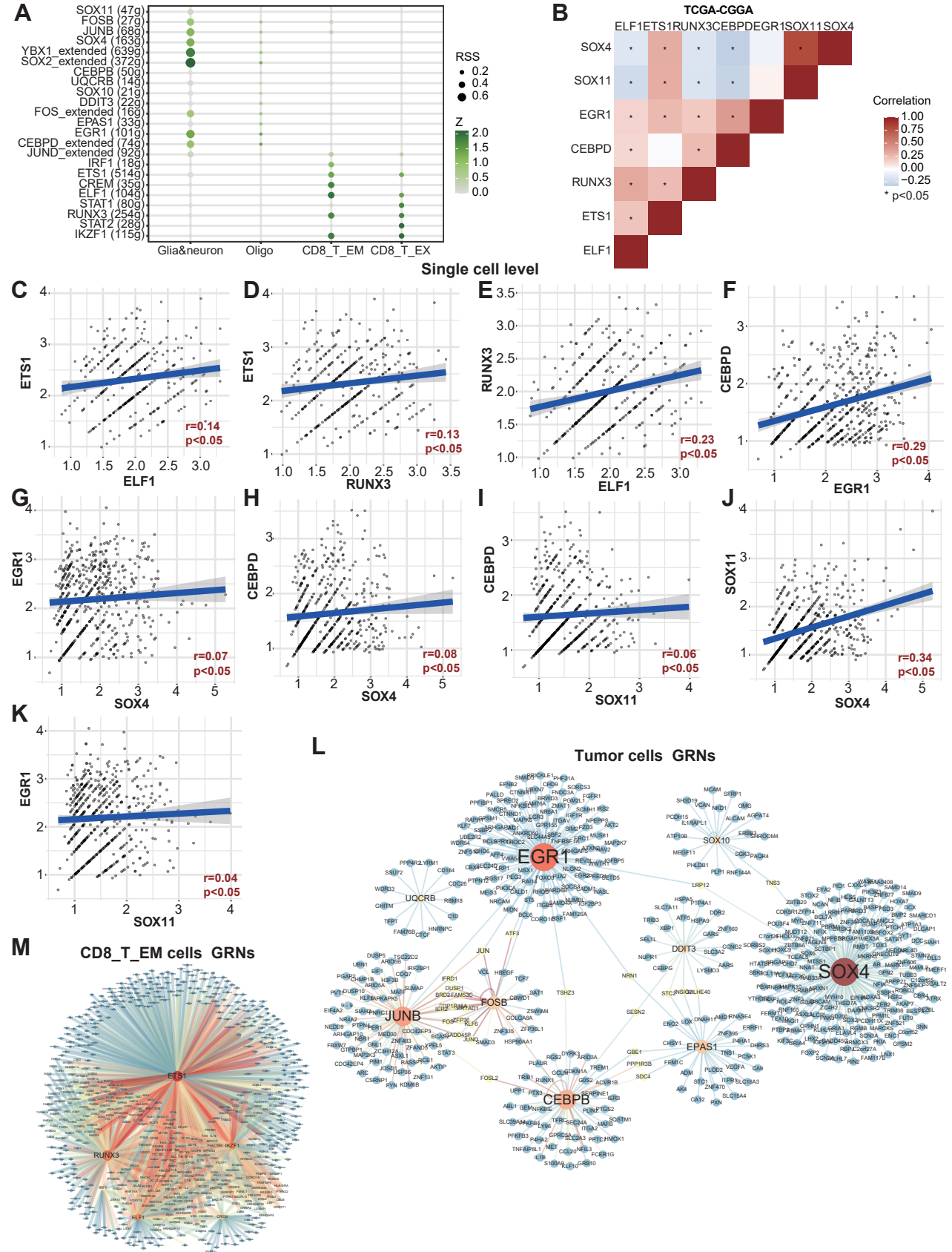

Supplement: giag035_Supplemental_Files [file giag035_supplemental_files.zip › Supplementary Figure4.pdf]

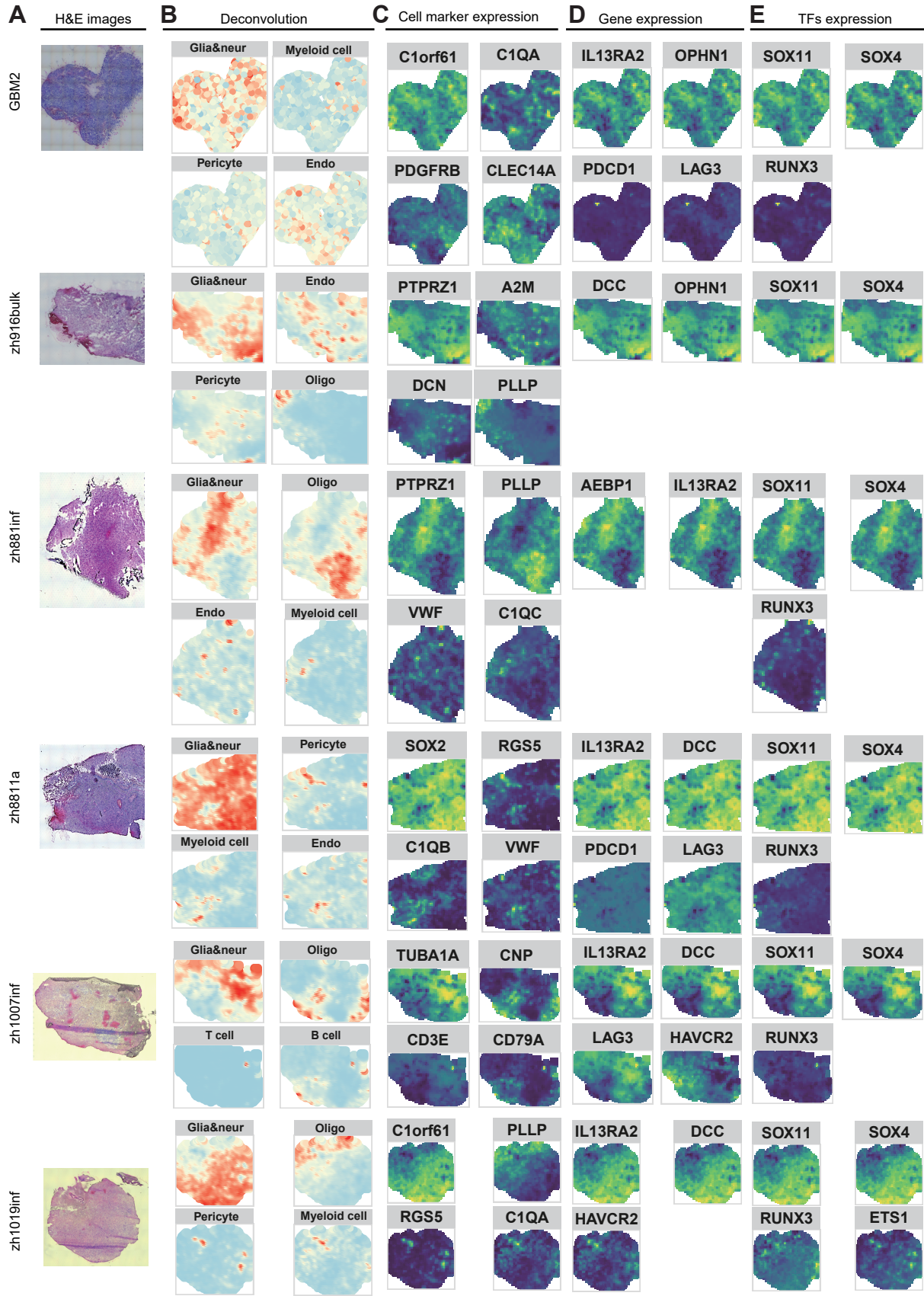

Supplement: giag035_Supplemental_Files [file giag035_supplemental_files.zip › Supplementary Figure5.pdf]
