# Supplementary material for: Dissecting glioblastoma risk signatures in the tumor immune microenvironment based on multi-dimensional transcriptomics
Source: Gigascience. 2026 Mar 25;15:giag035. doi: 10.1093/gigascience/giag035 (PMC13154832; doi:10.1093/gigascience/giag035)

GigaScience

Dissecting glioblastoma risk signatures in the tumor immune microenvironment based on multi-dimensional transcriptomics

--Manuscript Draft--

**Manuscript Number:**	GIGA-D-26-00037
**Full Title:**	Dissecting glioblastoma risk signatures in the tumor immune microenvironment based on multi-dimensional transcriptomics
**Article Type:**	Research
**Funding Information:**	National Major Science and Technology Projects of China (2024ZD0530500) — Prof Yunpeng Zhang<br>National Natural Science Foundation of China (62472131) — Prof Yunpeng Zhang<br>National Natural Science Foundation of China (62502128) — Dr Congxue Hu<br>Key Research and Development Program of Heilongjiang (2024ZX12C27) — Prof Yunpeng Zhang<br>China Postdoctoral Science Foundation (2024M760709) — Dr Congxue Hu<br>Heilongjiang Provincial Postdoctoral Science Foundation (LBH-Z24210) — Dr Congxue Hu<br>Longjiang New Era Outstanding Doctoral Dissertation Project Grant (LJYXL2024-069) — Dr Congxue Hu<br>Harbin Medical University Fund (31031250051) — Dr Congxue Hu
**Abstract:**	Glioblastoma (GBM) is characterized by pronounced tumor heterogeneity and a complex immune microenvironment, contributing to poor patient survival outcomes. In this study, we comprehensively dissected the tumor microenvironment (TME) and uncovered potential molecular mechanisms by integrating single-cell, bulk, and spatial transcriptomic data. Hallmarks of malignancy and cell cycle regulatory pathways were consistently enriched across these modalities, highlighting their pivotal roles in transcriptional regulation. We identified seven hallmark-related prognostic signatures (HMsig) using machine learning algorithm, namely AEBP1, ASF1A, PRPS1, DCC, OPHN1, IL13RA2, and HDAC5—whose importance in predicting patient outcomes was validated through SHAP algorithm analysis. Ligand-receptor (LR) interaction analysis revealed that interactions involving OPHN1 were associated with poorer prognosis. Additionally, Immune checkpoint genes (ICG) LAG3, PDCD1, and HAVCR2 were found to be substantially upregulated along the pseudotime trajectory of T-cell progression. Synergistic transcriptional regulation between tumor-related HMsig signatures and ICGs in T cells was identified as a key factor influencing patient survival. Spatial transcriptomic analysis consistently demonstrated the existence of synergistic gene interactions, deciphering the immunomodulatory functions of GBM biomarkers in the TME.
**Corresponding Author:**	Congxue Hu, Ph.D<br>Harbin Medical University<br>Harbin, Heilongjiang Province CHINA
**Corresponding Author Secondary Information:**	
**Corresponding Author's Institution:**	Harbin Medical University
**Corresponding Author's Secondary Institution:**	
**First Author:**	Tengyue Li

Powered by Editorial Manager® and ProduXion Manager® from Aries Systems Corporation

First Author Secondary Information:	
Order of Authors:	Tengyue Li
	Wanqi Mi
	Huarui Yan
	Yining Ma
	Han Jiang
	Xiaoxu Yang
	Yunpeng Zhang
	Congxue Hu, Ph.D
Order of Authors Secondary Information:	
Additional Information:	
Question	Response
Are you submitting this manuscript to a special series or article collection?	No
**Experimental design and statistics**<br><br>Full details of the experimental design and statistical methods used should be given in the Methods section, as detailed in our Minimum Standards Reporting Checklist. Information essential to interpreting the data presented should be made available in the figure legends.<br><br>Have you included all the information requested in your manuscript?	Yes
**Resources**<br><br>A description of all resources used, including antibodies, cell lines, animals and software tools, with enough information to allow them to be uniquely identified, should be included in the Methods section. Authors are strongly encouraged to cite Research Resource Identifiers (RRIDs) for antibodies, model organisms and tools, where possible.<br><br>Have you included the information requested as detailed in our Minimum Standards Reporting Checklist?	Yes

Powered by Editorial Manager® and ProduXion Manager® from Aries Systems Corporation

**Availability of data and materials**<br><br>All datasets and code on which the conclusions of the paper rely must be either included in your submission or deposited in publicly available repositories (where available and ethically appropriate), referencing such data using a unique identifier in the references and in the "Availability of Data and Materials" section of your manuscript.<br><br>Have you have met the above requirement as detailed in our Minimum Standards Reporting Checklist?	Yes
GigaScience has policies and guidelines in place for the use of generative AI-writing tools such as ChatGPT. If you have used such writing tools to assist with writing the manuscript this must be declared and cited in the text. Authors should not list AI-writing tools and other AI-assisted technologies as an author or co-author and should acknowledge that they are fully responsible for text generated or refined by AI-writing tools.<p><br><br>A summary of use (particularly in the introduction or among methods) needs to be included at the end of the paper, and the outputs should also be included as a supplementary file hosted in GigaDB or other open repositories. Please read our guidelines for more information. <p><br><br>By submitting to GigaScience, you are aware of the journal's AI-writing tools policy, and if you have declared use of such tools below, you have acknowledged this where appropriate in your manuscript and have made a summary of use and outputs available. <p><br><b>AI-assisted writing tools have been used in the preparation of this manuscript?	No

Manuscript Click here to access/download;Manuscript;Manuscript.docx

Dissecting glioblastoma risk signatures in the tumor immune microenvironment based on multi-dimensional transcriptomics

Tengyue Li[1] #, Mi wanqi[1] #, Huarui Yan[1], Yining Ma[1], Han Jiang[1], Xiaoxu Yang[1], Yunpeng Zhang[1]*, Congxue Hu[1]*

1College of Bioinformatics Science and Technology, Harbin Medical University, Harbin 150081, China

Lead contact: Congxue Hu: hucx1996@hrbmu.edu.cn

#These authors contributed equally to this work.

*Correspondence: Congxue Hu: hucx1996@hrbmu.edu.cn

Abstract

Glioblastoma (GBM) is characterized by pronounced tumor heterogeneity and a complex immune microenvironment, contributing to poor patient survival outcomes. In this study, we comprehensively dissected the tumor microenvironment (TME) and uncovered potential molecular mechanisms by integrating single-cell, bulk, and spatial transcriptomic data. Hallmarks of malignancy and cell cycle regulatory pathways were consistently enriched across these modalities, highlighting their pivotal roles in transcriptional regulation. We identified seven hallmark-related prognostic signatures (HMsig) using machine learning algorithm, namely AEBP1, ASF1A, PRPS1, DCC, OPHN1, IL13RA2, and HDAC5—whose importance in predicting patient outcomes was validated through SHAP algorithm analysis. Ligand-receptor (LR) interaction analysis revealed that interactions involving OPHN1 were associated with poorer

prognosis. Additionally, Immune checkpoint genes (ICG) LAG3, PDCD1, and HAVCR2 were found to be substantially upregulated along the pseudotime trajectory of T-cell progression. Synergistic transcriptional regulation between tumor-related HMsig signatures and ICGs in T cells was identified as a key factor influencing patient survival. Spatial transcriptomic analysis consistently demonstrated the existence of synergistic gene interactions, deciphering the immunomodulatory functions of GBM biomarkers in the TME.

Keywords: Glioblastoma; Tumor Microenvironment; Malignant Tumor Hallmarks; Gene Regulatory Networks; Cooperative TFs; Biomarkers

Introduction

Being a high-grade glioma assigned a World Health Organization (WHO) Grade IV, GBM stands as one of the tumors that resist cure most stubbornly. Among primary brain cancers, GBM is the most aggressive and occurs most frequently, with a median survival time of only 14-18 months[1]. Currently, the recurrence rate of GBM patients after treatment exceeds 90%. Only a negligible number of patients are likely to approach a state of cure. It's important to stress that the outlook for GBM patients is extremely poor. As a result, gaining a deeper understanding of the diversity of the pathological biology of GBM and exploring its potential TME characteristics is paramount.

Unsatisfactory therapeutic outcomes for GBM may be multifactorial, including the decline of T cell killing function, defects in tumor antigen presentation, and characteristics of the tumor's physical microenvironment. In brain tumors, there are more resident microglia and macrophages compared to infiltrating T cells[2]. Various studies support the hypothesis that GBM is an immunologically "cold" tumor[3]. In some patients, low-level immune infiltration may be attributed to the presence of the blood-brain barrier (BBB)[4]. Moreover, the scarcity of T cells in the TME starkly contrasts with findings in melanoma, lung cancer, and other tumor types[5]. Whether GBM is inherently non-immunogenic and the regulatory mechanisms between tumor cells and immune cells in the TME require further investigation.

The significant heterogeneity observed in GBM is largely driven by its regulation of core hallmarks. More precisely, the control of GBM cell proliferation, self-renewal,

and the inactivation of differentiation is mediated by the Wnt, Notch, and TGF-β signaling pathways[6]. The WNT/β-catenin cascade is closely associated with various malignant tumors. In normal cells, the Wnt signaling pathway is typically in an inactive state and is highly conserved[7]. Abnormal activation of the Wnt signaling promotes cancer cells to escape immune surveillance, inhibits T cell infiltration, and mediates the anti-tumor immune response[8]. This pathway has recently become an important determinant in the occurrence and development of GBM. GBM is also involved in alterations of multiple metabolic hallmarks, such as glycolysis, oxidative phosphorylation, and hypoxia[2]. GBM utilizes various unconventional molecules to sustain its growth as well. Hypoxia stimulates adenosine monophosphate-activated protein kinase (AMPK), which in turn regulates the energy acquisition of tumor cells[9]. Additionally, hallmarks related to transcriptional regulation are also involved in the development of tumor tissues. E2F7, a member of the E2F family transcription factors (E2Fs), not only participates in cell cycle regulation as a transcriptional repressor[10, 11], but also contributes to tumor cell proliferation and metastasis. Besides, studies have found that E2F7 may be involved in mediating immune cell infiltration[12, 13]. Thus, transcriptional regulation affects not only tumor cells themselves but also tumor progression by modulating the cellular and molecular components in the TME[14]. In published GBM related studies, the synergistic action of TFs is often observed within the same cell type[11, 15, 16]. However, the transcriptional regulatory mechanisms between TFs across different cell clusters remain unclear.

GBM diffusely infiltrates the brain, intermingling with non-neoplastic brain cells.

This intricate TME forms the biological basis for treatment response and tumor recurrence. It is of utmost importance to delve deeply into the interactions between GBM cells and their immune microenvironment. However, the knowledge in this regard is currently insufficient. The enhanced resolution of single-cell sequencing can be utilized to uncover the key characteristics of highly heterogeneous tumors. Accordingly, we integrated transcriptome data from both bulk and single-cell level. We screen for biomarkers and identify risk genes in GBM by employing machine learning techniques like stepwise Cox regression (StepCox) and random forests (RSF). Meanwhile, we utilize multi-omics analysis to identify TF-Gene activity regulation networks and explore the transcriptional regulatory mechanisms between tumor cells and T cells.

Materials and methods

Data collection

scRNA-seq data for GBM, GSE182109[17], were obtained from the Gene Expression Omnibus (GEO, https://www.ncbi.nlm.nih.gov/geo/). This study utilized 9 primary wild-type GBM samples to characterize the TME of primary GBM. For bulk transcriptome data, 769 primary GBM samples with survival information were collected from the GEO, the Cancer Genome Atlas (TCGA, https://xena.ucsc.edu), and the Chinese Glioma Genome Atlas (CGGA, http://www.cgga.org.cn/) databases. 105 normal brain samples were obtained from the Genotype-Tissue Expression (GTEx, http://www.gtexportal.org/home/) database for subsequent analysis. Furthermore, microarray sequencing data and survival data from the GEO datasets (GSE7696[18], GSE42669[19], GSE16011[20], and GSE108474[21]) were acquired to validate prognostic markers. Spatial transcriptome data for GBM (GSE194329[22] and GSE237183[23]) were collected and 12 primary wild-type GBM samples were used to analysis. For detailed information on the data, please refer to Supplementary Table 1.

Data preprocessing

For single-cell level data, cells expressing fewer than 200 genes or more than 2,500 genes were filtered out, and cells with mitochondrial gene content exceeding 20% were removed to ensure the quality of cells used in downstream analysis. After quality control filtering, 71,836 cells remained. A gene was retained if it was expressed in at least 3 cells. Following the removal of unqualified genes, 29,622 genes were retained

for analysis. For bulk level data, data from the TCGA and CGGA platforms were converted into transcripts per million (TPM). The Combat algorithm (sva R package, version 3.35.2) was used to remove batch effects from the bulk RNA-seq expression profiles of the TCGA and CGGA platforms. For cases where multiple ENSEMBL ids map to the same gene, we calculate the average expression level of each gene using "rowMeans" and select the gene with the highest expression level. The log2(x+1) transformation were performed to generate clean microarray data. For spatial transcriptomics (ST) data, we loaded raw 10x Visium data including gene expression matrixs, spatial coordinates, and tissue images using the "Load10X_Spatial" function, followed by data normalization and variance stabilization with "SCTransform" while retaining all genes for downstream analysis.

Dimensionality reduction and clustering of cells

The preprocessed gene expression matrix and cell annotation information were processed using the Seurat R package (version 5.1.0). The top 2,000 highly variable genes, identified using the standard deviation (SD) algorithm, were used for principal component analysis (PCA). Expression profiles were normalized using the LogNormalize method (feature counts per cell divided by the total counts for that cell, multiplied by a scale factor). To maximize the explanation of data variability with the fewest principal components, the top 16 principal components were manually selected for cell clustering analysis using the UMAP algorithm. The Harmony algorithm was implemented to mitigate batch effects among tumor samples during data integration.

Cell clusters were defined using marker genes specific to cell types in GBM tissues, collected from published literature and the CellMarker2.0.

Identification of malignant tumor cells

The inferCNV algorithm (https://github.com/broadinstitute/infercnv) was used to infer copy number states by calculating the ratio of gene expression levels in tumor cells to the average expression levels in one or more reference normal cells or cell populations in scRNA-seq data. For the dataset GSE182109, T cells and B cells were selected as reference normal cells for gene expression. All cell clusters, except myeloid cells, were used to calculate chromosomal copy number variations (eg: amplifications or deletions). The minimum average read count threshold for each gene in the reference cells of 10X genomics data is set to 0.1. Malignant cells were identified by comparing copy number patterns between normal cells and other cell clusters.

Immune phenotype of tumors based on transcriptomic profiling

Immune "hot" tumors refer to tumor types in which there is a large infiltration of immune cells in the tumor microenvironment and an active anti-tumor immune response is presented. Immune "cold" tumors lack immune cell infiltration and have a weak anti-tumor immune response. Differentially expressed genes (DEGs) for each immune cell clusters were ranked based on fold change values. We selected the top 50 DEGs for each immune cell type as the immune cell gene set. By comparing the gene expression data of each tumor sample with the immune cell gene set, a gene set enrichment score (GSES) was obtained to estimate the relative enrichment of the gene

set in each sample. Tumor samples were clustered using ConsensusClusterPlus (version 1.68.0) based on GSES. The tumor samples were divided with K=2, and the tumor samples with significantly enriched immune cells were defined as immune "hot" tumors. The GSES was normalized by the “scale” function, and half-violin plots and heatmaps were generated to visualize the enrichment results before and after scaling. Immune scores were calculated using “Estimate” (version 1.0.13) to validate the ssGSEA results.

Gene set enrichment analysis (GSEA)

We used the fgsea (version 1.13.0) R package to test the enrichment of hallmark genesets downloaded from MsigDB (msigdbr R package version 7.5.1). For single-cell level, the Wilcoxon rank sum test (presto R package version 1.0.0) was used to calculate the DEGs and the AUC value was used as the ranking index to generate the pre-sorted list of genes. For bulk-level input, a pre-ranked gene list generated from differential expression analysis using the limma R package (version 3.60.2) was applied. Genes with P-value<0.05 and |logFC|>1 were set as significant DEGs. The GSEA was used for 1000 permutation tests, and NES>0 and P.adj.value<0.05 were defined as significant up-regulation of hallmark.

Machine learning model construction and validation

Univariate Cox regression analysis was performed to identify prognostic features from input variables. Genes with a p-value<0.05 were considered prognostically significant. These genes were input into a machine learning framework. We used the established machine learning framework R package mime1[24] and constructed models

on the training dataset using 10-fold cross-validation. The model with the highest C-index in the validation set was selected as the optimal model due to its highest accuracy and lower risk of overfitting. To comprehensively evaluate the model, we conducted meta-analysis integrating both training and validation datasets, performed time-dependent ROC analyses for 1, 3, and 5-year survival predictions in GBM patients, and compared model performance against existing approaches using C-index and ROC values.

HMsig importance assessment

We employed the SHAP algorithm (fastshap R package version 0.1.1) to quantitatively assess the prognostic influence of filtered hallmark signature genes (HMsig). A survival analysis model was constructed through a combined machine learning algorithm model of forward stepwise Cox regression and random forest. Then, the “explain” function was used to calculate the SHAP value for each feature (HMsig) of each sample to measure their impact on the survival prediction results of patients. Visualize the importance of features through the “sv_importance” function.

Cell communication analysis

Cell communication analysis was performed by CellChat[25] (version 1.6.1). We employed the established CellChat method to investigate interactions between tumor cells and immune cells. CellChatDB integrates signaling interaction information from the KEGG pathway database and literature from experimental studies. The human LR database from CellChatDB was used as a reference to evaluate cell communication

networks between two cell types. DEGs across all cell clusters were identified using the Wilcoxon rank-sum test (Pvalue<0.05). To account for noise effects, the triMean quartile method was used to calculate the average expression of LR pairs in cell clusters.

Construction of HMsig-LR interaction network

The STRING database (https://string-db.org/) enables the exploration of potential interactions between genes, constructing interaction networks that illustrate relationships such as physical contacts or regulatory targeting among HMsig and LR pairs. We input the list of HMsig and LR pairs (identified through Cellchat) into the STRING database to obtain potential gene interactions. Subsequently, the gene interaction network was constructed and visualized using Cytoscape (https://cytoscape.org).

T cell developmental trajectory

Based on previous studies defining various T cell characteristics, we analyzed T cells and identified four T cell subtypes, which are: Naive T, Treg T, and CD8 T_EM cells and CD8 T_EX cells. Pseudotime trajectories for these T cell types were constructed using Monocle3[26] (version 1.3.3), a semi-supervised pseudotime analysis algorithm approximating PAGA. Dimensionality reduction was performed using the UMAP algorithm via the “reduceDimension” function. Naive_T cells were set as the trajectory starting point using the “get_earliest_principal_node” function. Pseudotime trajectories were built using the “learn_graph” function, and temporal distribution

density curves for the four T cell types were plotted using the ggridges R package (version 0.5.6).

Gene ontology enrichment analysis (GO)

GO enrichment analysis was performed using clusterProfiler (version 4.12.0) and org.Hs.eg.db (version 3.19.1). For the pseudotime developmental modules of T cells, GO enrichment analysis was performed based on significantly DEGs within each module. Regarding the transcriptional regulatory sub networks of tumor cells and T cells, GO enrichment analysis was conducted on genes driven by cell type-specific TFs.

Construction of transcriptional regulatory networks

We used SCENIC[27] R package (version 1.3.1) to identify regulons in single-cell transcriptomes. Single-cell datasets from two tumor cell types, CD8_T_EM, and CD8_T_EX were used as input. The GENIE3 algorithm was employed to construct regulatory networks based on motifs and ranked binding sites from RcisTarget. Regulon activity was calculated using AUCell. The regulon specificity score (RSS) for TFs in each cell type was computed using the "calcRSS" function (zThreshold = 0.1, thr = 0.1), to identify cell type-specific TFs. A binary matrix heatmap was generated, and TF-gene regulatory networks were constructed using Cytoscape (version 3.9.1). TF pairs with $r > 0.2$ and $p < 0.05$ were considered significantly positively correlated, indicating synergistic interactions. The "viewMotifs" function was used to examine motifs corresponding to TF-HMsig/ICG pairs, exploring the mechanisms of TF synergy.

Survival analysis

Survival analysis was conducted using the Survival (version 3.6.4) and survminer (version 0.4.9) R packages. For survival analysis of HMsig validated in GEO data, the StepCox(forward)+RSF model was used to calculate risk scores for each patient. Patients were stratified into high and low risk groups based on the median risk score, and differences in clinical outcomes were assessed using the Log-rank test. Kaplan-Meier curves were used to visualize survival characteristics.

To investigate the impact of HMsig ligand-receptor gene interactions on GBM patient survival, we specifically evaluated the prognostic value of OPHN1-EFNB1 interaction patterns. Accounting for gene-gene interaction effects, we calculated the product of both genes' expression values and the optimal cutoff threshold for stratifying high versus low expression groups was determined using the "surv_cutpoint" function. Differences in clinical outcomes were assessed using the Log-rank test, and Kaplan-Meier curves were generated to visualize survival characteristics.

For survival analysis of synergistic TF pairs in TCGA-CGGA, the mean expression sum of each TF pair was used as the threshold to stratify patients into high and low-expression groups. Differences in clinical outcomes were assessed using the Log-rank test, and Kaplan-Meier curves were generated to visualize survival characteristics.

Cell type deconvolution

The cell-type compositions for each spot were determined using the conditional autoregressive deconvolution (CARD) algorithm[28] (version 2.2.0). CARD object was

constructed from the ST data along with the scRNA annotated data described above to reference scRNA-seq profiles encompassing 7 cell types. CARD modeling uses spatial autoregressive priors and Bayesian statistical inference to estimate cell type proportions per spot, outputting a normalized abundance matrix of spatial cell distributions. We applied “CARD.imputation” function to improve CARD-based expression, enabling high-resolution mapping of both cell-type localization and imputed gene activity. Optimized ST data were chosen to reveal cellular distributions and gene expression patterns.

Statistical analysis

The results of the statistical analysis are presented using box plots and violin plots. The statistical significance of differences between groups was determined using the unpaired, two-tailed student t-test, with $P < 0.05$ considered statistically significant. The correlation analysis of TFs was performed using Spearman's correlation coefficient. These statistical analyses were performed using R software 4.4.1.

Result

Result 1 Identifying molecular hallmarks for malignant cells

After rigorous quality control and normalization, the gene expression profiles of 71,836 cells from 9 GBM patients were used for in-depth analysis. We employed the Uniform Manifold Approximation and Projection (UMAP) clustering algorithm, with which all cells were annotated into 7 distinct clusters. These clusters included B cells, epithelial cells, glial and neuronal cells, myeloid cells, oligodendrocytes, pericytes, and T cells. Notably, the clusters of glial and neuronal cells and myeloid cells accounted for a much larger proportion of the total cell population than B cells and T cells in GBM patients. Moreover, in certain patients, such as MDAG-1 and MDAG-7, a relatively low level of immune cell infiltration was observed, which prominently emphasizes the momentous individual heterogeneity within GBM tissues (Figure 1A-B, Supplementary Table 2).

To accurately distinguish the malignancy level of tumor cells, the inferCNV algorithm was utilized to compute the copy number variation based on the single-cell RNA sequencing (scRNA-seq) data (Figure 1C). Since glial and neuronal cells are recognized as the main origin of malignant GBM cells, those two types of nerve cells with a CNV score exceeding the mean were defined as high malignant. Conversely, 4,829 cells were designated as low malignant according to their CNV scores. CNVs were also detected in pericytes and epithelial cells (Supplementary Figure 1A). Subsequently, all high malignant and low malignant tumor cells were extracted and

subjected to UMAP algorithm once again to visualize the distribution of tumor cells (Figure 1D).

In order to delve deeper into the unique molecular hallmarks of malignant cells, a differential expression analysis was carried out between the high malignant and low malignant tumor cells. This analysis disclosed that several genes, such as IGFBP2 and VBP1, were significantly upregulated (Figure 1E). IGFBP2 promotes glioma progression by activating the PI3K/AKT pathway and MMP2[29], while VBP1 regulates hypoxia responses by degrading HIF-1α, a key transcriptional regulator[30]. GSEA results revealed that the markedly enriched hallmark pathways encompassed the KRAS signaling pathway, the NOTCH signaling pathway, oxidative phosphorylation (OXPHOS), spermatogenesis along with an additional 24 markedly up-regulated hallmarks (Figure 1F). In GBM, normal cells mainly rely on aerobic oxidation of glucose to generate energy. However, tumor cells, in addition to enhanced glycolysis, also utilize the OXPHOS to produce more adenosine triphosphate (ATP), providing ample energy for their abnormal biological behaviors. Beyond the tumor-related metabolic pathways, numerous studies have demonstrated that the Notch signaling pathway, which is associated with cell proliferation, is over-active in GBM[31]. The diversity of these hallmarks implies the complex TME of GBM.

Fig. 1. Single-cell transcriptome analysis of human glioma maligant cells. A UMAP projections of 71,836 aggregate single cells from 9 patients showing the composition of different cell types in human gliomas (upper panel). The stacked bar plot illustrates the proportion of each cell type across the 9 patients (lower panel). The colors of the dots and bars represent different cell types. B Dotplot

shows the expression of marker genes in different cell types in GBM. Dot size indicates the proportion of expressing cells, colored by average expression levels. C Heatmap shows the normal/malignant cell status determined by inferCNV analysis. It displays the relative expression intensity of each cell across various chromosomal regions, with colors representing CNV levels. The upper heatmap represents the results for the reference cells, while the lower heatmap represents the results for the observation cells. D UMAP projections are shown by malignant status. E Differential expression analysis of tumor malignant cells. The size and color gamut of the dots are determined by the -log10(padj) values. F GSEA analysis of tumor malignant cells. The size and color of the dots are determined by the normalized enrichment score (NES) values. The padj_score is derived by -log10(padj).

Result 2 Dissecting immune hot and cold tumors in GBM through single-cell and Bulk transcriptomics

In GBM, the microenvironment is typically characterized as "cold" due to limited immune infiltration[32]. The study found that the immune microenvironment significantly influences patient prognosis[33]. To systematically dissect these features and their clinical implications, we classified tumors into immune "cold" and "hot" states at the bulk level, integrating single-cell data to resolve limitations of single-modality analysis. We initially conducted subtype identification on T cells and myeloid cells. T cells were further classified into naive T cells, regulatory T cells (Treg), CD8 effector memory T cells (CD8_T_EM) and CD8 exhausted T cells (CD8_T_EX). Myeloid cells were identified as macrophages, dendritic cells and neutrophils (Supplementary Figure 1B-E). Based on the results of single-sample gene set

enrichment analysis (ssGSEA) of the top 50 differentially expressed genes in 8 immune cell clusters at the single-cell level, we conducted consensus clustering analysis. In that way, all GBM samples were divided into k (k=2–9) clusters. The cumulative distribution function (CDF) curve of the consensus score matrix and the delta area plot indicated that the optimal number was obtained when k=2 (Figure 2A-B, Supplementary Figure 1F). The two clusters (Cluster 1 and Cluster 2) exhibited clear differences in immune infiltration, in which B cells, Treg cells, CD8_T_EM cells, and other immune cells exhibit pronounced enrichment in the tumor samples of Cluster 2. Undoubtedly, the immune infiltration abundance in Cluster 2 was remarkably higher compared to Cluster 1 (Figure 2C-D). Therefore, we defined Cluster 1 as an immune "cold" tumor and Cluster 2 as an immune "hot" tumor. We noticed neutrophils are significantly enriched in immune-hot tumors. Neutrophils exert anti-tumor effects by directly killing tumor cells through ROS, NO, and granular protein release[34-38]. This implies neutrophil enrichment helps differentiate immune "cold" from immune "hot" states.

We also explored the distribution of patient gender, age, and survival status within immune "cold" and "hot" tumor samples. It is undeniable that the number of immune "hot" tumor samples is markedly less than that of immune "cold" tumors. Among GBM patients, the proportion of patients in the survival state was significantly lower than that of deceased patients. Considering the overall GBM patient population, the prognosis of male patients is generally worse than that of female patients[39]. Nevertheless, upon closer examination of the immune "hot" tumor cluster, an intriguing phenomenon

emerged. Among the living patients under 60 years old, all were male. This finding implies that within a specific GBM tumor immune subtype and age range, males may possess some undiscovered survival advantages, which deserves further research (Figure 2C). When additionally observing the relationship between immune cell infiltration and the TME, we noticed the special behavior of B cells in immune "cold" tumors. Regarding the enrichment phenomenon of B cells in immune "cold" tumors, it may be associated with an immunosuppressive function[40]. We employed the ESTIMATE algorithm to verify the analysis results, which corroborated the outcomes of our previous ssGSEA (Figure 2E).

We performed differential expression analysis on immune "cold" tumors and immune "hot" tumors, in order to deepen our understanding of the molecular characteristics of GBM hallmarks (Figure 2F). CCL20 and CXCL5, as chemokines, could attract immune cells (such as T cells and monocytes) into the TME. Their downregulation implies a weakened ability to recruit immune cells, leading to insufficient infiltration of immune cells in tumor tissues and thus giving rise to the "cold" tumor phenotype. Simultaneously, the GSEA result revealed that six significantly upregulated pathways, including DNA repair, Myc-targets-v1, KRAS signaling pathway, etc (Figure 2G). The IL2_STAT5, IL6_JAK_STAT3, and inflammatory response hallmarks are remarkably downregulated in immune "cold" tumors (Figure 2G). In the TME, the JAK-STAT signaling pathway is essential for maintaining the homeostasis of immune cells. STAT5 and STAT3, as dominant TFs in this pathway, can influence genes related to cell proliferation, apoptosis, and immune responses. STAT5

acts as a catalyst for promoting the proliferation of T cells; STAT3 is indispensable for maintaining the cell identity of macrophages[41]. The downregulation of their related pathways leads to the decline of immune killing functions and spurs the generation of an immunosuppressive microenvironment. There were five common hallmarks across both data modalities, namely the E2F transcription factor family, G2/M checkpoint, spindle mitosis, Wnt signaling pathway, and spermatogenesis. A recent pan-cancer study revealed that the androgen receptor (AR) is overexpressed in GBM[42]. In vitro experiments using anti-androgen drugs resulted in the inhibition of GBM cell proliferation[43, 44]. Spermatogenesis, a process regulated by androgens and highly active in the male reproductive system, showed significant enrichment in both datasets, suggesting that male patients may have unique susceptibilities or therapeutic responses in certain molecular mechanisms. Research has demonstrated that in high-grade gliomas, there is an enrichment of MYC_TARGETS_V1, G2M checkpoint, and E2F target hallmarks[45]. It is worth mentioning that these five pathways are significant in transcriptional regulation and are also involved in cell cycle regulation. Cancer cells utilize cell cycle checkpoints to delay mitosis and repair DNA damage. In particular, the G2/M phase of the cell cycle carried great weight in DNA repair[46]. The E2F family directly regulates the transcription of genes involved in DNA replication and cell cycle progression[47], further highlighting its transcriptional regulatory functions.

Fig. 2. Depicting the immune "cold" and "hot" status of GBM. A The consensus score matrix of GBM samples when k=2. B The CDF curves of consensus matrix for each k (showed by colors). C Heatmap shows the infiltration abundance of 8 immune cell types evaluated by ssGSEA for two

clusters. The distribution of patient categories, gender, age, and survival status can be seen at the top of the heatmap. D The violin plot shows distribution of 8 immune cell types infiltration between two clusters. The red color represents the cluster of immune “cold” tumor and blue represents the cluster of immune “hot” tumor. The significance of differences between the two clusters is indicated by “***”. E The boxplot shows the distribution of immune score inferred by ESTIMATE algorithm between two clusters. F Differential expression analysis of immune “cold” tumors. The size and color gamut of the dots are determined by the -log10(P.value) values. We set |log2FC|≥0.5 and pvalue≤0.05 as the thresholds for screening DEGs. G GSEA analysis of immune “cold” tumors. The size and color of the dots are determined by the NES (Normalized Enrichment Score) values. The padj_score is derived by -log10(padj).

Result 3 Establishment and validation of hallmark-related prognostic signatures in glioblastoma

To establish a GBM risk prediction model, we used a random combination of 11 machine learning algorithms[24] and integrated the previously analyzed hallmarks based on bulk-level GBM transcritional data with survival information. We adopted a strategy of selecting candidate features from the union of intersections taken pairwise from three gene sets. These gene sets consisted of the hallmark genes related to single-cell tumor cells, bulk immune “cold” tumor samples, and the up-regulated DEGs of bulk tumor samples (Figure 3A). In total, 352 immune-related tumor hallmark genes at the combined single-cell and bulk levels were obtained. These features will first be filtered using univariate Cox regression and then we calculated the C-index of each model in the training set and the validation set (Figure 3B). The optimal model was a

combination of StepCox(direction=forward) and RSF, which screened and identified 42 feature genes. This combination model had the highest C-index in validation datasets (Supplementary Figure 2A). Based on the results of the optimal model, we divided the patients into high-risk and low-risk groups. Survival analysis indicated that our filtered prognostic-related hallmark signatures (HMsig) could effectively distinguish patients with different risk levels (Figure 3C, Supplementary Figure 2B). What's more, the area under the curve (AUC) values for 1-year, 3-year, and 5-year survival in both the training set and the validation set were all greater than 0.85. Meta-analysis of the two datasets suggested that the score calculated by the StepCox(forward) and RSF model was a risk factor for GBM (Figure 3D, Supplementary Figure 3E-F). By comparing our obtained optimal model with GBM risk signature models constructed from different literature and calculating the C-index and AUC values, our optimized model ranked first (Figure 3F-I). All of these demonstrate that HMsig possess excellent predictive capabilities.

42 features were screened by univariate Cox regression and Kaplan-Meier (KM) survival analysis, and ultimately 7 features were obtained. ASF1A, AEBP1, DCC, HDAC5, IL13RA2, OPHN1 and PRPS1, were identified by the optimal model and show prognostic relevance in GBM. We then tallied the frequency with which they were selected as core features (Figure 3E, Supplementary Figure 3C). Currently, some studies have found that ASF1A was identified as a biomarker for malignant diseases such as lung adenocarcinoma and hepatocellular carcinoma[48-50]. However, no studies have yet reported that ASF1A and OPHN1 can be a risk gene for GBM. ASF1A regulates the expression of cell cycle-related genes and influences glial cell differentiation[51].

OPHN1 not only participates in cell cycle regulation[52, 53] but has also been found to promote tumor progression when overexpressed[54]. To further elucidate the impact of these seven genes on patient survival prediction, we employed the SHAP algorithm to calculate SHAP values based on risk scores derived from the StepCox (forward) and RSF combined model (Supplementary Figure 3D). The analysis revealed that genes AEBP1, ASF1A, and PRPS1 ranked highest by SHAP values, indicating their substantial influence on survival prediction outcomes. We imported several additional validation sets from GEO datasets. These 7 genes were also able to significantly distinguish the high-risk and low-risk patients in the GEO datasets, demonstrating good validation efficiency and strong generalization ability (Figure 3J-L).

Fig. 3. Identifcation of potential GBM features in GBM. A Overview of features selection workflow for patient tumors. The upper part shows the feature selection strategy at the single-cell level; the lower part shows the feature selection strategy at the bulk level; the volcano plot on the far right represents the differential analysis between tumor and normal samples at the bulk level. The drawing of tissues and equipments was completed by BioRender.com. B Combined machine learning model framework of Mime algorithm. The combination highlighted in red represents our optimal model. C Kaplan–Meier curves of OS according to the StepCox(forward)+RSF in test set. D Time-dependent ROC analysis for predicting OS at 1, 3, and 5 years. The specific ROC values are labeled in the figure. E Selection frequency of HMsig in various machine learning algorithms and their enriched hallmarks. The size of the dots corresponds to the frequency at which the features are selected. F-I C-index and ROC analysis comparing our optimized model with published GBM signatures in the TCGA-CGGA cohort. The dashed line corresponding to the red scale represents

the C-index/AUC value of our optimized model. J-L Kaplan–Meier curves of OS based on the StepCox(forward)+RSF model in an additional GEO validation set.

Result 4 Revealing immune regulatory mechanisms in TME through cell communication analysis

The TME is not merely an aggregation of cancer cells but rather a complex ecosystem composed of multiple cell types. These cells interact with one another via the secretion of factors, cytokines, and cell-to-cell contacts, jointly affecting the growth, invasion, and metastasis of tumors. For the purpose of deeply profiling the TME of GBM, the CellChat algorithm was applied to examine the communication between tumor cells and T cells, B cells, and myeloid cells. CD8_T_EM and CD8_T_EX cells engaged in intense interactions with tumor cells, myeloid cells, and other T cells (Figure 4A). Apparently, the two types of tumor cells have the strongest interaction with immune cells in macrophage migration inhibitory factor (MIF) signaling pathway, followed by pleiotrophin (PTN) signaling pathway. CD8_T_EX cells account for a relatively large proportion in the communication intensity with the two types of tumor cells in the MIF, major histocompatibility complex class I (MHC-I), and PTN signaling pathways (Figure 4B).

Thereafter, we conducted a more detailed examination of the expression of LR pairs. MIF−(CD74+CXCR4) mediated firm interactions between tumor cells and T cells (Figure 4C-D). In GBM, this interaction may lead to the suppression of immune cell activity. Tumor cells can inhibit macrophage activity through the MIF-(CD74-CXCR4)[55]. The interaction of CD99-CD99 was also robust between immune cells and

tumor cells (Figure 4C). Upon T cell activation, the engagement of CD99 and its ligand can upregulate the expression of IL-6 and TNF-α, which in turn promotes tumor cell proliferation and survival[56]. The interaction mediated by SPP1-CD44 between myeloid cells and other immune cells was remarkably strong (Figure 4E-F). SPP1-CD44 inhibits T cell infiltration, reducing the number of T cells in tumor tissues and exacerbating tumor immune evasion[57]. Other LR pairs, such as KLRB1-CLEC2D, although expressed at relatively low levels in certain cell types, are also essential to the TME. The interaction between the immune checkpoint molecule KLRB1 and CLEC2D is of great significance for tumor progression and immune evasion[58]. Previous studies have demonstrated that the interaction of KLRB1-CLEC2D can mediate immunosuppression and potentiate the development of GBM[59].

These findings implied that MIF, SPP1 and CD99 are indispensable for the TME. Therefore, we constructed hierarchical clustering maps for these three genes to illustrate the communication intensity between cell clusters and identify the key cell clusters involved in cellular communication. In line with our previous LR analysis, tumor cells and immune cells exhibited strong communication via the MIF signaling pathway, which was distinct from the SPP1 and CD99 signaling pathways (Figure 4G, J). This may be attributed to the expression of MIF receptors on various immune cells, such as CD74 on the surface of macrophages and dendritic cells. Additionally, the binding of MIF to its receptors activates multiple downstream signaling pathways, including ERK1/2, AMPK, and AKT, following MIF-receptor binding, thereby strengthening their interactions[60]. Within the SPP1 signaling pathway, the communication between

macrophages, dendritic cells, and T cells was particularly intense (Figure 4I). SPP1 might enhance the interaction between myeloid cells and T cells, thereby suppressing immune surveillance. Substantial intercellular signaling communication occurred between tumor cells and immune cells in the MIF, SPP1, and CD99 signaling pathways. Among tumor cells and T cells, the direct or indirect interaction became the main factor contributing to the immunosuppressive microenvironment in GBM[55, 61].

Since we did not clearly discern the interaction between the HMsig and LR pairs, we then constructed a protein-protein interaction (PPI) network to explore the associations between them (Figure 4K). It was found that EFNB1 had a ppi with OPHN1. In GBM, the high expression of EFNB1 was related to poor prognosis for patients and could potentially serve as a prognostic marker and therapeutic target[62]. The high expression of LR genes having interaction relationships with HMsig will lead to a poorer prognosis for patients (Supplementary Figure 3A).

Fig. 4. CellChat analysis of the communications between tumor cells and immune cells. Global communications are presented by circle plots showing the number of significant LR pairs in 10 cell clusters. B Chord diagram shows the communication strength at the gene signaling pathway level. C-F, H Significant LR pairs between tumor and immune cells, within myeloid cells, T cells and between myeloid and other immune cells. Dot color reflects communication probabilities, and dot size represents computed p-values (one-sided permutation test). Empty space indicates zero communication probability. G, I-J Three representative signaling pathways, MIF, SPP1, and CD99 pathways, were further analyzed. The inferred networks of communication between all cell types are displayed using hierarchical plots. K Protein-Protein Interaction (PPI) network of HMsig and

LR pairs. Orange dots represent HMsig, blue dots represent ligand-receptor pairs, and the color and thickness of the lines are determined by the combined score.

Result 5 Elevated expression of immune checkpoint genes during T cell differentiation

T cells show diverse differentiation trajectories as a result of the marked impact of the TME[63]. We established a 3D developmental trajectory for T cells. From this analysis, two common trajectories emerged. One trajectory commenced with naive T cells, passed through Treg cells, and ended at CD8_T_EX cells; the other started from naive T cells, proceeded through CD8_T_EM cells, and terminated at CD8_T_EX cells (Figure 5A, Supplementary Figure 3B). Meanwhile, we mapped the 2D trajectory for a more detailed and lucid depiction (Figure 5B-C). We used the R package clusterProfiler to conduct Gene Ontology (GO) functional enrichment analysis on genes related to T-lineage differentiation. This allowed us to explore the impact of these genes on cellular physiological functions (Figure 5D, Supplementary Table 3). Genes associated with the initial T cell differentiation stage, including CD4, CXCR4, CD69, and KLRB1, display high expression levels in Cluster1 and Cluster2. The up-regulation of CXCR4 may be related to the enhanced migration ability of T cells and contribute to the aggregation of T cells[64]. Serving as T cell activation markers, CD69 and KLRB1 are markedly upregulated in activated CD4+ T cells[65, 66]. GO enrichment analysis reveals that Cluster 1 and 2 are enriched in functional pathways like positive regulation of the inflammatory response and cytokine production. Notably, genes expressed by Tregs cells, such as TIGIT, FOXP3, and IL2RA, show high expression in Cluster3. Cells

clustered into Cluster3 possess functions such as regulation of T cell activation and positive regulation of leukocyte activation. These cells are crucial in immune response and cytotoxic killing. At the terminal stage of T cell differentiation, Cluster 5 characterized by high expression of CCL5 and LAG3 exhibited enrichment of functional pathways related to T cell exhaustion, including intercellular adhesion regulation and inhibiting immune cell activation.

At the same time, we monitored the expression changes of key genes that drive cellular development or state transitions during the pseudotime. CCL5 is a pivotal chemokine determining whether tumors will be infiltrated by T cells. As the T cell pseudotime trajectory progresses, the expression of CCL5 gradually increases (Figure 5E). While this is beneficial for recruiting T cells to infiltrate the tumor, it also serves as a marker of the malignant transformation process in GBM[67]. FOS, functioning as a TF, is involved in regulating various pathophysiological processes of cells. During T cell activation, the product of the FOS gene participated in modulating the expression of cytokine genes[68]. With the development of the T cell, the expression of FOS gradually decreased, which can affect the recruitment and activation state of T cells (Figure 5F). Besides, the analysis showed that the genes in Cluster 3 were highly expressed in the early stage of the trajectory and then gradually declined. This suggests that these genes might play an important role in the early stage but were rapidly suppressed or shut down, like FOXP3 and IL2RA expressed by Treg cells (Supplementary Figure 3C). However, Cluster 5 presented an opposite pattern (Figure 5D). For instance, the immune checkpoint molecules LAG3 was highly expressed at

the terminal stage, corresponding to the temporal distribution of CD8_T_EX. This displays that the high expression of immune checkpoint molecules would lead to the exhaustion state of T cells. Unexpectedly, we found that the LR pairs on the T cell surface were also key genes driving T-lineage evolution. Take HLA-DRA-CD4 for example, it promotes T cell activation and differentiation[69].

The co-expression of multiple co-inhibitory receptors is a crucial marker of T cell dysfunction. Building upon the previous analysis, the results demonstrated that LAG3 was highly expressed at the end stage of T cell differentiation. Thus, we investigated the gene expression of all immunosuppressive checkpoint molecules. We found that PD1 (PDCD1), LAG3 (LAG3), TIM3 (HAVCR2), and other two genes were highly expressed during the T-progression. The high expression of these three ICGs in CD8_T_EX cells is associated with the functional decline of CD8_T_EM cells, potentially facilitating their conversion into CD8_T_EX cells (Figure 5G-I, Supplementary Figure 3D-E).

Fig. 5 scRNA-seq profiles of dynamic changes in T cells. A-B Pseudotime analysis exploring the cellular trajectory of T cells with high variable genes. The lines correspond to the principal graph learned by Monocle3. Each dot in the trajectories represents one cell, which is color coded according to their cell types. It depicts the temporal distribution of T cells in both three-dimensional space (A, Supplementary Fig. 5A) and a planar state (B). C Pseudotime ordering of cells starting in the Naive_T cell cluster. D The cell distribution of each T cell cluster along with the pseudotime (upper panel), color-coded by T cell clusters. Heatmap showing dynamic expression changes of genes in T cells (lower panel). The GO enrichment terms for each cluster are displayed on the right of the

heatmap. E-F Gene expression dynamics along the trajectory, including CCL5, FOS and the others (Supplementary Fig. 5B-H). The color of dots coded according to their cell types and the line represents the gene expression at each time point. G-I Boxplot showed the expression of immune checkpoint molecules (HAVCR2, PDCD1, LAG3) in T cells. The color of dots coded according to their cell types. The significance of differences between each pair of cell clusters is marked by "*", with "ns" indicating no significant difference.

Result 6 Disclosing synergistic TFs regulation in tumor cells and T cells for prognosis

TFs, as upstream regulatory elements, can directly influence the behaviors of tumor cells and the activities of immune cells. We carried out SCENIC analysis on two categories of tumor cells, as well as on two types of CD8 T cells with strong interactions, aiming to identify the TF responsible for driving HMsig and immune checkpoint molecules. The cell-specific TFs, ETS1 and RUNX3 were in an active state in CD8_T_EM and CD8_T_EX cells; SOX2 and EPAS1 were in an active state in tumor cells (Figure 6A, Supplementary Figure 4A). Among them, ETS1 was involved in angiogenesis, which enhances the invasiveness and metastatic potential of tumor cells[70]. SOX2, on the other hand, promotes tumor cell proliferation by regulating genes associated with cell cycle progression[71]. All these suggest that TFs also participated in the regulation of the TME. Afterward, we constructed gene regulatory networks (GRNs) for CD8_T_EX, CD8_T_EM, and tumor cells to map specific TFs-gene interactions. (Figure 6B, Supplementary Figure 4L-M). The regulatory network centered around TFs like ETS1 and RUNX3, is essential for the occurrence, development and functional

activation of multiple immune cells[72-75]. In light of the HMsig we uncovered, we probed for their corresponding driving TFs and obtain four of them, namely SOX11, SOX4, CEBPD, and EGR1. We then created a schematic diagram of the TF-HMsig/ICG regulatory mechanism (Figure 6C, D).

Understanding the cooperative interaction among TFs and its impact on GBM is crucial as it significantly affects GBM prognosis. Hence, we scrutinized the correlations among TFs at the bulk transcriptome level and found that the TFs were concordant in expression and significantly associated (Figure 6E, Supplementary Figure 4B). Additionally, the expressions of the TFs in two cell types at the single-cell level were consistent as well (Supplementary Figure 4C-K). We discovered that RUNX3 and CEBPD showed similar expression patterns. These two TFs also significantly differentiate patients in the high-risk and low-risk groups within the TCGA-CGGA datasets. This shows that the synergistic action of TFs with different regulatory modes can influence patient prognosis (Figure 6F-G).

When cells are confronted with various environmental stresses, such as oxidative stress and nutrient deficiency, the cooperative action of TFs can regulate the gene expression within the cells, assisting the cells in adapting to environmental changes and maintaining cell survival and function[76]. During the process of cell differentiation, the cooperative action of TFs is also a key determinant of cell fate[77]. With the aim to explore the regulatory mechanisms of TFs with cooperative effects among cells, we performed functional enrichment analysis on the GRNs and searched for the motifs of TFs. In tumor cells, the sub networks regulated by SOX4 and SOX11 were enriched in

pathways related to developmental cell growth, neuron migration, and dendrite morphogenesis, among others. Meanwhile, in T cells, the sub-networks regulated by ELF1 and RUNX3 were enriched in functional pathways associated with regulation of T cell activation, lymphocyte mediated immunity, and lymphocyte proliferation. Although the specific regulatory mechanisms of TFs diverge, TFs in tumor cells and T cells play vital roles in different cellular functions. TFs in tumor cells impact cell development, and those in T cells coordinate the immune response. These findings show a functional similarity between the two clusters of TFs. They are both involved in key cellular processes essential for the functions and survival of their respective cell types (Figure 6H).

Subsequently, we identified the motifs of TFs associated with HMsig and ICGs, as their expression levels hinge predominantly on specific motifs in their regulatory regions and the binding of corresponding TFs. We found that RUNX3 might simultaneously regulate the expressions of two immune checkpoint molecules, LAG3 and PDCD1. Hence, RUNX3 may coordinate the expression of two immune checkpoint molecules by binding to the same motif, thereby establishing a "dual-brake" mechanism that more efficiently suppresses T cell activity. In the TME, this mechanism could be utilized by tumor cells to promote immune escape through upregulation of RUNX3 and simultaneous activation of PDCD1 and LAG3. Moreover, we discovered that the RUNX3 binding motif is highly conserved between humans and mice through extended motif analysis. Such conservation allows TFs to maintain stable regulation of gene expression and ensure proper biological function. RUNX3 has been shown to play a

critical role in T cell differentiation and function, and its dysregulation is associated with impaired immune responses and cancer progression[78]. Additionally, the conservation of TF binding motifs, such as those of RUNX3, is essential for maintaining immune homeostasis and preventing pathological conditions like autoimmune diseases and cancer[79]. The concordance in the preferred binding motif sequences of these two genes is clearly of vital importance for precisely and thoroughly elucidating the immune escape mechanisms.

Fig. 6 Regulatory roles of TFs between tumor cells and T cells. A Heatmap shows the binary activity of specific TFs. Specific TFs are chosen through RSS regulon specificity score (Supplementary Fig6A). B Transcription regulatory network constructed by CD8_T_EX specific TFs and its target genes. Warm-toned dots represent TFs, Cold-toned dots represent targets gene. The color of the lines represents the number of motifs shared between the TFs and their target genes. C-D Schematic diagram of TF-HMsig/ICG regulatory mechanism. Orange circles represent TFs driving HMsig in tumor cells, yellow circles represent TFs driving immune checkpoint molecules in T cells, and blue and green hexagons represent the identified HMsig and ICGs. E-F Correlation analysis of TFs in TCGA-CGGA cohort, including SOX4-SOX11 (E), RUNX3-CEBPD (F) and other TF pairs (Supplementary Fig. 6). The correlation coefficient and significance markers are indicated in the lower right corner of the figure. G Survival analysis of RUNX3-CEBPD in TCGA-CGGA cohort. The yellow and blue line respectively indicate the high and low gene expression of RUNX3-CEBPD in TCGA-CGGA cohort. H TF co-regulation mechanisms in two cell types. On the left are the GO terms enriched by the TF regulatory sub networks, while on the right are the TF regulatory sub networks with synergistic interactions, along with the motifs corresponding to the

HMsig/ICG regulated by these TFs. The synergistic mechanisms in tumor cells are shown in the upper section, with the regulatory network highlighted in orange. The synergistic mechanisms in T cells are also displayed in the upper section, with the regulatory network highlighted in yellow.

Result7 Spatial transcriptomics profiling of molecular heterogeneity and immunosuppressive microenvironment in glioblastoma

Spatial transcriptomic techniques allow high-resolution in situ measurement of gene expression, revealing expression gradients and identifying spatially organized cellular domains. To delineate the molecular and cellular architecture of GBM across anatomical regions, we collected 12 IDH-wt GBM spatial transcriptome samples (Figure 7A), including tumors from the right temporal lobe, left frontal lobe and other regions (Supplementary Table 1). To enable higher-resolution analysis at the cellular level, the CARD deconvolution algorithm was applied to map scRNA-seq-defined cell types onto spatial transcriptomic data, covering glia and neuronal cells, oligodendrocytes, epithelial cells, pericytes, myeloid cells, T cells, and B cells (Figure 7B). The tumor region's delineation aligns with prior studies[22, 23], with regions predominantly mapped to glia and neuronal cells and oligodendrocytes, validated by the expression of marker gene (Figure 7C).

The spatial distribution map of cells revealed interpatient heterogeneity. While GBM5_1 and zh881t1 both originated from the right temporal lobe, the tumor in zh881t1 nearly occupied the entire section, highlighting divergent tumor expansion patterns. Similarly, extensive tumor cell distribution was observed in left temporal lobe sections (Supplementary Figure5). Notably, 12 samples showed low abundance of T

cell, indicating GBM's inherent tendency to present as an immunologically “cold” tumor type (Figure 7A, Supplementary Figure5). GBM5_1 exhibited extensive pericyte infiltration, consistent with the brain's high vascularity and GBM's angiotropic behavior. Pericytes, as key components of the neurovascular unit (NVU), may thus foster a microenvironment for tumor progression[80].

We further examined spatial expression patterns of HMsig, ICGs and TFs mediating regulatory interactions. HMsig such as IL13RA2, DCC, AEBP1 and OPHN1 showed high expression in tumor cells (Figure 7D). In patient GBM5_1, the delineated tumor region was identified in study[22] as a hypoxic and invasive zone and the remaining areas were divided into vascular enrichment areas. Additionally, the tumor tissue of patient GBM2 is located in the corpus callosum, and analysis indicates that the tumor area is primarily situated at the tissue margin, which may suggest an invasive tendency of the tumor along the corpus callosum-cortical junction area or perivascular spaces[81] (Supplementary Figure5). The high expression of HMsig in this area suggests that it may directly reflect hypoxia stress intensity, spatially colocalizing with tumor invasion or vascular-sparse regions. T cells exhibited enrichment of immune checkpoint molecules, suggesting that there is a state of T cell exhaustion in the TME.

Furthermore, the high expression of cell type-specific TFs suggests their spatially coordinated co-expression patterns (Figure 7D-E). The tumor-associated TFs SOX4 and SOX11 demonstrate spatially co-regulated expression in diverse samples, underscoring their critical role in modulating HMsig. Meanwhile, T cell-related TF are also markedly expressed in localized tumor areas, indicating a synergistic interplay

between tumor cells and T cells in specific spatial regions. This interaction may contribute to the formation of an immunosuppressive TME. These findings are consistent with cellular distribution patterns and supported by bulk and single-cell transcriptome analyses, reinforcing the immunosuppressive nature of the TME.

Fig. 7. The spatial expression patterns of HMsig, ICG and TFs. A H&E micrographs of patient GBM5_1, mgh258, zh881t1, zh8811b, zh1007nec and zh1019t1 reveal the histological architecture of the tumor slices. B Description of ST data using cell type deconvolution. The color of each spot represents the proportion of each cell type in all spots, with the color intensity indicating the relative abundance. C-E The expression levels of cell marker, HMsig, ICG and TFs in representive samples. Dot colored by gene expression levels.

Discussion

This study characterized the TME of GBM by clustering cells from tumor tissues, revealing the existence of individual differences within the microenvironment. Our research demonstrated that hallmarks enriched at both the single-cell and bulk levels are pivotal to cell cycle regulation and profoundly influence the transcriptional governance of GBM. This suggests that investigating the regulation of cell cycle related genes and TFs is essential for understanding the uncontrolled proliferation of GBM cells. Although numerous studies have explored the GBM microenvironment across both data modalities, no study has yet considered the hallmark features of malignant cells and the immune characteristics of immune “cold” and “hot” tumor samples. Due to the heterogeneity of GBM tissue and the existence of the BBB, there are significant

differences in GBM patients' TME in terms of the degree of immune cell infiltration, immune activity, and responsiveness to immunotherapy. This is also a challenge we need to face currently. We can more vividly reproduce the true state of the patient's disease by identifying the characteristics of tumor malignant cells. Dividing GBM samples into immune “cold” and immune “hot” states according to immune characteristics can more accurately capture the immune related features of GBM, and further deeply analyze and clearly present its immunosuppressive microenvironment. We obtained features from the hallmarks of single-cell tumor cells, the hallmarks of bulk immune “cold” samples, and the upregulated differentially expressed genes in bulk tumor cells, with the aim of achieving a more nuanced molecular profile of GBM across multiple scales. We identified HMsig and optimized model by using combined machine learning methods. After multilayer verification, our optimized model has good predictive and validation efficiency.

In an effort to gain a more in-depth characterization and regulatory mechanisms of TME, we undertook cell communication and cell trajectory analysis. Cell communication analysis revealed that direct or indirect interactions between tumor cells and T cells are the primary cause of the immunosuppressive microenvironment in GBM. These interactions impair immune function, disrupting the TME balance. Cell trajectory analysis indicated that the upregulation of immune checkpoint molecules leads to a progressive decline in T cell function. This functional impairment enables tumor cells to escape immune surveillance, a key factor contributing to tumor growth and metastasis. TFs, as upstream regulatory elements, directly influence the behavior of

tumor cells and the activity of immune cells. Through SCENIC analysis, RUNX3 was found to simultaneously regulate the expression of LAG3 and PDCD1. This dual regulation inhibits the immune response of T cells within the TME, further highlighting the complex role of TFs in modulating the immune landscape. Our analysis also showed that TFs exhibit synergistic effects not only within cell clusters but also between different cell clusters. This regulatory interplay significantly impacts patient prognosis. Consistent with the studies by Timothy F et al[82, 83], we found that PDCD1 and LAG3 were significantly upregulated in glioblastoma multiforme (GBM). However, our study further revealed the mechanism by which RUNX3 simultaneously regulates these two genes through a shared motif, and this finding has not been reported previously. In the future, research on primary GBM could focus on using CRISPR-Cas9 genome editing technologies to verify the synergistic effect of RUNX3 and CEBPD in the process of immune escape, so as to better understand the dynamic immune evasion strategies in GBM.

In summary, this study revealed diverse immune escape mechanisms in GBM, including the interaction of key LR pairs between cell clusters, the upregulation of immune checkpoint molecules, and the synergistic regulation of RUNX3-CEBPD. These findings displayed the complexity of the TME and the refractory nature of GBM. Although this study identified only seven risk genes, five immune checkpoint molecules and a pair of synergistic TFs across cell clusters that affect patient prognosis, this may be due to the high heterogeneity and the limited representativeness of the tumor samples. Nonetheless, these results still offer fresh viewpoints and possible

biomarkers that can enhance our comprehension of the proliferation and prognosis of GBM. Collectively, these results not only enhance our understanding of the complexity of the TME but also provide important theoretical foundations and new research directions for the diagnosis and treatment of GBM.

Data availability

The authors declare no competing interests. All datasets analyzed in this study are publicly available from established repositories and can be accessed using the accession numbers listed below.

Single-cell RNA sequencing (scRNA-seq) data for glioblastoma (GBM) were obtained from the Gene Expression Omnibus (GEO) under accession GSE182109, comprising nine primary IDH-wildtype GBM samples. These data were used for the characterization of tumor microenvironmental cell states. Bulk transcriptomic data of primary GBM with corresponding clinical and survival information (n = 769) were collected from publicly available resources, including GEO, The Cancer Genome Atlas (TCGA; accessed through the UCSC Xena platform), and the Chinese Glioma Genome Atlas (CGGA). Normal brain transcriptomic profiles (n = 105) were retrieved from the Genotype-Tissue Expression (GTEx) project and used as non-tumor reference samples. For independent validation of prognostic markers, additional microarray-based gene expression datasets with survival annotations were obtained from GEO, including GSE7696, GSE42669, GSE16011, and GSE108474. Spatial transcriptomics data for primary IDH-wildtype GBM were also obtained from GEO under accessions

GSE194329 and GSE237183, comprising a total of twelve samples used for spatial analyses.

Data availability

All code used in this study is available at https://github.com/travilucas/GBM under the MIT License. The repository contains all scripts and workflows to reproduce the analyses.

Funding sources

This work was supported by grants from the National Science and Technology Major Program [2024ZD0530500]; and National Natural Science Foundation of China [62472131, 62502128], and the Key Research and Development Program of Heilongjiang Province [2024ZX12C27], and the China Postdoctoral Science Foundation [2024M760709], and Heilongjiang Postdoctoral Fund [LBH-Z24210], and Longjiang New Era Outstanding Doctoral Dissertation Project Grant [LJYXL2024-069], and Harbin Medical University Fund [31031250051].

Competing interests

The authors declare no competing interests.

CRediT authorship contribution statement

Tengyue Li: Writing–review & editing, Validation, Methodology, Conceptualization. **Wanqi Mi:** Validation, Methodology, Investigation. **Huarui Yan:** Writing–original draft, Methodology, Investigation. **Yining Ma:** Validation;

Investigation. **Han Jiang:** Visualization, Investigation. **Xiaoxu Yang:** Visualization; Investigation. **Yunpeng Zhang:** Conceptualization. **Congxue Hu:** Conceptualization.

References

1. Yeo, A.T., et al., *Single-cell RNA sequencing reveals evolution of immune landscape during glioblastoma progression.* Nature Immunology, 2022. **23**(6): p. 971-+.
2. Caniglia, J.L., et al., *Beyond glucose: alternative sources of energy in glioblastoma.* Theranostics, 2021. **11**(5): p. 2048-2057.
3. Wang, R., et al., *Single-cell RNA sequencing reveals changes in glioma-associated macrophage polarization and cellular states of malignant gliomas with high AQP4 expression.* Cancer Gene Therapy, 2023. **30**(5): p. 716-726.
4. Wolf, A., S. Agnihotri, and A. Guha, *Erratum: Targeting Metabolic Remodeling in Glioblastoma Multiforme.* Oncotarget, 2018. **9**(78): p. 34855.
5. Hambardzumyan, D., D.H. Gutmann, and H. Kettenmann, *The role of microglia and macrophages in glioma maintenance and progression.* Nature Neuroscience, 2016. **19**(1): p. 20-27.
6. Verdugo, E., I. Puerto, and M.A. Medina, *An update on the molecular biology of glioblastoma, with clinical implications and progress in its treatment.* Cancer Communications, 2022. **42**(11): p. 1083-1111.
7. Daisy Precilla, S., et al., *Crosstalk between PI3K/AKT/mTOR and WNT/β-Catenin signaling in GBM - Could combination therapy checkmate the collusion?* Cell Signal, 2022. **95**: p. 110350.
8. Yuan, Y.M., et al., *Wnt signaling: Modulating tumor-associated macrophages and related immunotherapeutic insights.* Biochemical Pharmacology, 2024. **223**.
9. Torrisi, F., et al., *The Hallmarks of Glioblastoma: Heterogeneity, Intercellular Crosstalk and Molecular Signature of Invasiveness and Progression.* Biomedicines, 2022. **10**(4).

10. Carvajal, L.A., et al., *E2F7, a novel target, is up-regulated by p53 and mediates DNA damage-dependent transcriptional repression.* Genes & Development, 2012. **26**(14): p. 1533-1545.

11. Lambert, S.A., et al., *The Human Transcription Factors.* Cell, 2018. **172**(4): p. 650-665.

12. Wang, Q.X., et al., *Comprehensive Analysis of the E2F Transcription Factor Family in Human Lung Adenocarcinoma.* International Journal of General Medicine, 2022. **15**: p. 5973-5984.

13. Liu, X.S., et al., *Comprehensive Analysis of Prognostic and Immune Infiltrates for E2F Transcription Factors in Human Pancreatic Adenocarcinoma.* Frontiers in Oncology, 2021. **10**.

14. Papavassiliou, K.A. and A.G. Papavassiliou, *Transcription factors in glioblastoma - Molecular pathogenesis and clinical implications.* Biochimica Et Biophysica Acta-Reviews on Cancer, 2022. **1877**(1).

15. Ma, T.J., et al., *Reprogramming Transcription Factors Oct4 and Sox2 Induce a BRD-Dependent Immunosuppressive Transcriptome in GBM-Propagating Cells.* Cancer Research, 2021. **81**(9): p. 2457-2469.

16. Myers, B.L., et al., *Transcription factors ASCL1 and OLIG2 drive glioblastoma initiation and co-regulate tumor cell types and migration.* Nature Communications, 2024. **15**(1).

17. Abdelfattah, N., et al., *Single-cell analysis of human glioma and immune cells identifies S100A4 as an immunotherapy target.* Nat Commun, 2022. **13**(1): p. 767.

18. Murat, A., et al., *Stem cell-related "self-renewal" signature and high epidermal growth factor receptor expression associated with resistance to concomitant chemoradiotherapy in glioblastoma.* J Clin Oncol, 2008. **26**(18): p. 3015-24.

19. Joo, K.M., et al., *Patient-specific orthotopic glioblastoma xenograft models recapitulate the histopathology and biology of human glioblastomas in situ.* Cell Rep, 2013. **3**(1): p. 260-73.

20. Gravendeel, L.A., et al., *Intrinsic gene expression profiles of gliomas are a better predictor of survival than histology.* Cancer Res, 2009. **69**(23): p. 9065-72.

21. Gusev, Y., et al., *The REMBRANDT study, a large collection of genomic data from brain cancer patients.* Sci Data, 2018. **5**: p. 180158.

22. Ren, Y., et al., *Spatial transcriptomics reveals niche-specific enrichment and vulnerabilities of radial glial stem-like cells in malignant gliomas.* Nat Commun, 2023. **14**(1): p. 1028.

23. Greenwald, A.C., et al., *Integrative spatial analysis reveals a multi-layered organization of glioblastoma.* Cell, 2024. **187**(10): p. 2485-2501.e26.

24. Liu, Z., et al., *Machine learning-based integration develops an immune-derived lncRNA signature for improving outcomes in colorectal cancer.* Nat Commun, 2022. **13**(1): p. 816.

25. Jin, S., et al., *Inference and analysis of cell-cell communication using CellChat.* Nat Commun, 2021. **12**(1): p. 1088.

26. Cao, J., et al., *The single-cell transcriptional landscape of mammalian organogenesis.* Nature, 2019. **566**(7745): p. 496-502.

27. Aibar, S., et al., *SCENIC: single-cell regulatory network inference and clustering.* Nat Methods, 2017. **14**(11): p. 1083-1086.

28. Ma, Y. and X. Zhou, *Spatially informed cell-type deconvolution for spatial transcriptomics.* Nat Biotechnol, 2022. **40**(9): p. 1349-1359.

29. Wang, Z.X., et al., *SVIP reduces IGFBP-2 expression and inhibits glioblastoma progression via stabilizing PTEN.* Cell Death Discovery, 2024. **10**(1).

30. Kim, J.A., et al., *VBP1 represses cancer metastasis by enhancing HIF-1α degradation induced by pVHL.* Febs Journal, 2018. **285**(1): p. 115-126.

31. Wang, S.H., et al., *Mechanism of Notch Signaling Pathway in Malignant Progression of Glioblastoma and Targeted Therapy.* Biomolecules, 2024. **14**(4).

32. Lin, H., et al., *Understanding the immunosuppressive microenvironment of glioma: mechanistic insights and clinical perspectives.* J Hematol Oncol, 2024. **17**(1): p. 31.

33. Fridman, W.H., et al., *The immune contexture in human tumours: impact on clinical outcome.* Nat Rev Cancer, 2012. **12**(4): p. 298-306.

34. Malan, A.F., *The need for regional planning of perinatal care.* S Afr Med J, 1975. **49**(33): p. 1363-4.

35. Di Carlo, E., G. Forni, and P. Musiani, *Neutrophils in the antitumoral immune response.* Chem Immunol Allergy, 2003. **83**: p. 182-203.

36. Nathan, C., *Neutrophils and immunity: challenges and opportunities.* Nat Rev Immunol, 2006. **6**(3): p. 173-82.

37. Hampton, M.B., A.J. Kettle, and C.C. Winterbourn, *Inside the neutrophil phagosome: oxidants, myeloperoxidase, and bacterial killing.* Blood, 1998. **92**(9): p. 3007-17.

38. Pham, C.T., *Neutrophil serine proteases: specific regulators of inflammation.* Nat Rev Immunol, 2006. **6**(7): p. 541-50.

39. Lysiak, M., et al., *Deletions on Chromosome Y and Downregulation of the <i>SRY</i> Gene in Tumor Tissue Are Associated with Worse Survival of Glioblastoma Patients.* Cancers, 2021. **13**(7).

40. Helmink, B.A., et al., *B cells and tertiary lymphoid structures promote immunotherapy response.* Nature, 2020. **577**(7791): p. 549-+.

41. Fortelny, N., et al., *JAK-STAT signaling maintains homeostasis in T cells and macrophages.*

Nature Immunology, 2024.

42. Hu, C., et al., *The androgen receptor expression and association with patient's survival in different cancers.* Genomics, 2020. **112**(2): p. 1926-1940.

43. Werner, C.K., et al., *Expression of the Androgen Receptor Governs Radiation Resistance in a Subset of Glioblastomas Vulnerable to Antiandrogen Therapy.* Mol Cancer Ther, 2020. **19**(10): p. 2163-2174.

44. Zhao, N., et al., *Androgen Receptor, Although Not a Specific Marker For, Is a Novel Target to Suppress Glioma Stem Cells as a Therapeutic Strategy for Glioblastoma.* Front Oncol, 2021. **11**: p. 616625.

45. Ghantasala, S., et al., *High-Grade Gliomas from Subventricular Zone: Proteomic Drivers of Aggressiveness Using Fluorescence-Guided Multiple Sampling.* Omics-a Journal of Integrative Biology, 2023. **27**(12): p. 598-606.

46. Lang, F.C., et al., *Abrogation of the G2/M checkpoint as a chemosensitization approach for alkylating agents.* Neuro-Oncology, 2024. **26**(6): p. 1083-1096.

47. Chen, H.Z., S.Y. Tsai, and G. Leone, *Emerging roles of E2Fs in cancer: an exit from cell cycle control.* Nat Rev Cancer, 2009. **9**(11): p. 785-97.

48. Li, F., et al., *In Vivo Epigenetic CRISPR Screen Identifies Asf1a as an Immunotherapeutic Target in Kras-Mutant Lung Adenocarcinoma.* Cancer Discov, 2020. **10**(2): p. 270-287.

49. Wu, Y., et al., *ASF1a inhibition induces p53-dependent growth arrest and senescence of cancer cells.* Cell Death Dis, 2019. **10**(2): p. 76.

50. Yin, X., et al., *Histone chaperone ASF1A accelerates chronic myeloid leukemia blast crisis by activating Notch signaling.* Cell Death Dis, 2022. **13**(10): p. 842.

51. Su, L., et al., *H2A.Z.1 crosstalk with H3K56-acetylation controls gliogenesis through the transcription of folate receptor.* Nucleic Acids Res, 2018. **46**(17): p. 8817-8831.

52. Wang, M.H., et al., *Oligophrenin-1 moderates behavioral responses to stress by regulating parvalbumin interneuron activity in the medial prefrontal cortex.* Neuron, 2021. **109**(10): p. 1636-+.

53. Huang, J.J., et al., *WDR62 regulates spindle dynamics as an adaptor protein between TPX2/Aurora A and katanin.* Journal of Cell Biology, 2021. **220**(8).

54. Liu, J., et al., *Androgen deprivation‐induced OPHN1 amplification promotes castration‐resistant prostate cancer.* Oncol Rep, 2022. **47**(1).

55. Chen, W., et al., *Single-cell RNA-seq reveals MIF-(CD74+CXCR4) dependent inhibition of macrophages in metastatic papillary thyroid carcinoma.* Oral Oncology, 2024. **148**.

56. Takheaw, N., et al., *The presence of membrane bound CD99 ligands on leukocyte surface.* BMC Res Notes, 2020. **13**(1): p. 496.

57. Yan, Y., et al., *Multi-omic profiling highlights factors associated with resistance to immuno-chemotherapy in non-small-cell lung cancer.* Nature Genetics, 2025. **57**(1).

58. Zhu, Y.Y., et al., *Comprehensive pan-cancer analysis of KLRB1-CLEC2D pair and identification of small molecule inhibitors to disrupt their interaction.* International Immunopharmacology, 2024. **140**.

59. Mathewson, N.D., et al., *Inhibitory CD161 receptor identified in glioma-infiltrating T cells by single-cell analysis.* Cell, 2021. **184**(5): p. 1281-+.

60. Jankauskas, S.S., et al., *Evolving complexity of MIF signaling.* Cellular Signalling, 2019. **57**: p. 76-88.

61. Klasen, C., et al., *MIF Promotes B Cell Chemotaxis through the Receptors CXCR4 and CD74 and ZAP-70 Signaling.* Journal of Immunology, 2014. **192**(11): p. 5273-5284.

62. Shi, Y.H., et al., *EFNB1 Acts as a Novel Prognosis Marker in Glioblastoma through Bioinformatics Methods and Experimental Validation.* Journal of Oncology, 2021. **2021**.

63. Liu, B.L., et al., *Single-cell meta-analyses reveal responses of tumor-reactive <i>CXCL13</i>⁺ T cells to immune-checkpoint blockade.* Nature Cancer, 2022. **3**(9): p. 1123-+.

64. Zhao, F.Y., et al., *The CXCR4-CXCL12 axis promotes T cell reconstitution via efficient hematopoietic immigration.* Journal of Genetics and Genomics, 2022. **49**(12): p. 1138-1150.

65. You, Y.C., et al., *Crystalline silica-induced recruitment and immuno-imbalance of CD4⁺ tissue resident memory T cells promote silicosis progression.* Communications Biology, 2024. **7**(1).

66. Zhang, Z.H., et al., *KLRB1 defines an activated phenotype of CD4+T cells and shows significant upregulation in patients with primary Sjo<spacing diaeresis>gren's syndrome.* International Immunopharmacology, 2024. **133**.

67. Yeo, E.C.F., et al., *The Role of Cytokines and Chemokines in Shaping the Immune Microenvironment of Glioblastoma: Implications for Immunotherapy.* Cells, 2021. **10**(3).

68. 涂冬萍, et al., *FOS 蛋白的研究进展及生物信息学分析 %J 湖北农业科学 %J Hubei Agricultural Sciences.* 2015(7): p. 1537-1542.

69. Künzli, M. and D. Masopust, *CD4⁺ T cell memory.* Nature Immunology, 2023. **24**(6): p. 903-914.

70. Yang, L.X., et al., *ETS1 promoted cell growth, metastasis and epithelial-mesenchymal transition*

process in melanoma by regulating miR-16-mediated SOX4 expression. Melanoma Research, 2021. **31**(4): p. 298-308.

71. Bhutada, I., et al., *CDK7 and CDK9 inhibition interferes with transcription, translation, and stemness, and induces cytotoxicity in GBM irrespective of temozolomide sensitivity.* Neuro-Oncology, 2024. **26**(1): p. 70-84.

72. Zhou, P.P., et al., *Single-cell CRISPR screens in vivo map T cell fate regulomes in cancer.* Nature, 2023. **624**(7990): p. 154-+.

73. Shin, B.Y., et al., *Runx factors launch T cell and innate lymphoid programs via direct and gene network-based mechanisms.* Nature Immunology, 2023.

74. Liu, Z.Z., et al., *Epigenetic reprogramming of Runx3 reinforces CD8+T-cell function and improves the clinical response to immunotherapy.* Molecular Cancer, 2023. **22**(1).

75. Milner, J.J., et al., *Runx3 programs CD8⁺ T cell residency in non-lymphoid tissues and tumours.* Nature, 2017. **552**(7684): p. 253-+.

76. Kang, P., et al., *<i>Drosophila</i> Kruppel homolog 1 represses lipolysis through interaction with dFOXO.* Scientific Reports, 2017. **7**.

77. Diacou, R., et al., *Cell fate decisions, transcription factors and signaling during early retinal development.* Prog Retin Eye Res, 2022. **91**: p. 101093.

78. Ito, Y. and K. Miyazono, *RUNX transcription factors as key targets of TGF-beta superfamily signaling.* Curr Opin Genet Dev, 2003. **13**(1): p. 43-7.

79. Egawa, T. and D.R. Littman, *ThPOK acts late in specification of the helper T cell lineage and suppresses Runx-mediated commitment to the cytotoxic T cell lineage.* Nat Immunol, 2008. **9**(10): p. 1131-9.

80. Pombero, A., R. Garcia-Lopez, and S. Martínez, *Pericyte-Glioblastoma Cell Interaction: A Key Target to Prevent Glioblastoma Progression.* Cells, 2023. **12**(9).

81. Mohan, S., et al., *Detection of occult neoplastic infiltration in the corpus callosum and prediction of overall survival in patients with glioblastoma using diffusion tensor imaging.* Eur J Radiol, 2019. **112**: p. 106-111.

82. Cloughesy, T.F., et al., *Neoadjuvant anti-PD-1 immunotherapy promotes a survival benefit with intratumoral and systemic immune responses in recurrent glioblastoma.* Nature Medicine, 2019. **25**(3): p. 477-+.

83. Mathios, D. and M. Lim, *Why is immunotherapy for glioblastoma "Lag"-ging.* Oncotarget, 2019. **10**(12): p. 1228-1229.

Figure 1

Click here to access/download;Figure;Figure 1.tif

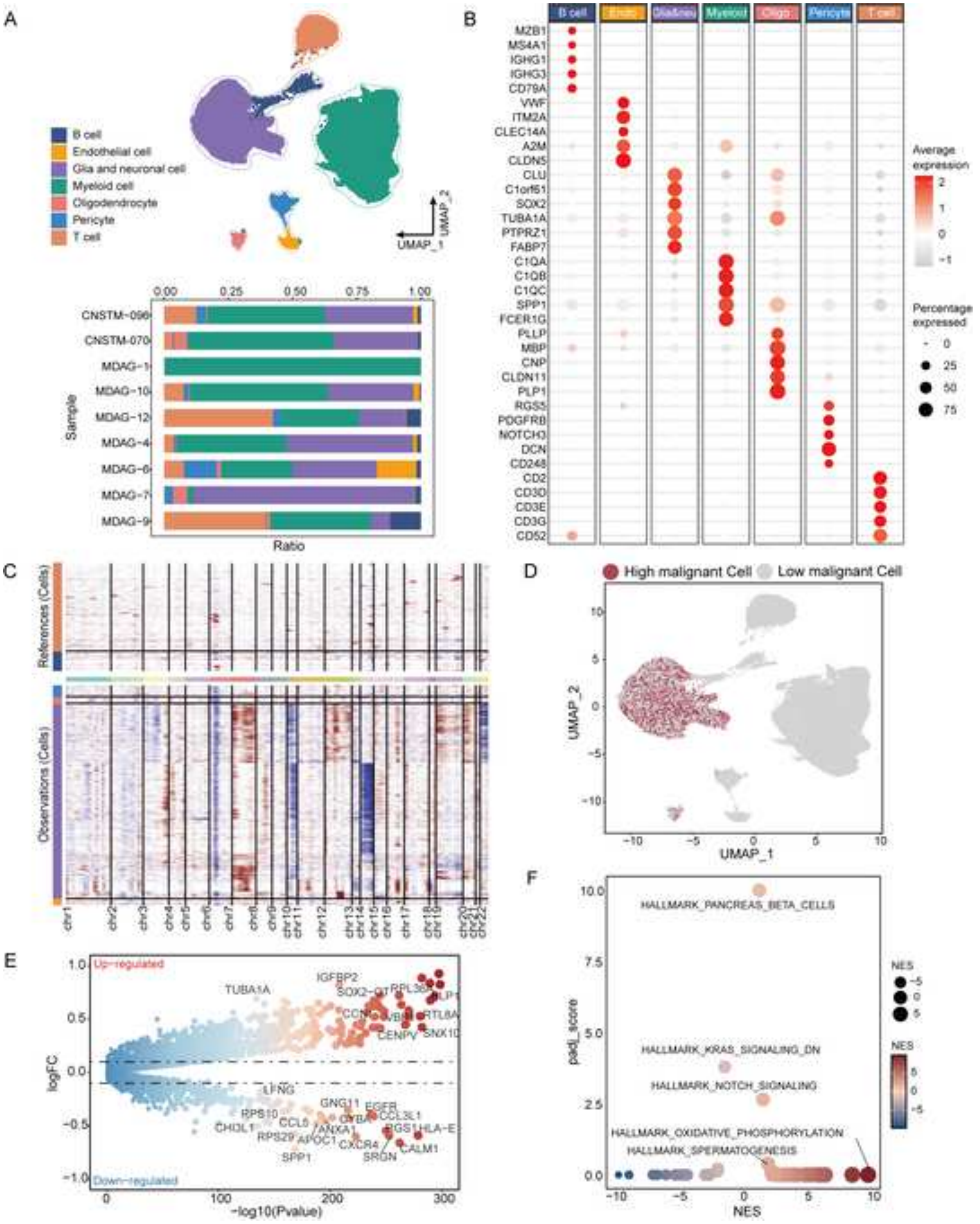

Figure 2

Click here to access/download;Figure;Figure 2.tif

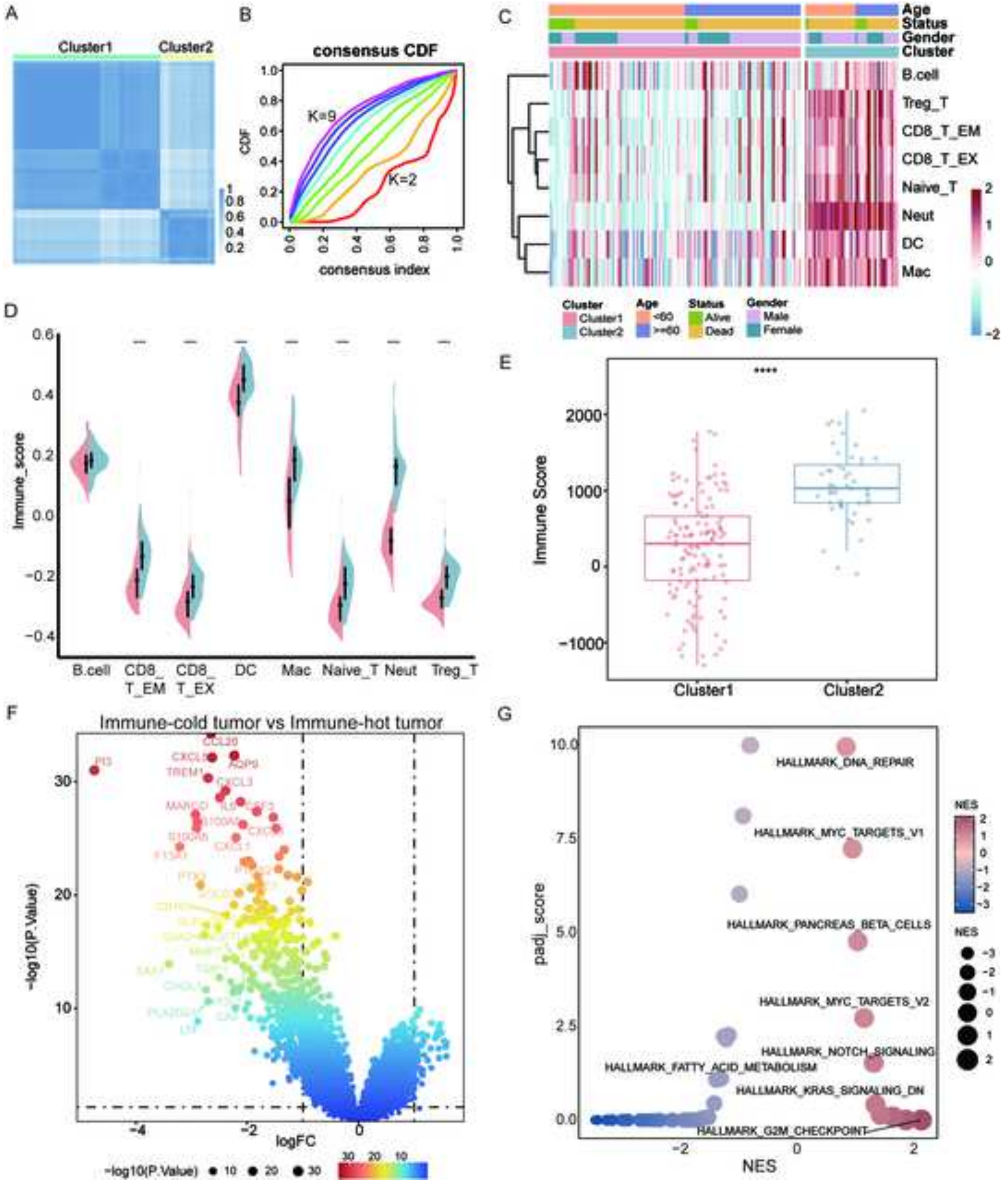

Figure 3 Click here to access/download;Figure;Figure 3.tif

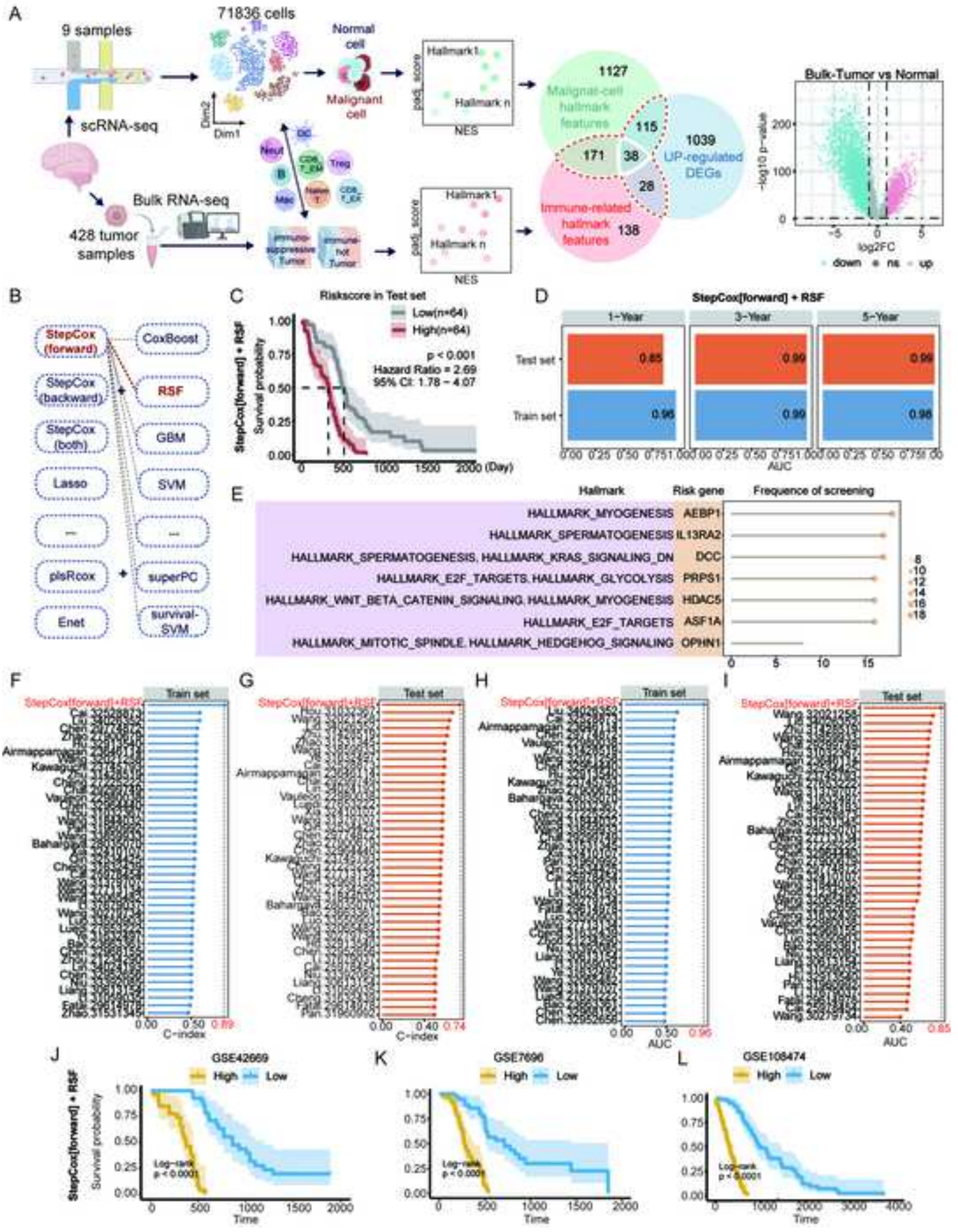

Figure 4 Click here to access/download;Figure;Figure 4.tif

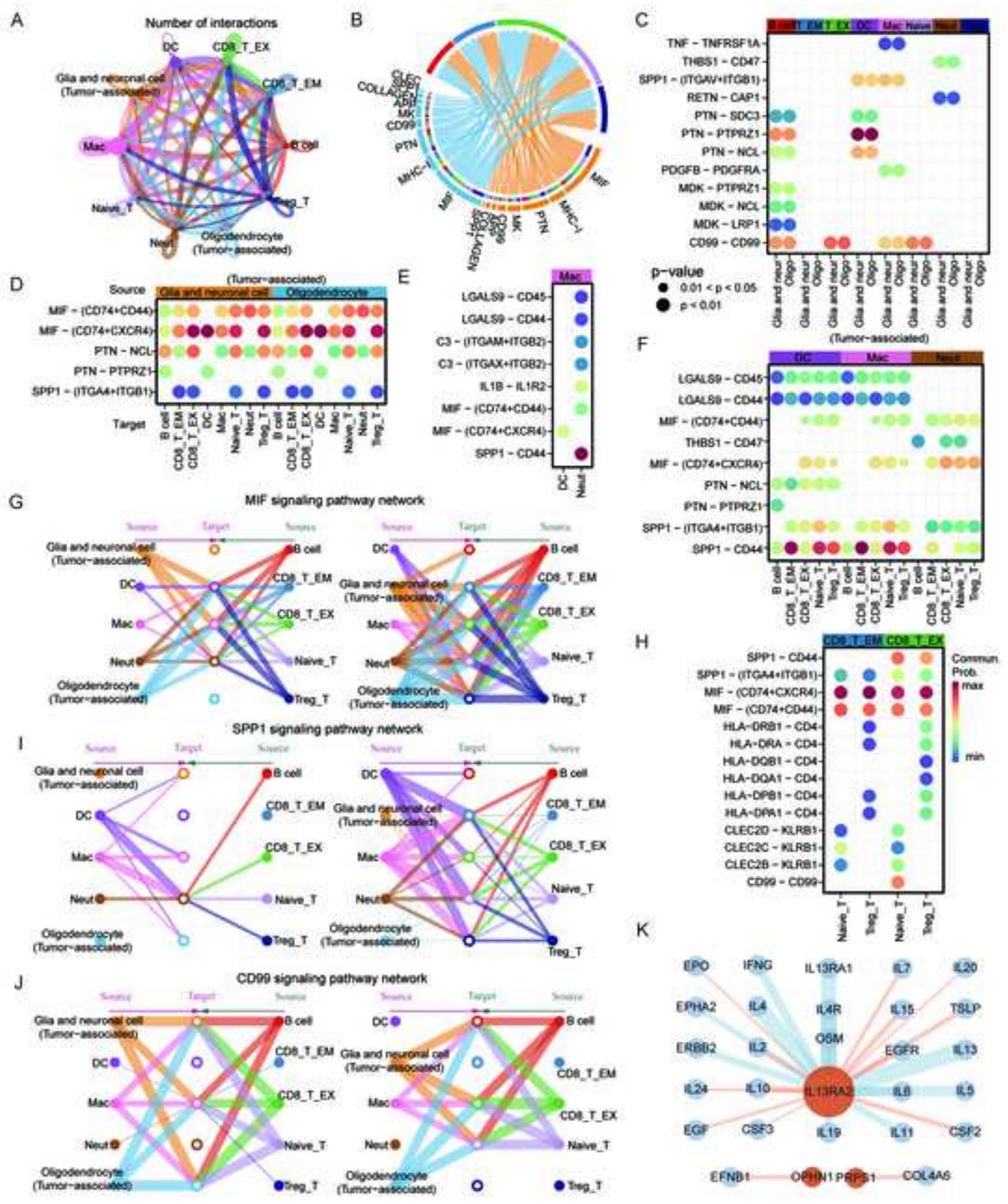

Figure 5

Click here to access/download;Figure;Figure 5.tif

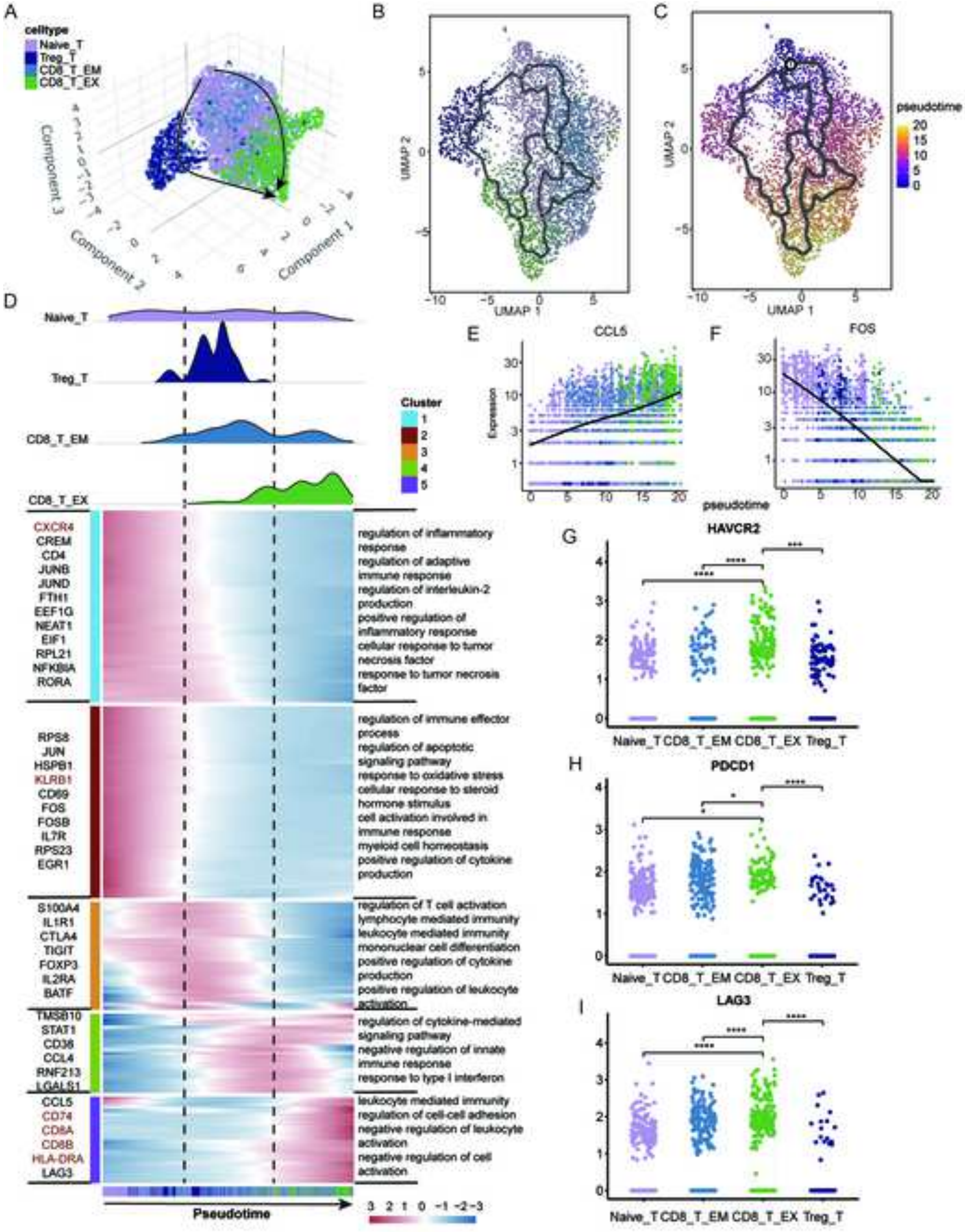

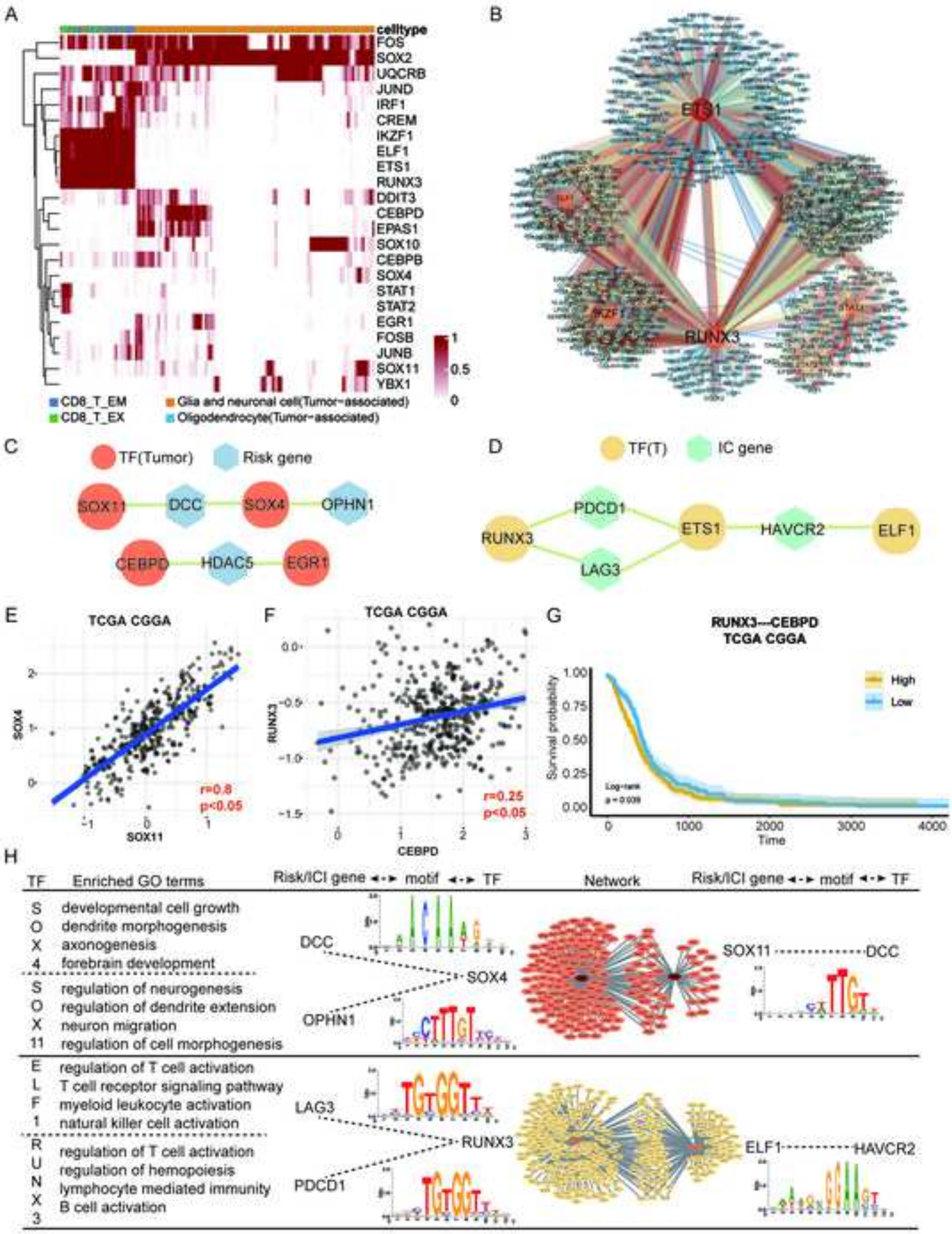
A
celltype
FOS
SOX2
UQCRB
JUND
IRF1
CREM
IKZF1
ELF1
ETS1
RUNX3
DDIT3
CEBPD
EPAS1
SOX10
CEBPB
SOX4
STAT1
STAT2
EGR1
FOSB
JUNB
SOX11
YBX1
1
0.5
0
CD8_T_EM
CD8_T_EX
Glia and neuronal cell(Tumor-associated)
Oligodendrocyte(Tumor-associated)
B
ETS1
RUNX3
C
TF(Tumor)
Risk gene
SOX11
DCC
SOX4
OPHN1
CEBPD
HDAC5
EGR1
D
TF(T)
IC gene
RUNX3
PDCD1
LAG3
ETS1
HAVCR2
ELF1
E
TCGA CGGA
SOX4
SOX11
r=0.8
p<0.05
F
TCGA CGGA
RUNX3
CEBPD
r=0.25
p<0.05
G
RUNX3---CEBPD
TCGA CGGA
Survival probability
High
Low
Time
H
TF
Enriched GO terms
Risk/ICI gene ◄-► motif ◄-► TF
Network
Risk/ICI gene ◄-► motif ◄-► TF
SOX4
developmental cell growth
dendrite morphogenesis
axonogenesis
forebrain development
SOX11
regulation of neurogenesis
regulation of dendrite extension
neuron migration
regulation of cell morphogenesis
DCC
SOX4
OPHN1
SOX11 DCC
ELF1
regulation of T cell activation
T cell receptor signaling pathway
myeloid leukocyte activation
natural killer cell activation
RUNX3
regulation of T cell activation
regulation of hemopoiesis
lymphocyte mediated immunity
B cell activation
LAG3
RUNX3
PDCD1
ELF1 HAVCR2

Figure 7

Click here to access/download;Figure;Figure 7.tif

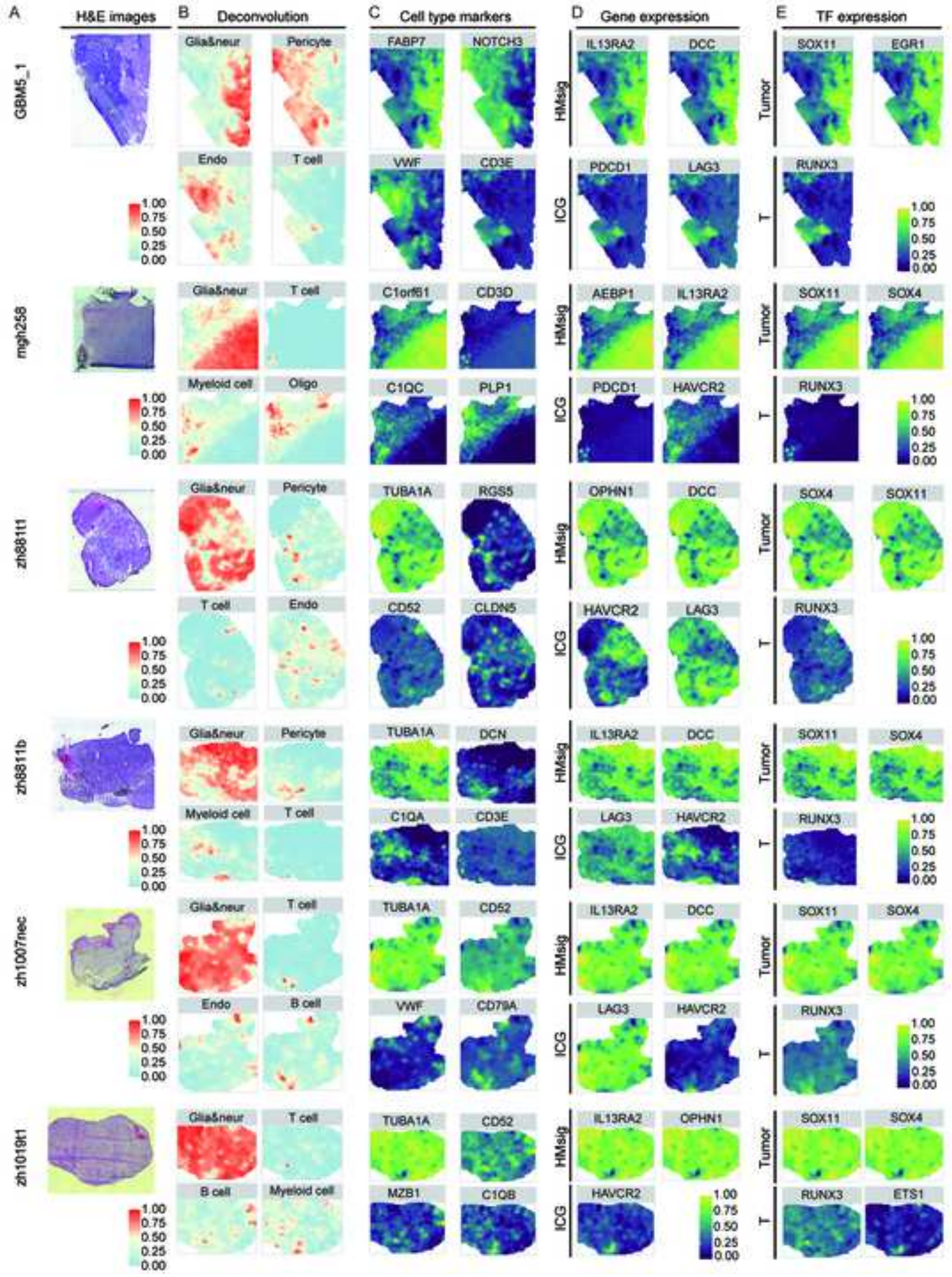

Click here to access/download
Supplementary Material
Supplementary Figure.docx

Supplementary Figure1

Click here to access/download
Supplementary Material
Supplementary Figure1.pdf

Supplementary Figure2

Click here to access/download
Supplementary Material
Supplementary Figure2.pdf

Supplementary Figure3

Click here to access/download
Supplementary Material
Supplementary Figure3.pdf

Supplementary Figure4

Click here to access/download
Supplementary Material
Supplementary Figure4.pdf

Click here to access/download
Supplementary Material
Supplementary Figure5.pdf

Supplementary Table 1

Click here to access/download
Supplementary Material
Supplementary Table 1.xlsx

Supplementary Table 2

Click here to access/download
Supplementary Material
Supplementary Table 2.xlsx

Click here to access/download

Supplementary Material

Supplementary Table 3.xlsx

Dear Editors of GigaScience:

We would like to submit the enclosed manuscript entitled "**Dissecting glioblastoma risk signatures in the tumor immune microenvironment based on multi-dimensional transcriptomics**", which we wish to be considered for publication in GigaScience.

Glioblastoma (GBM) diffusely infiltrates the brain, intermingling with non-neoplastic brain cells. This intricate TME forms the biological basis for treatment response and tumor recurrence. Currently, the recurrence rate of GBM patients after treatment exceeds 90%. Only a negligible number of patients are likely to approach a state of cure. As a result, it is of utmost importance to delve deeply into the interactions between GBM cells and their immune microenvironment.

Herein, we comprehensively dissected the tumor microenvironment (TME) and uncovered potential molecular mechanisms by integrating single-cell, bulk, and spatial transcriptomic data. We identified seven hallmark-related prognostic signatures (HMsig) using machine learning algorithm, namely AEBP1, ASF1A, PRPS1, DCC, OPHN1, IL13RA2, and HDAC5. The influence of HMsig on the prognosis of patients was confirmed by the SHAP algorithm. no studies have yet reported that ASF1A and OPHN1 can be a risk gene for GBM, and the interactions involving OPHN1 were associated with poorer prognosis. Additionally, this study revealed diverse immune escape mechanisms in GBM, including the interaction of key LR pairs between cell clusters, the upregulation of immune checkpoint genes (ICG), and the synergistic regulation between tumor-related HMsig signatures and ICGs. Spatial transcriptomic analysis consistently demonstrated the existence of synergistic gene interactions, deciphering the immunomodulatory functions of GBM biomarkers in the TME. Collectively, these results not only enhance our understanding of the complexity of the TME but also provide important theoretical foundations and new research directions for the diagnosis and treatment of GBM.

This paper is a full-length manuscript that has not simultaneously been submitted elsewhere for print or electronic publication. This submission has been agreed by all the co-authors for this paper.

All the co-authors declare no conflict of interest.

We are very glad to take this opportunity to submit our work to GigaScience and highly appreciate your editorial work and suggestions for this manuscript.

Respectfully yours,

Congxue Hu, Ph.D.
College of Bioinformatics Science and Technology
Harbin Medical University
194 Xuefu Road, Harbin 150081, China
Phone: 86-451-86615922
Fax: 86-451-86615922
Email: hucx1996@hrbmu.edu.cn

Graphical abstract

Click here to access/download;Graphical Abstract;Graphical abstract.pdf

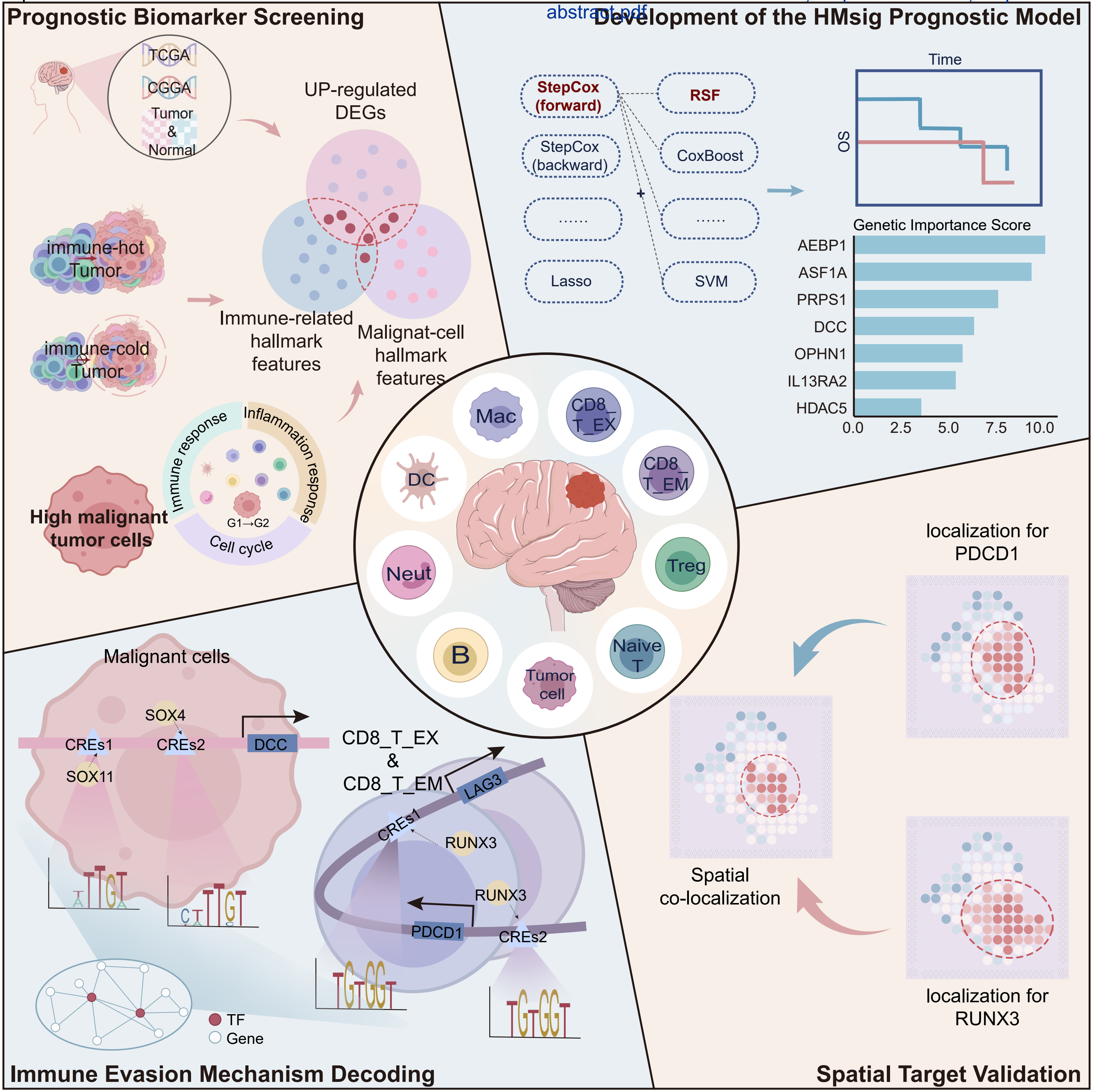

Supplement: giag035_GIGA-D-26-00037_original_submission [file giag035_giga-d-26-00037_original_submission.pdf]
