# Supplementary material for: Dissecting glioblastoma risk signatures in the tumor immune microenvironment based on multi-dimensional transcriptomics
Source: Gigascience. 2026 Mar 25;15:giag035. doi: 10.1093/gigascience/giag035 (PMC13154832; doi:10.1093/gigascience/giag035)
Supplement: giag035_GIGA-D-26-00037_Revision_1 [file giag035_giga-d-26-00037_revision_1.pdf]

GigaScience

Dissecting glioblastoma risk signatures in the tumor immune microenvironment based on multi-dimensional transcriptomics

--Manuscript Draft--

**Manuscript Number:**	GIGA-D-26-00037R1
**Full Title:**	Dissecting glioblastoma risk signatures in the tumor immune microenvironment based on multi-dimensional transcriptomics
**Article Type:**	Research
**Funding Information:**	National Major Science and Technology Projects of China (2024ZD0530500) — Prof Yunpeng Zhang<br>National Natural Science Foundation of China (62472131) — Prof Yunpeng Zhang<br>National Natural Science Foundation of China (62502128) — Dr Congxue Hu<br>Key Research and Development Program of Heilongjiang (2024ZX12C27) — Prof Yunpeng Zhang<br>China Postdoctoral Science Foundation (2024M760709) — Dr Congxue Hu<br>Heilongjiang Provincial Postdoctoral Science Foundation (LBH-Z24210) — Dr Congxue Hu<br>Longjiang New Era Outstanding Doctoral Dissertation Project Grant (LJYXL2024-069) — Dr Congxue Hu<br>Harbin Medical University Fund (2025-KYYWF-ZR0297) — Dr Congxue Hu
**Abstract:**	Glioblastoma (GBM) is characterized by pronounced tumor heterogeneity and a complex immune microenvironment, contributing to poor patient survival outcomes. In this study, we comprehensively dissected the tumor microenvironment (TME) and uncovered potential molecular mechanisms by integrating single-cell, bulk, and spatial transcriptomic data. Hallmarks of malignancy and cell cycle regulatory pathways were consistently enriched across these modalities, highlighting their pivotal roles in transcriptional regulation. We identified seven hallmark-related prognostic signatures (HMsig) using machine learning algorithm, namely AEBP1, ASF1A, PRPS1, DCC, OPHN1, IL13RA2, and HDAC5—whose importance in predicting patient outcomes was validated through SHAP algorithm analysis. Ligand-receptor (LR) interaction analysis revealed that interactions involving OPHN1 were associated with poorer prognosis. Additionally, Immune checkpoint genes (ICG) LAG3, PDCD1, and HAVCR2 were found to be substantially upregulated along the pseudotime trajectory of T-cell progression. Synergistic transcriptional regulation between tumor-related HMsig signatures and ICGs in T cells was identified as a key factor influencing patient survival. Spatial transcriptomic analysis consistently demonstrated the existence of synergistic gene interactions, deciphering the immunomodulatory functions of GBM biomarkers in the TME.
**Corresponding Author:**	Congxue Hu, Ph.D<br>Harbin Medical University<br>Harbin, Heilongjiang Province CHINA
**Corresponding Author Secondary Information:**	
**Corresponding Author's Institution:**	Harbin Medical University
**Corresponding Author's Secondary Institution:**	
**First Author:**	Tengyue Li

Powered by Editorial Manager® and ProduXion Manager® from Aries Systems Corporation

**First Author Secondary Information:**	
**Order of Authors:**	Tengyue Li
	Wanqi Mi
	Huarui Yan
	Yining Ma
	Han Jiang
	Xiaoxu Yang
	Yunpeng Zhang
	Congxue Hu, Ph.D
**Order of Authors Secondary Information:**	
**Response to Reviewers:**	Dear Editors of GigaScience:<br>We sincerely thank the Editor and the Reviewers for their careful evaluation and constructive comments on our manuscript, “Dissecting glioblastoma risk signatures in the tumor immune microenvironment based on multi-dimensional transcriptomics” (ID: GIGA-D-26-00037). The insightful comments have significantly helped us to strengthen our study.<br>First, we added a description of the spermatogenesis pathway in GBM and the mechanism of the OPHN1-EFNB1 interaction, along with relevant references. This analysis has been incorporated into the Results and Discussion sections to enhance the depth of the study and add biological significance to the findings.<br>Second, to improve the transparency and reproducibility of our methods, we provided a detailed explanation of the machine learning framework and feature selection process, which led to the final selection of 7 core genes (HMsig) from the initial 354 genes. Additionally, key parameters for all computational analyses, including software versions (e.g., Harmony v1.2.1 and inferCNV v1.10.1), clustering resolution, and root cell selection in Monocle3, are clearly documented in the Methods section to ensure the rigor of our analysis.<br>Next, to further clarify the immune evasion mechanism, the study focuses on the transition of CD8+ effector memory T cells to CD8+ exhausted T cells, which represents a key functional change in the GBM microenvironment. We also used the CARD algorithm for spatial deconvolution to confirm the co-localization of HMsig genes with transcription factors such as SOX4, SOX11, and RUNX3, providing a structural basis for the immunosuppressive environment in GBM.<br>We believe these revisions comprehensively address the reviewer’s concerns and improve the overall quality of our manuscript. All changes in the revised manuscript are highlighted in red font. The response letter will be attached with the revised manuscript, figures, tables, and supplementary materials. We sincerely thank you and the reviewers for your professional suggestions. Below are our detailed point-by-point responses to the reviewer’s comments:<br><br>Reviewer #1<br>Comment 1: In the first Results section, Gene Set Enrichment Analysis (GSEA) revealed significant enrichment of the "Spermatogenesis" pathway in both single-cell and bulk transcriptomic datasets. Given that GBM is not a tumor of the reproductive system, the biological relevance of this finding is unclear. The authors need to provide a mechanistic explanation or relevant literature support to justify this observation.<br>Response: We appreciate the reviewer pointing this out. To elucidate the biological significance of the "Spermatogenesis" pathway enrichment in GBM, we have incorporated mechanistic descriptions and supporting literature into the Results section of the revised manuscript. First, evidence indicates that the abnormal expression of spermatogenesis-related genes is strongly associated with glioma prognosis and malignancy grade, which suggests these genes have functional roles beyond reproductive tissues[1]. Second, recent studies suggest that GBM may exploit immune privilege mechanisms by expressing reproductive antigens. This process potentially recruits Tregs that were pre-activated in the reproductive system, and androgens may

further support the expression of these genes in GBM[2]. Third, the spermatogenesis-associated gene Sohlh1 has been demonstrated to suppress GBM cell proliferation, migration, and invasion by inhibiting the Wnt/β-catenin signaling pathway[3]. Collectively, these findings suggest that the spermatogenesis pathway play an important role in GBM progression. We have incorporated these points into the Results section.

Based on the comments above, we have revised the manuscript at the appropriate place. The main text is as follows:
Interestingly, the enrichment of the spermatogenesis pathway in GBM suggests that some genes involved in spermatogenesis may play a role beyond their traditional functions in reproductive tissues. Previous studies have indicated that abnormal expression of spermatogenesis-related genes is associated with glioma progression and malignancy (34). In addition, research has shown that GBM may exploit immune privilege mechanisms by expressing reproductive antigens, which could recruit Tregs activated in the reproductive system (35). Notably, the spermatogenesis gene Sohlh1 has been shown to inhibit GBM cell proliferation and migration by modulating the Wnt/β-catenin signaling pathway (36), further supporting the essential roles of spermatogenesis-related genes in GBM progression. (Page 17-18, Lines 331-341)
Comment 2: In Results 3, the authors report that 42 prognostic-related genes were initially identified through machine learning approaches, but the final HMsig model includes only seven genes. The specific filtering criteria and selection strategy that led from 42 to 7 genes should be explicitly described in the Methods section to improve transparency and reproducibility.
Response: We agree with the reviewer that methods transparency is required in the feature selection process. First, we screened all characteristic genes using univariate Cox regression and Kaplan-Meier (KM) survival analysis, retaining genes with significant prognostic value in both tests ($P < 0.05$), ultimately identifying 42 high-value candidate genes. Next, we evaluated these 42 genes using a combinatorial machine learning algorithm, finding that the "StepCox (forward) + RSF" combination had the highest C-index. Based on this optimal combination, we used StepCox (forward) to select 7 core genes from the 42 candidate genes that contributed most to survival prediction, defined as HMsig. Subsequently, we trained these 7 core genes using the RSF model, ultimately obtaining the best survival prediction performance. This transparent screening process has been detailed in the Methods section of our manuscript.

Based on the comments above, we have revised the manuscript at the appropriate place. The main text is as follows:
To construct a model for predicting the prognosis of GBM patients, we first divided all bulk tumor samples into training and test sets at a ratio of 7:3. Then, we performed univariate Cox regression and Kaplan-Meier (KM) survival analysis on the 354 GBM progression-related feature genes identified across multiple dimensions. Only genes that showed statistically significant prognostic relevance ($P < 0.05$) in both tests were retained, resulting in 42 high-value candidate genes. These pre-screened genes were input into an integrated machine learning framework built using the R package mime1(23).
We constructed and evaluated the model on both the training and test sets using 10-fold cross-validation, selecting the combined model with the highest C-index as the optimal model. Based on this optimal model, we further performed feature screening, ultimately identifying 7 prognostic hallmark characteristic genes (HMsig). To comprehensively evaluate the model, we performed a meta-analysis integrating the training and test sets, and conducted ROC analyses on 1-year, 3-year, and 5-year survival predictions for GBM patients. Model performance was compared with existing methods using C-index and ROC values. Furthermore, we collected three external validation sets from the GEO database to validate the model's performance in predicting patient outcomes (Supplementary Table 1). (Page 10, Lines 181-198)

Comment 3: The study intersects gene sets derived from three analytical dimensions to generate the final feature set used for model construction. The biological rationale and conceptual significance of this intersection strategy should be clarified in the Results section.
Response: We thank the reviewer for this insightful comment. Single-cell hallmark genes capture the intrinsic malignant states of GBM tumor cells; bulk tumor hallmark

genes reflect tumor immune cold characteristics; and the bulk DEGs ensure the features have sufficient expression abundance and universality across large patient cohorts. By performing intersection analysis on these three gene sets, we effectively integrated the hallmark features of multidimensional transcriptomic data. This feature gene set represents key genes that can both drive the intrinsic malignant proliferation of tumor cells and regulate the immunosuppressive state of the microenvironment. We have now clarified this intersection strategy in the revised Results section.

Based on the comments above, we have revised the manuscript at the appropriate place. The main text is as follows:
We used a multi-dimensional intersection strategy to identify hallmark-related feature set for prognostic modeling. This approach integrates three key aspects: the intrinsic malignant characteristics of GBM tumor cells captured by single-cell RNA-seq hallmark genes, the immune cold environment features reflected by bulk tumor hallmark genes, and the overall transcriptomic expression universality across patient populations represented by bulk DEGs. By focusing on the overlap of these gene sets, we prioritized genes that are critical not only for the malignant progression of GBM cells but also for shaping the immunosuppressive environment (Figure 3A). (Page 23, Lines 455-463)

Comment 4: In Results 4, the authors report a protein-protein interaction between OPHN1 and the ligand EFNB1 and suggest its association with patient prognosis. It would strengthen the manuscript if the Discussion section further explored the potential biological mechanisms underlying this interaction in GBM.
Response: We thank the reviewer for this insightful comment. We further investigated the biological mechanisms related to the interaction between EFNB1 and OPHN1. Current evidence identifies EFNB1 as a pivotal driver of glioma cell invasion and a key factor in remodeling the tumor immune microenvironment[4, 5]. The interaction between OPHN1 and EFNB1 likely converges on the Rho-GTPase signaling axis. Specifically, OPHN1 works as a Rho-GAP, which helps control the activity of RhoA and Rac1—both of which are important molecules involved in Ephrin signaling [6]. This interaction seems to help control the structure of the cell, which allows GBM cells to invade and spread more aggressively into the brain tissue. We have expanded our Discussion to explore the mechanistic synergy between OPHN1 and EFNB1.

Based on the comments above, we have revised the manuscript at the appropriate place. The main text is as follows:
Notably, the OPHN1-EFNB1 interaction emerged as a critical determinant of poor prognosis. As EFNB1 drives glioma invasion and immune remodeling(91, 92), its interplay with OPHN1—a Rho-GAP modulating RhoA/Rac1 activity—likely governs cytoskeletal plasticity(93). This synergy facilitates GBM cells to invade and spread more aggressively into the brain tissue. (Page 41, Lines 851-856)

Comment 5: For the T-cell developmental trajectory analysis using Monocle3, the manuscript provides limited methodological detail regarding root state selection and pseudotime initialization. The authors should clarify the computational procedures and specific functions used for trajectory inference and root cell designation in the Methods section.
Response: We thank the reviewer for highlighting the need for technical clarity in our trajectory analysis. To ensure the reproducibility and biological validity of the T-cell developmental inference, we followed a standardized computational procedure in Monocle3. The root of this trajectory selected Naive T cells. We used 'get_earliest_principal_node' function to identify the earliest principal node from Naive T cells, which is a helper function provided by monocle3. This node was then designated as the root node using the 'order_cells' function to calculate pseudotime values for all cells. We have updated the Methods section to include these specific functions and the underlying computational methods.

Based on the comments above, we have revised the manuscript at the appropriate place. The main text is as follows:
We used 'get_earliest_principal_node' function to identify the earliest principal node from Naive T cells, which is a helper function provided by monocle3. This node was then designated as the root node using the 'order_cells' function to calculate pseudotime values for all cells. (Page 12, Lines 231-235)

Powered by Editorial Manager® and ProduXion Manager® from Aries Systems Corporation

Comment 6: In the "Dimensionality reduction and clustering of cells" section, Harmony was used for batch correction across samples. Considering the availability of multiple single-cell batch integration algorithms, the authors should provide a technical justification for selecting Harmony over alternative approaches.
Response: We appreciate the reviewer’s comment and the opportunity to clarify our methodological choice. We chose Harmony for batch effect integration due to its established performance and computational efficiency, as supported by an independent benchmark study[7]. Harmony was selected over other methods such as Seurat v3 or LIGER for a few reasons. First, Harmony has been shown to effectively integrate datasets with common cell types, minimizing batch effects while preserving biological signals. Second, it demonstrated robustness when applied to diverse sequencing technologies and experimental conditions. Finally, Harmony’s computational efficiency, with a lower runtime and memory footprint compared to other methods, made it particularly suited for our large-scale data analysis. In summary, Harmony provided an optimal balance between accuracy and computational scalability, making it the ideal choice for our study.

Comment 7: Several computational tools (e.g., inferCNV, Harmony, CellMarker2.0) are mentioned without version numbers or corresponding references. To meet reproducibility standards, the Methods section should include detailed version information and appropriate citations.
Response: We thank the reviewer for this insightful comment. In accordance with the reviewer’s request, we have updated the Methods section to include the version information for key software packages and databases used in this study. Furthermore, we have added the references for inferCNV, Harmony, and CellMarker2.0 to the bibliography. The following table summarizes the updated information:
Tool/DatabaseVersionReference
Harmonyv1.2.1PMID: 31740819
inferCNVv1.10.1PMID: 24925914
CellMarker2.0Online DatabasePMID: 36300619
The Methods section has been revised to include these versions and citations (Page 7-8, Lines 135-136; Page 8, Lines 147; Page 8, Lines 143).

Comment 8: The inferCNV method is commonly applied to epithelial-derived tumors to infer copy number variation patterns. Given the relative scarcity of its application in brain tumors, the authors should provide theoretical justification and relevant references supporting the use of inferCNV in GBM.
Response: We appreciate the reviewer's comment. The method inferCNV was fundamentally inspired by and developed for the study of brain tumors. Single-cell studies in glioblastoma and oligodendroglioma established the theoretical framework for inferring copy number variations (CNVs) from transcriptomic data[8-10]. Furthermore, the official implementation of inferCNV by the Broad Institute uses oligodendroglioma expression data as its primary benchmark and example dataset. The Methods section has been revised to include the descriptions and citations.

Based on the comments above, we have revised the manuscript at the appropriate place. The main text is as follows:
This methodology is particularly robust for GBM as it was fundamentally established and validated through foundational single cell research in brain tumors (20-22). (Page 8, Lines 149-151)

Comment 9: In Results 7, spatial transcriptomic analyses revealed colocalization of certain GBM feature molecules at the tissue level. The Discussion section would benefit from a more comprehensive synthesis of these spatial colocalization patterns and their potential biological implications.
Response: We sincerely thank the reviewers for their valuable suggestions. In the revised text, we emphasize that the spatial co-localization of the HMsig gene and immune checkpoint genes, along with their corresponding transcription factors (such as SOX4, SOX11, and RUNX3), in specific tissue regions represents an immunosuppressive microenvironment. On one hand, SOX4 and SOX11 regulate the expression of the HMsig gene, promoting the malignant progression of GBM. On the other hand, RUNX3 upregulates immune checkpoint genes, thereby promoting immune escape by tumor cells. The synergistic expression of these two types of

transcription factors in space jointly promotes the malignant progression of GBM and enhances immune escape capabilities, forming an immunosuppressive microenvironment. We have incorporated these points into the discussion section.

Based on the comments above, we have revised the manuscript at the appropriate place. The main text is as follows:
Spatial transcriptomic analysis further confirmed the co-localization of the HMsig gene with immune checkpoint genes and their transcription factors (especially SOX4, SOX11, and RUNX3) in specific tissue regions, forming an immunosuppressive microenvironment. The co-expression of SOX4 and SOX11 suggests a spatial regulatory mechanism that enhances HMsig gene expression and drives tumor progression. RUNX3 upregulates the expression of immune checkpoint genes LAG3 and PDCD1, further promoting the co-regulation represented by the "double brake" mechanism, thereby facilitating immune escape by tumor cells. This spatial organization driven by a coordinated transcriptional program provides a structural basis for the persistent treatment resistance in GBM. (Page 42-43, Lines 872-882)

Comment 10: Although the Methods section describes clustering and subclustering of major cell types, the specific resolution parameters used for clustering are not reported. These parameters should be clearly stated to enhance methodological transparency.
Response: We thank the reviewer for this constructive suggestion. In our study, the selection of resolution parameters was guided by the clustree package, which allows for the visualization of clustering stability across a range of resolutions (Supplementary Figure 1A-C). A resolution of 0.2 was used to identify major cell lineages. For the detailed subclustering of T cells and myeloid cells, we applied higher resolutions of 0.9 and 0.8, respectively, to capture fine grained cell states. We have updated the Methods section and added Table S2 to clearly document these parameters.

Based on the comments above, we have revised the manuscript at the appropriate place. The main text is as follows:
To ensure the stability and of the clustering results, we evaluated various resolution parameters using the clustree package (version 0.5.1). A resolution of 0.2 was applied to identify major cell lineages. For the subclustering of specific populations, we utilized resolutions of 0.9 for T cells and 0.8 for myeloid cells to resolve fine grained cell subtypes. (Page 8, Lines 137-140)

Reviewer #2
Comment 1: The Methods section does not clearly describe the workflow or key parameters used for subclustering and annotation of T-cell and myeloid cell populations. A detailed account of the analytical pipeline should be provided to enhance reproducibility.
Response: We fully agree with the reviewer that methodological transparency is essential for scientific reproducibility. We have revised the Methods section to provide a comprehensive analytical pipeline used for the subclustering and annotation of T cell and myeloid cell populations. The updated section now explicitly details the following key procedures:
(1) The clustree package was employed to evaluate clustering stability, and we utilized resolutions of 0.9 for T cells and 0.8 for myeloid cells.
(2) We used canonical markers cross referenced with the CellMarker 2.0 database for precise cell annotation. Utilized cell type markers are shown in supplementary figure 1D-E.
To further enhance clarity, we have summarized these key parameters and marker genes in the newly added Table S2. The Methods section has been extensively revised to include these procedural details.

Based on the comments above, we have revised the manuscript at the appropriate place. The main text is as follows:

The complete set of markers used for cell type annotation, along with their corresponding clustering resolutions, is systematically documented (Supplementary Table 2). (Page 8, Lines 143-145)

Comment 2: In Results 2, the authors report concordant enrichment of tumor-associated hallmark pathways at both single-cell and bulk transcriptomic levels. The Discussion section would benefit from a more integrative interpretation of this concordance, particularly in terms of what it implies about transcriptional robustness and malignant program conservation across data modalities.
Response: We thank the reviewer for this suggestion. The five common hallmarks—the E2F targets, G2M checkpoint, spindle mitosis, Wnt signaling, and spermatogenesis—across single-cell and bulk data highlights the transcriptional robustness of the core malignant programs in GBM. These pathways, primarily associated with cell cycle regulation and proliferation, constitute a cross-modal conserved regulatory pattern, avoiding the influence of technical noise inherent in single-modal data. We have updated the Discussion accordingly.

Based on the comments above, we have revised the manuscript at the appropriate place. The main text is as follows:
The five common hallmarks between single cell and bulk dimensions confirms the transcriptional robustness of core malignant programs in GBM. These pathways, primarily associated with cell cycle regulation and proliferation, constitute a cross-modal conserved regulatory pattern, avoiding the influence of technical noise inherent in single-modal data. (Page 40-41, Lines 837-841)

Comment 3: The manuscript should provide appropriate citations and version information for the computational tools and packages used in the study (e.g., Harmony, clusterProfiler). It is essential to meet current standards of methodological transparency.
Response: We thank the reviewer for this important suggestion. In response, we have updated the Methods section to include the version information for all key bioinformatics tools and databases used in this study (Page 7-8, Lines 135-136; Page 12, Lines 139; Page 11, Lines 217-218; Page 12, Lines 223):
ToolVersionReference
Harmonyv1.2.1PMID: 31740819
clusterProfilerv4.12.0PMID: 39019974
STRINGOnline databasePMID: 36370105
Cytoscapev3.9.1PMID: 14597658

Comment 4: In Results 3, the authors analyze the origin the identified HMsig genes. Additional detail is warranted to clarify how these genes emerge from the integrative framework and to discuss the potential biological mechanisms underlying their selection and convergence.
Response: We thank the reviewer for this request for clarification. Our goal was to identify genes that sit at the intersection of three complementary analytical dimensions: hallmark genes from single-cell data capture malignant cell states at high resolution, hallmark genes from bulk cold tumors reflect the immunosuppressive feature, and DEGs from bulk data ensure the selected features are broadly expressed across large patient cohorts. This multi-dimensional filtering helps reduce noise and minimize biases that may exist in any single data type, ensuring that the final HMsig genes are not only biologically relevant but also clinically representative of the GBM microenvironment. We have revised the Results section.

Based on the comments above, we have revised the manuscript at the appropriate place. The main text is as follows:
We used a multi-dimensional intersection strategy to identify hallmark-related feature set for prognostic modeling. This approach integrates three key aspects: the intrinsic malignant characteristics of GBM tumor cells captured by single-cell RNA-seq hallmark genes, the immune cold environment features reflected by bulk tumor hallmark genes, and the overall transcriptomic expression universality across patient populations represented by bulk DEGs. By focusing on the overlap of these gene sets, we prioritized genes that are critical not only for the malignant progression of GBM cells but also for shaping the immunosuppressive environment (Figure 3A).
(Page 23, Lines 455-463)

Powered by Editorial Manager® and ProduXion Manager® from Aries Systems Corporation

Comment 5: The machine learning model incorporates SHAP analysis to quantify the contribution of each gene within HMsig. The manuscript would be strengthened by a more explicit discussion of the biological interpretation of these SHAP values—specifically the directionality and magnitude of feature importance.
Response: We thank the reviewer for this suggestion. The SHAP gene importance score quantifies the contribution of each gene in HMsig to the final risk prediction. Analysis showed that genes such as AEBP1, ASF1A, and PRPS1 were the most important and were the main drivers of the prognostic model. In terms of directionality, the SHAP values of HMsig genes were positively correlated with the risk score. This indicates that higher expression levels of these genes are associated with poorer predicted survival outcomes. We have revised the corresponding Results section.

Based on the comments above, we have revised the manuscript at the appropriate place. The main text is as follows:
SHAP analysis revealed that the AEBP1, ASF1A, and PRPS1 genes had the highest SHAP values, indicating their significant impact on survival prediction. These SHAP values suggest that these three genes are major predictors in HMsig, and their direction is consistently positively correlated with patient risk. This confirms that high expression of these HMsig genes is a key factor leading to elevated risk scores and indicates their role as high-risk determinants in the GBM microenvironment. (Page 25, Lines 494-500)

Comment 6: In Results 4, the authors suggest that CD99-CD99 interactions may upregulate IL-6 and TNF-α, thereby promoting tumor survival. Given that IL-6 is traditionally characterized as a pro-inflammatory cytokine, yet is associated with poor prognosis in GBM, a more detailed explanation of this pro-tumorigenic inflammatory mechanism should be incorporated into the Results section.
Response: We thank the reviewers for raising this important point. Previous studies have confirmed the association between high IL6 expression and poor prognosis in GBM patients through analysis of TCGA data[11]. We have further supplemented the description of IL-6 correlation in the results section and added supporting literature.

Based on the comments above, we have revised the manuscript at the appropriate place. The main text is as follows:
The engagement of CD99 and its ligand can upregulate the expression of IL-6 and TNF-α, which in turn promotes tumor cell proliferation and survival, ultimately correlating with poor clinical outcomes (61, 62). (Page 27, Lines 541-543)

Comment 7: In Results 5, pseudotime analysis identifies two distinct T-cell lineage trajectories. The authors are encouraged to elaborate on the biological significance of this bifurcation and discuss possible drivers.
Response: We thank the reviewer for this suggestion. The bifurcation observed in our pseudotime analysis represents two different trajectories of T cell exhaustion within the GBM microenvironment.
One trajectory (Naive T → Treg → CD8 T EX) represents an immunosuppressive differentiation route, where naive cells may transition through a regulatory state under the influence of suppressive cytokines (e.g. TGFβ) and eventually reaching terminal exhaustion. The other trajectory (Naive T → CD8 T EM → CD8 T EX) reflects a classic exhaustion pathway driven by chronic antigen stimulation, where effector memory cells progressively lose functional capacity. This indicates that T cell exhaustion is a convergent terminal state in the GBM microenvironment. We have expanded the Results section to demonstrate the biological significance of distinct T-lineage trajecories.

Based on the comments above, we have revised the manuscript at the appropriate place. The main text is as follows:
This bifurcation suggests that the exhaustion of T cells in GBM is a convergent process that can be achieved either through an immunosuppressive pathway or through the gradual decline of effector memory cell function. (Page 30, Lines 600-603)

Comment 8: In Results 6, the transcriptional regulatory networks were constructed for CD8_T_EM and CD8_T_EX subpopulations. Beyond their relatively high communication intensity with tumor cells, it remains unclear whether additional

biological or analytical considerations informed their selection. The criteria and rationale for focusing on these two subsets should be clearly articulated.
Response: We thank the reviewer for this comment. This study focuses on CD8_T_EM and CD8_T_EX cells not only because these two cell types exhibit strong cellular interactions, but also because these two subsets are involved in anti-tumor immune responses. In the GBM microenvironment, CD8+ T cells transition from an effector memory state to an exhaustion state, leading to a loss of immune killing function. Furthermore, pseudotime trajectory analysis shows that exhaustion is the endpoint of T cell differentiation in the tumor microenvironment. Therefore, researching these two cell types at critical differentiation stages from a transcriptional regulation perspective is crucial. We have updated the Results section.
Based on the comments above, we have revised the manuscript at the appropriate place. The main text is as follows:
To identify transcription factors that drive the expression of HMsig and immune checkpoint molecules, we performed SCENIC analysis on two tumor cell populations and two CD8 T cell subsets exhibiting strong cellular interactions. In addition, we focused specifically on CD8_T_EM and CD8_T_EX cells because they represent a key transition point in the T cell trajectory, where effector memory T cells with tumor-killing functions shift into exhausted T cells. This transition is highly relevant to the immune escape mechanisms in GBM. (Page 33, Lines 674-680)

Comment 9: In Results 7, the authors apply the CARD algorithm for spatial transcriptomic deconvolution to infer cell-type composition at the tissue level. A methodological justification should be provided, highlighting the advantages of CARD relative to other spatial deconvolution tools in the context of this dataset.
Response: We thank the reviewer for justifying our selection of the CARD algorithm. Unlike traditional reference-based methods, CARD utilizes a conditional autoregressive (CAR) model to resolve the inherent spatial information within tissue structures. In the context of GBM, the distribution of cell types is typically organized into specific microenvironmental structures. This spatial autoregressive prior can accurately estimate cell proportions by considering the similarity between neighboring spots, thus providing a more precise spatial characterization of the GBM microenvironment.

Comment 10: The spatial transcriptomic analysis reveals consistent colocalized expression of tumor-associated transcription factors SOX4 and SOX11 across samples, supporting their regulatory role in HMsig activation. The Discussion section should further explore the potential regulatory implications of this spatial co-expression pattern and its relevance to tumor progression.
Response: We thank the reviewer for this insightful comment. SOX4 and SOX11, as transcriptional factors of HMsig genes, are co-expressed in spatial tissues and play a crucial regulatory role in enhancing HMsig expression, further promoting the malignant progression of GBM. Additionally, other transcription factors, such as RUNX3, that regulate immune checkpoint genes, also contribute to the regulatory network in GBM microenvironment. We have revised the Discussion section to demonstrate the regulatory role of both tumor-associated transcription factors and T-cell-related transcription factors in the spatial co-expression patterns.

Based on the comments above, we have revised the manuscript at the appropriate place. The main text is as follows:
Spatial transcriptomic analysis further confirmed the co-localization of the HMsig genes with ICGs and their transcription factors (especially SOX4, SOX11, and RUNX3) in specific tissue regions, forming an immunosuppressive microenvironment. The co-expression of SOX4 and SOX11 suggests a spatial regulatory mechanism that enhances HMsig genes expression and drives tumor progression. RUNX3 upregulates the expression of immune checkpoint molecules LAG3 and PDCD1, further promoting the co-regulation represented by the “dual-brake” mechanism, thereby facilitating immune escape by tumor cells. This spatial organization driven by a coordinated transcriptional program provides a structural basis for the persistent treatment resistance in GBM. (Page 42, Lines 872-881)

Powered by Editorial Manager® and ProduXion Manager® from Aries Systems Corporation

Reviewer #3
Comment 1: The abstract mentions the pivotal roles of hallmarks of malignancy and cell cycle regulatory pathways in transcriptional regulation, yet fails to specify the exact mechanisms or functions. A concise description of these critical roles would enhance clarity and impact.
Response: We thank the reviewer for this constructive suggestion. We have revised the abstract to specify that these pathways are essential for promoting tumor cell proliferation and progression.

Based on the comments above, we have revised the manuscript at the appropriate place. The main text is as follows:
Hallmarks of malignancy and cell cycle regulatory pathways were consistently enriched across these modalities, promoting tumor cell proliferation and progression. (Page 1, Lines 15-17)

Comment 2: At present, the abstract appears disconnected without clear transitional logic; revision to establish explicit linkages between sentences is recommended.
Response: We thank the reviewer for this constructive suggestion. We have added linkages that connect the initial multi-scale data integration to the subsequent identification of core malignant pathways, the implementation of our machine learning framework, and the final spatial validation. These transitions emphasize how each analytical layer builds upon the biological findings of the previous stage, ensuring a more cohesive narrative that reflects our integrative methodology.

Based on the comments above, we have revised the manuscript at the appropriate place. The main text is as follows:
Glioblastoma (GBM) is characterized by pronounced tumor heterogeneity and a complex immune microenvironment, contributing to poor patient survival outcomes. In this study, we comprehensively dissected the tumor microenvironment (TME) and uncovered potential molecular mechanisms by integrating single-cell, bulk, and spatial transcriptomic data. Hallmarks of malignancy and cell cycle regulatory pathways were consistently enriched across these modalities, promoting tumor cell proliferation and progression. Using machine learning algorithm, we identified seven hallmark-related prognostic signatures (HMsig), namely AEBP1, ASF1A, PRPS1, DCC, OPHN1, IL13RA2, and HDAC5—whose predictive importance was validated through SHAP analysis. Ligand-receptor (LR) interaction analysis further revealed that interactions involving OPHN1 were associated with poorer prognosis. Along the pseudotime trajectory of T-cell differentiation, immune checkpoint genes (ICGs) LAG3, PDCD1, and HAVCR2 were substantially upregulated. Notably, synergistic transcriptional regulation between tumor-related HMsig genes and ICGs in T cells was identified as a key factor influencing patient survival. Spatial transcriptomic analysis demonstrated the existence of synergistic gene interactions, deciphering the immunomodulatory functions of GBM biomarkers within the TME. (Page 1-2, Lines 11-27)

Comment 3: The keywords should represent the core, recurrent themes throughout the entire study rather than terms mentioned only sporadically at the beginning or conclusion.
Response: We sincerely thank the reviewer for this constructive suggestion regarding the selection of keywords. We agree that keywords should summarize the core methods and biological themes that run through the entire study.

Based on the comments above, we have revised the manuscript at the appropriate place. The main text is as follows:
Glioblastoma; Single-cell transcriptomics; Spatial transcriptomics; Tumor microenvironment; Machine learning; Prognostic signature; Immune escape. (Page 1-2, Lines 28-29)

Powered by Editorial Manager® and ProduXion Manager® from Aries Systems Corporation

Comment 4: In the Introduction, the authors identify multiple factors contributing to the poor therapeutic outcomes in glioma, among which the 'characteristics of the tumor's physical microenvironment' is vaguely delineated and lacks conceptual precision.
Response: We sincerely thank the reviewer for this constructive suggestion. We further explored the impact of GBM physical microenvironment characteristics on poor treatment efficacy. The efficacy of most small molecule chemotherapy drugs is limited by the blood-brain barrier (BBB), which makes it difficult for drugs to penetrate the walls of brain blood vessels. This may be the main reason for the poor results in many clinical trials of blood-borne drugs. We have revised the Introduction section to specify biophysical characteristics in GBM.

Based on the comments above, we have revised the manuscript at the appropriate place. The main text is as follows:
Additionally, the efficacy of most small-molecule chemotherapy drugs is limited by the unique structure of brain blood vessels—the blood-brain barrier (BBB), which strictly regulates the homeostasis of the central nervous system. The inability of drugs to cross the walls of brain blood vessels is likely a major reason for the poor results in many clinical trials of blood-borne drugs (4). (Page 3, Line 41-52)

Comment 5: Within the subsection 'Immune phenotype of tumors based on transcriptomic profiling,' the initial two sentences comprise background material that is incongruous with the Methods section. The authors are advised to relocate this content to the Introduction to preserve the logical organization of the manuscript.
Response: We thank the reviewer for this constructive advice. We agree that the definitions of the immune “hot” and “cold” phenotypes are more appropriate for the introduction, as they provide necessary background information for the theoretical basis of our research. Therefore, we have moved these background definitions to the Introduction section and removed them from the Methods section.

Based on the comments above, we have revised the manuscript at the appropriate place. The main text is as follows:
Various studies support the hypothesis that GBM is an immunologically “cold” tumor(3). Unlike immune hot tumors, which show extensive immune infiltration and active anti-tumor immunity, cold tumors are defined by an absence of these responses and weakened immune defenses. (Page 3, Line 44-48)

Comment 6: In the Results section, certain findings lack corresponding textual description; for instance, Figure 1B is presented without adequate characterization in the narrative.
Response: We thank the reviewer for this observation. In response, we have added a description to the Results section specifying that the annotated cell clusters show highly specific expression of canonical lineage markers, providing robust molecular support for our cell type identification in the GBM microenvironment.

Based on the comments above, we have revised the manuscript at the appropriate place. The main text is as follows:
Canonical marker genes exhibited high expression specificity within their corresponding cell lineages, confirming the accuracy of cell type annotation (Figure 1B, Supplementary Table 2). (Page 16, Lines 304-306)

Comment 7: The Methods describe T cells and B cells as reference populations for inferring copy number variation, whereas the Results define highly malignant cells as those with above-average CNV scores within two neuronal subtypes. The former strategy is conventionally accepted; however, the latter lacks explicit methodological justification. Furthermore, the relationship between these two approaches—whether complementary or conflicting—requires detailed elucidation.
Response: We appreciate the valuable feedback from the reviewer. The method described in this paper identifies highly malignant cell populations in GBM based on the mean CNV score of all cells. The specific workflow is as follows: First, using T cells and B cells as reference normal cell populations, we select malignant cell lineages (including glia/neuronal cells and oligodendrocytes) as the observation set and calculate the CNV score of all cell populations. Second, we set the average CNV score as a threshold to divide the observation cell population into highly malignant and low-malignant subpopulations. This method enables more refined analysis of core hallmark

Powered by Editorial Manager® and ProduXion Manager® from Aries Systems Corporation

features in genomically unstable tumor cells. We have updated the methods section.

Based on the comments above, we have revised the manuscript at the appropriate place. The main text is as follows:
In the single-cell transcriptome data, we used T cells and B cells as the reference set to calculate the CNV score for all cells. Oligodendrocytes and glia/neuronal cells were used as sources of malignant cells in the observation set to identify highly malignant cells. We defined the two cell types with CNV scores exceeding the mean as highly malignant cells. (Page 8, Lines 151-155)

Comment 8: In Results section 2, the authors report that B-cell infiltration does not differ significantly between immune-hot and immune-cold tumors. Does this observation suggest that B cells may play a dual or context-dependent role within the GBM microenvironment? The authors are encouraged to provide further interpretation and discussion to clarify this point.
Response: We thank the reviewer for this valuable comment. The immune infiltration analysis shows no significant difference in B cell infiltration between immune hot and immune cold tumors. This suggests that B cells may play different functional roles in distinct microenvironments. Notably, the enrichment of B cells in immune cold microenvironments suggests that B cells may have an immunosuppressive function rather than promoting an active immune response. Previous studies have shown that B cells can directly suppress the activity of CD8+ T cells and recruit Tregs, thereby promoting tumor progression[12]. We have highlighted this point in the revised Results section.

Based on the comments above, we have revised the manuscript at the appropriate place. The main text is as follows:
Regarding the enrichment phenomenon of B cells in immune "cold" tumors, it may be associated with an immunosuppressive function(45, 46). This observation suggests that B cell activity in GBM is context dependent, potentially contributing to a pro-tumorigenic environment rather than an active anti-tumor response in specific TME. (Page 20-21, Lines 397-400)

Comment 9: The authors should explicitly define the 'combined score' depicted in Figure 4K, including its constituent variables and the algorithm or formula used for its calculation.
Response: We thank the reviewer for this valuable comment. The combined score shown in Figure 4K is the standard confidence metric utilized by the STRING database. The STRING database integrates different types of interaction evidence, assigns weights to each type, and finally calculates a combined score to evaluate the reliability of the protein-protein interaction. We have updated the legend for Figure 4K to concisely define this metric.

Based on the comments above, we have revised the manuscript at the appropriate place. The main text is as follows:
PPI networks between HMsig genes and LR pairs. Orange dots represent HMsig genes, blue dots represent LR pairs, and the color and thickness of the lines are determined by the combined score. The combined score is the standardized confidence metric to evaluate the PPI from the STRING database. (Page 29, Lines 586-589)

Comment 10: The T cell differentiation trajectory in Results section 5 appears poorly defined in two-dimensional space. Please provide additional annotation to clarify the inferred developmental path.
Response: We thank the reviewer for this suggestion. Figure 5A provides a three-dimensional developmental trajectory of T cells, with directional arrows illustrating the differentiation trend. Figure 5C utilizes continuous pseudo-time values to define the T cell differentiation trajectory in two-dimensional space. The transition from the dark purple root (representing naive T cells) to the yellow terminal state (representing CD8_T_EX cells) clearly presents the differentiation process. The black principal tree line in Figures 5B and 5C further marks specific branching points. We have updated the results section to clarify the specific differentiation trajectory paths.

Based on the comments above, we have revised the manuscript at the appropriate

Powered by Editorial Manager® and ProduXion Manager® from Aries Systems Corporation

place. The main text is as follows:
One trajectory commenced with naive T cells, passed through Treg cells, and ended at CD8_T_EX cells; the other started from naive T cells, proceeded through CD8_T_EM cells, and terminated at CD8_T_EX cells (Figure 5A, Supplementary Figure 3B). (Page 30, Lines 597-600)

Respectfully yours,
Conxue Hu, Ph.D, Yunpeng Zhang, Ph.D;
College of Bioinformatics Science and Technology
Harbin Medical University
194 Xuefu Road, Harbin 150081, China
Phone: 86-451-86615922
Fax: 86-451-86615922
Email: hucx1996@hrbmu.edu.cn, zhangyp@hrbmu.edu.cn;

References
1.Wang H, Wang X, Xu L, Zhang J, Cao H. Analysis of the Egfr Amplification and Cdkn2a Deletion Regulated Transcriptomic Signatures Reveals the Prognostic Significance of Spats2l in Patients with Glioma. Front Oncol. 2021;11:551160.
2.Sharpe MA, Baskin DS, Jenson AV, Baskin AM. Hijacking Sexual Immuno-Privilege in Gbm-an Immuno-Evasion Strategy. Int J Mol Sci. 2021;22(20).
3.Liu X, Gao Q, Zhao N, Zhang X, Cui W, Sun J, Fu J, Hao J. Sohlh1 Suppresses Glioblastoma Cell Proliferation, Migration, and Invasion by Inhibition of Wnt/Beta-Catenin Signaling. Mol Carcinog. 2018;57(4):494-502.
4.Shi Y, Sun Y, Cheng H, Wang C. Efnb1 Acts as a Novel Prognosis Marker in Glioblastoma through Bioinformatics Methods and Experimental Validation. J Oncol. 2021;2021:4701680.
5.Zheng Y, Shi J. Efnb1 Drives Glioma Progression and Shapes the Immune Microenvironment: A Potential Prognostic Biomarker. Discov Oncol. 2025;16(1):249.
6.Cho HJ, Hwang YS, Yoon J, Lee M, Lee HG, Daar IO. Ephrinb1 Promotes Cancer Cell Migration and Invasion through the Interaction with Rhogdi1. Oncogene. 2018;37(7):861-872.
7.Tran HTN, Ang KS, Chevrier M, Zhang X, Lee NYS, Goh M, Chen J. A Benchmark of Batch-Effect Correction Methods for Single-Cell Rna Sequencing Data. Genome Biol. 2020;21(1):12.
8.Venteicher AS, Tirosh I, Hebert C, Yizhak K, Neftel C, Filbin MG, Hovestadt V, Escalante LE, Shaw ML, Rodman C, Gillespie SM, Dionne D, Luo CC, Ravichandran H, Mylvaganam R, Mount C, Onozato ML, Nahed BV, Wakimoto H, Curry WT, Iafrate AJ, Rivera MN, Frosch MP, Golub TR, Brastianos PK, Getz G, Patel AP, Monje M, Cahill DP, Rozenblatt-Rosen O, Louis DN, Bernstein BE, Regev A, Suva ML. Decoupling Genetics, Lineages, and Microenvironment in Idh-Mutant Gliomas by Single-Cell Rna-Seq. Science. 2017;355(6332).
9.Tirosh I, Venteicher AS, Hebert C, Escalante LE, Patel AP, Yizhak K, Fisher JM, Rodman C, Mount C, Filbin MG, Neftel C, Desai N, Nyman J, Izar B, Luo CC, Francis JM, Patel AA, Onozato ML, Riggi N, Livak KJ, Gennert D, Satija R, Nahed BV, Curry WT, Martuza RL, Mylvaganam R, Iafrate AJ, Frosch MP, Golub TR, Rivera MN, Getz G, Rozenblatt-Rosen O, Cahill DP, Monje M, Bernstein BE, Louis DN, Regev A, Suva ML. Single-Cell Rna-Seq Supports a Developmental Hierarchy in Human Oligodendroglioma. Nature. 2016;539(7628):309-313.
10.Ahrends R, Ota A, Kovary KM, Kudo T, Park BO, Teruel MN. Controlling Low Rates

	of Cell Differentiation through Noise and Ultrahigh Feedback. Science. 2014;344(6190):1384-1389.<br>11.Lamano JB, Lamano JB, Li YD, DiDomenico JD, Choy W, Veliceasa D, Oyon DE, Fakurnejad S, Ampie L, Kesavabhotla K, Kaur R, Kaur G, Biyashev D, Unruh DJ, Horbinski CM, James CD, Parsa AT, Bloch O. Glioblastoma-Derived Il6 Induces Immunosuppressive Peripheral Myeloid Cell Pd-L1 and Promotes Tumor Growth. Clin Cancer Res. 2019;25(12):3643-3657.<br>12.Yang C, Lee H, Pal S, Jove V, Deng J, Zhang W, Hoon DS, Wakabayashi M, Forman S, Yu H. B Cells Promote Tumor Progression Via Stat3 Regulated-Angiogenesis. PLoS One. 2013;8(5):e64159.
Additional Information:	
**Question**	**Response**
Are you submitting this manuscript to a special series or article collection?	No
**Experimental design and statistics**<br><br>Full details of the experimental design and statistical methods used should be given in the Methods section, as detailed in our Minimum Standards Reporting Checklist. Information essential to interpreting the data presented should be made available in the figure legends.<br><br>Have you included all the information requested in your manuscript?	Yes
**Resources**<br><br>A description of all resources used, including antibodies, cell lines, animals and software tools, with enough information to allow them to be uniquely identified, should be included in the Methods section. Authors are strongly encouraged to cite Research Resource Identifiers (RRIDs) for antibodies, model organisms and tools, where possible.<br><br>Have you included the information requested as detailed in our Minimum Standards Reporting Checklist?	Yes
**Availability of data and materials**<br><br>All datasets and code on which the	Yes

conclusions of the paper rely must be either included in your submission or deposited in publicly available repositories (where available and ethically appropriate), referencing such data using a unique identifier in the references and in the "Availability of Data and Materials" section of your manuscript.<br><br>Have you have met the above requirement as detailed in our Minimum Standards Reporting Checklist?	
GigaScience has policies and guidelines in place for the use of generative AI-writing tools such as ChatGPT. If you have used such writing tools to assist with writing the manuscript this must be declared and cited in the text. Authors should not list AI-writing tools and other AI-assisted technologies as an author or co-author and should acknowledge that they are fully responsible for text generated or refined by AI-writing tools.<p><br><br>A summary of use (particularly in the introduction or among methods) needs to be included at the end of the paper, and the outputs should also be included as a supplementary file hosted in GigaDB or other open repositories. Please read our guidelines for more information. <p><br><br>By submitting to GigaScience, you are aware of the journal's AI-writing tools policy, and if you have declared use of such tools below, you have acknowledged this where appropriate in your manuscript and have made a summary of use and outputs available. <p><br><b>AI-assisted writing tools have been used in the preparation of this manuscript?	No

Manuscript

Click here to access/download;Manuscript;Manuscript-Revised with marks.docx

Dissecting glioblastoma risk signatures in the tumor immune microenvironment based on multi-dimensional transcriptomics

Tengyue Li[1] #, Mi wanqi[1] #, Huarui Yan[1], Yining Ma[1], Han Jiang[1], Xiaoxu Yang[1], Yunpeng Zhang[1]*, Congxue Hu[1]*

1College of Bioinformatics Science and Technology, Harbin Medical University, Harbin 150081, China

Lead contact: Congxue Hu: hucx1996@hrbmu.edu.cn

#These authors contributed equally to this work.

*Correspondence: Congxue Hu: hucx1996@hrbmu.edu.cn

ORCIDs: Tengyue Li [0000-0002-3724-7141], Mi wanqi [0000-0002-5263-1635], Huarui Yan [0009-0005-7066-4069], Yining Ma [0009-0004-1625-9380], Han Jiang [0009-0004-4312-6938], Xiaoxu Yang [0009-0004-5337-0766], Yunpeng Zhang [0000-0002-3709-3656], Congxue Hu [0009-0001-3279-9457]

Abstract

Glioblastoma (GBM) is characterized by pronounced tumor heterogeneity and a complex immune microenvironment, contributing to poor patient survival outcomes. In this study, we comprehensively dissected the tumor microenvironment (TME) and uncovered potential molecular mechanisms by integrating single-cell, bulk, and spatial transcriptomic data. Hallmarks of malignancy and cell cycle regulatory pathways were consistently enriched across these modalities, promoting tumor cell proliferation and progression. Using machine learning algorithm, we identified seven hallmark-related

prognostic signatures (HMsig), namely AEBP1, ASF1A, PRPS1, DCC, OPHN1, IL13RA2, and HDAC5—whose predictive importance was validated through SHAP analysis. Ligand-receptor (LR) interaction analysis further revealed that interactions involving OPHN1 were associated with poorer prognosis. Along the pseudotime trajectory of T-cell differentiation, immune checkpoint genes (ICGs) LAG3, PDCD1, and HAVCR2 were substantially upregulated. Notably, synergistic transcriptional regulation between tumor-related HMsig genes and ICGs in T cells was identified as a key factor influencing patient survival. Spatial transcriptomic analysis demonstrated the existence of synergistic gene interactions, deciphering the immunomodulatory functions of GBM biomarkers within the TME.

Keywords: Glioblastoma; Single-cell transcriptomics; Spatial transcriptomics; Tumor microenvironment; Machine learning; Prognostic signature; Immune escape.

Introduction

Being a high-grade glioma assigned a World Health Organization (WHO) Grade IV, GBM stands as one of the tumors that resist cure most stubbornly. Among primary brain cancers, GBM is the most aggressive and occurs most frequently, with a median survival time of only 14-18 months[1]. Currently, the recurrence rate of GBM patients after treatment exceeds 90%. Only a negligible number of patients are likely to approach a state of cure. It's important to stress that the outlook for GBM patients is extremely poor. As a result, gaining a deeper understanding of the diversity of the pathological biology of GBM and exploring its potential TME characteristics is paramount.

Unsatisfactory therapeutic outcomes for GBM may be multifactorial, including the decline of T cell killing function, defects in tumor antigen presentation, and characteristics of the tumor's physical microenvironment. In brain tumors, there are more resident microglia and macrophages compared to infiltrating T cells[2]. Various studies support the hypothesis that GBM is an immunologically "cold" tumor[3]. Unlike immune hot tumors, which show extensive immune infiltration and active anti-tumor immunity, cold tumors are defined by an absence of these responses and weakened immune defenses. Additionally, the efficacy of most small-molecule chemotherapy drugs is limited by the unique structure of brain blood vessels—the blood-brain barrier (BBB), which strictly regulates the homeostasis of the central nervous system. The inability of drugs to cross the walls of brain blood vessels is likely a major reason for the poor results in many clinical trials of blood-borne drugs[4]. Moreover, the scarcity

of T cells in the TME starkly contrasts with findings in melanoma, lung cancer, and other tumor types[5]. Whether GBM is inherently non-immunogenic and the regulatory mechanisms between tumor cells and immune cells in the TME require further investigation.

The significant heterogeneity observed in GBM is largely driven by its regulation of core hallmarks. More precisely, the control of GBM cell proliferation, self-renewal, and the inactivation of differentiation is mediated by the Wnt, Notch, and TGF-β signaling pathways[6]. The WNT/β-catenin cascade is closely associated with various malignant tumors. In normal cells, the Wnt signaling pathway is typically in an inactive state and is highly conserved[7]. Abnormal activation of the Wnt signaling promotes cancer cells to escape immune surveillance, inhibits T cell infiltration, and mediates the anti-tumor immune response[8]. This pathway has recently become an important determinant in the occurrence and development of GBM. GBM is also involved in alterations of multiple metabolic hallmarks, such as glycolysis, oxidative phosphorylation, and hypoxia[2]. GBM utilizes various unconventional molecules to sustain its growth as well. Hypoxia stimulates adenosine monophosphate-activated protein kinase (AMPK), which in turn regulates the energy acquisition of tumor cells[9]. Additionally, hallmarks related to transcriptional regulation are also involved in the development of tumor tissues. E2F7, a member of the E2F family transcription factors (E2Fs), not only participates in cell cycle regulation as a transcriptional repressor[10, 11], but also contributes to tumor cell proliferation and metastasis. Besides, studies have found that E2F7 may be involved in mediating immune cell infiltration[12, 13]. Thus,

transcriptional regulation affects not only tumor cells themselves but also tumor progression by modulating the cellular and molecular components in the TME[14]. In published GBM related studies, the synergistic action of TFs is often observed within the same cell type[11, 15, 16]. However, the transcriptional regulatory mechanisms between TFs across different cell clusters remain unclear.

GBM diffusely infiltrates the brain, intermingling with non-neoplastic brain cells. This intricate TME forms the biological basis for treatment response and tumor recurrence. It is of utmost importance to delve deeply into the interactions between GBM cells and their immune microenvironment. However, the knowledge in this regard is currently insufficient. The enhanced resolution of single-cell sequencing can be utilized to uncover the key characteristics of highly heterogeneous tumors. Accordingly, we integrated transcriptome data from both bulk and single-cell level. We screen for biomarkers and identify risk genes in GBM by employing machine learning techniques like stepwise Cox regression (StepCox) and random forests (RSF). Meanwhile, we utilize multi-omics analysis to identify TF-Gene activity regulation networks and explore the transcriptional regulatory mechanisms between tumor cells and T cells.

Methods

Data collection

The multi-dimensional transcriptomic datasets used in this study, including single-cell, bulk, and spatial data, are summarized in Supplementary Table 1 (detailed accession numbers and repository identifiers are provided in the "Data availability" section). For the single-cell RNA-seq analysis, we utilized 15 primary samples derived from 9 patients with wild-type GBM to characterize the tumor microenvironment. For the bulk transcriptome analysis, a total of 769 primary GBM samples with survival information were collected and divided into training and validation cohorts. The training cohort consisted of 428 primary GBM samples obtained from The Cancer Genome Atlas (TCGA) and the Chinese Glioma Genome Atlas (CGGA) databases. For independent validation of prognostic markers, an additional 341 microarray-based disease samples were acquired from four independent Gene Expression Omnibus (GEO) cohorts. Additionally, 105 normal brain samples from the Genotype-Tissue Expression (GTEx) database were utilized as non-tumor references for subsequent analysis. Finally, for the spatial transcriptomics profiling, 12 primary wild-type GBM samples were used to analyze the spatial cellular architecture.

Data preprocessing

For single-cell level data, cells expressing fewer than 200 genes or more than 2,500 genes were filtered out, and cells with mitochondrial gene content exceeding 20% were removed to ensure the quality of cells used in downstream analysis. After quality

control filtering, 71,836 cells remained. A gene was retained if it was expressed in at least 3 cells. Following the removal of unqualified genes, 29,622 genes were retained for analysis. For bulk level data, data from the TCGA and CGGA platforms were converted into transcripts per million (TPM). The Combat algorithm (sva R package, version 3.35.2) was used to remove batch effects from the bulk RNA-seq expression profiles of the TCGA and CGGA platforms. For cases where multiple ENSEMBL ids map to the same gene, we calculate the average expression level of each gene using "rowMeans" and select the gene with the highest expression level. The log2(x+1) transformation were performed to generate clean microarray data. For spatial transcriptomics (ST) data, we loaded raw 10x Visium data including gene expression matrixs, spatial coordinates, and tissue images using the "Load10X_Spatial" function, followed by data normalization and variance stabilization with "SCTransform" while retaining all genes for downstream analysis.

Dimensionality reduction and clustering of cells

The preprocessed gene expression matrix and cell annotation information were processed using the Seurat R package (version 5.1.0). The top 2,000 highly variable genes, identified using the standard deviation (SD) algorithm, were used for principal component analysis (PCA). Expression profiles were normalized using the LogNormalize method (feature counts per cell divided by the total counts for that cell, multiplied by a scale factor). To maximize the explanation of data variability with the fewest principal components, the top 16 principal components were manually selected for cell clustering analysis using the UMAP algorithm. The Harmony algorithm[17]

(version 1.2.1) was implemented to mitigate batch effects among tumor samples during data integration. To ensure the stability and of the clustering results, we evaluated various resolution parameters using the clustree package (version 0.5.1). A resolution of 0.2 was applied to identify major cell lineages. For the subclustering of specific populations, we utilized resolutions of 0.9 for T cells and 0.8 for myeloid cells to resolve fine grained cell subtypes. Cell clusters were defined using marker genes specific to cell types in GBM tissues, collected from published literature and the CellMarker2.0 database[18]. The complete set of markers used for cell type annotation, along with their corresponding clustering resolutions, is systematically documented (Supplementary Table 2).

Identification of malignant tumor cells

The inferCNV algorithm[19] (version 1.10.1) was used to infer copy number states by calculating the ratio of gene expression levels in tumor cells to the average expression levels in reference normal cell populations. This methodology is particularly robust for GBM as it was fundamentally established and validated through foundational single cell research in brain tumors[20-22]. In the single-cell transcriptome data, we used T cells and B cells as the reference set to calculate the CNV score for all cells. Oligodendrocytes and glia/neuronal cells were used as sources of malignant cells in the observation set to identify highly malignant cells. We defined the two cell types with CNV scores exceeding the mean as highly malignant cells. All cell clusters except myeloid cells were used to assess chromosomal copy number variations, including

amplifications and deletions. The minimum average read count threshold for each gene in the reference cells of 10X genomics data is set to 0.1.

Immune phenotype of tumors based on transcriptomic profiling

We selected the top 50 DEGs from immune cell subsets in single-cell transcriptome data to construct an immune cell gene set. The ssGSEA algorithm was used to calculate the immune gene set enrichment score (GSES) for each cell to estimate the infiltration degree of different immune cell types in tumor samples. Tumor samples were clustered using ConsensusClusterPlus (version 1.68.0) based on GSES. The tumor samples were divided with K=2, and the tumor samples with significantly enriched immune cells were defined as immune "hot" tumors. The GSES was normalized by the “scale” function, and half-violin plots and heatmaps were generated to visualize the enrichment results before and after scaling. Immune scores were calculated using “Estimate” (version 1.0.13) to validate the ssGSEA results.

Gene set enrichment analysis (GSEA)

We used the fgsea (version 1.13.0) R package to test the enrichment of hallmark genesets downloaded from MsigDB (msigdbr R package version 7.5.1). For single-cell level, the Wilcoxon rank sum test (presto R package version 1.0.0) was used to calculate the DEGs and the AUC value was used as the ranking index to generate the pre-sorted list of genes. For bulk-level input, a pre-ranked gene list generated from differential expression analysis using the limma R package (version 3.60.2) was applied. Genes with P-value<0.05 and $|logFC|>1$ were set as significant DEGs. The GSEA was used

for 1000 permutation tests, and NES>0 and P.adj.value<0.05 were defined as significant up-regulation of hallmark.

Machine learning model construction and validation

To construct a model for predicting the prognosis of GBM patients, we first divided all bulk tumor samples into training and test sets at a ratio of 7:3. Then, we performed univariate Cox regression and Kaplan-Meier (KM) survival analysis on the 354 GBM progression-related feature genes identified across multiple dimensions. Only genes that showed statistically significant prognostic relevance ($P < 0.05$) in both tests were retained, resulting in 42 high-value candidate genes. These pre-screened genes were input into an integrated machine learning framework built using the R package mime1[23].

We constructed and evaluated the model on both the training and test sets using 10-fold cross-validation, selecting the combined model with the highest C-index as the optimal model. Based on this optimal model, we further performed feature screening, ultimately identifying 7 prognostic hallmark characteristic genes (HMsig). To comprehensively evaluate the model, we performed a meta-analysis integrating the training and test sets, and conducted ROC analyses on 1-year, 3-year, and 5-year survival predictions for GBM patients. Model performance was compared with existing methods using C-index and ROC values. Furthermore, we collected three external validation sets from the GEO database to validate the model's performance in predicting patient outcomes (Supplementary Table 1).

HMsig importance assessment

We employed the SHAP algorithm (fastshap R package version 0.1.1) to quantitatively assess the prognostic influence of HMsig genes. A survival analysis model was constructed through a combined machine learning algorithm model of forward stepwise Cox regression and random forest. Then, the “explain” function was used to calculate the SHAP value for HMsig genes of each sample to measure their impact on the survival prediction results of patients. Visualize the importance of features through the “sv_importance” function.

Cell communication analysis

Cell communication analysis was performed by CellChat[24] (version 1.6.1). We employed the established CellChat method to investigate interactions between tumor cells and immune cells. CellChatDB integrates signaling interaction information from the KEGG pathway database and literature from experimental studies. The human LR database from CellChatDB was used as a reference to evaluate cell communication networks between two cell types. DEGs across all cell clusters were identified using the Wilcoxon rank-sum test (Pvalue<0.05). To account for noise effects, the triMean quartile method was used to calculate the average expression of LR pairs in cell clusters.

Construction of HMsig-LR interaction network

The Search Tool for the Retrieval of Interacting Genes/Proteins (STRING) database[25] enables the exploration of potential interactions between genes, constructing interaction networks that illustrate relationships such as physical contacts

or regulatory targeting among HMsig and LR pairs. The genes derived from HMsig and ligand–receptor (LR) pairs identified using CellChat were submitted to the STRING database to identify potential gene interaction networks. Subsequently, the gene interaction network was constructed and visualized using Cytoscape[26] (version 3.9.1).

T cell developmental trajectory

Based on previous studies defining various T cell characteristics, we analyzed T cells and identified four T cell subtypes, which are: Naive T cells, Tregs (regulatory T cells), and CD8_T_EM (CD8+ T effector memory) cells and CD8_T_EX (CD8+ T exhausted) cells. Pseudotime trajectories for these T cell types were constructed using Monocle3[27] (version 1.3.3), a semi-supervised pseudotime analysis algorithm approximating PAGA (Partition-based graph abstraction). Dimensionality reduction was performed using the UMAP algorithm via the "reduceDimension" function. We used 'get_earliest_principal_node' function to identify the earliest principal node from Naive T cells, which is a helper function provided by monocle3. This node was then designated as the root node using the 'order_cells' function to calculate pseudotime values for all cells and infer the differentiation trajectory. Temporal distribution density curves for the T cell subtypes were visualized using the ggridges R package (version 0.5.6).

Gene ontology enrichment analysis (GO)

GO enrichment analysis was performed using clusterProfiler[28] (version 4.12.0) and org.Hs.eg.db (version 3.19.1). For the pseudotime developmental modules of T

cells, GO enrichment analysis was performed based on significantly DEGs within each module. Regarding the transcriptional regulatory sub networks of tumor cells and T cells, GO enrichment analysis was conducted on genes driven by cell type-specific TFs.

Construction of transcriptional regulatory networks

We used SCENIC[29] package (version 1.3.1) to identify regulons in single-cell transcriptomes. Single-cell datasets from two tumor cell types, CD8_T_EM, and CD8_T_EX cells were used as input. The GENIE3 algorithm was employed to construct regulatory networks based on motifs and ranked binding sites from RcisTarget. Regulon activity was calculated using AUCell. The regulon specificity score (RSS) for TFs in each cell type was computed using the "calcRSS" function (zThreshold = 0.1, thr = 0.1), to identify cell type-specific TFs. A binary matrix heatmap was generated, and TF-gene regulatory networks were constructed using Cytoscape (version 3.9.1). TF pairs with $r > 0.2$ and $p < 0.05$ were considered significantly positively correlated, indicating synergistic interactions. The "viewMotifs" function was used to examine motifs corresponding to TF-HMsig/ICG pairs, exploring the mechanisms of TF synergy.

Survival analysis

Survival analysis was conducted using the Survival (version 3.6.4) and survminer (version 0.4.9) R packages. For survival analysis of HMsig validated in GEO data, the StepCox(forward)+RSF model was used to calculate risk scores for each patient. Patients were stratified into high and low risk groups based on the median risk score,

and differences in clinical outcomes were assessed using the Log-rank test. Kaplan-Meier curves were used to visualize survival characteristics.

To investigate the impact of HMsig ligand-receptor gene interactions on GBM patient survival, we specifically evaluated the prognostic value of OPHN1-EFNB1 interaction patterns. Accounting for gene-gene interaction effects, we calculated the product of both genes' expression values and the optimal cutoff threshold for stratifying high versus low expression groups was determined using the "surv_cutpoint" function. Differences in clinical outcomes were assessed using the Log-rank test, and Kaplan-Meier curves were generated to visualize survival characteristics.

For survival analysis of synergistic TF pairs in TCGA-CGGA, the mean expression sum of each TF pair was used as the threshold to stratify patients into high and low-expression groups. Differences in clinical outcomes were assessed using the Log-rank test, and Kaplan-Meier curves were generated to visualize survival characteristics.

Cell type deconvolution

The cell-type compositions for each spot were determined using the conditional autoregressive deconvolution (CARD) algorithm[30] (version 2.2.0). CARD object was constructed from the ST data along with the scRNA annotated data described above to reference single-cell RNA sequencing (scRNA-seq) profiles encompassing 7 cell types. We applied "CARD.imputation" function to improve CARD-based expression, enabling high-resolution mapping of both cell-type localization and imputed gene

activity. Optimized ST data were chosen to reveal cellular distributions and gene expression patterns.

Statistical analysis

The results of the statistical analysis are presented using box plots and violin plots. The statistical significance of differences between groups was determined using the unpaired, two-tailed student t-test, with $P < 0.05$ considered statistically significant. The correlation analysis of TFs was performed using Spearman's correlation coefficient. These statistical analyses were performed using R software 4.4.1.

Result

Result 1 Identifying molecular hallmarks for malignant cells

After rigorous quality control and normalization, the gene expression profiles of 71,836 cells from 9 GBM patients were used for in-depth analysis. We employed the Uniform Manifold Approximation and Projection (UMAP) clustering algorithm, with which all cells were annotated into 7 distinct clusters (Figure 1A, Supplementary Figure 1A). These clusters included B cells, endothelial cells, glial and neuronal cells, myeloid cells, oligodendrocytes, pericytes, and T cells. Notably, the clusters of glial and neuronal cells and myeloid cells accounted for a much larger proportion of the total cell population than B cells and T cells in GBM patients. Moreover, in certain patients, such as MDAG-1 and MDAG-7, a relatively low level of immune cell infiltration was observed, which prominently emphasizes the momentous individual heterogeneity within GBM tissues. Canonical marker genes exhibited high expression specificity within their corresponding cell lineages, confirming the accuracy of cell type annotation (Figure 1B, Supplementary Table 2).

To accurately distinguish the malignancy level of tumor cells, the inferCNV algorithm was utilized to compute the copy number variation based on scRNA-seq data (Figure 1C). Since glial and neuronal cells are recognized as the main origin of malignant GBM cells, those two types of nerve cells with a CNV score exceeding the mean were defined as high malignant. Conversely, 4,829 cells were designated as low malignant according to their CNV scores. CNVs were also detected in pericytes and endothelial cells (Supplementary Figure 1D). Subsequently, all high malignant and low

malignant tumor cells were extracted and subjected to UMAP algorithm once again to visualize the distribution of tumor cells (Figure 1D).

In order to delve deeper into the unique molecular hallmarks of malignant cells, a differential expression analysis was carried out between the high malignant and low malignant tumor cells. This analysis disclosed that several genes, such as IGFBP2 and VBP1, were significantly upregulated (Figure 1E). IGFBP2 promotes glioma progression by activating the PI3K/AKT pathway and MMP2[31], while VBP1 regulates hypoxia responses by degrading HIF-1α, a key transcriptional regulator[32]. GSEA results revealed that the markedly enriched hallmark pathways encompassed the KRAS signaling pathway, the NOTCH signaling pathway, oxidative phosphorylation (OXPHOS), spermatogenesis along with an additional 24 markedly up-regulated hallmarks (Figure 1F). In GBM, normal cells mainly rely on aerobic oxidation of glucose to generate energy. However, tumor cells, in addition to enhanced glycolysis, also utilize the OXPHOS to produce more adenosine triphosphate (ATP), providing ample energy for their abnormal biological behaviors. Beyond the tumor-related metabolic pathways, numerous studies have demonstrated that the Notch signaling pathway, which is associated with cell proliferation, is over-active in GBM[33]. The diversity of these hallmarks implies the complex TME of GBM. Interestingly, the enrichment of the spermatogenesis pathway in GBM suggests that some genes involved in spermatogenesis may play a role beyond their traditional functions in reproductive tissues. Previous studies have indicated that abnormal expression of spermatogenesis-related genes is associated with glioma progression and malignancy[34]. In addition,

research has shown that GBM may exploit immune privilege mechanisms by expressing reproductive antigens, which could recruit Tregs activated in the reproductive system[35]. Notably, the spermatogenesis gene Sohlh1 has been shown to inhibit GBM cell proliferation and migration by modulating the Wnt/β-catenin signaling pathway[36], further supporting the essential roles of spermatogenesis-related genes in GBM progression.

Fig. 1. Single-cell transcriptome analysis of human glioma malignant cells. A UMAP projections of 71,836 aggregate single cells from 9 patients showing the composition of different cell types in human gliomas (upper panel). The stacked bar plot illustrates the proportion of each cell type across the 9 patients (lower panel). The colors of the dots and bars represent different cell types. B Dotplot shows the expression of marker genes in different cell types in GBM. Dot size indicates the proportion of expressing cells, colored by average expression levels. C Heatmap shows the normal/malignant cell status determined by inferCNV analysis. It displays the relative expression intensity of each cell across various chromosomal regions, with colors representing CNV levels. The upper heatmap represents the results for the reference cells, while the lower heatmap represents the results for the observation cells. D UMAP projections are shown by malignant status. E Differential expression analysis of tumor malignant cells. The size and color gamut of the dots are determined by the -log10(padj) values. F GSEA analysis of tumor malignant cells. The size and color of the dots are determined by the normalized enrichment score (NES) values. The padj_score is derived by -log10(padj).

Alt text: Graphs and data on single-cell transcriptome analysis of human GBM cells, with subfigures labelled from A to F, illustrating UMAP projections, gene expression profiles, normal and malignant cell classification, and hallmark-related GSEA enrichment results.

Result 2 Dissecting immune hot and cold tumors in GBM through single-cell and Bulk transcriptomics

In GBM, the microenvironment is typically characterized as "cold" due to limited immune infiltration[37]. The study found that the immune microenvironment significantly influences patient prognosis[38]. To systematically dissect these features and their clinical implications, we classified tumors into immune "cold" and "hot" states at the bulk level, integrating single-cell data to resolve limitations of single-modality analysis. We initially conducted subtype identification on T cells and myeloid cells (Supplementary Figure 1B-C). T cells were categorized into naive T cells, regulatory T cells (Treg), CD8 T EM, and CD8 T EX, whereas myeloid cells were identified as macrophages, dendritic cells, and neutrophils (Supplementary Figure 1E-H). Based on the results of single-sample gene set enrichment analysis (ssGSEA) of the top 50 differentially expressed genes in 8 immune cell clusters at the single-cell level, we conducted consensus clustering analysis. In that way, all GBM samples were divided into k (k=2–9) clusters. The cumulative distribution function (CDF) curve of the consensus score matrix and the delta area plot indicated that the optimal number was obtained when k=2 (Figure 2A-B, Supplementary Figure 1I). The two clusters (Cluster 1 and Cluster 2) exhibited clear differences in immune infiltration, in which B cells, Treg cells, CD8_T_EM cells, and other immune cells exhibit pronounced

enrichment in the tumor samples of Cluster 2. Undoubtedly, the immune infiltration abundance in Cluster 2 was remarkably higher compared to Cluster 1 (Figure 2C-D). Therefore, we defined Cluster 1 as an immune "cold" tumor and Cluster 2 as an immune "hot" tumor. We noticed neutrophils are significantly enriched in immune-hot tumors. Neutrophils exert anti-tumor effects by directly killing tumor cells through ROS, NO, and granular protein release[39-43]. This implies neutrophil enrichment helps differentiate immune "cold" from immune "hot" states.

We also explored the distribution of patient gender, age, and survival status within immune "cold" and "hot" tumor samples. It is undeniable that the number of immune "hot" tumor samples is markedly less than that of immune "cold" tumors. Among GBM patients, the proportion of patients in the survival state was significantly lower than that of deceased patients. Considering the overall GBM patient population, the prognosis of male patients is generally worse than that of female patients[44]. Nevertheless, upon closer examination of the immune "hot" tumor cluster, an intriguing phenomenon emerged. Among the living patients under 60 years old, all were male. This finding implies that within a specific GBM tumor immune subtype and age range, males may possess some undiscovered survival advantages, which deserves further research (Figure 2C). When additionally observing the relationship between immune cell infiltration and the TME, we noticed the special behavior of B cells in immune "cold" tumors. Regarding the enrichment phenomenon of B cells in immune "cold" tumors, it may be associated with an immunosuppressive function[45, 46]. This observation suggests that B cell activity in GBM is context dependent, potentially contributing to a

pro-tumorigenic environment rather than an active anti-tumor response in specific TME. We employed the ESTIMATE algorithm to verify the analysis results, which corroborated the outcomes of our previous ssGSEA (Figure 2E).

We performed differential expression analysis on immune “cold” tumors and immune “hot” tumors, in order to deepen our understanding of the molecular characteristics of GBM hallmarks (Figure 2F). CCL20 and CXCL5, as chemokines, could attract immune cells (such as T cells and monocytes) into the TME. Their downregulation implies a weakened ability to recruit immune cells, leading to insufficient infiltration of immune cells in tumor tissues and thus giving rise to the “cold” tumor phenotype. Simultaneously, the GSEA result revealed that six significantly upregulated pathways, including DNA repair, Myc-targets-v1, KRAS signaling pathway, etc (Figure 2G). The IL2_STAT5, IL6_JAK_STAT3, and inflammatory response hallmarks are remarkably downregulated in immune "cold" tumors (Figure 2G). In the TME, the JAK-STAT signaling pathway is essential for maintaining the homeostasis of immune cells. STAT5 and STAT3, as dominant TFs in this pathway, can influence genes related to cell proliferation, apoptosis, and immune responses. STAT5 acts as a catalyst for promoting the proliferation of T cells; STAT3 is indispensable for maintaining the cell identity of macrophages[47]. The downregulation of their related pathways leads to the decline of immune killing functions and spurs the generation of an immunosuppressive microenvironment. There were five common hallmarks across both data modalities, namely the E2F transcription factor family, G2/M checkpoint, spindle mitosis, Wnt signaling pathway, and spermatogenesis. A recent pan-cancer

study revealed that the androgen receptor (AR) is overexpressed in GBM[48]. In vitro experiments using anti-androgen drugs resulted in the inhibition of GBM cell proliferation[49, 50]. Spermatogenesis, a process regulated by androgens and highly active in the male reproductive system, showed significant enrichment in both datasets, suggesting that male patients may have unique susceptibilities or therapeutic responses in certain molecular mechanisms. Research has demonstrated that in high-grade gliomas, there is an enrichment of MYC_TARGETS_V1, G2M checkpoint, and E2F target hallmarks[51]. It is worth mentioning that these five pathways are significant in transcriptional regulation and are also involved in cell cycle regulation. Cancer cells utilize cell cycle checkpoints to delay mitosis and repair DNA damage. In particular, the G2/M phase of the cell cycle carried great weight in DNA repair[52]. The E2F family directly regulates the transcription of genes involved in DNA replication and cell cycle progression[53], further highlighting its transcriptional regulatory functions.

Fig. 2. Depicting the immune "cold" and "hot" status of GBM. A The consensus score matrix of GBM samples when k=2. B The CDF curves of consensus matrix for each k (showed by colors). C Heatmap shows the infiltration abundance of 8 immune cell types evaluated by ssGSEA for two clusters. The distribution of patient categories, gender, age, and survival status can be seen at the top of the heatmap. D The violin plot shows distribution of 8 immune cell types infiltration between two clusters. The red color represents the cluster of immune "cold" tumor and blue represents the cluster of immune "hot" tumor. The significance of differences between the two clusters is indicated by "***". E The boxplot shows the distribution of immune score inferred by ESTIMATE algorithm between two clusters. F Differential expression analysis of immune "cold" tumors. The size and

color gamut of the dots are determined by the -log10(P.value) values. We set |log2FC|≥0.5 and pvalue≤0.05 as the thresholds for screening DEGs. G GSEA analysis of immune "cold" tumors. The size and color of the dots are determined by the NES (Normalized Enrichment Score) values. The padj_score is derived by -log10(padj).

Alt text: Graphs and data on bulk transcriptomic analysis of immune "cold" and "hot" status in GBM, with subfigures labelled from A to G, illustrating consensus clustering analyses, immune cell infiltration profiles, differential gene expression, and hallmark-related GSEA enrichment results.

Result 3 Establishment and validation of hallmark-related prognostic signatures in glioblastoma

To establish a GBM risk prediction model, we used a random combination of 11 machine learning algorithms[23] and integrated the previously analyzed hallmarks based on bulk-level GBM transcriptional data with survival information. We used a multi-dimensional intersection strategy to identify hallmark-related feature set for prognostic modeling. This approach integrates three key aspects: the intrinsic malignant characteristics of GBM tumor cells captured by single-cell RNA-seq hallmark genes, the immune cold environment features reflected by bulk tumor hallmark genes, and the overall transcriptomic expression universality across patient populations represented by bulk DEGs. By focusing on the overlap of these gene sets, we prioritized genes that are critical not only for the malignant progression of GBM cells but also for shaping the immunosuppressive environment (Figure 3A). In total, 352 immune-related tumor hallmark genes at the combined single-cell and bulk levels were obtained. These features were first filtered using univariate Cox regression and KM survival analysis,

which identified 42 high-value prognostic candidate genes. Subsequently, these 42 genes were input into our integrated machine learning framework to identify the most essential predictors. The combination of StepCox (forward) and RSF was identified as the optimal model, as it achieved the highest C-index in the validation datasets (Figure 3B, Supplementary Figure 2A). Among the evaluated algorithms, the StepCox (forward) model was utilized to achieve an optimal balance between predictive power and model parsimony by effectively eliminating redundant variables, ultimately pinpointing 7 core hallmark-related prognostic signatures (HMsig): AEBP1, ASF1A, PRPS1, DCC, OPHN1, IL13RA2, and HDAC5. Survival analysis indicated that the HMsig could effectively distinguish patients with different risk levels, with AUC values for 1-, 3-, and 5-year survival exceeding 0.85 in both sets (Figure 3C-D, Supplementary Figure 2B).

Meta-analysis further suggested that the risk score calculated by the StepCox (forward) and RSF model was a risk factor for GBM (Supplementary Figure 2E-F). We then tallied the frequency with which these 7 genes were selected as core features across different algorithms (Figure 3E, Supplementary Figure 2C). By comparing our obtained optimal model with existing GBM risk models constructed from different literature and calculating the C-index and AUC values, our optimized model ranked first (Figure 3F-I), demonstrating superior predictive stability.

Concerning the biological basis of HMsig, recent studies have found that ASF1A was identified as a biomarker for malignant diseases such as lung adenocarcinoma and hepatocellular carcinoma[54-56]. However, no studies have yet reported that ASF1A and

OPHN1 can be a risk gene for GBM. ASF1A regulates the expression of cell cycle-related genes and influences glial cell differentiation[57]. OPHN1 not only participates in cell cycle regulation[58, 59] but has also been found to promote tumor progression when overexpressed[60]. To further elucidate the impact of these seven genes on patient survival prediction, we employed the SHAP algorithm to calculate SHAP values based on risk scores derived from the StepCox (forward) and RSF combined model (Supplementary Figure 2D). SHAP analysis revealed that the AEBP1, ASF1A, and PRPS1 genes had the highest SHAP values, indicating their significant impact on survival prediction. These SHAP values suggest that these three genes are major predictors in HMsig, and their direction is consistently positively correlated with patient risk. This confirms that high expression of these HMsig genes is a key factor leading to elevated risk scores and indicates their role as high-risk determinants in the GBM microenvironment. Additionally, several independent validation datasets from GEO datasets confirmed that HMsig effectively distinguished high-risk from low-risk patients, demonstrating robust validation and strong generalizability (Figure 3J-L).

Fig. 3. Identifcation of potential GBM features in GBM. A An Overview of features selection workflow for patient tumors. The upper part shows the feature selection strategy at the single-cell level; the lower part shows the feature selection strategy at the bulk level; the volcano plot on the far right represents the differential analysis between tumor and normal samples at the bulk level. B Combined machine learning model framework of Mime algorithm. The combination highlighted in red represents our optimal model. C Kaplan–Meier curves of OS according to the StepCox(forward)+RSF in test set. D Time-dependent ROC analysis for predicting OS at 1, 3, and

5 years. The specific ROC values are labeled in the figure. E Selection frequency of HMsig in various machine learning algorithms and their enriched hallmarks. The size of the dots corresponds to the frequency at which the features are selected. F-I C-index and ROC analysis comparing our optimized model with published GBM signatures in the TCGA-CGGA cohort. The dashed line corresponding to the red scale represents the C-index/AUC value of our optimized model. J-L Kaplan–Meier curves of OS based on the StepCox(forward)+RSF model in an additional GEO validation set.

Alt text: Graphs and data on machine learning-based identification of prognostic features in GBM, with subfigures labelled from A to L, illustrating feature selection workflows, machine learning frameworks, Kaplan-Meier survival curves, model performance comparisons, and external cohort validation.

Result 4 Revealing immune regulatory mechanisms in TME through cell communication analysis

The TME is not merely an aggregation of cancer cells but rather a complex ecosystem composed of multiple cell types. These cells interact with one another via the secretion of factors, cytokines, and cell-to-cell contacts, jointly affecting the growth, invasion, and metastasis of tumors. For the purpose of deeply profiling the TME of GBM, the CellChat algorithm was applied to examine the communication between tumor cells and T cells, B cells, and myeloid cells. CD8_T_EM and CD8_T_EX cells engaged in intense interactions with tumor cells, myeloid cells, and other T cells (Figure 4A). Apparently, the two types of tumor cells have the strongest interaction with immune cells in macrophage migration inhibitory factor (MIF) signaling pathway,

followed by pleiotrophin (PTN) signaling pathway. CD8_T_EX cells account for a relatively large proportion in the communication intensity with the two types of tumor cells in the MIF, major histocompatibility complex class I (MHC-I), and PTN signaling pathways (Figure 4B).

Thereafter, we conducted a more detailed examination of the expression of LR pairs. MIF−(CD74+CXCR4) mediated firm interactions between tumor cells and T cells (Figure 4C-D). In GBM, this interaction may lead to the suppression of immune cell activity. Tumor cells can inhibit macrophage activity through the MIF-(CD74-CXCR4)[61]. The interaction of CD99-CD99 was also robust between immune cells and tumor cells (Figure 4C). The engagement of CD99 and its ligand can upregulate the expression of IL-6 and TNF-α, which in turn promotes tumor cell proliferation and survival, ultimately correlating with poor clinical outcomes[62, 63]. The interaction mediated by SPP1-CD44 between myeloid cells and other immune cells was remarkably strong (Figure 4E-F). SPP1-CD44 inhibits T cell infiltration, reducing the number of T cells in tumor tissues and exacerbating tumor immune evasion[64]. Other LR pairs, such as KLRB1-CLEC2D, although expressed at relatively low levels in certain cell types, are also essential to the TME. The interaction between the immune checkpoint molecule KLRB1 and CLEC2D is of great significance for tumor progression and immune evasion[65]. Previous studies have demonstrated that the interaction of KLRB1-CLEC2D can mediate immunosuppression and potentiate the development of GBM[66].

These findings implied that MIF, SPP1 and CD99 are indispensable for the TME. Therefore, we constructed hierarchical clustering maps for these three genes to illustrate the communication intensity between cell clusters and identify the key cell clusters involved in cellular communication. In line with our previous LR analysis, tumor cells and immune cells exhibited strong communication via the MIF signaling pathway, which was distinct from the SPP1 and CD99 signaling pathways (Figure 4G, J). This may be attributed to the expression of MIF receptors on various immune cells, such as CD74 on the surface of macrophages and dendritic cells. Additionally, the binding of MIF to its receptors activates multiple downstream signaling pathways, including ERK1/2, AMPK, and AKT, following MIF-receptor binding, thereby strengthening their interactions[67]. Within the SPP1 signaling pathway, the communication between macrophages, dendritic cells, and T cells was particularly intense (Figure 4I). SPP1 might enhance the interaction between myeloid cells and T cells, thereby suppressing immune surveillance. Substantial intercellular signaling communication occurred between tumor cells and immune cells in the MIF, SPP1, and CD99 signaling pathways. Among tumor cells and T cells, the direct or indirect interaction became the main factor contributing to the immunosuppressive microenvironment in GBM[61, 68].

Since we did not clearly discern the interaction between the HMsig and LR pairs, we then constructed a protein-protein interaction (PPI) network to explore the associations between them (Figure 4K). The network analysis identified a direct interaction between EFNB1 and OPHN1. In GBM, the high expression of EFNB1 was related to poor prognosis for patients and could potentially serve as a prognostic marker

and therapeutic target[69]. Moreover, elevated expression of both EFNB1 and OPHN1, which interact within the PPI network, was associated with worse patient outcomes (Supplementary Figure 3A).

Fig. 4. CellChat analysis of the communications between tumor cells and immune cells. Global communications are presented by circle plots showing the number of significant LR pairs in 10 cell clusters. B Chord diagram shows the communication strength at the gene signaling pathway level. C-F, H Significant LR pairs between tumor and immune cells, within myeloid cells, T cells and between myeloid and other immune cells. Dot color reflects communication probabilities, and dot size represents computed p-values (one-sided permutation test). Empty space indicates zero communication probability. G, I-J Three representative signaling pathways, MIF, SPP1, and CD99 pathways, were further analyzed. The inferred networks of communication between all cell types are displayed using hierarchical plots. K PPI networks between HMsig genes and LR pairs. Orange dots represent HMsig genes, blue dots represent LR pairs, and the color and thickness of the lines are determined by the combined score. The combined score is the standardized confidence metric from the STRING database.

Alt text: Graphs and data on cell communication between tumor cells and immune cells in GBM, with subfigures labelled from A to K, illustrating global communication networks, significant ligand-receptor pairs, hierarchical signaling pathways, and PPI networks.

Result 5 Elevated expression of immune checkpoint genes during T cell differentiation

T cells show diverse differentiation trajectories as a result of the marked impact of the TME[70]. We established a 3D developmental trajectory for T cells. From this

analysis, two common trajectories emerged. One trajectory commenced with naive T cells, passed through Treg cells, and ended at CD8_T_EX cells; the other started from naive T cells, proceeded through CD8_T_EM cells, and terminated at CD8_T_EX cells (Figure 5A, Supplementary Figure 3B). This bifurcation suggests that the exhaustion of T cells in GBM is a convergent process that can be achieved either through an immunosuppressive pathway or through the gradual decline of effector memory cell function. Meanwhile, we mapped the 2D trajectory for a more detailed and lucid depiction (Figure 5B-C). We used the R package clusterProfiler to conduct GO functional enrichment analysis on genes related to T-lineage differentiation. This allowed us to explore the impact of these genes on cellular physiological functions (Figure 5D, Supplementary Table 3). Genes associated with the initial T cell differentiation stage, including CD4, CXCR4, CD69, and KLRB1, display high expression levels in Cluster1 and Cluster2. The up-regulation of CXCR4 may be related to the enhanced migration ability of T cells and contribute to the aggregation of T cells[71]. Serving as T cell activation markers, CD69 and KLRB1 are markedly upregulated in activated CD4+ T cells[72, 73]. GO enrichment analysis reveals that Cluster 1 and 2 are enriched in functional pathways like positive regulation of the inflammatory response and cytokine production. Notably, genes expressed by Tregs cells, such as TIGIT, FOXP3, and IL2RA, show high expression in Cluster3. Cells clustered into Cluster3 possess functions such as regulation of T cell activation and positive regulation of leukocyte activation. These cells are crucial in immune response and cytotoxic killing. At the terminal stage of T cell differentiation, Cluster 5

characterized by high expression of CCL5 and LAG3 exhibited enrichment of functional pathways related to T cell exhaustion, including intercellular adhesion regulation and inhibiting immune cell activation.

At the same time, we monitored the expression changes of key genes that drive cellular development or state transitions during the pseudotime. CCL5 is a pivotal chemokine determining whether tumors will be infiltrated by T cells. As the T cell pseudotime trajectory progresses, the expression of CCL5 gradually increases (Figure 5E). While this is beneficial for recruiting T cells to infiltrate the tumor, it also serves as a marker of the malignant transformation process in GBM[74]. FOS, functioning as a TF, is involved in regulating various pathophysiological processes of cells. During T cell activation, the product of the FOS gene participated in modulating the expression of cytokine genes[75]. With the development of the T cell, the expression of FOS gradually decreased, which can affect the recruitment and activation state of T cells (Figure 5F). Besides, the analysis showed that the genes in Cluster 3 were highly expressed in the early stage of the trajectory and then gradually declined. This suggests that these genes might play an important role in the early stage but were rapidly suppressed or shut down, like FOXP3 and IL2RA expressed by Treg cells (Supplementary Figure 3C). However, Cluster 5 presented an opposite pattern (Figure 5D). For instance, the immune checkpoint molecules LAG3 was highly expressed at the terminal stage, corresponding to the temporal distribution of CD8_T_EX. This displays that the high expression of immune checkpoint molecules would lead to the exhaustion state of T cells. Unexpectedly, we found that the LR pairs on the T cell

surface were also key genes driving T-lineage evolution. Take HLA-DRA-CD4 for example, it promotes T cell activation and differentiation[76].

The co-expression of multiple co-inhibitory receptors is a crucial marker of T cell dysfunction. Building upon the previous analysis, the results demonstrated that LAG3 was highly expressed at the end stage of T cell differentiation. Thus, we investigated the gene expression of all immunosuppressive checkpoint molecules. We found that PD1 (PDCD1), LAG3 (LAG3), TIM3 (HAVCR2), and other two genes were highly expressed during the T-progression. The high expression of these three ICGs in CD8_T_EX cells is associated with the functional decline of CD8_T_EM cells, potentially facilitating their conversion into CD8_T_EX cells (Figure 5G-I, Supplementary Figure 3D-E).

Fig. 5 scRNA-seq profiles of dynamic changes in T cells. A Three-dimensional pseudotime analysis exploring the cellular trajectory of T cells based on highly variable genes. The black lines with arrows correspond to the principal graph learned by Monocle3, depicting the global developmental flow in 3D space. Individual dots represent single cells color-coded by their respective T cell subtypes. B Two-dimensional planar projection of the T cell developmental trajectory. C Pseudotime ordering of T cells initialized at the Naive_T cell cluster. The color gradient, ranging from purple (root) to yellow (terminal), represents the continuous progression of cells along the inferred developmental timeline. D The cell distribution of each T cell cluster along with the pseudotime (upper panel), color-coded by T cell clusters. Heatmap showing dynamic expression changes of genes in T cells (lower panel). The GO enrichment terms for each cluster are displayed on the right of the heatmap. E-F Gene expression dynamics along the trajectory, including

CCL5 (E), FOS (F). The color of dots coded according to their cell types and the line represents the gene expression at each time point. G-I Boxplot showed the expression of immune checkpoint molecules (HAVCR2, PDCD1, LAG3) in T cells. The color of dots coded according to their cell types. The significance of differences between each pair of cell clusters is marked by "*", with "ns" indicating no significant difference.

Alt text: Graphs and data on single-cell trajectory analysis of T cells, with subfigures labelled from A to I, illustrating pseudotime cellular trajectories, dynamic gene expression heatmaps, and immune checkpoint molecule expression levels.

Result 6 Disclosing synergistic TFs regulation in tumor cells and T cells for prognosis

TFs, as upstream regulatory elements, can directly influence the behaviors of tumor cells and the activities of immune cells. To identify transcription factors that drive the expression of HMsig and immune checkpoint molecules, we performed SCENIC analysis on two tumor cell populations and two CD8 T cell subsets exhibiting strong cellular interactions. In addition, we focused specifically on CD8_T_EM and CD8_T_EX cells because they represent a key transition point in the T cell trajectory, where effector memory T cells with tumor-killing functions shift into exhausted T cells. This transition is highly relevant to the immune escape mechanisms in GBM. The cell-specific TFs, ETS1 and RUNX3 were in an active state in CD8_T_EM and CD8_T_EX cells; SOX2 and EPAS1 were in an active state in tumor cells (Figure 6A, Supplementary Figure 4A). Among them, ETS1 was involved in angiogenesis, which enhances the invasiveness and metastatic potential of tumor cells[77]. SOX2, on the

other hand, promotes tumor cell proliferation by regulating genes associated with cell cycle progression[78]. All these suggest that TFs also participated in the regulation of the TME. Afterward, we constructed gene regulatory networks (GRNs) for CD8_T_EX, CD8_T_EM, and tumor cells to map specific TFs-gene interactions. (Figure 6B, Supplementary Figure 4L-M). The regulatory network centered around TFs like ETS1 and RUNX3, is essential for the occurrence, development and functional activation of multiple immune cells[79-82]. In light of the HMsig we uncovered, we probed for their corresponding driving TFs and obtain four of them, namely SOX11, SOX4, CEBPD, and EGR1. We then created a schematic diagram of the TF-HMsig/ICG regulatory mechanism (Figure 6C, D).

Understanding the cooperative interaction among TFs and its impact on GBM is crucial as it significantly affects GBM prognosis. Hence, we scrutinized the correlations among TFs at the bulk transcriptome level and found that the TFs were concordant in expression and significantly associated (Figure 6E, Supplementary Figure 4B). Additionally, the expressions of the TFs in two cell types at the single-cell level were consistent as well (Supplementary Figure 4C-K). We discovered that RUNX3 and CEBPD showed similar expression patterns. These two TFs also significantly differentiate patients in the high-risk and low-risk groups within the TCGA-CGGA datasets. This shows that the synergistic action of TFs with different regulatory modes can influence patient prognosis (Figure 6F-G).

When cells are confronted with various environmental stresses, such as oxidative stress and nutrient deficiency, the cooperative action of TFs can regulate the gene

expression within the cells, assisting the cells in adapting to environmental changes and maintaining cell survival and function[83]. During the process of cell differentiation, the cooperative action of TFs is also a key determinant of cell fate[84]. With the aim to explore the regulatory mechanisms of TFs with cooperative effects among cells, we performed functional enrichment analysis on the GRNs and searched for the motifs of TFs. In tumor cells, the sub networks regulated by SOX4 and SOX11 were enriched in pathways related to developmental cell growth, neuron migration, and dendrite morphogenesis, among others. Meanwhile, in T cells, the sub-networks regulated by ELF1 and RUNX3 were enriched in functional pathways associated with regulation of T cell activation, lymphocyte mediated immunity, and lymphocyte proliferation. Although the specific regulatory mechanisms of TFs diverge, TFs in tumor cells and T cells play vital roles in different cellular functions. TFs in tumor cells impact cell development, and those in T cells coordinate the immune response. These findings show a functional similarity between the two clusters of TFs. They are both involved in key cellular processes essential for the functions and survival of their respective cell types (Figure 6H).

Subsequently, we identified the motifs of TFs associated with HMsig and ICGs, as their expression levels hinge predominantly on specific motifs in their regulatory regions and the binding of corresponding TFs. We found that RUNX3 might simultaneously regulate the expressions of two immune checkpoint molecules, LAG3 and PDCD1. Hence, RUNX3 may coordinate the expression of two immune checkpoint molecules by binding to the same motif, thereby establishing a “dual-brake” mechanism

that more efficiently suppresses T cell activity. In the TME, this mechanism could be utilized by tumor cells to promote immune escape through upregulation of RUNX3 and simultaneous activation of PDCD1 and LAG3. Moreover, we discovered that the RUNX3 binding motif is highly conserved between humans and mice through extended motif analysis. Such conservation allows TFs to maintain stable regulation of gene expression and ensure proper biological function. RUNX3 has been shown to play a critical role in T cell differentiation and function, and its dysregulation is associated with impaired immune responses and cancer progression[85]. Additionally, the conservation of TF binding motifs, such as those of RUNX3, is essential for maintaining immune homeostasis and preventing pathological conditions like autoimmune diseases and cancer[86]. The concordance in the preferred binding motif sequences of these two genes is clearly of vital importance for precisely and thoroughly elucidating the immune escape mechanisms.

Fig. 6 Regulatory roles of TFs between tumor cells and T cells. A Heatmap shows the binary activity of cell-type-specific TFs identified via regulon specificity scores in tumor and CD8+ T cells. B Transcription regulatory network constructed by CD8_T_EX specific TFs and its target genes. Warm-toned dots represent TFs, Cold-toned dots represent targets gene. The color of the lines represents the number of motifs shared between the TFs and their target genes. C-D Schematic diagram of TF-HMsig/ICG regulatory mechanism. Orange circles represent TFs driving HMsig in tumor cells, yellow circles represent TFs driving immune checkpoint molecules in T cells, and blue and green hexagons represent the identified HMsig and ICGs. E-F Correlation analysis of TFs in TCGA-CGGA cohort, including SOX4-SOX11 (E) and RUNX3-CEBPD (F). The correlation

coefficient and significance markers are indicated in the lower right corner of the figure. G Survival analysis of RUNX3-CEBPD in TCGA-CGGA cohort. The yellow and blue line respectively indicate the high and low gene expression of RUNX3-CEBPD in TCGA-CGGA cohort. H TF co-regulation mechanisms in two cell types. On the left are the GO terms enriched by the TF regulatory sub networks, while on the right are the TF regulatory sub networks with synergistic interactions, along with the motifs corresponding to the HMsig/ICG regulated by these TFs. The synergistic mechanisms in tumor cells are shown in the upper section, with the regulatory network highlighted in orange. The synergistic mechanisms in T cells are also displayed in the upper section, with the regulatory network highlighted in yellow.

Alt text: Graphs and diagrams on the regulatory roles of transcription factors in tumor and CD8+ T cells, with subfigures labelled from A to H, illustrating transcription regulatory networks, synergistic TF interactions, survival analyses, and conserved binding motifs.

Result7 Spatial transcriptomics profiling of molecular heterogeneity and immunosuppressive microenvironment in glioblastoma

Spatial transcriptomic techniques allow high-resolution in situ measurement of gene expression, revealing expression gradients and identifying spatially organized cellular domains. To delineate the molecular and cellular architecture of GBM across anatomical regions, we collected 12 IDH-wt GBM spatial transcriptome samples (Figure 7A), including tumors from the right temporal lobe, left frontal lobe and other regions (Supplementary Table 1). To enable higher-resolution analysis at the cellular level, the CARD deconvolution algorithm was applied to map scRNA-seq-defined cell types onto spatial transcriptomic data, covering glia/neuronal cells, oligodendrocytes,

endothelial cells, pericytes, myeloid cells, T cells, and B cells (Figure 7B). The tumor region's delineation aligns with prior studies[87, 88], with regions predominantly mapped to glia/neuronal cells and oligodendrocytes, validated by the expression of marker gene (Figure 7C).

The spatial distribution map of cells revealed interpatient heterogeneity. While GBM5_1 and zh881t1 both originated from the right temporal lobe, the tumor in zh881t1 nearly occupied the entire section, highlighting divergent tumor expansion patterns. Similarly, extensive tumor cell distribution was observed in left temporal lobe sections (Supplementary Figure5). Notably, 12 samples showed low abundance of T cell, indicating GBM's inherent tendency to present as an immunologically "cold" tumor type. GBM5_1 exhibited extensive pericyte infiltration, consistent with the brain's high vascularity and GBM's angiotropic behavior. Pericytes, as key components of the neurovascular unit, may thus foster a microenvironment for tumor progression[89].

We further examined spatial expression patterns of HMsig, ICGs and TFs mediating regulatory interactions. HMsig such as IL13RA2, DCC, AEBP1 and OPHN1 showed high expression in tumor cells (Figure 7D). In patient GBM5_1, the delineated tumor region was identified in study[87] as a hypoxic and invasive zone and the remaining areas were divided into vascular enrichment areas. Additionally, the tumor tissue of patient GBM2 is located in the corpus callosum, and analysis indicates that the tumor area is primarily situated at the tissue margin, which may suggest an invasive tendency of the tumor along the corpus callosum-cortical junction area or perivascular spaces[90] (Supplementary Figure5). The high expression of HMsig in this area suggests

that it may directly reflect hypoxia stress intensity, spatially colocalizing with tumor invasion or vascular-sparse regions. T cells exhibited enrichment of immune checkpoint molecules, suggesting that there is a state of T cell exhaustion in the TME.

Furthermore, the high expression of cell type-specific TFs suggests their spatially coordinated co-expression patterns (Figure 7D-E). The tumor-associated TFs SOX4 and SOX11 demonstrate spatially co-regulated expression in diverse samples, underscoring their critical role in modulating HMsig. Meanwhile, T cell-related TF are also markedly expressed in localized tumor areas, indicating a synergistic interplay between tumor cells and T cells in specific spatial regions. This interaction may contribute to the formation of an immunosuppressive TME. These findings are consistent with cellular distribution patterns and supported by bulk and single-cell transcriptome analyses, reinforcing the immunosuppressive nature of the TME.

Fig. 7. The spatial expression patterns of HMsig, ICG and TFs. A H&E histological images of patient GBM5_1, mgh258, zh881t1, zh8811b, zh1007nec and zh1019t1 reveal the histological architecture of the tumor slices. B Description of ST data using cell type deconvolution. The color of each spot represents the proportion of each cell type in all spots, with the color intensity indicating the relative abundance. C-E The expression levels of cell marker, HMsig, ICG and TFs in representative samples. Dot colored by gene expression levels.

Alt text: Graphs and data on the spatial expression patterns of GBM biomarkers, with subfigures labelled from A to E, illustrating histological H&E histological images, spatial cell type deconvolution, and the localized expression of cell-lineage markers, HMsig genes, ICGs, and TFs.

Discussion

This study characterized the TME of GBM by clustering cells from tumor tissues, revealing the existence of individual differences within the microenvironment. Our research demonstrated that hallmarks enriched at both the single-cell and bulk levels are pivotal to cell cycle regulation and profoundly influence the transcriptional governance of GBM. This suggests that investigating the regulation of cell cycle related genes and TFs is essential for understanding the uncontrolled proliferation of GBM cells. Although numerous studies have explored the GBM microenvironment across both data modalities, no study has yet considered the hallmark features of malignant cells and the immune characteristics of immune “cold” and “hot” tumor samples. Due to the heterogeneity of GBM tissue and the existence of the BBB, there are significant differences in GBM patients’ TME in terms of the degree of immune cell infiltration, immune activity, and responsiveness to immunotherapy. This is also a challenge we need to face currently. We can more vividly reproduce the true state of the patient's disease by identifying the characteristics of tumor malignant cells. Dividing GBM samples into immune “cold” and immune “hot” states according to immune characteristics can more accurately capture the immune related features of GBM, and further deeply analyze and clearly present its immunosuppressive microenvironment. We obtained features from the hallmarks of single-cell tumor cells, the hallmarks of bulk immune “cold” samples, and the upregulated differentially expressed genes in bulk tumor cells, with the aim of achieving a more nuanced molecular profile of GBM across multiple scales. The five common hallmarks between single cell and bulk dimensions

confirms the transcriptional robustness of core malignant programs in GBM. These pathways, primarily associated with cell cycle regulation and proliferation, constitute a cross-modal conserved regulatory pattern, avoiding the influence of technical noise inherent in single-modal data. To ensure the clinical relevance of our model, we intersected these hallmark genes with bulk level immune cold hallmarks and upregulated DEGs from bulk tumor samples. Leveraging this curated gene set, we implemented a combined machine learning framework to construct an optimal prognostic model, leading to the identification of HMsig. After multilayer verification, our optimized model has good predictive and validation efficiency.

In an effort to gain a more in-depth characterization and regulatory mechanisms of TME, we undertook cell communication and cell trajectory analysis. Cell communication analysis revealed that direct or indirect interactions between tumor cells and T cells are the primary cause of the immunosuppressive microenvironment in GBM. These interactions impair immune function, disrupting the TME balance. Notably, the OPHN1-EFNB1 interaction emerged as a critical determinant of poor prognosis. As EFNB1 drives glioma invasion and immune remodeling[91, 92], its interplay with OPHN1—a Rho-GAP modulating RhoA/Rac1 activity—likely governs cytoskeletal plasticity[93]. This synergy facilitates GBM cells to invade and spread more aggressively into the brain tissue. Cell trajectory analysis indicated that the upregulation of immune checkpoint molecules leads to a progressive decline in T cell function. This functional impairment enables tumor cells to escape immune surveillance, a key factor contributing to tumor growth and metastasis.

Through SCENIC analysis, RUNX3 was found to simultaneously regulate the expression of LAG3 and PDCD1. This dual regulation inhibits the immune response of T cells within the TME, further highlighting the complex role of TFs in modulating the immune landscape. Our analysis also showed that TFs exhibit synergistic effects not only within cell clusters but also between different cell clusters. This regulatory interplay significantly impacts patient prognosis. Consistent with the studies by Timothy F et al[94, 95], we found that PDCD1 and LAG3 were significantly upregulated in glioblastoma multiforme. However, our study further revealed the mechanism by which RUNX3 simultaneously regulates these two genes through a shared motif, and this finding has not been reported previously. In the future, research on primary GBM could focus on using CRISPR-Cas9 genome editing technologies to verify the synergistic effect of RUNX3 and CEBPD in the process of immune escape, so as to better understand the dynamic immune evasion strategies in GBM. Spatial transcriptomic analysis further confirmed the co-localization of the HMsig genes with ICGs and their transcription factors (especially SOX4, SOX11, and RUNX3) in specific tissue regions, forming an immunosuppressive microenvironment. The co-expression of SOX4 and SOX11 suggests a spatial regulatory mechanism that enhances HMsig genes expression and drives tumor progression. RUNX3 upregulates the expression of immune checkpoint molecules LAG3 and PDCD1, further promoting the co-regulation represented by the “dual-brake” mechanism, thereby facilitating immune escape by tumor cells. This spatial organization driven by a coordinated transcriptional program provides a structural basis for the persistent treatment resistance in GBM.

In summary, this study revealed diverse immune escape mechanisms in GBM, including the interaction of key LR pairs between cell clusters, the upregulation of immune checkpoint molecules, and the synergistic regulation of RUNX3-CEBPD. These findings displayed the complexity of the TME and the refractory nature of GBM. Although this study identified only seven risk genes, five immune checkpoint molecules and a pair of synergistic TFs across cell clusters that affect patient prognosis, this may be due to the high heterogeneity and the limited representativeness of the tumor samples. Nonetheless, these results still offer fresh viewpoints and possible biomarkers that can enhance our comprehension of the proliferation and prognosis of GBM. Collectively, these results not only enhance our understanding of the complexity of the TME but also provide important theoretical foundations and new research directions for the diagnosis and treatment of GBM.

Data availability

All datasets analyzed in this study are publicly available from established repositories. Single-cell RNA-seq data for GBM were obtained from the GEO under accession GSE182109[96]. Bulk transcriptomic training cohort (n = 428) was assembled from TCGA (TCGA-GBM[97], TCGA-GBMLGG[98]) and CGGA (CGGA.mRNAseq_325.RSEM-genes[99], CGGA.mRNAseq_693.RSEM-genes[100]). Normal brain transcriptomic profiles (n = 105) were retrieved from GTEx project [101]. Independent validation microarray datasets were obtained from GEO under accessions GSE7696[102], GSE42669[103], GSE16011[104], and GSE108474[105]. Spatial

transcriptomics data for primary IDH-wildtype GBM were obtained from GEO under accessions GSE194329[87] and GSE237183[88].

Availability of Source Code and Requirements

Project name: GBM-TIME

Project homepage: https://github.com/travilucas/GBM

Operating system(s): Platform independent

Programming language: R

Other requirements: R 4.2.0 or higher

License: MIT license

RRID: SCR_027948

Additional Files

Supplementary Fig. S1. Detailed characterization of the GBM microenvironment. (A-C) Clustree plots visualizing the clustering stability of major cell lineages (A), T cell subtypes (B), and myeloid cell subtypes (C) across varying clustering resolutions (0.1 to 1.0). Dot size represents cell count, and colors indicate clustering stability. (D) Box plot illustrating TotalCNV scores across different cell types to distinguish non-malignant clusters. The red dashed line represents the mean of the overall scores. (E, F) UMAP projections showing identified sub-clusters within T cells (E) and myeloid cells (F). (G, H) Dot plots showing the expression of canonical marker genes in myeloid subtypes (G) and T cell subtypes (H). Dot size indicates the proportion of expressing cells, and color intensity represents average expression levels. (I) Delta area plot showing the relative change in the area under the CDF curve for each k (k=2-9).

Supplementary Fig. S2. Identification and assessment of the optimal model and HMsig. (A) Heatmap showing the C-index of 101 machine learning-based prediction models across the train and test sets, with the StepCox(forward) + RSF model identified as the optimal combination. (B) Kaplan–Meier curves of OS for high- and low-risk groups in the train set determined by the StepCox(forward) + RSF model. (C) Venn diagram illustrating the overlap of candidate genes between malignant-cell hallmark features, immune-related hallmark features, and up-regulated DEGs. (D) Horizontal bar plot displaying the genetic importance scores of the seven core HMsig genes. (E) Forest plot summarizing the meta-analysis of univariate Cox regression for the optimized model in both train and test cohorts. (F) Heatmap comparing the HR values of our optimized model with other previously published GBM signatures in both train and test sets. * $p < 0.05$, ** $p < 0.01$.

Supplementary Fig. S3. The role of intercellular interactions and T-cell pseudotime dynamics in GBM. (A) Kaplan-Meier survival analysis of overall survival (OS) based on the co-expression of OPHN1 and EFNB1 LR pairs in the GSE7696 cohort. (B) Three-dimensional UMAP visualization of the T-cell developmental trajectory inferred by Monocle3, with cells color-coded by pseudotime beginning from the Naive_T cell cluster. (C) Gene expression dynamics of IL2RA along the T-cell pseudotime trajectory (D, E) Box plots showing the expression levels of immune checkpoint molecules KLRB1 (D) and CLEC2D (E) across T-cell subtypes. The color of dots is coded according to cell types. * $p < 0.05$, **** $p < 0.0001$; ns: no significant difference.

Supplementary Fig. S4. TFs exhibit synergistic effects between tumor cells and T cells. (A) Dot plot illustrating the regulon specificity score used to identify representative regulons for each cell type. The size and color gradient of the dots represent the magnitude and significance of the specificity scores. (B) Heatmap showing the correlation analysis of TFs in the TCGA-CGGA

cohort, with colors indicating correlation coefficients and asterisks denoting significance ($p < 0.05$). (C–K) Scatter plots demonstrating the correlation between specific TF pairs at the single-cell level, with correlation coefficients (r) and p-values indicated in each panel. (L, M) Gene regulatory networks of TFs and their target genes in tumor cells (L) and CD8_T_EM cells (M). Warm-toned dots represent TFs, cold-toned dots represent target genes, and line colors indicate shared motifs.

Supplementary Fig. S5. The spatial expression patterns of HMsig, ICGs and TFs in GBM. (A) H&E histological images of patients GBM2, zh916bulk, zh881inf, zh8811a, zh1007inf, and zh1019inf, revealing the structural architecture of the tumor slices. (B) Spatial transcriptomics data visualized through cell type deconvolution across various samples, where color represents cell type and intensity indicates relative abundance. (C) Spatial expression maps of representative cell type markers used to validate deconvolution results. (D, E) Localized expression patterns of risk-related HMsig genes, ICGs and TFs within specific histological regions.

Supplementary Data S1. Comprehensive inventory of multi-dimensional transcriptomic datasets utilized in this study. This file provides a detailed overview of all data cohorts, including single-cell RNA-seq, bulk RNA-seq, and spatial transcriptomics datasets.

Supplementary Data S2. Optimized clustering resolution parameters and marker genes for cell type annotation. This table details the specific resolution settings used for clustering and catalogs the marker genes employed to identify major cell lineages and sub-clusters within T-cell and myeloid populations.

Supplementary Data S3. GO enrichment results of functional modules along T-cell trajectories. This dataset catalogs the biological processes and molecular pathways associated with the dynamic gene expression changes identified in the T-cell pseudotime analysis.

Funding sources

This work was supported by grants from the National Science and Technology Major Program [2024ZD0530500]; and National Natural Science Foundation of China [62472131, 62502128], and the Key Research and Development Program of Heilongjiang Province [2024ZX12C27], and the China Postdoctoral Science Foundation [2024M760709], and Heilongjiang Postdoctoral Fund [LBH-Z24210], and Longjiang New Era Outstanding Doctoral Dissertation Project Grant [LJYXL2024-069], and Harbin Medical University Fund [2025-KYYWF-ZR0297].

Competing interests

The authors declare no competing interests.

CRediT authorship contribution statement

Tengyue Li: Writing–review & editing, Validation, Methodology, Conceptualization. **Wanqi Mi:** Validation, Methodology, Investigation. **Huarui Yan:** Writing–original draft, Methodology, Investigation. **Yining Ma:** Validation; Investigation. **Han Jiang:** Visualization, Investigation. **Xiaoxu Yang:** Visualization; Investigation. **Yunpeng Zhang:** Conceptualization. **Congxue Hu:** Conceptualization.

References

1. Yeo, A.T., et al., *Single-cell RNA sequencing reveals evolution of immune landscape during glioblastoma progression.* Nature Immunology, 2022. **23**(6): p. 971-+.
2. Caniglia, J.L., et al., *Beyond glucose: alternative sources of energy in glioblastoma.* Theranostics, 2021. **11**(5): p. 2048-2057.
3. Wang, R., et al., *Single-cell RNA sequencing reveals changes in glioma-associated macrophage polarization and cellular states of malignant gliomas with high AQP4 expression.* Cancer Gene Therapy, 2023. **30**(5): p. 716-726.
4. Xie, Y., et al., *Key molecular alterations in endothelial cells in human glioblastoma uncovered through single-cell RNA sequencing.* JCI Insight, 2021. **6**(15).
5. Hambardzumyan, D., D.H. Gutmann, and H. Kettenmann, *The role of microglia and macrophages in glioma maintenance and progression.* Nature Neuroscience, 2016. **19**(1): p. 20-27.
6. Verdugo, E., I. Puerto, and M.A. Medina, *An update on the molecular biology of glioblastoma, with clinical implications and progress in its treatment.* Cancer Communications, 2022. **42**(11): p. 1083-1111.
7. Daisy Precilla, S., et al., *Crosstalk between PI3K/AKT/mTOR and WNT/β-Catenin signaling in GBM - Could combination therapy checkmate the collusion?* Cell Signal, 2022. **95**: p. 110350.
8. Yuan, Y.M., et al., *Wnt signaling: Modulating tumor-associated macrophages and related immunotherapeutic insights.* Biochemical Pharmacology, 2024. **223**.
9. Torrisi, F., et al., *The Hallmarks of Glioblastoma: Heterogeneity, Intercellular Crosstalk and Molecular Signature of Invasiveness and Progression.* Biomedicines, 2022. **10**(4).
10. Carvajal, L.A., et al., *E2F7, a novel target, is up-regulated by p53 and mediates DNA damage-dependent transcriptional repression.* Genes & Development, 2012. **26**(14): p. 1533-1545.
11. Lambert, S.A., et al., *The Human Transcription Factors.* Cell, 2018. **172**(4): p. 650-665.
12. Wang, Q.X., et al., *Comprehensive Analysis of the E2F Transcription Factor Family in Human Lung Adenocarcinoma.* International Journal of General Medicine, 2022. **15**: p. 5973-5984.
13. Liu, X.S., et al., *Comprehensive Analysis of Prognostic and Immune Infiltrates for E2F Transcription Factors in Human Pancreatic Adenocarcinoma.* Frontiers in Oncology, 2021. **10**.
14. Papavassiliou, K.A. and A.G. Papavassiliou, *Transcription factors in glioblastoma - Molecular pathogenesis and clinical implications.* Biochimica Et Biophysica Acta-Reviews on Cancer, 2022. **1877**(1).
15. Ma, T.J., et al., *Reprogramming Transcription Factors Oct4 and Sox2 Induce a BRD-Dependent Immunosuppressive Transcriptome in GBM-Propagating Cells.* Cancer Research, 2021. **81**(9): p. 2457-2469.
16. Myers, B.L., et al., *Transcription factors ASCL1 and OLIG2 drive glioblastoma initiation and co-regulate tumor cell types and migration.* Nature Communications, 2024. **15**(1).
17. Korsunsky, I., et al., *Fast, sensitive and accurate integration of single-cell data with Harmony.* Nat Methods, 2019. **16**(12): p. 1289-1296.
18. Hu, C., et al., *CellMarker 2.0: an updated database of manually curated cell markers in human/mouse and web tools based on scRNA-seq data.* Nucleic Acids Res, 2023. **51**(D1): p. D870-D876.
19. Patel, A.P., et al., *Single-cell RNA-seq highlights intratumoral heterogeneity in primary*

glioblastoma. Science, 2014. **344**(6190): p. 1396-401.
20. Venteicher, A.S., et al., *Decoupling genetics, lineages, and microenvironment in IDH-mutant gliomas by single-cell RNA-seq.* Science, 2017. **355**(6332).
21. Tirosh, I., et al., *Single-cell RNA-seq supports a developmental hierarchy in human oligodendroglioma.* Nature, 2016. **539**(7628): p. 309-313.
22. Ahrends, R., et al., *Controlling low rates of cell differentiation through noise and ultrahigh feedback.* Science, 2014. **344**(6190): p. 1384-9.
23. Liu, Z., et al., *Machine learning-based integration develops an immune-derived lncRNA signature for improving outcomes in colorectal cancer.* Nat Commun, 2022. **13**(1): p. 816.
24. Jin, S., et al., *Inference and analysis of cell-cell communication using CellChat.* Nat Commun, 2021. **12**(1): p. 1088.
25. Szklarczyk, D., et al., *The STRING database in 2023: protein-protein association networks and functional enrichment analyses for any sequenced genome of interest.* Nucleic Acids Res, 2023. **51**(D1): p. D638-D646.
26. Shannon, P., et al., *Cytoscape: a software environment for integrated models of biomolecular interaction networks.* Genome Res, 2003. **13**(11): p. 2498-504.
27. Cao, J., et al., *The single-cell transcriptional landscape of mammalian organogenesis.* Nature, 2019. **566**(7745): p. 496-502.
28. Xu, S., et al., *Using clusterProfiler to characterize multiomics data.* Nat Protoc, 2024. **19**(11): p. 3292-3320.
29. Aibar, S., et al., *SCENIC: single-cell regulatory network inference and clustering.* Nat Methods, 2017. **14**(11): p. 1083-1086.
30. Ma, Y. and X. Zhou, *Spatially informed cell-type deconvolution for spatial transcriptomics.* Nat Biotechnol, 2022. **40**(9): p. 1349-1359.
31. Wang, Z.X., et al., *SVIP reduces IGFBP-2 expression and inhibits glioblastoma progression via stabilizing PTEN.* Cell Death Discovery, 2024. **10**(1).
32. Kim, J.A., et al., *VBP1 represses cancer metastasis by enhancing HIF-1α degradation induced by pVHL.* Febs Journal, 2018. **285**(1): p. 115-126.
33. Wang, S.H., et al., *Mechanism of Notch Signaling Pathway in Malignant Progression of Glioblastoma and Targeted Therapy.* Biomolecules, 2024. **14**(4).
34. Wang, H., et al., *Analysis of the EGFR Amplification and CDKN2A Deletion Regulated Transcriptomic Signatures Reveals the Prognostic Significance of SPATS2L in Patients With Glioma.* Front Oncol, 2021. **11**: p. 551160.
35. Sharpe, M.A., et al., *Hijacking Sexual Immuno-Privilege in GBM-An Immuno-Evasion Strategy.* Int J Mol Sci, 2021. **22**(20).
36. Liu, X., et al., *Sohlh1 suppresses glioblastoma cell proliferation, migration, and invasion by inhibition of Wnt/beta-catenin signaling.* Mol Carcinog, 2018. **57**(4): p. 494-502.
37. Lin, H., et al., *Understanding the immunosuppressive microenvironment of glioma: mechanistic insights and clinical perspectives.* J Hematol Oncol, 2024. **17**(1): p. 31.
38. Fridman, W.H., et al., *The immune contexture in human tumours: impact on clinical outcome.* Nat Rev Cancer, 2012. **12**(4): p. 298-306.
39. Malan, A.F., *The need for regional planning of perinatal care.* S Afr Med J, 1975. **49**(33): p. 1363-4.
40. Di Carlo, E., G. Forni, and P. Musiani, *Neutrophils in the antitumoral immune response.* Chem

Immunol Allergy, 2003. **83**: p. 182-203.

41. Nathan, C., *Neutrophils and immunity: challenges and opportunities.* Nat Rev Immunol, 2006. **6**(3): p. 173-82.
42. Hampton, M.B., A.J. Kettle, and C.C. Winterbourn, *Inside the neutrophil phagosome: oxidants, myeloperoxidase, and bacterial killing.* Blood, 1998. **92**(9): p. 3007-17.
43. Pham, C.T., *Neutrophil serine proteases: specific regulators of inflammation.* Nat Rev Immunol, 2006. **6**(7): p. 541-50.
44. Lysiak, M., et al., *Deletions on Chromosome Y and Downregulation of the <i>SRY</i> Gene in Tumor Tissue Are Associated with Worse Survival of Glioblastoma Patients.* Cancers, 2021. **13**(7).
45. Helmink, B.A., et al., *B cells and tertiary lymphoid structures promote immunotherapy response.* Nature, 2020. **577**(7791): p. 549-+.
46. Yang, C., et al., *B cells promote tumor progression via STAT3 regulated-angiogenesis.* PLoS One, 2013. **8**(5): p. e64159.
47. Fortelny, N., et al., *JAK-STAT signaling maintains homeostasis in T cells and macrophages.* Nature Immunology, 2024.
48. Hu, C., et al., *The androgen receptor expression and association with patient's survival in different cancers.* Genomics, 2020. **112**(2): p. 1926-1940.
49. Werner, C.K., et al., *Expression of the Androgen Receptor Governs Radiation Resistance in a Subset of Glioblastomas Vulnerable to Antiandrogen Therapy.* Mol Cancer Ther, 2020. **19**(10): p. 2163-2174.
50. Zhao, N., et al., *Androgen Receptor, Although Not a Specific Marker For, Is a Novel Target to Suppress Glioma Stem Cells as a Therapeutic Strategy for Glioblastoma.* Front Oncol, 2021. **11**: p. 616625.
51. Ghantasala, S., et al., *High-Grade Gliomas from Subventricular Zone: Proteomic Drivers of Aggressiveness Using Fluorescence-Guided Multiple Sampling.* Omics-a Journal of Integrative Biology, 2023. **27**(12): p. 598-606.
52. Lang, F.C., et al., *Abrogation of the G2/M checkpoint as a chemosensitization approach for alkylating agents.* Neuro-Oncology, 2024. **26**(6): p. 1083-1096.
53. Chen, H.Z., S.Y. Tsai, and G. Leone, *Emerging roles of E2Fs in cancer: an exit from cell cycle control.* Nat Rev Cancer, 2009. **9**(11): p. 785-97.
54. Li, F., et al., *In Vivo Epigenetic CRISPR Screen Identifies Asf1a as an Immunotherapeutic Target in Kras-Mutant Lung Adenocarcinoma.* Cancer Discov, 2020. **10**(2): p. 270-287.
55. Wu, Y., et al., *ASF1a inhibition induces p53-dependent growth arrest and senescence of cancer cells.* Cell Death Dis, 2019. **10**(2): p. 76.
56. Yin, X., et al., *Histone chaperone ASF1A accelerates chronic myeloid leukemia blast crisis by activating Notch signaling.* Cell Death Dis, 2022. **13**(10): p. 842.
57. Su, L., et al., *H2A.Z.1 crosstalk with H3K56-acetylation controls gliogenesis through the transcription of folate receptor.* Nucleic Acids Res, 2018. **46**(17): p. 8817-8831.
58. Wang, M.H., et al., *Oligophrenin-1 moderates behavioral responses to stress by regulating parvalbumin interneuron activity in the medial prefrontal cortex.* Neuron, 2021. **109**(10): p. 1636-+.
59. Huang, J.J., et al., *WDR62 regulates spindle dynamics as an adaptor protein between TPX2/Aurora A and katanin.* Journal of Cell Biology, 2021. **220**(8).

60. Liu, J., et al., *Androgen deprivation‑induced OPHN1 amplification promotes castration‑resistant prostate cancer.* Oncol Rep, 2022. **47**(1).
61. Chen, W., et al., *Single-cell RNA-seq reveals MIF-(CD74+CXCR4) dependent inhibition of macrophages in metastatic papillary thyroid carcinoma.* Oral Oncology, 2024. **148**.
62. Takheaw, N., et al., *The presence of membrane bound CD99 ligands on leukocyte surface.* BMC Res Notes, 2020. **13**(1): p. 496.
63. Lamano, J.B., et al., *Glioblastoma-Derived IL6 Induces Immunosuppressive Peripheral Myeloid Cell PD-L1 and Promotes Tumor Growth.* Clin Cancer Res, 2019. **25**(12): p. 3643-3657.
64. Yan, Y., et al., *Multi-omic profiling highlights factors associated with resistance to immuno-chemotherapy in non-small-cell lung cancer.* Nature Genetics, 2025. **57**(1).
65. Zhu, Y.Y., et al., *Comprehensive pan-cancer analysis of KLRB1-CLEC2D pair and identification of small molecule inhibitors to disrupt their interaction.* International Immunopharmacology, 2024. **140**.
66. Mathewson, N.D., et al., *Inhibitory CD161 receptor identified in glioma-infiltrating T cells by single-cell analysis.* Cell, 2021. **184**(5): p. 1281-+.
67. Jankauskas, S.S., et al., *Evolving complexity of MIF signaling.* Cellular Signalling, 2019. **57**: p. 76-88.
68. Klasen, C., et al., *MIF Promotes B Cell Chemotaxis through the Receptors CXCR4 and CD74 and ZAP-70 Signaling.* Journal of Immunology, 2014. **192**(11): p. 5273-5284.
69. Shi, Y.H., et al., *EFNB1 Acts as a Novel Prognosis Marker in Glioblastoma through Bioinformatics Methods and Experimental Validation.* Journal of Oncology, 2021. **2021**.
70. Liu, B.L., et al., *Single-cell meta-analyses reveal responses of tumor-reactive <i>CXCL13</i>⁺ T cells to immune-checkpoint blockade.* Nature Cancer, 2022. **3**(9): p. 1123-+.
71. Zhao, F.Y., et al., *The CXCR4-CXCL12 axis promotes T cell reconstitution via efficient hematopoietic immigration.* Journal of Genetics and Genomics, 2022. **49**(12): p. 1138-1150.
72. You, Y.C., et al., *Crystalline silica-induced recruitment and immuno-imbalance of CD4⁺ tissue resident memory T cells promote silicosis progression.* Communications Biology, 2024. **7**(1).
73. Zhang, Z.H., et al., *KLRB1 defines an activated phenotype of CD4+T cells and shows significant upregulation in patients with primary Sjo<spacing diaeresis>gren's syndrome.* International Immunopharmacology, 2024. **133**.
74. Yeo, E.C.F., et al., *The Role of Cytokines and Chemokines in Shaping the Immune Microenvironment of Glioblastoma: Implications for Immunotherapy.* Cells, 2021. **10**(3).
75. Zhang, Y., et al., *An advanced network pharmacology investigation of Shufeng Jiedu formula in the treatment for respiratory tract infection based on LC-MS, data mining, molecular docking, and molecular dynamic simulation.* Brief Bioinform, 2025. **26**(4).
76. Künzli, M. and D. Masopust, *CD4⁺ T cell memory.* Nature Immunology, 2023. **24**(6): p. 903-914.
77. Yang, L.X., et al., *ETS1 promoted cell growth, metastasis and epithelial-mesenchymal transition process in melanoma by regulating miR-16-mediated SOX4 expression.* Melanoma Research, 2021. **31**(4): p. 298-308.
78. Bhutada, I., et al., *CDK7 and CDK9 inhibition interferes with transcription, translation, and stemness, and induces cytotoxicity in GBM irrespective of temozolomide sensitivity.* Neuro-

Oncology, 2024. **26**(1): p. 70-84.

79. Zhou, P.P., et al., *Single-cell CRISPR screens in vivo map T cell fate regulomes in cancer.* Nature, 2023. **624**(7990): p. 154-+.

80. Shin, B.Y., et al., *Runx factors launch T cell and innate lymphoid programs via direct and gene network-based mechanisms.* Nature Immunology, 2023.

81. Liu, Z.Z., et al., *Epigenetic reprogramming of Runx3 reinforces CD8+T-cell function and improves the clinical response to immunotherapy.* Molecular Cancer, 2023. **22**(1).

82. Milner, J.J., et al., *Runx3 programs CD8⁺ T cell residency in non-lymphoid tissues and tumours.* Nature, 2017. **552**(7684): p. 253-+.

83. Kang, P., et al., *<i>Drosophila</i> Kruppel homolog 1 represses lipolysis through interaction with dFOXO.* Scientific Reports, 2017. **7**.

84. Diacou, R., et al., *Cell fate decisions, transcription factors and signaling during early retinal development.* Prog Retin Eye Res, 2022. **91**: p. 101093.

85. Ito, Y. and K. Miyazono, *RUNX transcription factors as key targets of TGF-beta superfamily signaling.* Curr Opin Genet Dev, 2003. **13**(1): p. 43-7.

86. Egawa, T. and D.R. Littman, *ThPOK acts late in specification of the helper T cell lineage and suppresses Runx-mediated commitment to the cytotoxic T cell lineage.* Nat Immunol, 2008. **9**(10): p. 1131-9.

87. Ren, Y., et al., *Spatial transcriptomics reveals niche-specific enrichment and vulnerabilities of radial glial stem-like cells in malignant gliomas.* Nat Commun, 2023. **14**(1): p. 1028.

88. Greenwald, A.C., et al., *Integrative spatial analysis reveals a multi-layered organization of glioblastoma.* Cell, 2024. **187**(10): p. 2485-2501.e26.

89. Pombero, A., R. Garcia-Lopez, and S. Martínez, *Pericyte-Glioblastoma Cell Interaction: A Key Target to Prevent Glioblastoma Progression.* Cells, 2023. **12**(9).

90. Mohan, S., et al., *Detection of occult neoplastic infiltration in the corpus callosum and prediction of overall survival in patients with glioblastoma using diffusion tensor imaging.* Eur J Radiol, 2019. **112**: p. 106-111.

91. Zheng, Y. and J. Shi, *EFNB1 drives glioma progression and shapes the immune microenvironment: a potential prognostic biomarker.* Discov Oncol, 2025. **16**(1): p. 249.

92. Shi, Y., et al., *EFNB1 Acts as a Novel Prognosis Marker in Glioblastoma through Bioinformatics Methods and Experimental Validation.* J Oncol, 2021. **2021**: p. 4701680.

93. Cho, H.J., et al., *EphrinB1 promotes cancer cell migration and invasion through the interaction with RhoGDI1.* Oncogene, 2018. **37**(7): p. 861-872.

94. Cloughesy, T.F., et al., *Neoadjuvant anti-PD-1 immunotherapy promotes a survival benefit with intratumoral and systemic immune responses in recurrent glioblastoma.* Nature Medicine, 2019. **25**(3): p. 477-+.

95. Mathios, D. and M. Lim, *Why is immunotherapy for glioblastoma "Lag"-ging.* Oncotarget, 2019. **10**(12): p. 1228-1229.

96. Abdelfattah, N., et al., *Single-cell analysis of human glioma and immune cells identifies S100A4 as an immunotherapy target.* Nat Commun, 2022. **13**(1): p. 767.

97. Cancer Genome Atlas Research Network. *TCGA-GBM.htseq_counts*. 2024; https://xenabrowser.net/datapages/.

98. Cancer Genome Atlas Research Network. *TCGA.GBMLGG.sampleMap_HiSeqV2*. 2024; https://xenabrowser.net/datapages/.

99. Chinese Glioma Genome Atlas. *CGGA.mRNAseq_325.RSEM-genes*. 2024; http://www.cgga.org.cn/.
100. Chinese Glioma Genome Atlas. *CGGA.mRNAseq_693.RSEM-genes*. 2024; http://www.cgga.org.cn/.
101. GTEx Consortium. *gtex_gene_expected_count (Brain-cortex)*. 2024; https://gtexportal.org/home/downloads/adult-gtex/bulk_tissue_expression.
102. Murat, A., et al., *Stem cell-related "self-renewal" signature and high epidermal growth factor receptor expression associated with resistance to concomitant chemoradiotherapy in glioblastoma.* J Clin Oncol, 2008. **26**(18): p. 3015-24.
103. Joo, K.M., et al., *Patient-specific orthotopic glioblastoma xenograft models recapitulate the histopathology and biology of human glioblastomas in situ.* Cell Rep, 2013. **3**(1): p. 260-73.
104. Gravendeel, L.A., et al., *Intrinsic gene expression profiles of gliomas are a better predictor of survival than histology.* Cancer Res, 2009. **69**(23): p. 9065-72.
105. Gusev, Y., et al., *The REMBRANDT study, a large collection of genomic data from brain cancer patients.* Sci Data, 2018. **5**: p. 180158.

Figure 1

Click here to access/download;Figure;Figure 1.tif

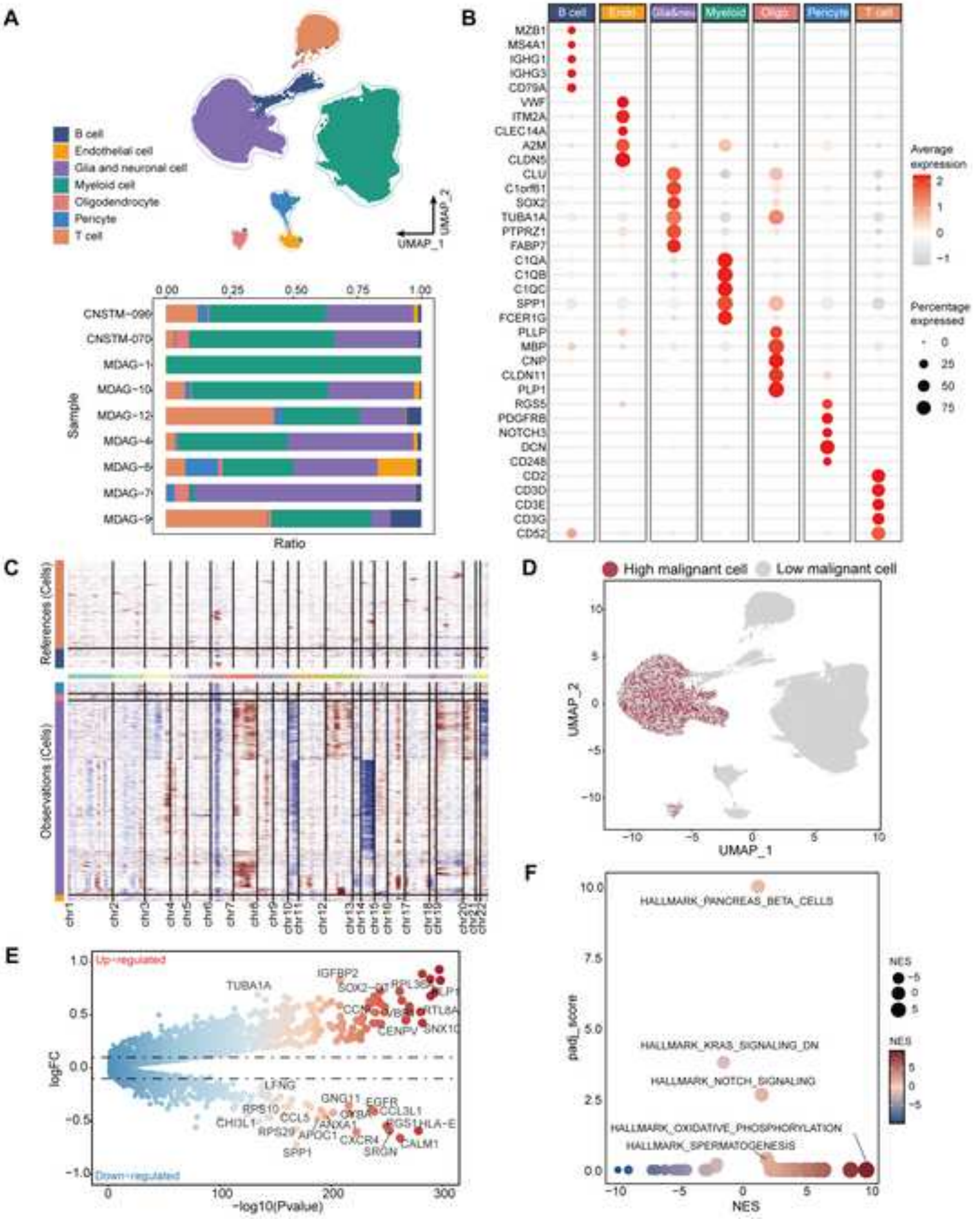

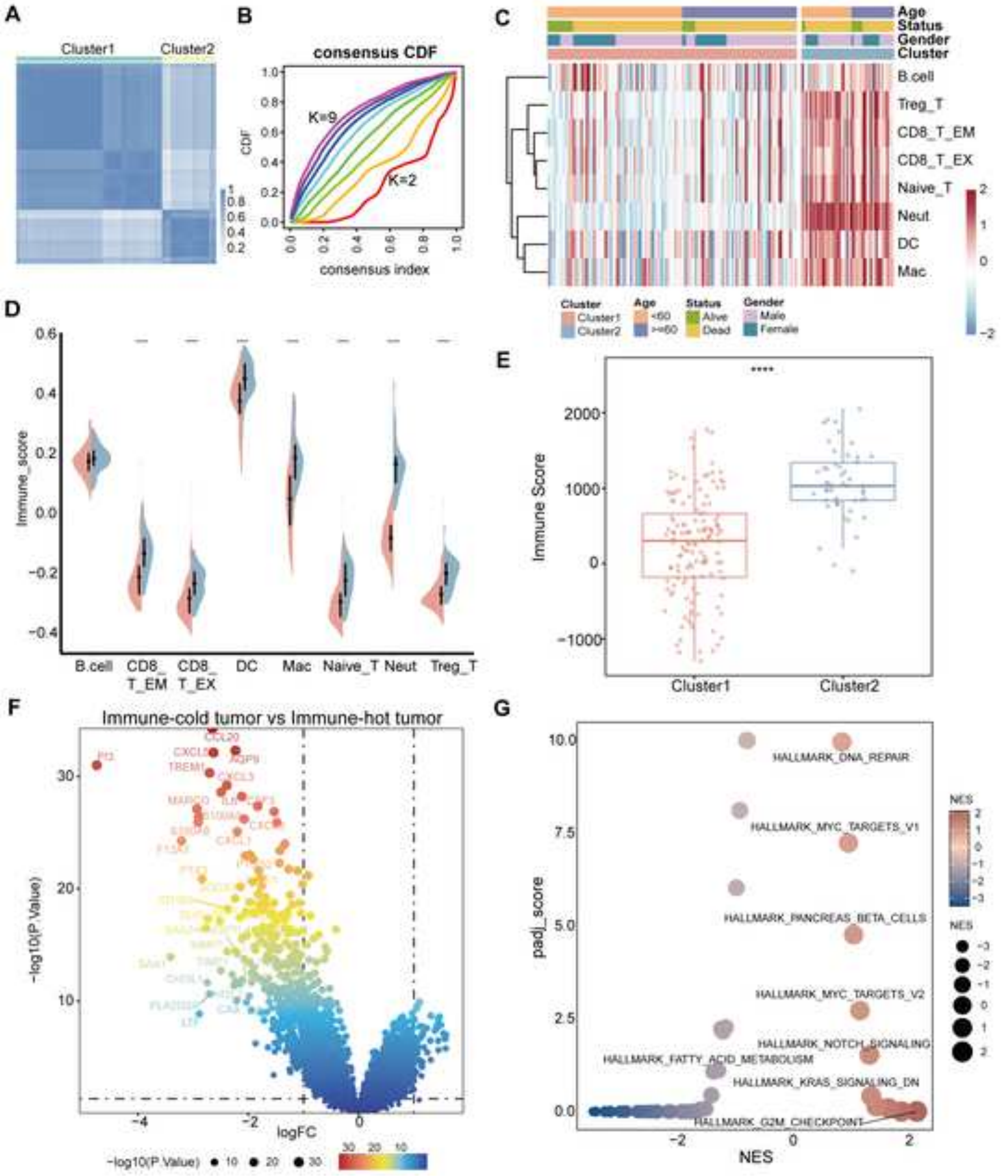
A
Cluster1
Cluster2
B
consensus CDF
K=9
K=2
CDF
consensus index
C
Age
Status
Gender
Cluster
B.cell
Treg_T
CD8_T_EM
CD8_T_EX
Naive_T
Neut
DC
Mac
Cluster
Cluster1
Cluster2
Age
<60
>=60
Status
Alive
Dead
Gender
Male
Female
D
Immune_score
B.cell
CD8_T_EM
CD8_T_EX
DC
Mac
Naive_T
Neut
Treg_T
E
Immune Score
****
Cluster1
Cluster2
F
Immune-cold tumor vs Immune-hot tumor
-log10(P.Value)
logFC
G
padj_score
NES
HALLMARK_DNA_REPAIR
HALLMARK_MYC_TARGETS_V1
HALLMARK_PANCREAS_BETA_CELLS
HALLMARK_MYC_TARGETS_V2
HALLMARK_NOTCH_SIGNALING
HALLMARK_FATTY_ACID_METABOLISM
HALLMARK_KRAS_SIGNALING_DN
HALLMARK_G2M_CHECKPOINT

Figure 3

Click here to access/download;Figure;Figure 3.tif

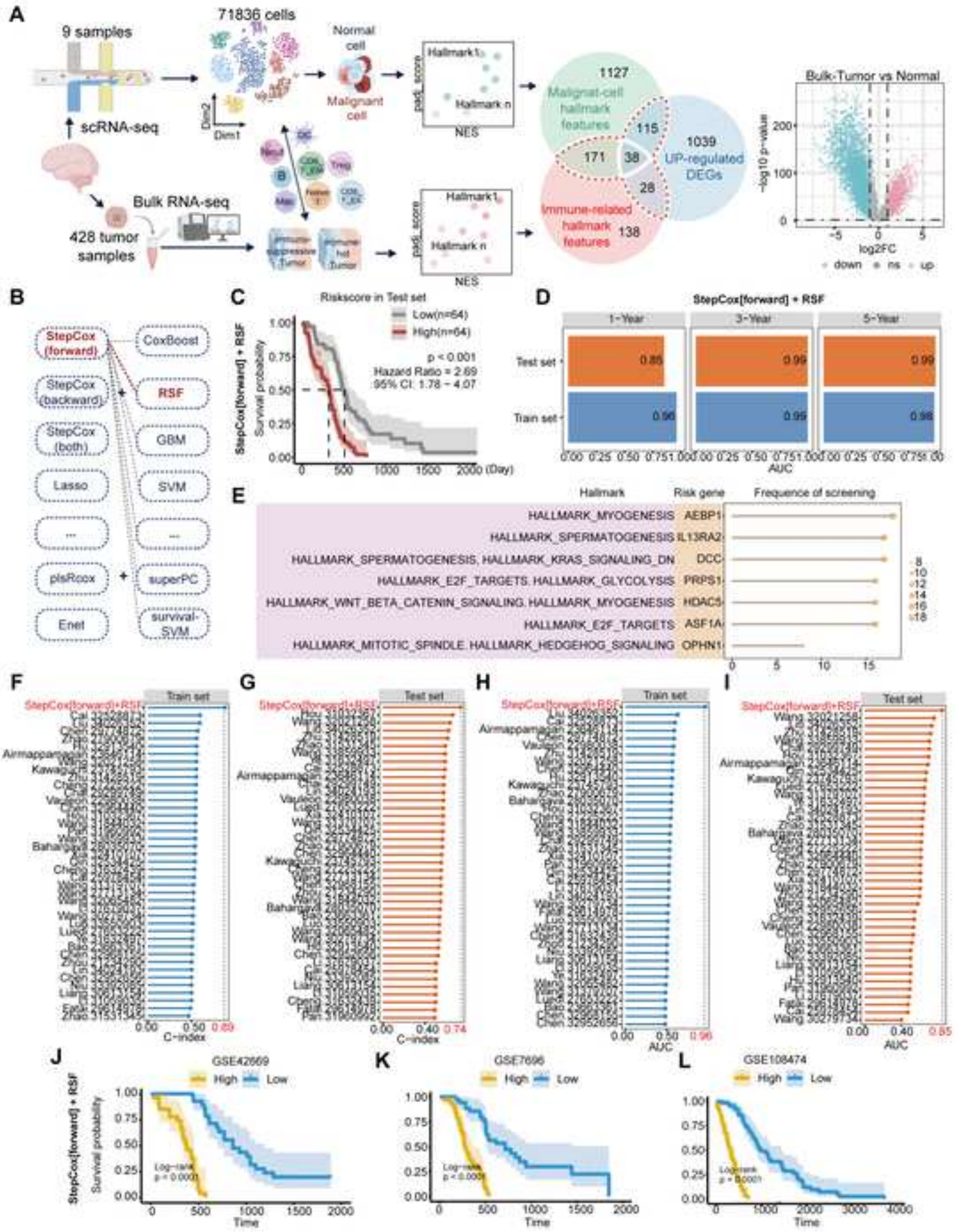

Figure 4 Click here to access/download;Figure;Figure 4.tif

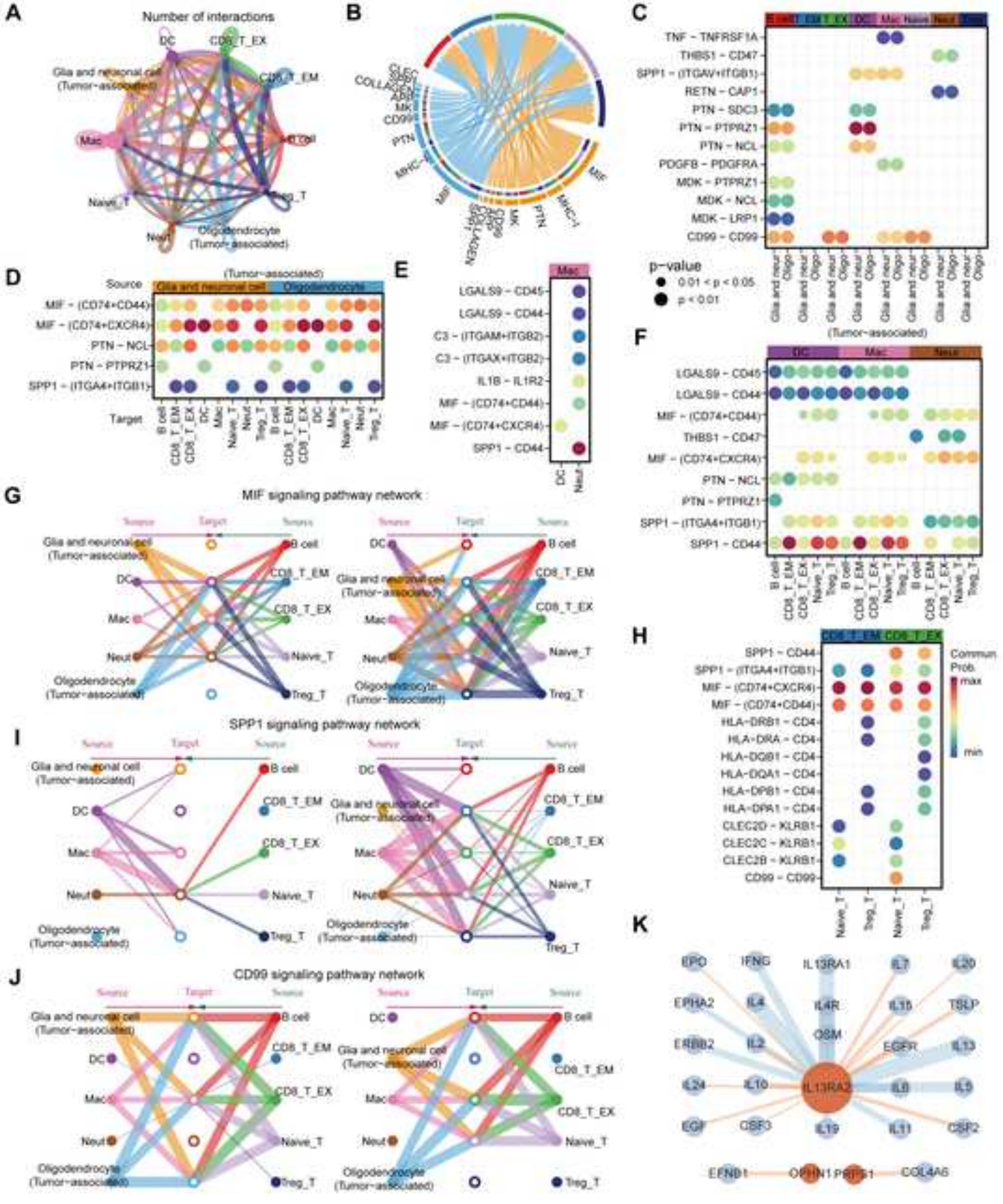

Figure 5 Click here to access/download;Figure;Figure 5.tif

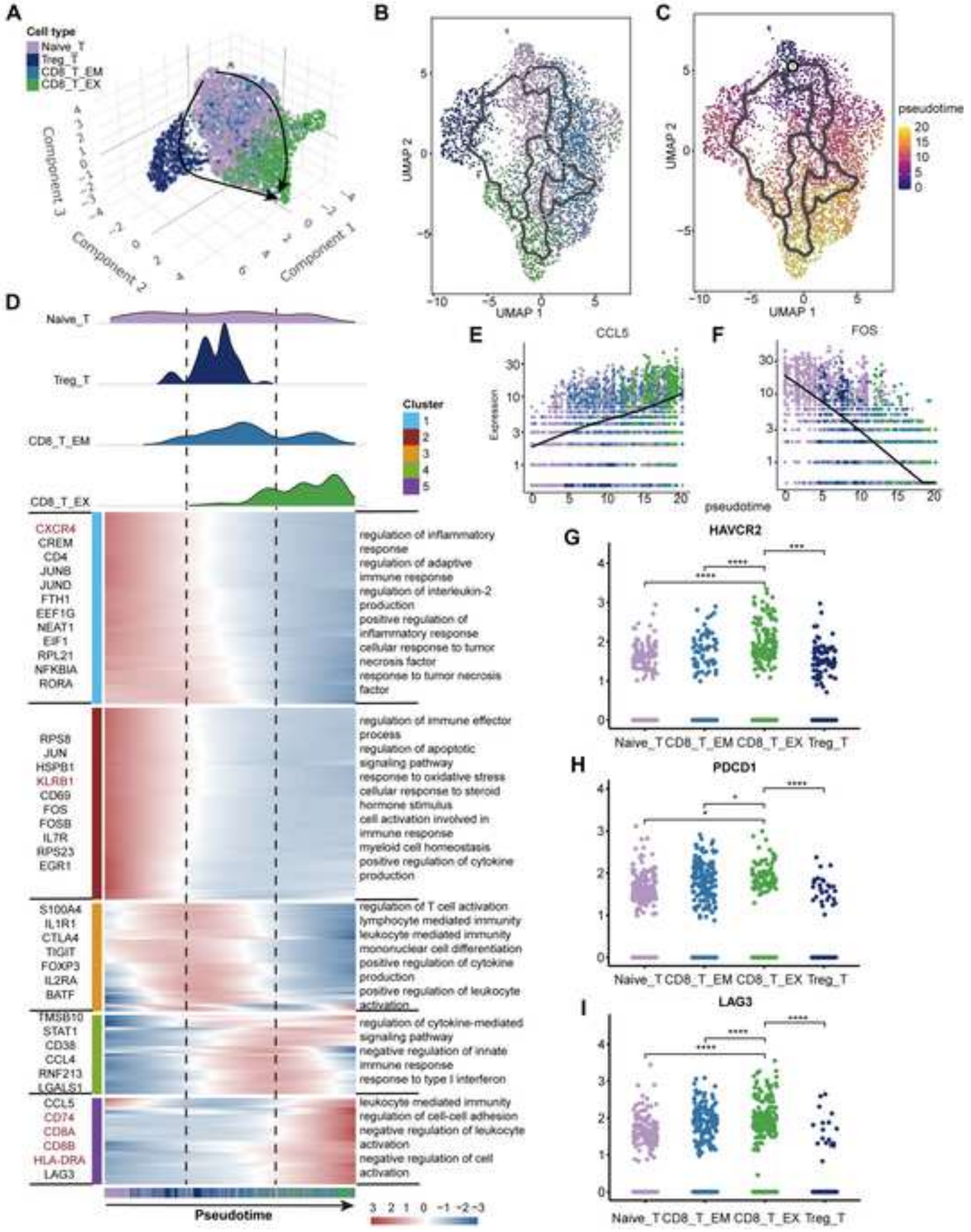

Figure 6

Click here to access/download;Figure;Figure 6.tif

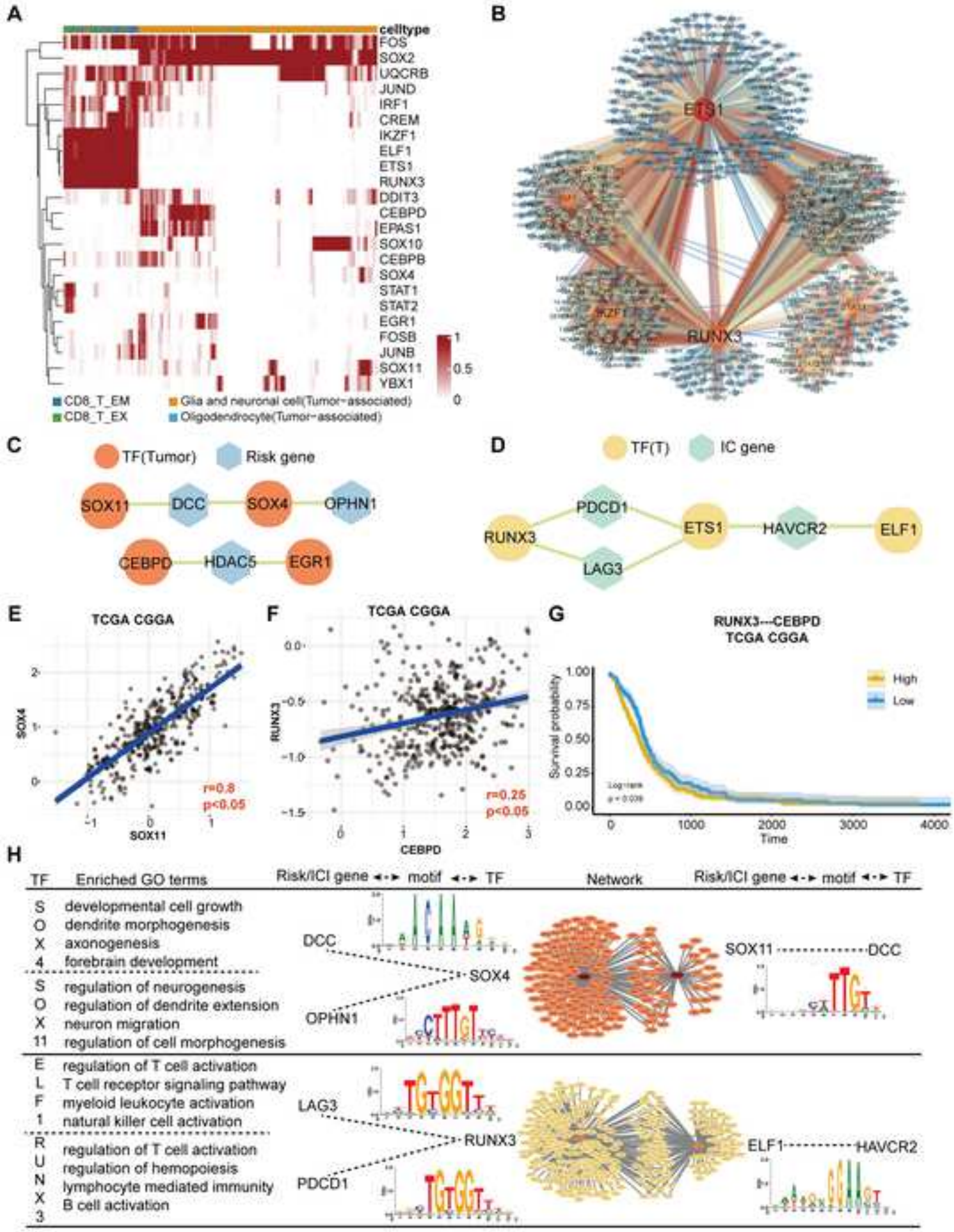

Figure 7

Click here to access/download;Figure;Figure 7.tif

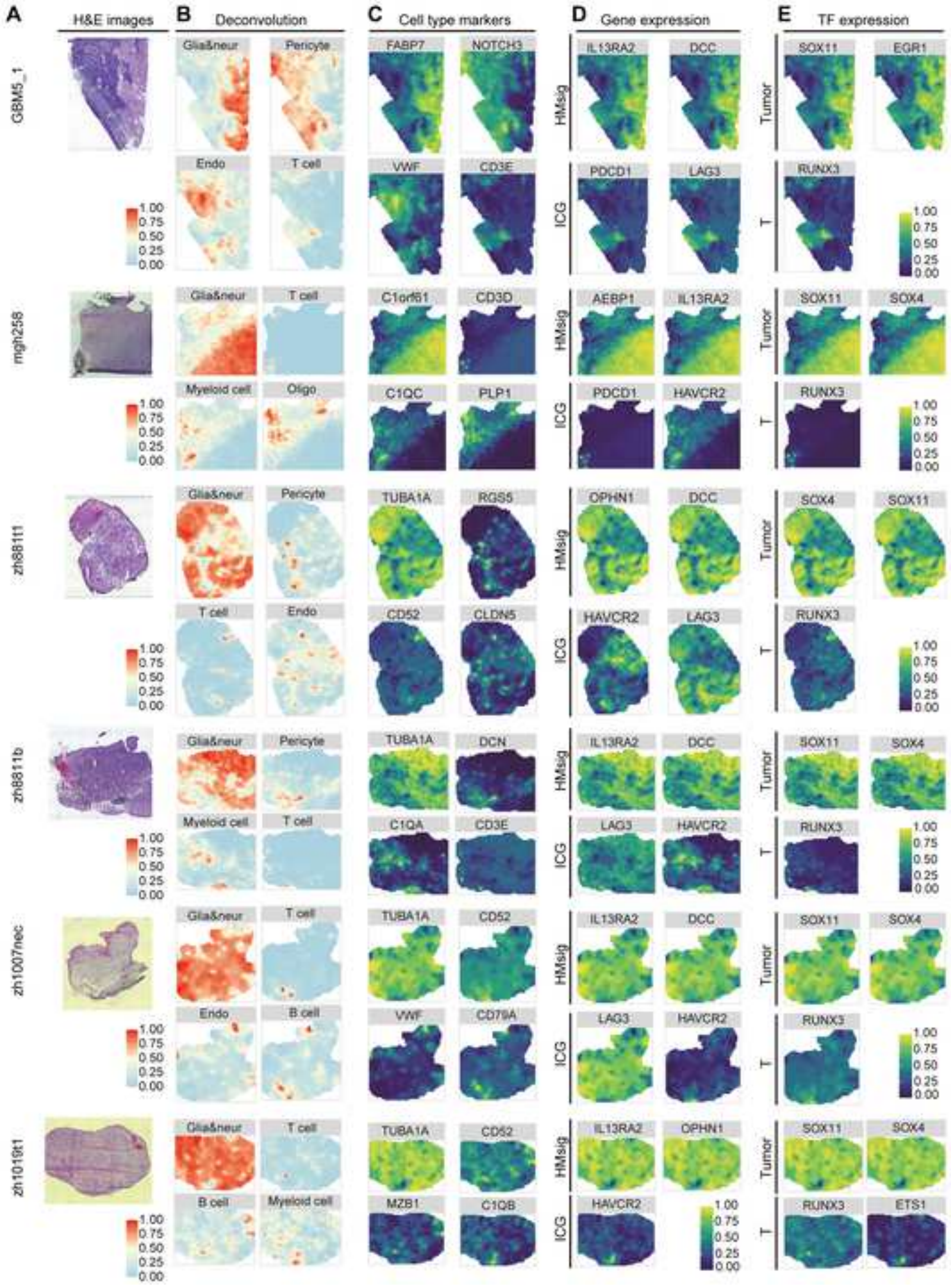

Click here to access/download
Supplementary Material
Responses to Reviewers.docx

Supplementary Figure1

Click here to access/download
Supplementary Material
Supplementary Figure1.pdf

Supplementary Figure2

Click here to access/download
Supplementary Material
Supplementary Figure2.pdf

Supplementary Figure3

Click here to access/download
Supplementary Material
Supplementary Figure3.pdf

Supplementary Figure4

Click here to access/download
Supplementary Material
Supplementary Figure4.pdf

Click here to access/download
Supplementary Material
Supplementary Figure5.pdf

Supplementary Table 1

Click here to access/download
Supplementary Material
Supplementary Table 1.xlsx

Supplementary Table 2

Click here to access/download
Supplementary Material
Supplementary Table 2.xlsx

Supplementary Table 3

Click here to access/download
Supplementary Material
Supplementary Table 3.xlsx

Additional Files

Click here to access/download
Supplementary Material
Additional Files.docx
