# Supplementary material for: Dissecting glioblastoma risk signatures in the tumor immune microenvironment based on multi-dimensional transcriptomics
Source: Gigascience. 2026 Mar 25;15:giag035. doi: 10.1093/gigascience/giag035 (PMC13154832; doi:10.1093/gigascience/giag035)
Supplement: giag035_Supplemental_Files [file Supplementary Figure2.pdf › Supplementary Figure2.pdf]

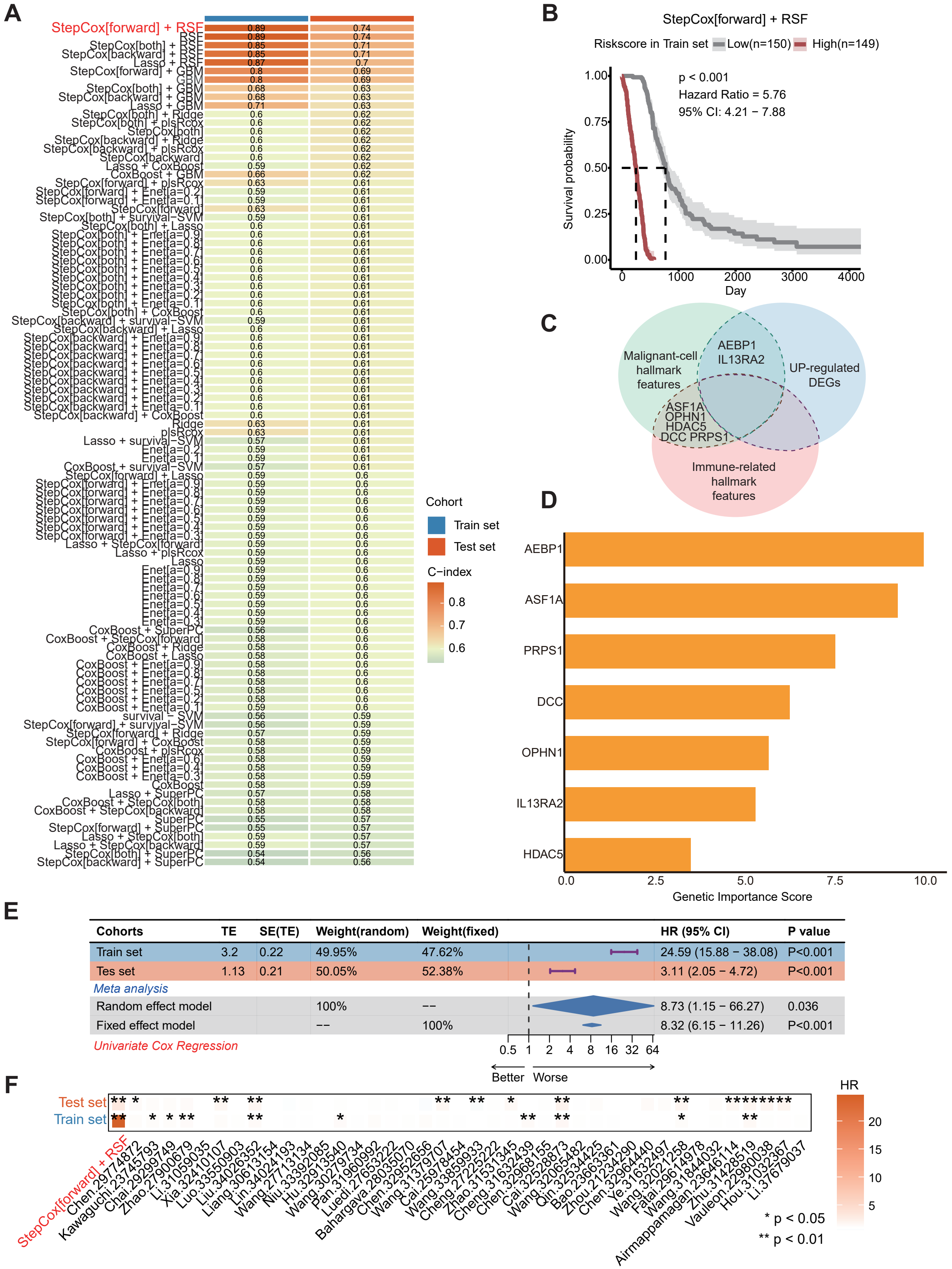
A
StepCox[forward] + RSF
RSF
StepCox[both] + RSF
StepCox[backward] + RSF
Lasso + RSF
StepCox[forward] + GBM
GBM
StepCox[both] + GBM
StepCox[backward] + GBM
Lasso + GBM
Cohort
Train set
Test set
C−index
0.8
0.7
0.6
B
StepCox[forward] + RSF
Riskscore in Train set
Low(n=150)
High(n=149)
p < 0.001
Hazard Ratio = 5.76
95% CI: 4.21 − 7.88
Survival probability
Day
C
Malignant-cell hallmark features
AEBP1
IL13RA2
UP-regulated DEGs
ASF1A
OPHN1
HDAC5
DCC PRPS1
Immune-related hallmark features
D
AEBP1
ASF1A
PRPS1
DCC
OPHN1
IL13RA2
HDAC5
Genetic Importance Score
E
Cohorts | TE | SE(TE) | Weight(random) | Weight(fixed) | HR (95% CI) | P value
Train set | 3.2 | 0.22 | 49.95% | 47.62% | 24.59 (15.88 − 38.08) | P<0.001
Tes set | 1.13 | 0.21 | 50.05% | 52.38% | 3.11 (2.05 − 4.72) | P<0.001
Meta analysis
Random effect model | 100% | −− | 8.73 (1.15 − 66.27) | 0.036
Fixed effect model | −− | 100% | 8.32 (6.15 − 11.26) | P<0.001
Univariate Cox Regression
Better
Worse
F
Test set
Train set
HR
* p < 0.05
** p < 0.01
