# Supplementary material for: Dissecting glioblastoma risk signatures in the tumor immune microenvironment based on multi-dimensional transcriptomics
Source: Gigascience. 2026 Mar 25;15:giag035. doi: 10.1093/gigascience/giag035 (PMC13154832; doi:10.1093/gigascience/giag035)
Supplement: giag035_Authors_Response_To_Reviewer_Comments_original_submission [file giag035_authors_response_to_reviewer_comments_original_submission.pdf]

Author's Response To Reviewer Comments

Dear Editors of GigaScience:
We sincerely thank the Editor and the Reviewers for their careful evaluation and constructive comments on our manuscript, “Dissecting glioblastoma risk signatures in the tumor immune microenvironment based on multi-dimensional transcriptomics” (ID: GIGA-D-26-00037). The insightful comments have significantly helped us to strengthen our study.
First, we added a description of the spermatogenesis pathway in GBM and the mechanism of the OPHN1-EFNB1 interaction, along with relevant references. This analysis has been incorporated into the Results and Discussion sections to enhance the depth of the study and add biological significance to the findings.
Second, to improve the transparency and reproducibility of our methods, we provided a detailed explanation of the machine learning framework and feature selection process, which led to the final selection of 7 core genes (HMsig) from the initial 354 genes. Additionally, key parameters for all computational analyses, including software versions (e.g., Harmony v1.2.1 and inferCNV v1.10.1), clustering resolution, and root cell selection in Monocle3, are clearly documented in the Methods section to ensure the rigor of our analysis.
Next, to further clarify the immune evasion mechanism, the study focuses on the transition of CD8+ effector memory T cells to CD8+ exhausted T cells, which represents a key functional change in the GBM microenvironment. We also used the CARD algorithm for spatial deconvolution to confirm the co-localization of HMsig genes with transcription factors such as SOX4, SOX11, and RUNX3, providing a structural basis for the immunosuppressive environment in GBM.
We believe these revisions comprehensively address the reviewer’s concerns and improve the overall quality of our manuscript. All changes in the revised manuscript are highlighted in red font. The response letter will be attached with the revised manuscript, figures, tables, and supplementary materials. We sincerely thank you and the reviewers for your professional suggestions. Below are our detailed point-by-point responses to the reviewer’s comments:

Reviewer #1
Comment 1: In the first Results section, Gene Set Enrichment Analysis (GSEA) revealed significant enrichment of the "Spermatogenesis" pathway in both single-cell and bulk transcriptomic datasets. Given that GBM is not a tumor of the reproductive system, the biological relevance of this finding is unclear. The authors need to provide a mechanistic explanation or relevant literature support to justify this observation.
Response: We appreciate the reviewer pointing this out. To elucidate the biological significance of the "Spermatogenesis" pathway enrichment in GBM, we have incorporated mechanistic descriptions and supporting literature into the Results section of the revised manuscript. First, evidence indicates that the abnormal expression of spermatogenesis-related genes is strongly associated with glioma prognosis and malignancy grade, which suggests these genes have functional roles beyond reproductive tissues[1]. Second, recent studies suggest that GBM may exploit immune privilege mechanisms by expressing reproductive antigens. This process potentially recruits Tregs that were pre-activated in the reproductive system, and androgens may further support the expression of these genes in GBM[2]. Third, the spermatogenesis-associated gene Sohlh1 has been demonstrated to suppress GBM cell proliferation, migration, and invasion by inhibiting the Wnt/β-catenin signaling pathway[3]. Collectively, these findings suggest that the spermatogenesis pathway play an important role in GBM progression. We have incorporated these points into the Results section.

Based on the comments above, we have revised the manuscript at the appropriate place. The main text is as follows:
Interestingly, the enrichment of the spermatogenesis pathway in GBM suggests that some genes involved in spermatogenesis may play a role beyond their traditional functions in reproductive tissues. Previous studies have indicated that abnormal expression of spermatogenesis-related genes is associated with glioma progression and malignancy (34). In addition, research has shown that GBM may exploit immune privilege mechanisms by expressing reproductive antigens, which could recruit Tregs activated in the

reproductive system (35). Notably, the spermatogenesis gene Sohlh1 has been shown to inhibit GBM cell proliferation and migration by modulating the Wnt/β-catenin signaling pathway (36), further supporting the essential roles of spermatogenesis-related genes in GBM progression. (Page 17-18, Lines 331-341)
Comment 2: In Results 3, the authors report that 42 prognostic-related genes were initially identified through machine learning approaches, but the final HMsig model includes only seven genes. The specific filtering criteria and selection strategy that led from 42 to 7 genes should be explicitly described in the Methods section to improve transparency and reproducibility.
Response: We agree with the reviewer that methods transparency is required in the feature selection process. First, we screened all characteristic genes using univariate Cox regression and Kaplan-Meier (KM) survival analysis, retaining genes with significant prognostic value in both tests ($P < 0.05$), ultimately identifying 42 high-value candidate genes. Next, we evaluated these 42 genes using a combinatorial machine learning algorithm, finding that the "StepCox (forward) + RSF" combination had the highest C-index. Based on this optimal combination, we used StepCox (forward) to select 7 core genes from the 42 candidate genes that contributed most to survival prediction, defined as HMsig. Subsequently, we trained these 7 core genes using the RSF model, ultimately obtaining the best survival prediction performance. This transparent screening process has been detailed in the Methods section of our manuscript.

Based on the comments above, we have revised the manuscript at the appropriate place. The main text is as follows:
To construct a model for predicting the prognosis of GBM patients, we first divided all bulk tumor samples into training and test sets at a ratio of 7:3. Then, we performed univariate Cox regression and Kaplan-Meier (KM) survival analysis on the 354 GBM progression-related feature genes identified across multiple dimensions. Only genes that showed statistically significant prognostic relevance ($P < 0.05$) in both tests were retained, resulting in 42 high-value candidate genes. These pre-screened genes were input into an integrated machine learning framework built using the R package mime1(23).
We constructed and evaluated the model on both the training and test sets using 10-fold cross-validation, selecting the combined model with the highest C-index as the optimal model. Based on this optimal model, we further performed feature screening, ultimately identifying 7 prognostic hallmark characteristic genes (HMsig). To comprehensively evaluate the model, we performed a meta-analysis integrating the training and test sets, and conducted ROC analyses on 1-year, 3-year, and 5-year survival predictions for GBM patients. Model performance was compared with existing methods using C-index and ROC values. Furthermore, we collected three external validation sets from the GEO database to validate the model's performance in predicting patient outcomes (Supplementary Table 1). (Page 10, Lines 181-198)

Comment 3: The study intersects gene sets derived from three analytical dimensions to generate the final feature set used for model construction. The biological rationale and conceptual significance of this intersection strategy should be clarified in the Results section.
Response: We thank the reviewer for this insightful comment. Single-cell hallmark genes capture the intrinsic malignant states of GBM tumor cells; bulk tumor hallmark genes reflect tumor immune cold characteristics; and the bulk DEGs ensure the features have sufficient expression abundance and universality across large patient cohorts. By performing intersection analysis on these three gene sets, we effectively integrated the hallmark features of multidimensional transcriptomic data. This feature gene set represents key genes that can both drive the intrinsic malignant proliferation of tumor cells and regulate the immunosuppressive state of the microenvironment. We have now clarified this intersection strategy in the revised Results section.

Based on the comments above, we have revised the manuscript at the appropriate place. The main text is as follows:
We used a multi-dimensional intersection strategy to identify hallmark-related feature set for prognostic modeling. This approach integrates three key aspects: the intrinsic malignant characteristics of GBM tumor cells captured by single-cell RNA-seq hallmark genes, the immune cold environment features reflected by bulk tumor hallmark genes, and the overall transcriptomic expression universality across patient populations represented by bulk DEGs. By focusing on the overlap of these gene sets, we prioritized genes that are critical not only for the malignant progression of GBM cells but also for shaping the immunosuppressive environment (Figure 3A). (Page 23, Lines 455-463)

Comment 4: In Results 4, the authors report a protein-protein interaction between OPHN1 and the ligand

EFNB1 and suggest its association with patient prognosis. It would strengthen the manuscript if the Discussion section further explored the potential biological mechanisms underlying this interaction in GBM.
Response: We thank the reviewer for this insightful comment. We further investigated the biological mechanisms related to the interaction between EFNB1 and OPHN1. Current evidence identifies EFNB1 as a pivotal driver of glioma cell invasion and a key factor in remodeling the tumor immune microenvironment[4, 5]. The interaction between OPHN1 and EFNB1 likely converges on the Rho-GTPase signaling axis. Specifically, OPHN1 works as a Rho-GAP, which helps control the activity of RhoA and Rac1—both of which are important molecules involved in Ephrin signaling [6]. This interaction seems to help control the structure of the cell, which allows GBM cells to invade and spread more aggressively into the brain tissue. We have expanded our Discussion to explore the mechanistic synergy between OPHN1 and EFNB1.

Based on the comments above, we have revised the manuscript at the appropriate place. The main text is as follows:
Notably, the OPHN1-EFNB1 interaction emerged as a critical determinant of poor prognosis. As EFNB1 drives glioma invasion and immune remodeling(91, 92), its interplay with OPHN1—a Rho-GAP modulating RhoA/Rac1 activity—likely governs cytoskeletal plasticity(93). This synergy facilitates GBM cells to invade and spread more aggressively into the brain tissue. (Page 41, Lines 851-856)

Comment 5: For the T-cell developmental trajectory analysis using Monocle3, the manuscript provides limited methodological detail regarding root state selection and pseudotime initialization. The authors should clarify the computational procedures and specific functions used for trajectory inference and root cell designation in the Methods section.
Response: We thank the reviewer for highlighting the need for technical clarity in our trajectory analysis. To ensure the reproducibility and biological validity of the T-cell developmental inference, we followed a standardized computational procedure in Monocle3. The root of this trajectory selected Naive T cells. We used ‘get_earliest_principal_node’ function to identify the earliest principal node from Naive T cells, which is a helper function provided by monocle3. This node was then designated as the root node using the ‘order_cells’ function to calculate pseudotime values for all cells. We have updated the Methods section to include these specific functions and the underlying computational methods.

Based on the comments above, we have revised the manuscript at the appropriate place. The main text is as follows:
We used ‘get_earliest_principal_node’ function to identify the earliest principal node from Naive T cells, which is a helper function provided by monocle3. This node was then designated as the root node using the ‘order_cells’ function to calculate pseudotime values for all cells. (Page 12, Lines 231-235)

Comment 6: In the "Dimensionality reduction and clustering of cells" section, Harmony was used for batch correction across samples. Considering the availability of multiple single-cell batch integration algorithms, the authors should provide a technical justification for selecting Harmony over alternative approaches.
Response: We appreciate the reviewer’s comment and the opportunity to clarify our methodological choice. We chose Harmony for batch effect integration due to its established performance and computational efficiency, as supported by an independent benchmark study[7]. Harmony was selected over other methods such as Seurat v3 or LIGER for a few reasons. First, Harmony has been shown to effectively integrate datasets with common cell types, minimizing batch effects while preserving biological signals. Second, it demonstrated robustness when applied to diverse sequencing technologies and experimental conditions. Finally, Harmony’s computational efficiency, with a lower runtime and memory footprint compared to other methods, made it particularly suited for our large-scale data analysis. In summary, Harmony provided an optimal balance between accuracy and computational scalability, making it the ideal choice for our study.

Comment 7: Several computational tools (e.g., inferCNV, Harmony, CellMarker2.0) are mentioned without version numbers or corresponding references. To meet reproducibility standards, the Methods section should include detailed version information and appropriate citations.
Response: We thank the reviewer for this insightful comment. In accordance with the reviewer’s request, we have updated the Methods section to include the version information for key software packages and databases used in this study. Furthermore, we have added the references for inferCNV, Harmony, and

CellMarker2.0 to the bibliography. The following table summarizes the updated information:
Tool/Database Version Reference
Harmony v1.2.1 PMID: 31740819
inferCNV v1.10.1 PMID: 24925914
CellMarker2.0 Online Database PMID: 36300619
The Methods section has been revised to include these versions and citations (Page 7-8, Lines 135-136; Page 8, Lines 147; Page 8, Lines 143).

Comment 8: The inferCNV method is commonly applied to epithelial-derived tumors to infer copy number variation patterns. Given the relative scarcity of its application in brain tumors, the authors should provide theoretical justification and relevant references supporting the use of inferCNV in GBM.
Response: We appreciate the reviewer's comment. The method inferCNV was fundamentally inspired by and developed for the study of brain tumors. Single-cell studies in glioblastoma and oligodendroglioma established the theoretical framework for inferring copy number variations (CNVs) from transcriptomic data[8-10]. Furthermore, the official implementation of inferCNV by the Broad Institute uses oligodendroglioma expression data as its primary benchmark and example dataset. The Methods section has been revised to include the descriptions and citations.

Based on the comments above, we have revised the manuscript at the appropriate place. The main text is as follows:
This methodology is particularly robust for GBM as it was fundamentally established and validated through foundational single cell research in brain tumors (20-22). (Page 8, Lines 149-151)

Comment 9: In Results 7, spatial transcriptomic analyses revealed colocalization of certain GBM feature molecules at the tissue level. The Discussion section would benefit from a more comprehensive synthesis of these spatial colocalization patterns and their potential biological implications.
Response: We sincerely thank the reviewers for their valuable suggestions. In the revised text, we emphasize that the spatial co-localization of the HMsig gene and immune checkpoint genes, along with their corresponding transcription factors (such as SOX4, SOX11, and RUNX3), in specific tissue regions represents an immunosuppressive microenvironment. On one hand, SOX4 and SOX11 regulate the expression of the HMsig gene, promoting the malignant progression of GBM. On the other hand, RUNX3 upregulates immune checkpoint genes, thereby promoting immune escape by tumor cells. The synergistic expression of these two types of transcription factors in space jointly promotes the malignant progression of GBM and enhances immune escape capabilities, forming an immunosuppressive microenvironment. We have incorporated these points into the discussion section.

Based on the comments above, we have revised the manuscript at the appropriate place. The main text is as follows:
Spatial transcriptomic analysis further confirmed the co-localization of the HMsig gene with immune checkpoint genes and their transcription factors (especially SOX4, SOX11, and RUNX3) in specific tissue regions, forming an immunosuppressive microenvironment. The co-expression of SOX4 and SOX11 suggests a spatial regulatory mechanism that enhances HMsig gene expression and drives tumor progression. RUNX3 upregulates the expression of immune checkpoint genes LAG3 and PDCD1, further promoting the co-regulation represented by the "double brake" mechanism, thereby facilitating immune escape by tumor cells. This spatial organization driven by a coordinated transcriptional program provides a structural basis for the persistent treatment resistance in GBM. (Page 42-43, Lines 872-882)

Comment 10: Although the Methods section describes clustering and subclustering of major cell types, the specific resolution parameters used for clustering are not reported. These parameters should be clearly stated to enhance methodological transparency.
Response: We thank the reviewer for this constructive suggestion. In our study, the selection of resolution parameters was guided by the clustree package, which allows for the visualization of clustering stability across a range of resolutions (Supplementary Figure 1A-C). A resolution of 0.2 was used to identify major cell lineages. For the detailed subclustering of T cells and myeloid cells, we applied higher resolutions of 0.9 and 0.8, respectively, to capture fine grained cell states. We have updated the Methods section and added Table S2 to clearly document these parameters.

Based on the comments above, we have revised the manuscript at the appropriate place. The main text is as follows:
To ensure the stability and of the clustering results, we evaluated various resolution parameters using the clustree package (version 0.5.1). A resolution of 0.2 was applied to identify major cell lineages. For the subclustering of specific populations, we utilized resolutions of 0.9 for T cells and 0.8 for myeloid cells to resolve fine grained cell subtypes. (Page 8, Lines 137-140)

Reviewer #2
Comment 1: The Methods section does not clearly describe the workflow or key parameters used for subclustering and annotation of T-cell and myeloid cell populations. A detailed account of the analytical pipeline should be provided to enhance reproducibility.
Response: We fully agree with the reviewer that methodological transparency is essential for scientific reproducibility. We have revised the Methods section to provide a comprehensive analytical pipeline used for the subclustering and annotation of T cell and myeloid cell populations. The updated section now explicitly details the following key procedures:
(1) The clustree package was employed to evaluate clustering stability, and we utilized resolutions of 0.9 for T cells and 0.8 for myeloid cells.
(2) We used canonical markers cross referenced with the CellMarker 2.0 database for precise cell annotation. Utilized cell type markers are shown in supplementary figure 1D-E.
To further enhance clarity, we have summarized these key parameters and marker genes in the newly added Table S2. The Methods section has been extensively revised to include these procedural details.

Based on the comments above, we have revised the manuscript at the appropriate place. The main text is as follows:
The complete set of markers used for cell type annotation, along with their corresponding clustering resolutions, is systematically documented (Supplementary Table 2). (Page 8, Lines 143-145)

Comment 2: In Results 2, the authors report concordant enrichment of tumor-associated hallmark pathways at both single-cell and bulk transcriptomic levels. The Discussion section would benefit from a more integrative interpretation of this concordance, particularly in terms of what it implies about transcriptional robustness and malignant program conservation across data modalities.
Response: We thank the reviewer for this suggestion. The five common hallmarks—the E2F targets, G2M checkpoint, spindle mitosis, Wnt signaling, and spermatogenesis—across single-cell and bulk data highlights the transcriptional robustness of the core malignant programs in GBM. These pathways, primarily associated with cell cycle regulation and proliferation, constitute a cross-modal conserved regulatory pattern, avoiding the influence of technical noise inherent in single-modal data. We have updated the Discussion accordingly.

Based on the comments above, we have revised the manuscript at the appropriate place. The main text is as follows:
The five common hallmarks between single cell and bulk dimensions confirms the transcriptional robustness of core malignant programs in GBM. These pathways, primarily associated with cell cycle regulation and proliferation, constitute a cross-modal conserved regulatory pattern, avoiding the influence of technical noise inherent in single-modal data. (Page 40-41, Lines 837-841)

Comment 3: The manuscript should provide appropriate citations and version information for the computational tools and packages used in the study (e.g., Harmony, clusterProfiler). It is essential to meet current standards of methodological transparency.
Response: We thank the reviewer for this important suggestion. In response, we have updated the Methods section to include the version information for all key bioinformatics tools and databases used in

this study (Page 7-8, Lines 135-136; Page 12, Lines 139; Page 11, Lines 217-218; Page 12, Lines 223):
Tool Version Reference
Harmony v1.2.1 PMID: 31740819
clusterProfiler v4.12.0 PMID: 39019974
STRING Online database PMID: 36370105
Cytoscape v3.9.1 PMID: 14597658

Comment 4: In Results 3, the authors analyze the origin the identified HMsig genes. Additional detail is warranted to clarify how these genes emerge from the integrative framework and to discuss the potential biological mechanisms underlying their selection and convergence.
Response: We thank the reviewer for this request for clarification. Our goal was to identify genes that sit at the intersection of three complementary analytical dimensions: hallmark genes from single-cell data capture malignant cell states at high resolution, hallmark genes from bulk cold tumors reflect the immunosuppressive feature, and DEGs from bulk data ensure the selected features are broadly expressed across large patient cohorts. This multi-dimensional filtering helps reduce noise and minimize biases that may exist in any single data type, ensuring that the final HMsig genes are not only biologically relevant but also clinically representative of the GBM microenvironment. We have revised the Results section.

Based on the comments above, we have revised the manuscript at the appropriate place. The main text is as follows:
We used a multi-dimensional intersection strategy to identify hallmark-related feature set for prognostic modeling. This approach integrates three key aspects: the intrinsic malignant characteristics of GBM tumor cells captured by single-cell RNA-seq hallmark genes, the immune cold environment features reflected by bulk tumor hallmark genes, and the overall transcriptomic expression universality across patient populations represented by bulk DEGs. By focusing on the overlap of these gene sets, we prioritized genes that are critical not only for the malignant progression of GBM cells but also for shaping the immunosuppressive environment (Figure 3A).
(Page 23, Lines 455-463)

Comment 5: The machine learning model incorporates SHAP analysis to quantify the contribution of each gene within HMsig. The manuscript would be strengthened by a more explicit discussion of the biological interpretation of these SHAP values—specifically the directionality and magnitude of feature importance.
Response: We thank the reviewer for this suggestion. The SHAP gene importance score quantifies the contribution of each gene in HMsig to the final risk prediction. Analysis showed that genes such as AEBP1, ASF1A, and PRPS1 were the most important and were the main drivers of the prognostic model. In terms of directionality, the SHAP values of HMsig genes were positively correlated with the risk score. This indicates that higher expression levels of these genes are associated with poorer predicted survival outcomes. We have revised the corresponding Results section.

Based on the comments above, we have revised the manuscript at the appropriate place. The main text is as follows:
SHAP analysis revealed that the AEBP1, ASF1A, and PRPS1 genes had the highest SHAP values, indicating their significant impact on survival prediction. These SHAP values suggest that these three genes are major predictors in HMsig, and their direction is consistently positively correlated with patient risk. This confirms that high expression of these HMsig genes is a key factor leading to elevated risk scores and indicates their role as high-risk determinants in the GBM microenvironment. (Page 25, Lines 494-500)

Comment 6: In Results 4, the authors suggest that CD99-CD99 interactions may upregulate IL-6 and TNF-α, thereby promoting tumor survival. Given that IL-6 is traditionally characterized as a pro-inflammatory cytokine, yet is associated with poor prognosis in GBM, a more detailed explanation of this pro-tumorigenic inflammatory mechanism should be incorporated into the Results section.
Response: We thank the reviewers for raising this important point. Previous studies have confirmed the association between high IL6 expression and poor prognosis in GBM patients through analysis of TCGA data[11]. We have further supplemented the description of IL-6 correlation in the results section and added supporting literature.

Based on the comments above, we have revised the manuscript at the appropriate place. The main text is

as follows:
The engagement of CD99 and its ligand can upregulate the expression of IL-6 and TNF-α, which in turn promotes tumor cell proliferation and survival, ultimately correlating with poor clinical outcomes (61, 62). (Page 27, Lines 541-543)

Comment 7: In Results 5, pseudotime analysis identifies two distinct T-cell lineage trajectories. The authors are encouraged to elaborate on the biological significance of this bifurcation and discuss possible drivers.
Response: We thank the reviewer for this suggestion. The bifurcation observed in our pseudotime analysis represents two different trajectories of T cell exhaustion within the GBM microenvironment.
One trajectory (Naive T → Treg → CD8 T EX) represents an immunosuppressive differentiation route, where naive cells may transition through a regulatory state under the influence of suppressive cytokines (e.g. TGFβ) and eventually reaching terminal exhaustion. The other trajectory (Naive T → CD8 T EM → CD8 T EX) reflects a classic exhaustion pathway driven by chronic antigen stimulation, where effector memory cells progressively lose functional capacity. This indicates that T cell exhaustion is a convergent terminal state in the GBM microenvironment. We have expanded the Results section to demonstrate the biological significance of distinct T-lineage trajecories.

Based on the comments above, we have revised the manuscript at the appropriate place. The main text is as follows:
This bifurcation suggests that the exhaustion of T cells in GBM is a convergent process that can be achieved either through an immunosuppressive pathway or through the gradual decline of effector memory cell function. (Page 30, Lines 600-603)

Comment 8: In Results 6, the transcriptional regulatory networks were constructed for CD8_T_EM and CD8_T_EX subpopulations. Beyond their relatively high communication intensity with tumor cells, it remains unclear whether additional biological or analytical considerations informed their selection. The criteria and rationale for focusing on these two subsets should be clearly articulated.
Response: We thank the reviewer for this comment. This study focuses on CD8_T_EM and CD8_T_EX cells not only because these two cell types exhibit strong cellular interactions, but also because these two subsets are involved in anti-tumor immune responses. In the GBM microenvironment, CD8+ T cells transition from an effector memory state to an exhaustion state, leading to a loss of immune killing function. Furthermore, pseudotime trajectory analysis shows that exhaustion is the endpoint of T cell differentiation in the tumor microenvironment. Therefore, researching these two cell types at critical differentiation stages from a transcriptional regulation perspective is crucial. We have updated the Results section.
Based on the comments above, we have revised the manuscript at the appropriate place. The main text is as follows:
To identify transcription factors that drive the expression of HMsig and immune checkpoint molecules, we performed SCENIC analysis on two tumor cell populations and two CD8 T cell subsets exhibiting strong cellular interactions. In addition, we focused specifically on CD8_T_EM and CD8_T_EX cells because they represent a key transition point in the T cell trajectory, where effector memory T cells with tumor-killing functions shift into exhausted T cells. This transition is highly relevant to the immune escape mechanisms in GBM. (Page 33, Lines 674-680)

Comment 9: In Results 7, the authors apply the CARD algorithm for spatial transcriptomic deconvolution to infer cell-type composition at the tissue level. A methodological justification should be provided, highlighting the advantages of CARD relative to other spatial deconvolution tools in the context of this dataset.
Response: We thank the reviewer for justifying our selection of the CARD algorithm. Unlike traditional reference-based methods, CARD utilizes a conditional autoregressive (CAR) model to resolve the inherent spatial information within tissue structures. In the context of GBM, the distribution of cell types is typically organized into specific microenvironmental structures. This spatial autoregressive prior can accurately estimate cell proportions by considering the similarity between neighboring spots, thus providing a more precise spatial characterization of the GBM microenvironment.

Comment 10: The spatial transcriptomic analysis reveals consistent colocalized expression of tumor-

associated transcription factors SOX4 and SOX11 across samples, supporting their regulatory role in HMsig activation. The Discussion section should further explore the potential regulatory implications of this spatial co-expression pattern and its relevance to tumor progression.
Response: We thank the reviewer for this insightful comment. SOX4 and SOX11, as transcriptional factors of HMsig genes, are co-expressed in spatial tissues and play a crucial regulatory role in enhancing HMsig expression, further promoting the malignant progression of GBM. Additionally, other transcription factors, such as RUNX3, that regulate immune checkpoint genes, also contribute to the regulatory network in GBM microenvironment. We have revised the Discussion section to demonstrate the regulatory role of both tumor-associated transcription factors and T-cell-related transcription factors in the spatial co-expression patterns.

Based on the comments above, we have revised the manuscript at the appropriate place. The main text is as follows:
Spatial transcriptomic analysis further confirmed the co-localization of the HMsig genes with ICGs and their transcription factors (especially SOX4, SOX11, and RUNX3) in specific tissue regions, forming an immunosuppressive microenvironment. The co-expression of SOX4 and SOX11 suggests a spatial regulatory mechanism that enhances HMsig genes expression and drives tumor progression. RUNX3 upregulates the expression of immune checkpoint molecules LAG3 and PDCD1, further promoting the co-regulation represented by the “dual-brake” mechanism, thereby facilitating immune escape by tumor cells. This spatial organization driven by a coordinated transcriptional program provides a structural basis for the persistent treatment resistance in GBM. (Page 42, Lines 872-881)

Reviewer #3
Comment 1: The abstract mentions the pivotal roles of hallmarks of malignancy and cell cycle regulatory pathways in transcriptional regulation, yet fails to specify the exact mechanisms or functions. A concise description of these critical roles would enhance clarity and impact.
Response: We thank the reviewer for this constructive suggestion. We have revised the abstract to specify that these pathways are essential for promoting tumor cell proliferation and progression.

Based on the comments above, we have revised the manuscript at the appropriate place. The main text is as follows:
Hallmarks of malignancy and cell cycle regulatory pathways were consistently enriched across these modalities, promoting tumor cell proliferation and progression. (Page 1, Lines 15-17)

Comment 2: At present, the abstract appears disconnected without clear transitional logic; revision to establish explicit linkages between sentences is recommended.
Response: We thank the reviewer for this constructive suggestion. We have added linkages that connect the initial multi-scale data integration to the subsequent identification of core malignant pathways, the implementation of our machine learning framework, and the final spatial validation. These transitions emphasize how each analytical layer builds upon the biological findings of the previous stage, ensuring a more cohesive narrative that reflects our integrative methodology.

Based on the comments above, we have revised the manuscript at the appropriate place. The main text is as follows:
Glioblastoma (GBM) is characterized by pronounced tumor heterogeneity and a complex immune microenvironment, contributing to poor patient survival outcomes. In this study, we comprehensively

dissected the tumor microenvironment (TME) and uncovered potential molecular mechanisms by integrating single-cell, bulk, and spatial transcriptomic data. Hallmarks of malignancy and cell cycle regulatory pathways were consistently enriched across these modalities, promoting tumor cell proliferation and progression. Using machine learning algorithm, we identified seven hallmark-related prognostic signatures (HMsig), namely AEBP1, ASF1A, PRPS1, DCC, OPHN1, IL13RA2, and HDAC5—whose predictive importance was validated through SHAP analysis. Ligand-receptor (LR) interaction analysis further revealed that interactions involving OPHN1 were associated with poorer prognosis. Along the pseudotime trajectory of T-cell differentiation, immune checkpoint genes (ICGs) LAG3, PDCD1, and HAVCR2 were substantially upregulated. Notably, synergistic transcriptional regulation between tumor-related HMsig genes and ICGs in T cells was identified as a key factor influencing patient survival. Spatial transcriptomic analysis demonstrated the existence of synergistic gene interactions, deciphering the immunomodulatory functions of GBM biomarkers within the TME. (Page 1-2, Lines 11-27)

Comment 3: The keywords should represent the core, recurrent themes throughout the entire study rather than terms mentioned only sporadically at the beginning or conclusion.
Response: We sincerely thank the reviewer for this constructive suggestion regarding the selection of keywords. We agree that keywords should summarize the core methods and biological themes that run through the entire study.

Based on the comments above, we have revised the manuscript at the appropriate place. The main text is as follows:
Glioblastoma; Single-cell transcriptomics; Spatial transcriptomics; Tumor microenvironment; Machine learning; Prognostic signature; Immune escape. (Page 1-2, Lines 28-29)

Comment 4: In the Introduction, the authors identify multiple factors contributing to the poor therapeutic outcomes in glioma, among which the 'characteristics of the tumor's physical microenvironment' is vaguely delineated and lacks conceptual precision.
Response: We sincerely thank the reviewer for this constructive suggestion. We further explored the impact of GBM physical microenvironment characteristics on poor treatment efficacy. The efficacy of most small molecule chemotherapy drugs is limited by the blood-brain barrier (BBB), which makes it difficult for drugs to penetrate the walls of brain blood vessels. This may be the main reason for the poor results in many clinical trials of blood-borne drugs. We have revised the Introduction section to specify biophysical characteristics in GBM.

Based on the comments above, we have revised the manuscript at the appropriate place. The main text is as follows:
Additionally, the efficacy of most small-molecule chemotherapy drugs is limited by the unique structure of brain blood vessels—the blood-brain barrier (BBB), which strictly regulates the homeostasis of the central nervous system. The inability of drugs to cross the walls of brain blood vessels is likely a major reason for the poor results in many clinical trials of blood-borne drugs (4). (Page 3, Line 41-52)

Comment 5: Within the subsection 'Immune phenotype of tumors based on transcriptomic profiling,' the initial two sentences comprise background material that is incongruous with the Methods section. The authors are advised to relocate this content to the Introduction to preserve the logical organization of the manuscript.
Response: We thank the reviewer for this constructive advice. We agree that the definitions of the immune “hot” and “cold” phenotypes are more appropriate for the introduction, as they provide necessary background information for the theoretical basis of our research. Therefore, we have moved these background definitions to the Introduction section and removed them from the Methods section.

Based on the comments above, we have revised the manuscript at the appropriate place. The main text is as follows:
Various studies support the hypothesis that GBM is an immunologically “cold” tumor(3). Unlike immune hot tumors, which show extensive immune infiltration and active anti-tumor immunity, cold tumors are defined by an absence of these responses and weakened immune defenses. (Page 3, Line 44-48)

Comment 6: In the Results section, certain findings lack corresponding textual description; for instance,

Figure 1B is presented without adequate characterization in the narrative.
Response: We thank the reviewer for this observation. In response, we have added a description to the Results section specifying that the annotated cell clusters show highly specific expression of canonical lineage markers, providing robust molecular support for our cell type identification in the GBM microenvironment.

Based on the comments above, we have revised the manuscript at the appropriate place. The main text is as follows:
Canonical marker genes exhibited high expression specificity within their corresponding cell lineages, confirming the accuracy of cell type annotation (Figure 1B, Supplementary Table 2). (Page 16, Lines 304-306)

Comment 7: The Methods describe T cells and B cells as reference populations for inferring copy number variation, whereas the Results define highly malignant cells as those with above-average CNV scores within two neuronal subtypes. The former strategy is conventionally accepted; however, the latter lacks explicit methodological justification. Furthermore, the relationship between these two approaches—whether complementary or conflicting—requires detailed elucidation.
Response: We appreciate the valuable feedback from the reviewer. The method described in this paper identifies highly malignant cell populations in GBM based on the mean CNV score of all cells. The specific workflow is as follows: First, using T cells and B cells as reference normal cell populations, we select malignant cell lineages (including glia/neuronal cells and oligodendrocytes) as the observation set and calculate the CNV score of all cell populations. Second, we set the average CNV score as a threshold to divide the observation cell population into highly malignant and low-malignant subpopulations. This method enables more refined analysis of core hallmark features in genomically unstable tumor cells. We have updated the methods section.

Based on the comments above, we have revised the manuscript at the appropriate place. The main text is as follows:
In the single-cell transcriptome data, we used T cells and B cells as the reference set to calculate the CNV score for all cells. Oligodendrocytes and glia/neuronal cells were used as sources of malignant cells in the observation set to identify highly malignant cells. We defined the two cell types with CNV scores exceeding the mean as highly malignant cells. (Page 8, Lines 151-155)

Comment 8: In Results section 2, the authors report that B-cell infiltration does not differ significantly between immune-hot and immune-cold tumors. Does this observation suggest that B cells may play a dual or context-dependent role within the GBM microenvironment? The authors are encouraged to provide further interpretation and discussion to clarify this point.
Response: We thank the reviewer for this valuable comment. The immune infiltration analysis shows no significant difference in B cell infiltration between immune hot and immune cold tumors. This suggests that B cells may play different functional roles in distinct microenvironments. Notably, the enrichment of B cells in immune cold microenvironments suggests that B cells may have an immunosuppressive function rather than promoting an active immune response. Previous studies have shown that B cells can directly suppress the activity of CD8+ T cells and recruit Tregs, thereby promoting tumor progression[12]. We have highlighted this point in the revised Results section.

Based on the comments above, we have revised the manuscript at the appropriate place. The main text is as follows:
Regarding the enrichment phenomenon of B cells in immune "cold" tumors, it may be associated with an immunosuppressive function(45, 46). This observation suggests that B cell activity in GBM is context dependent, potentially contributing to a pro-tumorigenic environment rather than an active anti-tumor response in specific TME. (Page 20-21, Lines 397-400)

Comment 9: The authors should explicitly define the 'combined score' depicted in Figure 4K, including its constituent variables and the algorithm or formula used for its calculation.
Response: We thank the reviewer for this valuable comment. The combined score shown in Figure 4K is the standard confidence metric utilized by the STRING database. The STRING database integrates different types of interaction evidence, assigns weights to each type, and finally calculates a combined score to

evaluate the reliability of the protein-protein interaction. We have updated the legend for Figure 4K to concisely define this metric.

Based on the comments above, we have revised the manuscript at the appropriate place. The main text is as follows:
PPI networks between HMsig genes and LR pairs. Orange dots represent HMsig genes, blue dots represent LR pairs, and the color and thickness of the lines are determined by the combined score. The combined score is the standardized confidence metric to evaluate the PPI from the STRING database. (Page 29, Lines 586-589)

Comment 10: The T cell differentiation trajectory in Results section 5 appears poorly defined in two-dimensional space. Please provide additional annotation to clarify the inferred developmental path.
Response: We thank the reviewer for this suggestion. Figure 5A provides a three-dimensional developmental trajectory of T cells, with directional arrows illustrating the differentiation trend. Figure 5C utilizes continuous pseudo-time values to define the T cell differentiation trajectory in two-dimensional space. The transition from the dark purple root (representing naive T cells) to the yellow terminal state (representing CD8_T_EX cells) clearly presents the differentiation process. The black principal tree line in Figures 5B and 5C further marks specific branching points. We have updated the results section to clarify the specific differentiation trajectory paths.

Based on the comments above, we have revised the manuscript at the appropriate place. The main text is as follows:
One trajectory commenced with naive T cells, passed through Treg cells, and ended at CD8_T_EX cells; the other started from naive T cells, proceeded through CD8_T_EM cells, and terminated at CD8_T_EX cells (Figure 5A, Supplementary Figure 3B). (Page 30, Lines 597-600)

Respectfully yours,
Conxue Hu, Ph.D, Yunpeng Zhang, Ph.D;
College of Bioinformatics Science and Technology
Harbin Medical University
194 Xuefu Road, Harbin 150081, China
Phone: 86-451-86615922
Fax: 86-451-86615922
Email: hucx1996@hrbmu.edu.cn, zhangyp@hrbmu.edu.cn;

References
1. Wang H, Wang X, Xu L, Zhang J, Cao H. Analysis of the Egfr Amplification and Cdkn2a Deletion Regulated Transcriptomic Signatures Reveals the Prognostic Significance of Spats2l in Patients with Glioma. Front Oncol. 2021;11:551160.
2. Sharpe MA, Baskin DS, Jenson AV, Baskin AM. Hijacking Sexual Immuno-Privilege in Gbm-an Immuno-Evasion Strategy. Int J Mol Sci. 2021;22(20).
3. Liu X, Gao Q, Zhao N, Zhang X, Cui W, Sun J, Fu J, Hao J. Sohlh1 Suppresses Glioblastoma Cell Proliferation, Migration, and Invasion by Inhibition of Wnt/Beta-Catenin Signaling. Mol Carcinog.

2018;57(4):494-502.
4. Shi Y, Sun Y, Cheng H, Wang C. Efnb1 Acts as a Novel Prognosis Marker in Glioblastoma through Bioinformatics Methods and Experimental Validation. J Oncol. 2021;2021:4701680.
5. Zheng Y, Shi J. Efnb1 Drives Glioma Progression and Shapes the Immune Microenvironment: A Potential Prognostic Biomarker. Discov Oncol. 2025;16(1):249.
6. Cho HJ, Hwang YS, Yoon J, Lee M, Lee HG, Daar IO. Ephrinb1 Promotes Cancer Cell Migration and Invasion through the Interaction with Rhogdi1. Oncogene. 2018;37(7):861-872.
7. Tran HTN, Ang KS, Chevrier M, Zhang X, Lee NYS, Goh M, Chen J. A Benchmark of Batch-Effect Correction Methods for Single-Cell Rna Sequencing Data. Genome Biol. 2020;21(1):12.
8. Venteicher AS, Tirosh I, Hebert C, Yizhak K, Neftel C, Filbin MG, Hovestadt V, Escalante LE, Shaw ML, Rodman C, Gillespie SM, Dionne D, Luo CC, Ravichandran H, Mylvaganam R, Mount C, Onozato ML, Nahed BV, Wakimoto H, Curry WT, Iafrate AJ, Rivera MN, Frosch MP, Golub TR, Brastianos PK, Getz G, Patel AP, Monje M, Cahill DP, Rozenblatt-Rosen O, Louis DN, Bernstein BE, Regev A, Suva ML. Decoupling Genetics, Lineages, and Microenvironment in Idh-Mutant Gliomas by Single-Cell Rna-Seq. Science. 2017;355(6332).
9. Tirosh I, Venteicher AS, Hebert C, Escalante LE, Patel AP, Yizhak K, Fisher JM, Rodman C, Mount C, Filbin MG, Neftel C, Desai N, Nyman J, Izar B, Luo CC, Francis JM, Patel AA, Onozato ML, Riggi N, Livak KJ, Gennert D, Satija R, Nahed BV, Curry WT, Martuza RL, Mylvaganam R, Iafrate AJ, Frosch MP, Golub TR, Rivera MN, Getz G, Rozenblatt-Rosen O, Cahill DP, Monje M, Bernstein BE, Louis DN, Regev A, Suva ML. Single-Cell Rna-Seq Supports a Developmental Hierarchy in Human Oligodendroglioma. Nature. 2016;539(7628):309-313.
10. Ahrends R, Ota A, Kovary KM, Kudo T, Park BO, Teruel MN. Controlling Low Rates of Cell Differentiation through Noise and Ultrahigh Feedback. Science. 2014;344(6190):1384-1389.
11. Lamano JB, Lamano JB, Li YD, DiDomenico JD, Choy W, Veliceasa D, Oyon DE, Fakurnejad S, Ampie L, Kesavabhotla K, Kaur R, Kaur G, Biyashev D, Unruh DJ, Horbinski CM, James CD, Parsa AT, Bloch O. Glioblastoma-Derived Il6 Induces Immunosuppressive Peripheral Myeloid Cell Pd-L1 and Promotes Tumor Growth. Clin Cancer Res. 2019;25(12):3643-3657.
12. Yang C, Lee H, Pal S, Jove V, Deng J, Zhang W, Hoon DS, Wakabayashi M, Forman S, Yu H. B Cells Promote Tumor Progression Via Stat3 Regulated-Angiogenesis. PLoS One. 2013;8(5):e64159.

Editorial Manager®

Cookie Preference Center

We use cookies which are necessary to make our site work. We may also use additional cookies to analyze, improve and personalize our content and your digital experience. For more information, see our Cookie Policy.

You may choose not to allow some types of cookies. However, blocking some types may impact your experience of our site and the services we are able to offer. See the different category headings below to find out more or change your settings.

Allow all

Manage Consent Preferences

Strictly Necessary Cookies

Always active

These cookies are necessary for the website to function and cannot be switched off in our systems. They are usually only set in response to actions made by you which amount to a request for services, such as setting your privacy preferences, logging in or filling in forms. You can set your browser to block or alert you about these cookies, but some parts of the site will not then work.

Functional Cookies

These cookies enable the website to provide enhanced functionality and personalisation. They may be set by us or by third party providers whose services we have added to our pages. If you do not allow these cookies then some or all of these services may not function properly.

Performance Cookies

These cookies allow us to count visits and traffic sources so we can measure and improve the performance of our site. They help us to know which pages are the most and least popular and see how visitors move around the site.

Targeting Cookies

These cookies may be set through our site by our advertising partners. They may be used by those companies to build a profile of your interests and show you relevant adverts on other sites. If you do not allow these cookies, you will experience less targeted advertising.

Cookie List

Clear

Apply Cancel

Consent Leg.Interest

Confirm my choices

Powe
