# Supplementary material for: Dissecting glioblastoma risk signatures in the tumor immune microenvironment based on multi-dimensional transcriptomics
Source: Gigascience. 2026 Mar 25;15:giag035. doi: 10.1093/gigascience/giag035 (PMC13154832; doi:10.1093/gigascience/giag035)
Supplement: giag035_Reviewer_1_Report_original_submission — Reviewer 1 -- 2/15/2026 [file giag035_reviewer_1_report_original_submission.pdf]

Reviewer Report

Title: Dissecting glioblastoma risk signatures in the tumor immune microenvironment based on multi-dimensional transcriptomics

Version: Original Submission **Date:** 2/15/2026

Reviewer name: Reviewer 1

Reviewer Comments to Author:

The study integrates single-cell transcriptomics, bulk RNA-seq, and spatial transcriptomics to characterize the heterogeneity of the glioblastoma tumor microenvironment and to explore its underlying molecular mechanisms. The authors first identified hallmark features of glioblastoma and constructed machine learning models, ultimately deriving a seven-gene prognostic signature (HMsig). The authors further performed cell-cell communication analysis, pseudotime trajectory analysis, and transcription factor regulatory network inference to comprehensively delineate the tumor microenvironment and to interpret the biological and clinical relevance of their findings. Finally, spatial transcriptomic data were used to validate the identified molecular features through colocalization analyses.

The following issues merit further clarification and elaboration:

1. In the first Results section, Gene Set Enrichment Analysis (GSEA) revealed significant enrichment of the "Spermatogenesis" pathway in both single-cell and bulk transcriptomic datasets. Given that GBM is not a tumor of the reproductive system, the biological relevance of this finding is unclear. The authors need to provide a mechanistic explanation or relevant literature support to justify this observation.
2. In Results 3, the authors report that 42 prognostic-related genes were initially identified through machine learning approaches, but the final HMsig model includes only seven genes. The specific filtering criteria and selection strategy that led from 42 to 7 genes should be explicitly described in the Methods section to improve transparency and reproducibility.
3. The study intersects gene sets derived from three analytical dimensions to generate the final feature set used for model construction. The biological rationale and conceptual significance of this intersection strategy should be clarified in the Results section.
4. In Results 4, the authors report a protein-protein interaction between OPHN1 and the ligand EFNB1 and suggest its association with patient prognosis. It would strengthen the manuscript if the Discussion section further explored the potential biological mechanisms underlying this interaction in GBM.
5. For the T-cell developmental trajectory analysis using Monocle3, the manuscript provides limited methodological detail regarding root state selection and pseudotime initialization. The authors should clarify the computational procedures and specific functions used for trajectory inference and root cell designation in the Methods section.
6. In the "Dimensionality reduction and clustering of cells" section, Harmony was used for batch correction across samples. Considering the availability of multiple single-cell batch integration algorithms, the authors should provide a technical justification for selecting Harmony over alternative approaches.

7. Several computational tools (e.g., inferCNV, Harmony, CellMarker2.0) are mentioned without version numbers or corresponding references. To meet reproducibility standards, the Methods section should include detailed version information and appropriate citations.
8. The inferCNV method is commonly applied to epithelial-derived tumors to infer copy number variation patterns. Given the relative scarcity of its application in brain tumors, the authors should provide theoretical justification and relevant references supporting the use of inferCNV in GBM.
9. In Results 7, spatial transcriptomic analyses revealed colocalization of certain GBM feature molecules at the tissue level. The Discussion section would benefit from a more comprehensive synthesis of these spatial colocalization patterns and their potential biological implications.
10. Although the Methods section describes clustering and subclustering of major cell types, the specific resolution parameters used for clustering are not reported. These parameters should be clearly stated to enhance methodological transparency.

Methods

Are the methods appropriate to the aims of the study, are they well described, and are necessary controls included? Choose an item.

Conclusions

Are the conclusions adequately supported by the data shown? Choose an item.

Reporting Standards

Does the manuscript adhere to the journal's guidelines on [minimum standards of reporting?] Choose an item.

Choose an item.

Statistics

Are you able to assess all statistics in the manuscript, including the appropriateness of statistical tests used? Choose an item.

Quality of Written English

Please indicate the quality of language in the manuscript: Choose an item.

Declaration of Competing Interests

Please complete a declaration of competing interests, considering the following questions:

- Have you in the past five years received reimbursements, fees, funding, or salary from an organisation that may in any way gain or lose financially from the publication of this manuscript, either now or in the future?

- Do you hold any stocks or shares in an organisation that may in any way gain or lose financially from the publication of this manuscript, either now or in the future?
- Do you hold or are you currently applying for any patents relating to the content of the manuscript?
- Have you received reimbursements, fees, funding, or salary from an organization that holds or has applied for patents relating to the content of the manuscript?
- Do you have any other financial competing interests?
- Do you have any non-financial competing interests in relation to this paper?

If you can answer no to all of the above, write 'I declare that I have no competing interests' below. If your reply is yes to any, please give details below.

I declare that I have no competing interests

I agree to the open peer review policy of the journal. I understand that my name will be included on my report to the authors and, if the manuscript is accepted for publication, my named report including any attachments I upload will be posted on the website along with the authors' responses. I agree for my report to be made available under an Open Access Creative Commons CC-BY license (http://creativecommons.org/licenses/by/4.0/). I understand that any comments which I do not wish to be included in my named report can be included as confidential comments to the editors, which will not be published.

I agree to the open peer review policy of the journal

To further support our reviewers, we have joined with Publons, where you can gain additional credit to further highlight your hard work (see: https://publons.com/journal/530/gigascience). On publication of this paper, your review will be automatically added to Publons, you can then choose whether or not to claim your Publons credit. I understand this statement.

Yes Choose an item.
