# Supplementary material for: Dissecting glioblastoma risk signatures in the tumor immune microenvironment based on multi-dimensional transcriptomics
Source: Gigascience. 2026 Mar 25;15:giag035. doi: 10.1093/gigascience/giag035 (PMC13154832; doi:10.1093/gigascience/giag035)
Supplement: giag035_Reviewer_2_Report_original_submission — Reviewer 2 -- 2/17/2026 [file giag035_reviewer_2_report_original_submission.pdf]

Reviewer Report

Title: Dissecting glioblastoma risk signatures in the tumor immune microenvironment based on multi-dimensional transcriptomics

Version: Original Submission **Date:** 2/17/2026

Reviewer name: Reviewer 2

Reviewer Comments to Author:

This study proposes an analytical framework integrating multi-dimensional transcriptomic data to dissect the tumor microenvironment of glioblastoma. Through hallmark enrichment analyses at both single-cell and bulk transcriptomic levels, the authors identify malignant features of glioblastoma. Building upon these findings, a machine learning model was developed to predict patient survival, leading to the identification of prognostic signature (HMsig: AEBP1, ASF1A, PRPS1, DCC, OPHN1, IL13RA2, and HDAC5). The study further leverages single-cell RNA-seq data to investigate the molecular mechanisms underlying tumor microenvironment heterogeneity, employing cell-cell communication analysis, pseudotime trajectory inference, and transcription factor regulatory network analysis to elucidate the functional role of HMsig within the microenvironment. Finally, spatial transcriptomics data were integrated to map these molecular features onto tissue architecture, and spatial colocalization analyses were conducted to validate their expression patterns in situ.
While the study is comprehensive and methodologically rich, several issues require clarification or further elaboration:
1. The Methods section does not clearly describe the workflow or key parameters used for subclustering and annotation of T-cell and myeloid cell populations. A detailed account of the analytical pipeline should be provided to enhance reproducibility.
2. In Results 2, the authors report concordant enrichment of tumor-associated hallmark pathways at both single-cell and bulk transcriptomic levels. The Discussion section would benefit from a more integrative interpretation of this concordance, particularly in terms of what it implies about transcriptional robustness and malignant program conservation across data modalities.
3. The manuscript should provide appropriate citations and version information for the computational tools and packages used in the study (e.g., Harmony, clusterProfiler). It is essential to meet current standards of methodological transparency.
4. In Results 3, the authors analyze the origin the identified HMsig genes. Additional detail is warranted to clarify how these genes emerge from the integrative framework and to discuss the potential biological mechanisms underlying their selection and convergence.
5. The machine learning model incorporates SHAP analysis to quantify the contribution of each gene within HMsig. The manuscript would be strengthened by a more explicit discussion of the biological interpretation of these SHAP valuesâ€”specifically the directionality and magnitude of feature importance.
6. In Results 4, the authors suggest that CD99-CD99 interactions may upregulate IL-6 and TNF-Î±, thereby promoting tumor survival. Given that IL-6 is traditionally characterized as a pro-inflammatory

cytokine, yet is associated with poor prognosis in GBM, a more detailed explanation of this pro-tumorigenic inflammatory mechanism should be incorporated into the Results section.
7. In Results 5, pseudotime analysis identifies two distinct T-cell lineage trajectories. The authors are encouraged to elaborate on the biological significance of this bifurcation and discuss possible drivers.
8. In Results 6, the transcriptional regulatory networks were constructed for CD8_T_EM and CD8_T_EX subpopulations. Beyond their relatively high communication intensity with tumor cells, it remains unclear whether additional biological or analytical considerations informed their selection. The criteria and rationale for focusing on these two subsets should be clearly articulated.
9. In Results 7, the authors apply the CARD algorithm for spatial transcriptomic deconvolution to infer cell-type composition at the tissue level. A methodological justification should be provided, highlighting the advantages of CARD relative to other spatial deconvolution tools in the context of this dataset.
10. The spatial transcriptomic analysis reveals consistent colocalized expression of tumor-associated transcription factors SOX4 and SOX11 across samples, supporting their regulatory role in HMsig activation. The Discussion section should further explore the potential regulatory implications of this spatial co-expression pattern and its relevance to tumor progression.

Methods

Are the methods appropriate to the aims of the study, are they well described, and are necessary controls included? Choose an item.

Conclusions

Are the conclusions adequately supported by the data shown? Choose an item.

Reporting Standards

Does the manuscript adhere to the journal's guidelines on [minimum standards of reporting?]() Choose an item.

Choose an item.

Statistics

Are you able to assess all statistics in the manuscript, including the appropriateness of statistical tests used? Choose an item.

Quality of Written English

Please indicate the quality of language in the manuscript: Choose an item.

Declaration of Competing Interests

Please complete a declaration of competing interests, considering the following questions:

- Have you in the past five years received reimbursements, fees, funding, or salary from an organisation that may in any way gain or lose financially from the publication of this manuscript, either now or in the future?
- Do you hold any stocks or shares in an organisation that may in any way gain or lose financially from the publication of this manuscript, either now or in the future?
- Do you hold or are you currently applying for any patents relating to the content of the manuscript?
- Have you received reimbursements, fees, funding, or salary from an organization that holds or has applied for patents relating to the content of the manuscript?
- Do you have any other financial competing interests?
- Do you have any non-financial competing interests in relation to this paper?

If you can answer no to all of the above, write 'I declare that I have no competing interests' below. If your reply is yes to any, please give details below.

I declare that I have no competing interests

I agree to the open peer review policy of the journal. I understand that my name will be included on my report to the authors and, if the manuscript is accepted for publication, my named report including any attachments I upload will be posted on the website along with the authors' responses. I agree for my report to be made available under an Open Access Creative Commons CC-BY license (http://creativecommons.org/licenses/by/4.0/). I understand that any comments which I do not wish to be included in my named report can be included as confidential comments to the editors, which will not be published.

I agree to the open peer review policy of the journal

To further support our reviewers, we have joined with Publons, where you can gain additional credit to further highlight your hard work (see: https://publons.com/journal/530/gigascience). On publication of this paper, your review will be automatically added to Publons, you can then choose whether or not to claim your Publons credit. I understand this statement.

Yes Choose an item.
