# Supplementary material for: Dissecting glioblastoma risk signatures in the tumor immune microenvironment based on multi-dimensional transcriptomics
Source: Gigascience. 2026 Mar 25;15:giag035. doi: 10.1093/gigascience/giag035 (PMC13154832; doi:10.1093/gigascience/giag035)
Supplement: giag035_Reviewer_3_Report_original_submission — Reviewer 3 -- 2/28/2026 [file giag035_reviewer_3_report_original_submission.pdf]

Reviewer Report

Title: Dissecting glioblastoma risk signatures in the tumor immune microenvironment based on multi-dimensional transcriptomics

Version: Original Submission **Date:** 2/28/2026

Reviewer name: Reviewer 3

Reviewer Comments to Author:

This manuscript elucidates multiple immune escape mechanisms in glioblastoma through integrative analysis of single-cell, bulk, and spatial transcriptomics, and identifies seven hallmark-related prognostic signatures using machine learning algorithms. The overall analytical logic is clearly articulated and well-structured. However, several critical issues must be addressed prior to consideration for publication.

1. The abstract mentions the pivotal roles of hallmarks of malignancy and cell cycle regulatory pathways in transcriptional regulation, yet fails to specify the exact mechanisms or functions. A concise description of these critical roles would enhance clarity and impact.
2. At present, the abstract appears disconnected without clear transitional logic; revision to establish explicit linkages between sentences is recommended.
3. The keywords should represent the core, recurrent themes throughout the entire study rather than terms mentioned only sporadically at the beginning or conclusion.
4. In the Introduction, the authors identify multiple factors contributing to the poor therapeutic outcomes in glioma, among which the 'characteristics of the tumor's physical microenvironment' is vaguely delineated and lacks conceptual precision.
5. Within the subsection 'Immune phenotype of tumors based on transcriptomic profiling,' the initial two sentences comprise background material that is incongruous with the Methods section. The authors are advised to relocate this content to the Introduction to preserve the logical organization of the manuscript.
6. In the Results section, certain findings lack corresponding textual description; for instance, Figure 1B is presented without adequate characterization in the narrative.
7. The Methods describe T cells and B cells as reference populations for inferring copy number variation, whereas the Results define highly malignant cells as those with above-average CNV scores within two neuronal subtypes. The former strategy is conventionally accepted; however, the latter lacks explicit methodological justification. Furthermore, the relationship between these two approachesâ€"whether complementary or conflictingâ€"requires detailed elucidation.
8. In Results section 2, the authors report that B-cell infiltration does not differ significantly between immune-hot and immune-cold tumors. Does this observation suggest that B cells may play a dual or context-dependent role within the GBM microenvironment? The authors are encouraged to provide further interpretation and discussion to clarify this point.
9. The authors should explicitly define the 'combined score' depicted in Figure 4K, including its constituent variables and the algorithm or formula used for its calculation.
10. The T cell differentiation trajectory in Results section 5 appears poorly defined in two-dimensional

space. Please provide additional annotation to clarify the inferred developmental path.

Methods

Are the methods appropriate to the aims of the study, are they well described, and are necessary controls included? Choose an item.

Conclusions

Are the conclusions adequately supported by the data shown? Choose an item.

Reporting Standards

Does the manuscript adhere to the journal's guidelines on minimum standards of reporting? Choose an item.

Choose an item.

Statistics

Are you able to assess all statistics in the manuscript, including the appropriateness of statistical tests used? Choose an item.

Quality of Written English

Please indicate the quality of language in the manuscript: Choose an item.

Declaration of Competing Interests

Please complete a declaration of competing interests, considering the following questions:

- Have you in the past five years received reimbursements, fees, funding, or salary from an organisation that may in any way gain or lose financially from the publication of this manuscript, either now or in the future?
- Do you hold any stocks or shares in an organisation that may in any way gain or lose financially from the publication of this manuscript, either now or in the future?
- Do you hold or are you currently applying for any patents relating to the content of the manuscript?
- Have you received reimbursements, fees, funding, or salary from an organization that holds or has applied for patents relating to the content of the manuscript?
- Do you have any other financial competing interests?
- Do you have any non-financial competing interests in relation to this paper?

If you can answer no to all of the above, write 'I declare that I have no competing interests' below. If your reply is yes to any, please give details below.

I declare that I have no competing interests

I agree to the open peer review policy of the journal. I understand that my name will be included on my report to the authors and, if the manuscript is accepted for publication, my named report including any attachments I upload will be posted on the website along with the authors' responses. I agree for my report to be made available under an Open Access Creative Commons CC-BY license (http://creativecommons.org/licenses/by/4.0/). I understand that any comments which I do not wish to be included in my named report can be included as confidential comments to the editors, which will not be published.

I agree to the open peer review policy of the journal

To further support our reviewers, we have joined with Publons, where you can gain additional credit to further highlight your hard work (see: https://publons.com/journal/530/gigascience). On publication of this paper, your review will be automatically added to Publons, you can then choose whether or not to claim your Publons credit. I understand this statement.

Yes Choose an item.
